# Ancient Traces of Tailless Retropseudogenes in Therian Genomes

**DOI:** 10.1093/gbe/evv040

**Published:** 2015-02-26

**Authors:** Angela Noll, Carsten A. Raabe, Gennady Churakov, Jürgen Brosius, Jürgen Schmitz

**Affiliations:** ^1^Institute of Experimental Pathology, ZMBE, University of Münster, Germany; ^2^Institute of Evolutionary and Medical Genomics, Brandenburg Medical School, Neuruppin, Germany; ^3^Institute of Evolution and Biodiversity, University of Münster, Germany

**Keywords:** LINE1, SINEs, tailless retropseudogenes, relaxed LINE1 retrotransposition, 5S rRNA, U2, integration site complementarity

## Abstract

Transposable elements, once described by Barbara McClintock as controlling genetic units, not only occupy the largest part of our genome but are also a prominent moving force of genomic plasticity and innovation. They usually replicate and reintegrate into genomes silently, sometimes causing malfunctions or misregulations, but occasionally millions of years later, a few may evolve into new functional units. Retrotransposons make their way into the genome following reverse transcription of RNA molecules and chromosomal insertion. In therian mammals, long interspersed elements 1 (LINE1s) self-propagate but also coretropose many RNAs, including mRNAs and small RNAs that usually exhibit an oligo(A) tail. The revitalization of specific LINE1 elements in the mammalian lineage about 150 Ma parallels the rise of many other nonautonomous mobilized genomic elements. We previously identified and described hundreds of tRNA-derived retropseudogenes missing characteristic oligo(A) tails consequently termed tailless retropseudogenes. Additional analyses now revealed hundreds of thousands of tailless retropseudogenes derived from nearly all types of RNAs. We extracted 2,402 perfect tailless sequences (with discernible flanking target site duplications) originating from tRNAs, spliceosomal RNAs, 5S rRNAs, 7SK RNAs, mRNAs, and others. Interestingly, all are truncated at one or more defined positions that coincide with internal single-stranded regions. 5S ribosomal and U2 spliceosomal RNAs were analyzed in the context of mammalian phylogeny to discern the origin of the therian LINE1 retropositional system that evolved in our 150-Myr-old ancestor.

## Introduction

Long interspersed element 1 (LINE1 or simply L1) autonomous retrotransposons evolved less than 450 Ma in the common ancestor of deuterostomes, a clade comprising Echinodermata and Chordata, and, over time, gave raise to more than 300 diverse element families and a plethora of subfamilies (Kordis et al. 2006). Frequently, the 3′-untranslated region (3′-UTR) of transcribed L1 mRNA forms characteristic structural stem loops that are recognized by the associated enzymatic machinery, thereby leading to element-specific retroposition (Hayashi et al. 2014). However, in some cases, similar structures can favor coretroposition of “free-riders,” usually abundant cellular RNAs that are devoid of their own enzymatic equipment and in turn may evolve into highly repetitive short interspersed elements (SINEs). Such tight association of autonomous and specific nonautonomous elements that harbor, for example, LINE-like tails, is characteristic for many SINEs. For instance, Bov-tA SINEs of ruminants share identical RNA 3′-termini with the associated autonomous BovB LINEs (Ohshima and Okada 2005).

In the reduced population of mammalian progenitors ∼150 Ma, a significant loss of L1 diversity occurred (Kordis et al. 2006), leading to a complete inactivation of L1 elements in monotremes and to only a single active lineage in therian mammals (comprising marsupials and placentals). However, this single surviving LINE family, presumably due to lack of competition, became highly active and caused an explosive radiation of retrotransposons in placentals, with an especially high activity peak in primates and rodents (e.g., around 500,000 copies in the human genome compared with 96,000 LINE-derived CR1 elements in chicken or only 4,000 LINEs in the pufferfish fugu; Aparicio et al. 2002; Wicker et al. 2005; Mandal and Kazazian 2008). One defining characteristic of the new L1 lineage in therians was the replacement of the stringent (structural) mode of mRNA recognition, apparently by a simple oligo(A) recognition of the element mRNA terminus (Ohshima 2012), and a specific consensus insertion site TT/AAAA in the genomic target DNA (Jurka 1997). Similar to previously active LINE elements, the new lineage still preferred *cis*-retrotransposition of their own RNA, but also retrotransposed any RNAs equipped with at least an oligo(A) tail. This included many SINEs, such as Alu elements (Dewannieux et al. 2003), polyadenylated messenger RNAs (Esnault et al. 2000), and other adenylated cytoplasmic or nuclear RNAs. Interestingly, the relaxed 3′-end recognition of L1 is not restricted to the mammalian lineage but was also found in some sauropsids and some plants (Kordis et al. 2006; Ohshima 2012).

The full-length form of the human L1 element is ∼6-kb long and its transcript harbors a 900-nt 5′-UTR comprising internal RNA polymerase II (pol II) promoter sequences and an ∼300-nt-long polyadenylated 3′-UTR. The two open reading frames (ORFs) encode a functionally largely, as yet, uncharacterized but essential RNA binding protein with nucleic acid chaperon activity (ORF1) (Hohjoh and Singer 1996; Martin and Bushman 2001; Martin, 2006; Goodier et al. 2013) and a protein with two different domains encoding the endonuclease (EN) and reverse transcriptase (RT) (ORF2) (Goodier and Kazazian 2008). Both proteins are involved in *cis*-retroposition of their own or associated nonautonomous elements (Moran et al. 1996). Interestingly, the ORF1 protein is not required for efficient *trans*-retroposition (coretroposition) of nonautonomous elements (Hulme et al. 2007; Wallace et al. 2008).

**Fig. 1.—:**
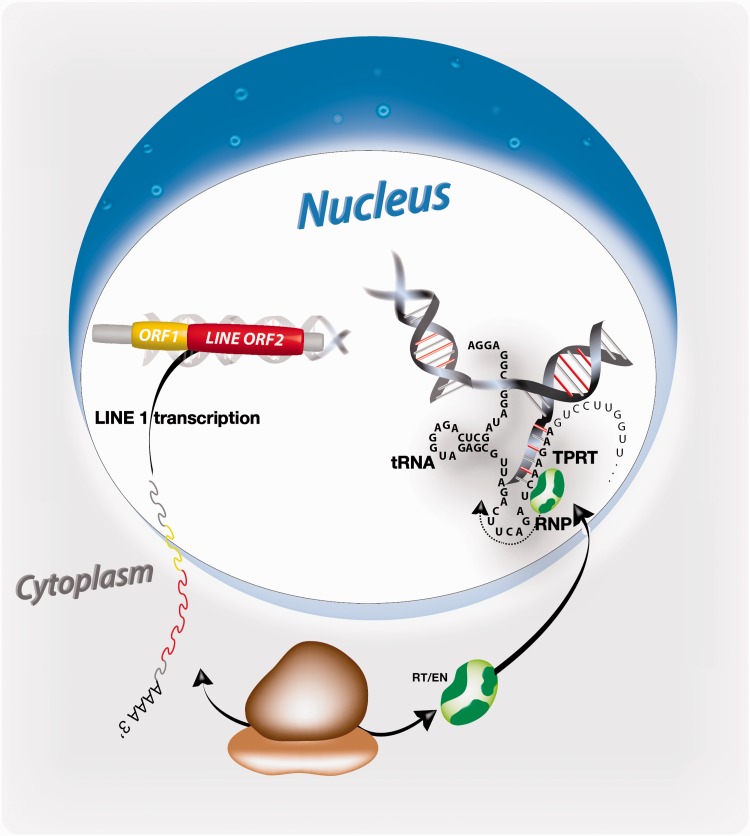
Process of TPRT of a tailless tRNA. The transcribed LINE1 mRNA migrates to the cytoplasm and is translated on ribosomes yielding the retrotranspositionally active protein, including RT/EN. *Cis*-attached RNA of the LINE1 element or alternative/additional suitable template RNAs form an RNP complex that is actively transported back into the nucleus. There, LINE1-encoded RNA components can be replaced by, for example, a tRNA. tRNA internal complementary regions may be used to integrate a part of the tRNA into the genome.

The molecular mechanism of LINE retrotransposition, known as target primed reverse transcription (TPRT), mediates the reverse flow of genetic material from cellular RNA back to the genome (fig. 1). Active L1 elements release its mRNA into the cytoplasm where ribosomes translate both the ORF1-encoded binding protein and the ORF2-encoded RT/EN. The newly synthesized proteins migrate back into the nucleus as ribonucleoprotein (RNP) particles occasionally decorated with cytoplasmic RNAs. Alternatively, the proteins are charged with RNA in the nucleus. The L1-encoded EN nicks the chromosomal DNA target sites and exposed 3′-hydroxyl termini serve as primer to enable reverse transcription. A second strand nick occurs at an 8–30 nt staggered position, which causes the typical target site duplications (TSDs) at the flanks of almost all retroposed elements (Luan et al. 1993). However, the RT/EN machinery can also utilize pre-existing nicks to prime reverse transcription, whereby blunt-end breaks seem to be more efficient than overhanging ends (Cost et al. 2002). Widespread in mammals and represented by nearly 2,100 copies in the human genome is an additional truncated, actively transcribed L1 population called “half L1s” or HAL1s encoding only the ORF1 with a poly(A) tail and flanked by TSDs (Bao and Jurka 2010). Similar to SINEs, HAL1s depend on the RT/EN of full-length LINEs.

**Fig. 2.—:**
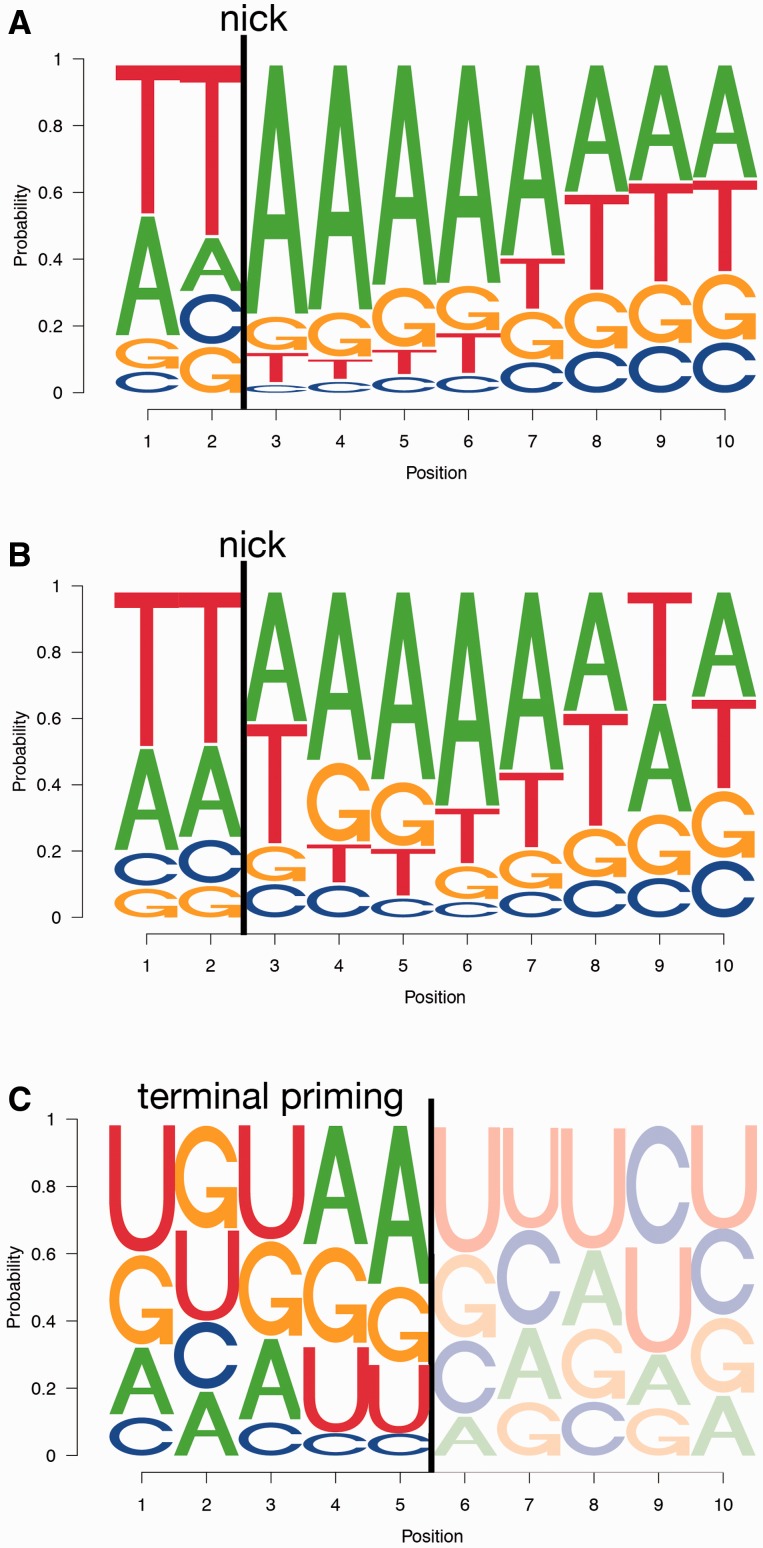
Sequence logos of retroposition target sites. (*A*) Sequence logos for 1,183 randomly chosen LINE1/Alu target sites for elements with perfect TSDs. (*B*) Sequence logos for 1,183 tailless retropseudogenes with perfect TSDs. (*C*) Sequence logos for endpoints of the 1,183 tailless retropseudogenes. The consensus refers to the largest letters in the logo (top).

The oligo(A) tail length of pol III transcripts appears to be crucial for successful retroposition events (Roy-Engel et al. 2002). In contrast, a decade ago we discovered hundreds of retroposed 3′-truncated tRNAs, processed or unprocessed and devoid of any terminal oligo(A) tail that were termed tailless retropseudogenes (Schmitz et al. 2004). The truncation typically mapped to RNA structural loops or other single-stranded regions and the genomic insertion site was complementary to the terminal 2–18 nt of the annealed RNA, which reveals a consensus target site somewhat different from the common TT/AAAA motif (fig. 2*A* and *B*). Besides all mechanistic requirements, element-specific activity patterns and population structures, two major factors influence the occurrence and frequency of inheritable genomic tailless retropseudogenes: 1) The germline expression rate of source RNAs and 2) the activity of the relaxed L1 element that occasionally compete for limited host factors with other autonomous retrotransposons. At least two probably active retropositional systems are known in cow, elephant, and marsupials (L1, RTE). In lizards, many different systems compete for retrotranspositional dominance (L1, L2, CR1, RTE, R4). In the human genome, L1s are the only active autonomous LINE retrotransposons, and nearly 100 of them are still capable of retrotransposition (i.e., full-length elements with intact promoters and ORFs). Activity assays have demonstrated that six source loci produce more than 80% of all retrocopies and are classified as hot L1s (Brouha et al. 2003). Tailless retropseudogenes were originally considered to represent only a minor population of short sequences that are occasionally inserted in the genome (Schmitz et al. 2004). With the availability of numerous genomes, we are now able to conduct mammalian-wide screens for tailless retropseudogenes, to analyze their distribution patterns and structures, to explore their sources, to trace their phylogenetic origins, and to expose the mechanistic principles behind this “by-product” of the L1 retropositional system.

## Materials and Methods

### Database of RNA Genes

To detect different types of tailless retropseudogenes genome wide, we chiefly screened the human genome for all known small RNAs, ribosomal RNA genes, and some mRNA subsets, including LINEs, histone, monoexonic mRNA, and housekeeping genes, compiled in a user-defined library using the local version of RepeatMasker (http://www.repeatmasker.org/RMDownload.html, last accessed March 11, 2015). Human sources included predicted tRNAs from the Genomic tRNA Database (http://gtrnadb.ucsc.edu/, last accessed March 11, 2015; hg19, 506 tRNAs), LINEs (repbase Issue 1, 2011), histones, monoexonic and housekeeping mRNAs (supplementary file S2, Supplementary Material online), 18S rRNA (K03432), 28S rRNA (M11167), 5.8S rRNA (U13369), 5S rRNA (X51545), Y RNAs (hY1, V00584; hY3, www.girinst.org, last accessed March 11, 2015), 7SK RNA (NR_001445.2), U1, U4, U5, U6, U7, and U13 (repbase Issue 1, 2011), U2 (K03022), U4atac (NR_023343.1), U6atac (NR_023344.1), U11 (NR_004407.1), U12 (NR_029422.1), snoRNAs U3, U8, U14, U17 (repbase Issue 1, 2011), and others from snoRNAbase (Lestrade and Weber, 2006). For all other species (supplementary table S8, Supplementary Material online), the sources of 5S rRNA and U2 are listed in the supplementary tables S9 and S10, Supplementary Material online. In cases where consensus sequences were not available, we generated a Consensus60 sequence after BLASTN searches for full-length elements with the 5S rRNA or U2 snRNA sequence of the closest relative species or the vertebrate consensus sequence from the RepeatMasker library (http://www.girinst.org/, last accessed March 11, 2015) as input.

### Python Script for Screening and Filtering Most Perfect Tailless Forms

With a designed Python script (tailless.py; supplementary fig. S1, Supplementary Material online; script available after request), we filtered the RepeatMasker results for 5′-(almost) complete (5-nt nonmatching sequences/overhangs were allowed) and 3′-truncated (at least 10 nt) hits flanked by 8–35 nt TSDs (with at least 70% sequence identity) and a target sequence identity to the consensus sequences of more than 69%. To further compensate for random mutations, we allowed a 15 nt gap between the 3′-element terminus and the TSD start. The minimal length of a tailless retropseudogene was set to 30 nt. For searches of tRNA-derived tailless retropseudogenes, we used more relaxed conditions that permit 20 nt between the 5′-terminal TSD and the element start in order to account for potential precursor sequences.

The “tailless” Python script used different kinds of alignment generations. The local Smith Waterman algorithm (http://fsbao.net, last accessed March 11, 2015) was used to identify TSDs according the following settings: Gap penalty −25, match +8, mismatch −17, and applying the substitution matrix S11 (supplementary table S11, Supplementary Material online). In comparison, the global Needleman Wunsch algorithm was employed for alignment of the tailless RNA hits with the corresponding source RNA. Here, the following settings were elaborated: Match +7, mismatch −5, gap opening penalty −25, gap extension penalty −3, and applying the substitution matrix S12 (supplementary table S12, Supplementary Material online). From the ∼2,500 human hits with TSDs, we eliminated all multiple hits of the same genomic region (e.g., multiple tRNA hits) and genomic duplications. A genomic duplication was identified in cases where TSDs and the ∼100-nt flanking sequences were 70% or more identical. The Python script relied on the same Smith Waterman settings introduced above. Interrupted elements wrongly assigned as tailless elements were detected and excluded by screening the RepeatMasker output files for identical repeat-specific IDs. Finally, we received 2,402 clear instances of tailless retropseudogenes from different reverse-transcribed RNAs. The same procedure was applied to screen all other vertebrate genomes and plants in this study. To ensure data comparability, the number of tailless retropseudogenes per genome was normalized per gigabase genome sequence.

### Screening for Oligoadenylated Truncated RNAs

To uncover possible RNA pol III-transcribed (tailless) retropseudogenes with additional, so far unknown, polyadenylation, we used the human RepeatMasker output (hg19; RepeatMasker Library db20140131; http://www.repeatmasker.org/genomicDatasets/RMGenomicDatasets.html, last accessed March 11, 2015) to search for 5S rRNA and U2 snRNA hits (full-length and tailless) flanked by simple repeats or low complexity regions at respective 3′-ends.

### Analysis of TSDs

To compute the minimum and maximum priming length of the tailless elements with target sites, we used all 2,454 human tailless elements (including genomic duplications), extracted the corresponding priming sites (if available), and calculated their respective length. The common target site pattern of LINE and SINE elements, so far described as TT/AAAA (Jurka 1997), was recalculated with the human (hg19; RepeatMasker Library db20140131) and mouse (mm10; RepeatMasker Library db20140131) RepeatMasker outfile downloaded from the RepeatMasker website (http://www.repeatmasker.org/genomicDatasets/RMGenomicDatasets.html, last accessed March 11, 2015), using only elements with 100% identical TSDs. To enable unbiased comparison of both 3′-full-length and 3′-tailless elements (limited to 1,183, 3′-tailless elements with 100% identical TSDs), we utilized identical numbers of either case. Thus, we screened RepeatMasker output files for 1,183 randomly selected LINE/L1 and SINE/Alu repeats (human), and LINE/L1 and B1 elements (mouse). The designed Python script (TSDfinder.py) permits an overlap with the elements 3′-end of maximally 20 nt as a priming site. The length of TSDs ranged between 8 and 35 nt. There was no discrimination of a minimal or maximal insert length, but the 3′-ends were required to be not truncated (maximally 9 nt of nonmatching sequence was allowed). To calculate the target site pattern, 2 nt upstream of the 5′-TSD and the 5′-TSD itself were considered. After counting the number of nucleotides for each position, the connected frequency site distribution was calculated to build a sequence logo motive with the help of the R seqLogo package (Bembom 2014). The calculation of the common target site pattern of the tailless elements was conducted identically except that we directly used the output alignments from the “tailless Python script.”

### Breakpoint Patterns

To calculate the “breakpoint” patterns, we used all human tailless elements with 100% identical TSDs and their corresponding RNA targets. We selected regions 5 nt upstream and downstream relative to the breakpoint within the target RNA and counted the frequency of each nucleotide per position. The depiction as sequence logo was conducted as described above.

### Calculating the Number of Full-Length Versus Tailless Retropseudogenes

To compare the numbers of tailless and full-length L1-dependent integrations, we masked the human genome for L1 elements (extracted from the RepeatMasker library; http://www.girinst.org/ last accessed March 11, 2015), and then screened the outfile for 4′-full-length (maximally 5 nt of terminal nonmatching sequence) and 5′-truncated (at least 5 nt of nonmatching sequences), 3′-tailless (at least 10 nt of nonmatching sequences) and 3′-full-length (maximally 9 nt of nonmatching sequences) elements with the TSDfinder Python script introduced above. Furthermore, we demanded sequence identities of at least 70% for the respective TSDs.

### Truncated RNA Genes

We generated a human cDNA library for reverse-transcribed short RNAs (10–600 nt) corresponding to the methods described in Schmitz et al. (2008). The ∼60,000 experimentally derived cDNAs (data not shown) were screened for 3′-truncated 5S rRNA and U2 snRNA sequences whose 3′-ends corresponded to the breakpoints of tailless retropseudogenes using the RepeatMasker (-e crossmatch <RNA-seq-library> -lib <human-RNAs>). After recovering RNA-seq-hits of 5S rRNA and U2 snRNA starting at position 1, we selected the endpoints and counted their frequencies within the data set. The tailless Python script with relaxed conditions at the 5′-end (i.e., all hits with more than 5 nt of nonmatching sequence at the 5′-end were allowed) was used to recover the 5′-truncated and 3′-tailless forms of 18S and 28S rRNAs as well as histones in the human genome.

### Full-Length L1 Screening

**Fig. 3.—:**
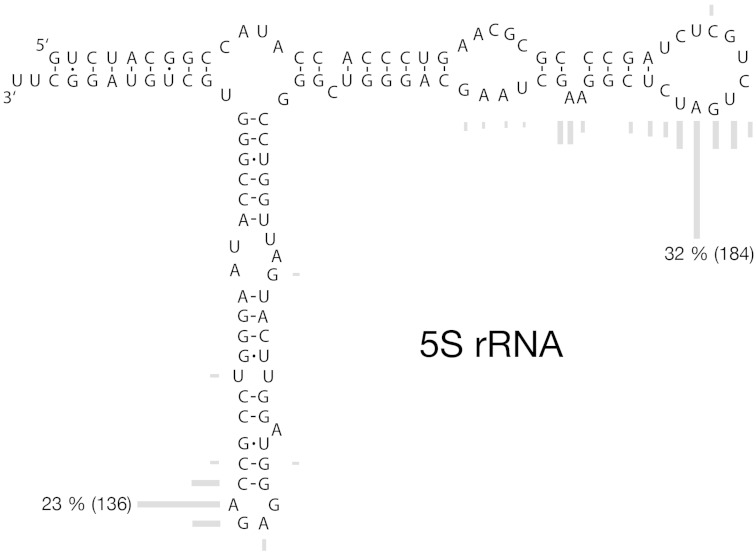
Secondary structure of 5S rRNA and truncation hotspots. Gray bars represent the relative numbers of truncated fragments ending at the corresponding nucleotide (in total 555 5S rRNA tailless retropseudogenes). Comparable with tRNAs (Schmitz et al. 2004), most truncations in 5S rRNA appear in loops or other single-stranded regions.

For figure 3, only uninterrupted (potentially active) full-length L1 and HAL1 elements were considered (maximally 20 nt of nonmatching sequence at each of the 5′- and 3′-ends). To detect these elements, we used RepeatMasker output files (http://www.repeatmasker.org/genomicDatasets/RMGenomicDatasets.html, last accessed March 11, 2015; RepeatMasker Library 4.0.5 20140131) of human (hg19), mouse (mm10), dog (canFam3), cow (bosTau7), sloth (choHof1), elephant (loxAfr3), tenrec (echTel2), manatee (triMan1), rock hyrax (proCap1), opossum (monDom5), wallaby (macEug2), Tasmanian devil (sarHar1), platypus (ornAna1), chicken (galGal4), and lizard (anoCar2).

### Genome-Wide Presence/Absence Screening

We used the online genome-wide “Presence/Absence Compiler” (GPAC tool, http://www.bioinformatics.uni-muenster.de/tools/gpac, last accessed March 11, 2015; Noll et al. 2015) to analyze the presence/absence patterns of solitary ERV–LTRs and tailless retropseudogenes based on the vertebrate 46-way alignment with human as the leading species. The presence/absence patterns were analyzed for all primates and tree shrew as outgroup. For solitary ERV–LTRs, we used the coordinates of all full-length element (less than 10 nt of nonmatching sequence at the 5′- and 3′-ends) hits retrieved from the human RepeatMasker outfile (http://www.repeatmasker.org/genomicDatasets/RMGenomicDatasets.html, last accessed March 11, 2015).

## Results and Discussion

### Generation of Tailless Retropseudogenes

Tailless retropseudogenes also use TPRT for retroposition, but presumably are primed internally at loops or other single-stranded regions of source RNAs (fig. 1; see below), with a preference for adenosine-rich internal sequences. The genomic target sites show complementarity to the regions of the source RNA that constitutes the 3′-end of the tailless element. The corresponding consensus target site that we derived is TT/WAAAAWWX_(0__–__17)_ (fig. 2*B*), which differs slightly from the canonical TT/AAAA (Jurka 1997) L1 consensus target sequence (fig. 2*A*). The latter sequence was reconfirmed by random extraction and analysis of equal amounts of L1 and Alu elements with perfect TSDs and full-length 3′-ends (1,183 cases). Interestingly, the consensus sequence for tailless retropseudogene insertions carries an A or T (W) nucleotide at the break position and continues with four A’s and two additional W’s. X_(0__–__17)_ represents a more heterogeneous complementarity of the insertion site internally base paired with the corresponding template RNA. The variant motifs suggest that the processes of retroposition of 3′-oligoadenylated RNAs and tailless retropseudogenes vary somewhat between classical and tailless retroposition.

### Breakpoints or Internal Priming?

In general, there are two entirely different scenarios that might lead to the generation of tailless retropseudogenes with preferential 3′-endpoints (fig. 3; 5S rRNA tailless retropseudogenes): 1) The integration of enzymatically truncated source RNAs or 2) the internal priming of A-rich regions of source RNAs (fig. 2*C*). On the basis of the assumption that truncated RNAs resulting from endonucleolytic processing or degradation represent bona fide targets for RNA polyadenylation and subsequent retroposition, we scanned the entire human genome for 5S rRNA and U2-derived retropseudogenes. The corresponding retropseudogenes should then harbor oligo(A) tails at sites of theoretical truncations. However, we failed to identify any convincing signals consistent with truncated RNAs serving as intermediates for tailless retropseudogene generation. Furthermore, we unsuccessfully scanned human small RNA transcriptome libraries for 5S rRNA and U2 RNA-derived cDNAs, whose 3′-termini would be consistent with the truncation of source RNAs as templates for tailless major breakpoint (data not shown). By default, this strongly suggests internal priming as the actual scenario.

Internal priming of dimeric Alu SINEs could well explain the “revived” activity of long-extinct Alu monomeric elements (Kojima 2011). Instead of contributions from “undead” sources, many monomers may have originated from still active dimeric Alu transcripts. In this case, however, internal priming events are hard to distinguish from simple recombination events involving the terminal and internal oligo(A) stretches and leaving behind the TSDs. Nevertheless, our discovery of 38 seemingly monomeric Alu elements also contain a few proximal nucleotides of the right monomer corresponding to the internal priming site of tailless retropseudogenes and strengthens our interpretation (supplementary table S1, Supplementary Material online).

**Fig. 4.—:**
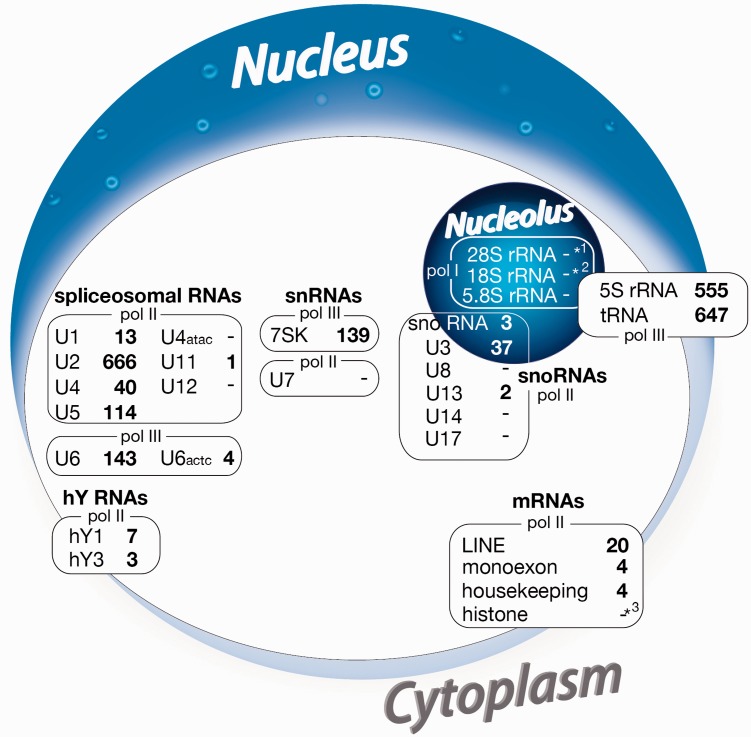
Sources of 2,402 human tailless retropseudogenes. A list of various RNAs from which the selected human tailless retropseudogenes were derived. The asterisks denotes 10 (*^1^), 2 (*^2^), and 51 (*^3^) tailless forms that we found in 5′-truncated 28S, 18S rRNA, and histone sequences, respectively. Boxes represent the sub-cellular locations (partially overlapping) of the source genes. pol I-transcribed nucleolar rRNA tailless retropseudogenes are underrepresented. Both pol II- and pol III-derived RNAs possessing a significant nuclear phase vary in the number of derived tailless retropseudogenes.

There is an additional trace in our data set indicating that a terminal oligo(A) tail is not essential for retrotransposition. For example, RNA pol III-transcribed 7SK RNA, which participates in the control of RNA pol II transcription during elongation, is not polyadenylated but was the source of 139 tailless retropseudogenes within our data set (fig. 4 and supplementary table S2, Supplementary Material online). Furthermore, we detected 51 5′-truncated tailless histone retropseudogenes derived from mRNAs, known for their terminally nonpolyadenylated mRNAs (supplementary table S3, Supplementary Material online).

### Cellular Localization for L1-Binding of Tailless Source RNAs

Usually, the interaction of RT/EN protein and L1 mRNA constituting the RNP complex competent for retrotransposition takes place within the cytoplasm. This is also the bona fide site for the recruitment of SINE source RNAs related to tRNA, 5S rRNA, and 7SL RNA, such as, for example, B2, ID, B1, and Alu. Such SINE transcripts are significant sources for tailless retropseudogenes as well (data not shown). It is expected that binding of source RNA during LINE RNP formation might preferentially occur within the cytoplasm. However, our data revealed that nuclear RNAs, such as U6 RNA (Köhler and Hurt 2007), unprocessed tRNAs (Schmitz et al. 2004), and 7SK RNA (Peterlin et al. 2012), which supposedly are restricted to the nucleus, are well represented as tailless retropseudogenes and therefore should bind to the LINE RNP in the nucleus (fig. 1). In comparison, except for two 5′-truncated 18S rRNAs, ten 5′-truncated 28S rRNAs, and 42 snoRNA-derived tailless retropseudogenes (fig. 4 and supplementary tables S2 and S3, Supplementary Material online), we found no tailless element related to the highly abundant nucleolar RNAs.

### Phylogenetic Distribution of Tailless Retropseudogenes in Primates

**Fig. 5.—:**
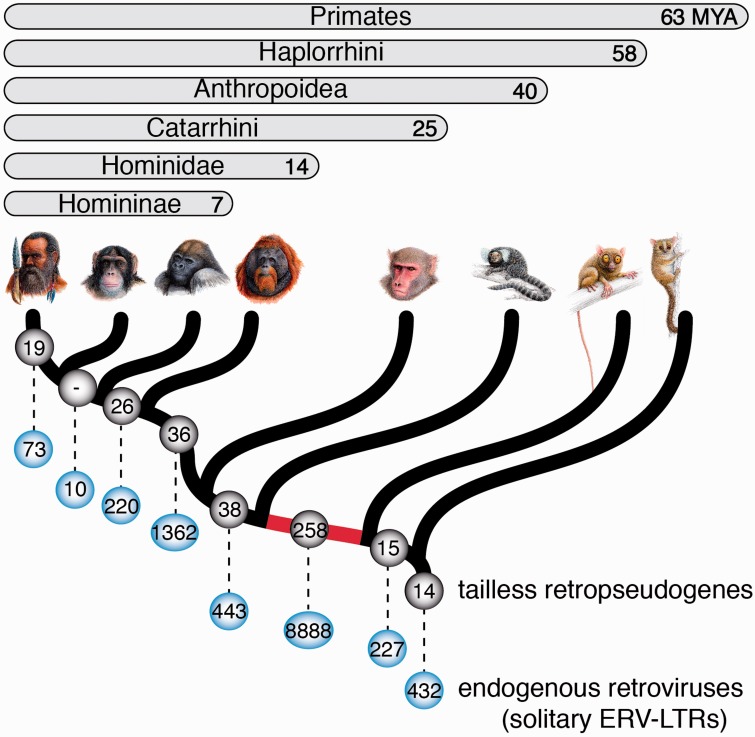
Phylogenetic distribution of tailless retropseudogenes and endogenous retroviruses in primates. The number of tailless retropseudogenes and endogenous retroviruses are represented in gray and blue balls, respectively. The red internal branch leading to Anthropoidea marks the hotspot of tailless retropseudogene as well as retroviral insertions. Branching dates are indicated as Ma (Goodman et al. 1998).

Three major factors determine the level of genomic fixation of tailless retropseudogenes in germ lines: 1) The expression rate of the source RNAs and their capacity to offer templates to retroposition over time, 2) the L1 activity over time, and finally 3) population structures and speciation events. Given the unique genomic information available for human and at least substantial genomic information for most other primate lineages, the ∼63 Myr of primate evolution (Goodman et al. 1998) are well suited for analyzing the temporal deposition pattern of tailless retropseudogenes. We propose that the temporal activity of L1, usually determined by sequence divergence data of different element subfamilies, can also indirectly be accessed from the temporal distribution of associated coretroposed elements. We developed a GPAC (Noll et al. 2015) to directly map retrotransposon insertions to internal branches of the well-defined phylogenetic tree of primates. The associated web tool (http://www.bioinformatics.uni-muenster.de/tools/gpac, last accessed March 11, 2015) derived presence/absence patterns for our 2,454 human tailless retropseudogenes (including genomic duplications; coordinates see supplementary table S2, Supplementary Material online). Selection of GPAC patterns with clear presence/absence boundaries in all investigated primates yielded 19 human-, 26 Homininae-, 36 Hominidae-, 38 Catarrhini-, 258 Anthropoidea-, 15 Haplorrhini-, and 14 primate-specific insertions (fig. 5 and supplementary table S4, Supplementary Material online). Figure 5 illustrates the detailed distribution patterns of tailless retropseudogenes and retroviral (endogenous retroviral, solitary ERV–LTRs) insertions. The data indicate a remarkable burst of tailless retropseudogene as well as solitary ERV–LTR insertions in the lineage leading to anthropoid primates. This perfectly agrees with data from Khan et al. (2006) and others indicative of explosive retrotransposon and processed pseudogene amplifications during the radiation of anthropoid primates (Goodier and Kazazian 2008). Some authors even suggest that the explosive radiation of retrotransposons may have facilitated speciation events and was a potential driving force during primate evolution (Ohshima et al. 2003). We tend to argue that the high activity and radiation of elements about 40–58 Ma were possibly associated with population bottlenecks and/or correlate with the long internal branch leading to anthropoid primates. This is supported by the fact that LINE-independent insertion events, such as endogenous retroviruses (ERV–LTRs) (fig. 5 and supplementary table S5, Supplementary Material online), display a similar distribution pattern as tailless elements.

### Distribution of Tailless Retropseudogenes in Vertebrates

**Fig. 6.—:**
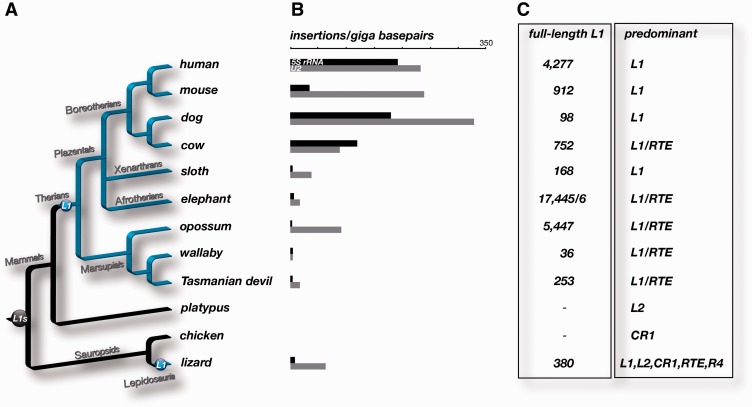
Vertebrate-wide distribution of tailless retropseudogenes correlates with the phylogenetic distribution of LINE1 retroposition. (*A*) Phylogenetic tree of representative vertebrates. L1 (in dark gray ball) represents the activity of LINE1 elements in all vertebrates. L1s (in blue balls) denotes the independent origin of the revitalized activity in therians and lizards. Blue branches indicate the evolutionary activity of the relaxed autonomous LINE1 system. (*B*) The bars represent the frequencies of 5S rRNA (black) and U2 (gray) tailless forms in the various vertebrate species. (*C*) The numbers of full-length LINE1 elements per species are indicated in the first column and the predominant active autonomous retrotransposons in the second column of the table.

Transcription and reverse transcription are the two major processes that any successful autonomous or nonautonomous master element has to undergo to be propagated. During the course of evolution, the activities of individual LINE and SINE subfamilies rise and fall in successive waves, due to, for example, changing measures of host defense and/or alteration of the retropositional efficiency in case of individual master genes. The presence of SINEs, processed retropseudogenes, and tailless retropseudogenes is a strong indication for successful L1 activity. With inactivation of the vigorous LINEs, the nonautonomous elements depending on them concomitantly are silenced. One prominent example of this dependency is a mammalian-wide interspersed repeat that coevolved with LINE3 elements and then ceased to retropose in mammalian genomes about 130 Ma concurrent with the “perishing” of the partner LINE3 system (Smit and Riggs 1995). L1 activity in the mega bat genome vanished about 24 Ma (Cantrell et al. 2008). Since then, L1-dependent partner SINEs, processed retropseudogenes, and tailless retroposed forms can be found only in older deposits of the fossilized history of the genome. The distribution patterns of tailless retropseudogenes, exemplified by 5S rRNA and U2 (fig. 6 and supplementary file S1 and tables S2 and S6, Supplementary Material online), perfectly agree with the activity range of L1 in some vertebrates and reach highest abundance in Boreotheria (represented by cow, dog, mouse, and human; fig. 6*A* and *B*). Currently, we have no explanation for the marked difference between 5S rRNA tailless retropseudogenes in mouse (90 occurrences absolute, 34 per gigabase) versus human (555 occurrences absolute, 194 per gigabase). The higher number of 5S rRNA tailless retropseudogenes in the guinea pig (414 occurrences absolute, 155 per gigabase, data not shown) resembles more closely that of human. Despite a low abundance of tailless retrotransposons, the elephant has an enormous number of lineage-specific full-length L1 elements (17,445; fig. 6*C*). In contrast, the relatively closely related manatee has only six full-length versions of L1 elements. In afrotherians, RTEs represent the dominant retrotranspositional system, as indicated by coretroposed AfroSINEs (Gogolevsky et al. 2008). In marsupials, both L1 and RTE systems were recently active, whereby the dominance of either system is lineage specific. For example, L1 was most active in the opossum. This is also discernable from the “transposition in transposition” pattern of L1- and RTE-derived SINEs (Zemann et al. 2013). There is no full-length LINE element in platypus or chicken and we failed to identify tailless retropseudogenes, hence supporting the strong dependency of tailless forms on the L1 retroposition machinery. Apart from mammals, the green anole lizard is the only other vertebrate lineage in which we detected tailless retropseudogenes. Therefore, we predict a similar relaxed L1 retropositional system in lizards, indicating similar recognition efficiency in some sauropsids. We speculate that the relaxed retroposing L1 lineage is one of the many different extinct deuterostomian L1s that survived and acquired dominance in a few animal lineages, including therian mammals, as evidenced by the appearance of retroposed tailless retropseudogenes. Tailless retropseudogenes further imply that the retropositional machineries of relaxed L1 elements are not restricted to the interaction with A-tails in template RNAs but that other RNA structures are necessary for their more or less efficient use as templates for retroposition. Although relaxed L1 retroposition is described for some plants (Ohshima 2012), we detected only eight U2-derived tailless retropseudogenes in *Zea mays* (supplementary file S1 and table S7, Supplementary Material online), indicating possible additional restrictions of random RNA retroposition.

### Frequency of Tailless Retropseudogenes

From a rough selection of about 450,000 human 3′-truncated tailless retropseudogene hits, we selected 2,402 clear cases with flanking TSDs (without genomic duplications) derived from various template RNAs (fig. 4). This selection is restricted to elements that are almost full-length with respect to the 5′-end of the template RNA. It includes most of the short tailless elements, but probably underrepresents the number of longer 5′-truncated tailless retropseudogenes genome wide, especially those derived from mRNAs. For an approximate estimate of tailless versus full-length mRNAs and randomly fragmented sequences, we calculated the number of full-length L1 elements (708), 3′-truncated (90), and 3′/5′-truncated (5,593) (data not shown) tailless forms (under relaxed screening conditions using the TSDfinder.py Python script; see Materials and Methods) all flanked by TSDs. The data revealed that the genome also harbors a much higher number of tailless forms of 5′- and 3′-truncated mRNAs and other long RNAs than presented in our restricted screening for 5′-full-length elements (fig. 4).

### Tailless Retropseudogenes or Tailless SINEs?

Singer first introduced the abbreviations SINEs and LINEs for short and long interspersed sequences, respectively, in 1982 (Singer 1982), and also referred to them as short or long repeated segments. The distinction was chiefly based on size, with an arbitrary cutoff at ∼500 nucleotides. A further important distinction was introduced later; namely that at least full-length LINEs, like endogeneous retroviruses, contain protein-coding genes essential for autonomous retroposition, while SINEs are nonautonomous and must rely on the LINE-encoded molecular retroposition machinery and even might require LINE-derived RNA structures for retro amplification (Singer 1990). As the vast majority of SINEs feature internal RNA pol III promoter elements (box A and box B), oligoadenosine stretches at 3′-ends and short TSDs at sites of genomic integration; these hallmarks inevitably were added to the definition of a SINE (Jagadeeswaran et al. 1981). Now that our knowledge about retroposition and its contributions to genome evolution is much advanced, it is time to consider whether some of these definitions may be too narrow. Just as SINEs were assumed to be highly repeated and arbitrarily defined as representing 10^4^ or more copies, today we know that retroposition is an ancient process, and that the mostly neutrally evolving products, including SINEs, LINEs, and mRNA-derived retrogenes, decay by mutational attrition over a time span of a few hundred million years. A few members of very ancient SINE families, however, persisted. For example, from the Amniota SINE1 family, about 1,000 copies are still discernible in the human genome, and no one questions, despite their relatively low copy number, that they are SINEs.

Reverse transcription is primed mainly by a recessed DNA end (3′ overhang) on RNA as a template, but it does not seem to matter whether this priming occurs on the oligoadenosine-rich 3′-end of a transcribed master SINE (preferred) or internally (on occasion), which leads to 3′-truncated “SINEs” that still feature TSDs. Copy numbers in the range of about 600 are reported for this latter group (Schmitz et al. 2004). Clearly, these are “tailless SINEs” in the sense of short elements; however, we were prompted to use the term tailless retropseudogenes for publication, as some of the more narrow criteria for SINE definition (an A-rich tail and internal promoter boxes) were not met. Also is it of importance whether an efficiently retroposed RNA template for a SINE amplification is transcribed only by RNA pol III? In platypus, for example, we described a composite master gene that features a small nucleolar RNA and as a second domain the 3′-end of an RTE LINE element (Schmitz et al. 2008). This domain makes the transcript a highly efficient template for retroposition and there are ∼40,000 copies in the genome of *Ornithorhynchus anatinus*. Usually, snoRNAs are transcribed by RNA pol II, often located in introns of protein-coding host genes and processed to yield the mature product.

Finally, in Tarsius, there are short interspersed sequences that are derived from a LTR77_TS element. The promoters feature a TATA box followed ∼80 nt downstream by a polyadenylation signal. These bona fide RNA pol II transcripts seem to be efficient templates for retroposition, yielding in the prosimian tarsier about 22,000 copies of the so-called TINE elements (http://www.girinst.org, last accessed March 11, 2015). Like so often in biology, borders are hard to delineate on a continuum, in this case also underscored by the blurred distinction of RNA pol II and III promoter elements (Murphy et al. 1989).

## Conclusion

The human genome and likewise the genomes of most mammalian relatives are inundated with insertions derived from or comobilized by L1-retroposed sequences. We suggest that tailless retropseudogenes (tailless SINEs) are L1 mobilized, 3′-truncated RNA descendants with an overlapping phylogenetic distribution to their L1 drivers. There are not only tRNA-derived elements, but also high copy derivatives of 5S rRNA, U2 RNA, 7SK RNA, and many more. Here, we describe 2,402 of the most unambiguous cases of tailless retroposons detected in the human genome. The abundance of different types of tailless retropseudogenes somewhat corresponds to the expected expression activity of their source RNAs. In primates, they accumulated most successfully in the common ancestor of anthropoid primates (64% of all clear presence/absence cases). A similar distribution occurred for independently derived ERV–LTR insertions, indicating that population bottlenecks and/or a long anthropoid ancestry are more probable causes of this lopsided accumulation than variation in L1 activity. Because our stringent search criteria were limited to perfect cases, we present only a small fraction of the actual number of tailless retropseudogenes. The large number of tailless retrotransposons confirms the previously debated process of generating 3′-truncated tailless retropseudogenes, and provides convincing arguments against the previously proposed strict necessity of oligo(A) tails for L1-mediated retrotransposition and instead exemplifies an extensive, somewhat adenosine-rich complementarity during internal priming. The process of generating tailless retropseudogenes dates back to the common Jurassic ancestor of therian mammals and to the evolution of a L1 retroposition machinery that is more relaxed with respect to template RNAs.

## Supplementary Material

Supplementary figure S1, tables S1–S12, and files S1 and S2 are available at *Genome Biology and Evolution* online (http://www.gbe.oxfordjournals.org/).

Supplementary Data
