# Supplementary material for: Ancient Traces of Tailless Retropseudogenes in Therian Genomes
Source: Genome Biol Evol. 2015 Feb 26;7(3):889–900. doi: 10.1093/gbe/evv040 (PMC5322556; doi:10.1093/gbe/evv040)
Supplement: Supplementary Data [file supp_7_3_889__index.html]

Ancient Traces of Tailless Retropseudogenes in Therian Genomes — Ancient Traces of Tailless Retropseudogenes in Therian Genomes — Supplementary Data 

# Ancient Traces of Tailless Retropseudogenes in Therian Genomes

## Supplementary Data

files

**Files in this Data Supplement:**

- Supplementary Data - docx file
- Supplementary Data - xlsx file
- Supplementary Data - docx file
- Supplementary Data - docx file
- Supplementary Data - docx file
