# Supplementary material for: Ancient Traces of Tailless Retropseudogenes in Therian Genomes
Source: Genome Biol Evol. 2015 Feb 26;7(3):889–900. doi: 10.1093/gbe/evv040 (PMC5322556; doi:10.1093/gbe/evv040)
Supplement: Supplementary Data [file supp_evv040_Figure_S1_and_Table_S1-S5.docx]

**
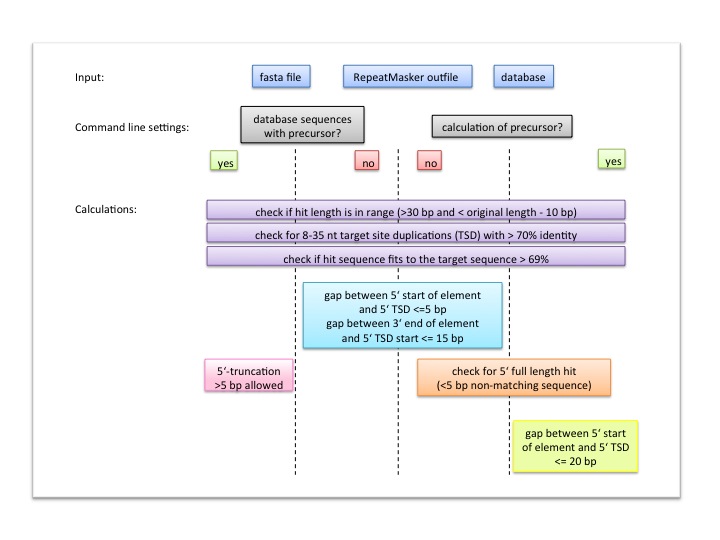
**

**Figure S1. Graphical flowchart of the Python script “tailless.py”**

After indicating the fasta file (containing the genomic sequences of the analyzed species) the RepeatMasker tabular outfile and the applied database (containing the sequences used for repeatmasking (e.g., 5S rRNA, U2 snRNA, etc.)), further settings can be applied, e.g., if the database contains sequences with precursors or if the tailless hits may include precursor sequences (command line settings). The default settings are in both cases “no”. Subsequently the script is searching for target site duplications (TSD) flanking the hits; and for special sequence identities between both TSDs and the original element sequences and the element hits within the genome. In a final step, the distance between the end of the 5’ TSD and the start of the genomic hit as well as the distance between the end of the genomic hit and the start of the 3’ TSD is calculated depending on the command line settings.

**Table S1. Tailless Alu SINE hits**

Listed are the coordinates and the Alu element names of tailless Alu SINEs truncated shortly after the element-internal oligoA-stretch.

| **Chr** | **Start** | **End** | **Type** |
| --- | --- | --- | --- |
| chr1 | 32984404 | 32984552 | AluJo |
| chr1 | 36407312 | 36407461 | AluJb |
| chr1 | 45079145 | 45079288 | AluJr |
| chr1 | 193545051 | 193545192 | AluJo |
| chr2 | 113512871 | 113513016 | AluSp |
| chr3 | 2192769 | 2192908 | AluSz6 |
| chr3 | 127758460 | 127758605 | AluSg |
| chr3 | 196420236 | 196420384 | AluY |
| chr7 | 45034284 | 45034422 | AluSc |
| chr7 | 137693835 | 137693985 | AluJo |
| chr8 | 37994637 | 37994782 | AluSx |
| chr8 | 61138401 | 61138550 | AluSc |
| chr9 | 26174802 | 26174951 | AluSc |
| chr10 | 76499165 | 76499294 | AluSz |
| chr11 | 26035568 | 26035715 | AluJb |
| chr11 | 57917785 | 57917925 | AluJb |
| chr12 | 8222046 | 8222191 | AluSx |
| chr12 | 17342364 | 17342514 | AluY |
| chr12 | 112791353 | 112791498 | AluSx4 |
| chr13 | 23880186 | 23880301 | AluJb |
| chr14 | 73535434 | 73535581 | AluSc |
| chr15 | 45228441 | 45228584 | AluSz |
| chr15 | 45236159 | 45236302 | AluSz |
| chr15 | 45874643 | 45874783 | AluJo |
| chr15 | 75262305 | 75262451 | AluSx |
| chr15 | 75839658 | 75839806 | AluSg7 |
| chr16 | 9021720 | 9021861 | AluJo |
| chr17 | 58388115 | 58388256 | AluJb |
| chr17 | 76231054 | 76231196 | AluSq10 |
| chr20 | 47758150 | 47758293 | AluSz |
| chr20 | 50849615 | 50849764 | AluY |
| chr19 | 7263763 | 7263898 | AluSx |
| chr19 | 42808490 | 42808622 | AluJb |
| chr19 | 48283383 | 48283514 | AluSx |
| chr19 | 56347067 | 56347206 | AluSz |
| chr22 | 39761857 | 39761988 | AluSz |
| chrX | 109115939 | 109116106 | AluSx |
| chrX | 113299712 | 113299852 | AluSz |

**Table S2. Human tailless retropseudogenes**

Listed are the coordinates of all perfect human tailless retropseudogenes (5’ full-length and flanked by TSDs)

| **chr** | | **start** | | **end** | | **type** |
| --- | --- | --- | --- | --- | --- | --- |
| **LINE1** | | | | | | |
| chr2 | 75819811 | | 75819883 | | HAL1ME | |
| chr2 | 166933493 | | 166933660 | | L1M3f | |
| chr3 | 110607662 | | 110609395 | | L1HS | |
| chr4 | 33333310 | | 33335924 | | L1PA15-16 | |
| chr4 | 171294259 | | 171294328 | | L1M2a1 | |
| chr5 | 123294087 | | 123294265 | | L1ME4b | |
| chr5 | 33903095 | | 33904295 | | L1PBa1 | |
| chr5 | 42076422 | | 42077393 | | L1PA12 | |
| chr5 | 78233598 | | 78234065 | | L1M3f | |
| chr6 | 93574279 | | 93575068 | | L1MCa | |
| chr6 | 77585477 | | 77586792 | | L1HS | |
| chr7 | 47766558 | | 47766827 | | L1MDa | |
| chr7 | 88210623 | | 88210778 | | L1M3d | |
| chr9 | 10573484 | | 10573699 | | L1PA13 | |
| chr10 | 131009860 | | 131009915 | | L1M6 | |
| chr11 | 48451615 | | 48452158 | | L1PA12 | |
| chr11 | 51517818 | | 51518361 | | L1PA12 | |
| chr13 | 55146370 | | 55147256 | | L1HS | |
| chr16 | 58979225 | | 58979281 | | L1M3c | |
| chr16 | 88917795 | | 88919214 | | L1P2 | |
| chrX | 98879241 | | 98879322 | | L1MCa | |
| **Mono-exonic retropseudogenes** | | | | | | |
| chr11 | 1687238 | | 1687381 | | NM_001012708 | |
| chr11 | 1706281 | | 1706424 | | NM_001012708 | |
| chr18 | 66010223 | | 66010271 | | NM_001007249 | |
| chr20 | 20732036 | | 20732087 | | NM_001004706 | |
| **housekeeping retropseudogenes** | | | | | | |
| chr2 | 109867790 | | 109868235 | | NM_003096 | |
| chr5 | 31923403 | | 31923839 | | NM_019059 | |
| chr7 | 144343984 | | 144345731 | | NM_001402 | |
| chr12 | 19661795 | | 19662225 | | NM_000995 | |
| **5S rRNA** | | | | | | |
| chr1 | 15976865 | | 15976906 | | human5SrRNA | |
| chr1 | 31583542 | | 31583592 | | human5SrRNA | |
| chr1 | 31851407 | | 31851445 | | human5SrRNA | |
| chr1 | 34578551 | | 34578639 | | human5SrRNA | |
| chr1 | 39619879 | | 39619968 | | human5SrRNA | |
| chr1 | 39673434 | | 39673475 | | human5SrRNA | |
| chr1 | 41932608 | | 41932698 | | human5SrRNA | |
| chr1 | 43662088 | | 43662177 | | human5SrRNA | |
| chr1 | 44480551 | | 44480593 | | human5SrRNA | |
| chr1 | 45136059 | | 45136137 | | human5SrRNA | |
| chr1 | 45955547 | | 45955598 | | human5SrRNA | |
| chr1 | 51524306 | | 51524347 | | human5SrRNA | |
| chr1 | 52439083 | | 52439177 | | human5SrRNA | |
| chr1 | 64356533 | | 64356571 | | human5SrRNA | |
| chr1 | 72642634 | | 72642675 | | human5SrRNA | |
| chr1 | 74215219 | | 74215309 | | human5SrRNA | |
| chr1 | 78840874 | | 78840952 | | human5SrRNA | |
| chr1 | 80851093 | | 80851133 | | human5SrRNA | |
| chr1 | 87918967 | | 87919056 | | human5SrRNA | |
| chr1 | 111584456 | | 111584545 | | human5SrRNA | |
| chr1 | 118807025 | | 118807112 | | human5SrRNA | |
| chr1 | 144935534 | | 144935575 | | human5SrRNA | |
| chr1 | 145045797 | | 145045844 | | human5SrRNA | |
| chr1 | 146124158 | | 146124246 | | human5SrRNA | |
| chr1 | 147665990 | | 147666078 | | human5SrRNA | |
| chr1 | 153874352 | | 153874393 | | human5SrRNA | |
| chr1 | 153911262 | | 153911303 | | human5SrRNA | |
| chr1 | 159148263 | | 159148352 | | human5SrRNA | |
| chr1 | 162308433 | | 162308512 | | human5SrRNA | |
| chr1 | 163438306 | | 163438395 | | human5SrRNA | |
| chr1 | 163479274 | | 163479362 | | human5SrRNA | |
| chr1 | 164838403 | | 164838442 | | human5SrRNA | |
| chr1 | 169036502 | | 169036578 | | human5SrRNA | |
| chr1 | 173890208 | | 173890285 | | human5SrRNA | |
| chr1 | 176951666 | | 176951709 | | human5SrRNA | |
| chr1 | 186341760 | | 186341849 | | human5SrRNA | |
| chr1 | 188332023 | | 188332061 | | human5SrRNA | |
| chr1 | 189635307 | | 189635385 | | human5SrRNA | |
| chr1 | 192939928 | | 192939968 | | human5SrRNA | |
| chr1 | 207714086 | | 207714135 | | human5SrRNA | |
| chr1 | 207732643 | | 207732692 | | human5SrRNA | |
| chr1 | 207749694 | | 207749743 | | human5SrRNA | |
| chr1 | 207848679 | | 207848721 | | human5SrRNA | |
| chr1 | 207882276 | | 207882325 | | human5SrRNA | |
| chr1 | 211412446 | | 211412491 | | human5SrRNA | |
| chr1 | 219836017 | | 219836058 | | human5SrRNA | |
| chr1 | 222869881 | | 222869970 | | human5SrRNA | |
| chr1 | 225449613 | | 225449652 | | human5SrRNA | |
| chr1 | 226655844 | | 226655885 | | human5SrRNA | |
| chr1 | 237980428 | | 237980466 | | human5SrRNA | |
| chr1 | 238839888 | | 238839937 | | human5SrRNA | |
| chr1 | 239864163 | | 239864201 | | human5SrRNA | |
| chr1 | 242118383 | | 242118420 | | human5SrRNA | |
| chr1 | 247603962 | | 247603999 | | human5SrRNA | |
| chr2 | 2741843 | | 2741884 | | human5SrRNA | |
| chr2 | 12185873 | | 12185914 | | human5SrRNA | |
| chr2 | 15671493 | | 15671542 | | human5SrRNA | |
| chr2 | 26794602 | | 26794643 | | human5SrRNA | |
| chr2 | 28780916 | | 28780958 | | human5SrRNA | |
| chr2 | 28906842 | | 28906922 | | human5SrRNA | |
| chr2 | 45093025 | | 45093066 | | human5SrRNA | |
| chr2 | 47414071 | | 47414109 | | human5SrRNA | |
| chr2 | 48569826 | | 48569867 | | human5SrRNA | |
| chr2 | 50196805 | | 50196894 | | human5SrRNA | |
| chr2 | 54173551 | | 54173592 | | human5SrRNA | |
| chr2 | 56462367 | | 56462465 | | human5SrRNA | |
| chr2 | 59549315 | | 59549403 | | human5SrRNA | |
| chr2 | 59921897 | | 59921986 | | human5SrRNA | |
| chr2 | 61225887 | | 61225973 | | human5SrRNA | |
| chr2 | 63997343 | | 63997384 | | human5SrRNA | |
| chr2 | 72489595 | | 72489645 | | human5SrRNA | |
| chr2 | 73854006 | | 73854061 | | human5SrRNA | |
| chr2 | 73914345 | | 73914400 | | human5SrRNA | |
| chr2 | 81723362 | | 81723451 | | human5SrRNA | |
| chr2 | 97622463 | | 97622552 | | human5SrRNA | |
| chr2 | 101169114 | | 101169152 | | human5SrRNA | |
| chr2 | 115075275 | | 115075316 | | human5SrRNA | |
| chr2 | 115585780 | | 115585822 | | human5SrRNA | |
| chr2 | 116509736 | | 116509792 | | human5SrRNA | |
| chr2 | 118649525 | | 118649562 | | human5SrRNA | |
| chr2 | 122557539 | | 122557581 | | human5SrRNA | |
| chr2 | 131584241 | | 131584291 | | human5SrRNA | |
| chr2 | 138269695 | | 138269782 | | human5SrRNA | |
| chr2 | 143438001 | | 143438042 | | human5SrRNA | |
| chr2 | 149225076 | | 149225116 | | human5SrRNA | |
| chr2 | 156396960 | | 156397000 | | human5SrRNA | |
| chr2 | 160870910 | | 160870951 | | human5SrRNA | |
| chr2 | 163353447 | | 163353535 | | human5SrRNA | |
| chr2 | 163574715 | | 163574757 | | human5SrRNA | |
| chr2 | 182913549 | | 182913639 | | human5SrRNA | |
| chr2 | 189141483 | | 189141571 | | human5SrRNA | |
| chr2 | 194384037 | | 194384085 | | human5SrRNA | |
| chr2 | 195208717 | | 195208758 | | human5SrRNA | |
| chr2 | 195594419 | | 195594460 | | human5SrRNA | |
| chr2 | 199466271 | | 199466312 | | human5SrRNA | |
| chr2 | 202793904 | | 202793943 | | human5SrRNA | |
| chr2 | 212400378 | | 212400467 | | human5SrRNA | |
| chr2 | 214122837 | | 214122884 | | human5SrRNA | |
| chr2 | 215572057 | | 215572098 | | human5SrRNA | |
| chr2 | 224080703 | | 224080734 | | human5SrRNA | |
| chr2 | 232687135 | | 232687177 | | human5SrRNA | |
| chr2 | 235098242 | | 235098283 | | human5SrRNA | |
| chr2 | 242524383 | | 242524472 | | human5SrRNA | |
| chr3 | 12196531 | | 12196571 | | human5SrRNA | |
| chr3 | 12368311 | | 12368352 | | human5SrRNA | |
| chr3 | 18189731 | | 18189770 | | human5SrRNA | |
| chr3 | 25066498 | | 25066584 | | human5SrRNA | |
| chr3 | 25393784 | | 25393883 | | human5SrRNA | |
| chr3 | 27558845 | | 27558889 | | human5SrRNA | |
| chr3 | 30072743 | | 30072787 | | human5SrRNA | |
| chr3 | 31270207 | | 31270295 | | human5SrRNA | |
| chr3 | 37383140 | | 37383229 | | human5SrRNA | |
| chr3 | 44364153 | | 44364196 | | human5SrRNA | |
| chr3 | 46499884 | | 46499939 | | human5SrRNA | |
| chr3 | 46693774 | | 46693815 | | human5SrRNA | |
| chr3 | 49448637 | | 49448677 | | human5SrRNA | |
| chr3 | 50561993 | | 50562034 | | human5SrRNA | |
| chr3 | 50795939 | | 50795979 | | human5SrRNA | |
| chr3 | 61618206 | | 61618247 | | human5SrRNA | |
| chr3 | 61684872 | | 61684911 | | human5SrRNA | |
| chr3 | 63531116 | | 63531196 | | human5SrRNA | |
| chr3 | 68932119 | | 68932196 | | human5SrRNA | |
| chr3 | 72740874 | | 72740962 | | human5SrRNA | |
| chr3 | 99619318 | | 99619359 | | human5SrRNA | |
| chr3 | 100139012 | | 100139065 | | human5SrRNA | |
| chr3 | 121460314 | | 121460355 | | human5SrRNA | |
| chr3 | 121809469 | | 121809516 | | human5SrRNA | |
| chr3 | 125058701 | | 125058742 | | human5SrRNA | |
| chr3 | 126283432 | | 126283520 | | human5SrRNA | |
| chr3 | 127682451 | | 127682540 | | human5SrRNA | |
| chr3 | 133428930 | | 133429019 | | human5SrRNA | |
| chr3 | 134502308 | | 134502397 | | human5SrRNA | |
| chr3 | 136249262 | | 136249310 | | human5SrRNA | |
| chr3 | 137237008 | | 137237097 | | human5SrRNA | |
| chr3 | 142099317 | | 142099358 | | human5SrRNA | |
| chr3 | 142310544 | | 142310633 | | human5SrRNA | |
| chr3 | 150905886 | | 150905971 | | human5SrRNA | |
| chr3 | 155824013 | | 155824051 | | human5SrRNA | |
| chr3 | 169728250 | | 169728287 | | human5SrRNA | |
| chr3 | 178146988 | | 178147077 | | human5SrRNA | |
| chr3 | 179069097 | | 179069138 | | human5SrRNA | |
| chr3 | 179879675 | | 179879765 | | human5SrRNA | |
| chr3 | 185178988 | | 185179029 | | human5SrRNA | |
| chr3 | 185841108 | | 185841148 | | human5SrRNA | |
| chr3 | 185971409 | | 185971489 | | human5SrRNA | |
| chr3 | 188637948 | | 188637989 | | human5SrRNA | |
| chr3 | 192406339 | | 192406389 | | human5SrRNA | |
| chr3 | 194194316 | | 194194368 | | human5SrRNA | |
| chr3 | 197084624 | | 197084679 | | human5SrRNA | |
| chr4 | 4346866 | | 4346907 | | human5SrRNA | |
| chr4 | 40533260 | | 40533301 | | human5SrRNA | |
| chr4 | 56115961 | | 56115996 | | human5SrRNA | |
| chr4 | 56963557 | | 56963645 | | human5SrRNA | |
| chr4 | 73089741 | | 73089779 | | human5SrRNA | |
| chr4 | 124078771 | | 124078821 | | human5SrRNA | |
| chr4 | 149479099 | | 149479189 | | human5SrRNA | |
| chr4 | 150828712 | | 150828801 | | human5SrRNA | |
| chr4 | 151694075 | | 151694116 | | human5SrRNA | |
| chr4 | 161154570 | | 161154612 | | human5SrRNA | |
| chr4 | 178378293 | | 178378382 | | human5SrRNA | |
| chr5 | 14903461 | | 14903502 | | human5SrRNA | |
| chr5 | 16033377 | | 16033464 | | human5SrRNA | |
| chr5 | 16854103 | | 16854196 | | human5SrRNA | |
| chr5 | 17157101 | | 17157182 | | human5SrRNA | |
| chr5 | 34802385 | | 34802473 | | human5SrRNA | |
| chr5 | 38074927 | | 38074969 | | human5SrRNA | |
| chr5 | 39010231 | | 39010271 | | human5SrRNA | |
| chr5 | 44638026 | | 44638076 | | human5SrRNA | |
| chr5 | 54480766 | | 54480855 | | human5SrRNA | |
| chr5 | 55556184 | | 55556271 | | human5SrRNA | |
| chr5 | 62110348 | | 62110389 | | human5SrRNA | |
| chr5 | 63691038 | | 63691127 | | human5SrRNA | |
| chr5 | 64014706 | | 64014745 | | human5SrRNA | |
| chr5 | 65472413 | | 65472455 | | human5SrRNA | |
| chr5 | 70788830 | | 70788913 | | human5SrRNA | |
| chr5 | 75486186 | | 75486226 | | human5SrRNA | |
| chr5 | 80743488 | | 80743529 | | human5SrRNA | |
| chr5 | 81058036 | | 81058078 | | human5SrRNA | |
| chr5 | 87514082 | | 87514123 | | human5SrRNA | |
| chr5 | 91196810 | | 91196851 | | human5SrRNA | |
| chr5 | 97326427 | | 97326479 | | human5SrRNA | |
| chr5 | 99629460 | | 99629502 | | human5SrRNA | |
| chr5 | 101466756 | | 101466845 | | human5SrRNA | |
| chr5 | 105258722 | | 105258815 | | human5SrRNA | |
| chr5 | 113734993 | | 113735028 | | human5SrRNA | |
| chr5 | 114480661 | | 114480699 | | human5SrRNA | |
| chr5 | 116153784 | | 116153826 | | human5SrRNA | |
| chr5 | 129450096 | | 129450185 | | human5SrRNA | |
| chr5 | 132076635 | | 132076676 | | human5SrRNA | |
| chr5 | 132184269 | | 132184358 | | human5SrRNA | |
| chr5 | 135301000 | | 135301034 | | human5SrRNA | |
| chr5 | 137469193 | | 137469234 | | human5SrRNA | |
| chr5 | 137556192 | | 137556233 | | human5SrRNA | |
| chr5 | 140111672 | | 140111759 | | human5SrRNA | |
| chr5 | 141499848 | | 141499888 | | human5SrRNA | |
| chr5 | 148430266 | | 148430304 | | human5SrRNA | |
| chr5 | 148853022 | | 148853062 | | human5SrRNA | |
| chr5 | 150306499 | | 150306549 | | human5SrRNA | |
| chr5 | 151255165 | | 151255253 | | human5SrRNA | |
| chr5 | 151517968 | | 151518010 | | human5SrRNA | |
| chr5 | 155272447 | | 155272537 | | human5SrRNA | |
| chr5 | 157460234 | | 157460281 | | human5SrRNA | |
| chr5 | 172643261 | | 172643310 | | human5SrRNA | |
| chr5 | 172719537 | | 172719626 | | human5SrRNA | |
| chr6 | 3088954 | | 3089043 | | human5SrRNA | |
| chr6 | 5830714 | | 5830755 | | human5SrRNA | |
| chr6 | 7776203 | | 7776239 | | human5SrRNA | |
| chr6 | 12390275 | | 12390323 | | human5SrRNA | |
| chr6 | 13988996 | | 13989035 | | human5SrRNA | |
| chr6 | 14404513 | | 14404554 | | human5SrRNA | |
| chr6 | 19438514 | | 19438604 | | human5SrRNA | |
| chr6 | 27740109 | | 27740150 | | human5SrRNA | |
| chr6 | 28124040 | | 28124077 | | human5SrRNA | |
| chr6 | 30510277 | | 30510318 | | human5SrRNA | |
| chr6 | 37312722 | | 37312759 | | human5SrRNA | |
| chr6 | 41892169 | | 41892210 | | human5SrRNA | |
| chr6 | 42555543 | | 42555584 | | human5SrRNA | |
| chr6 | 43625576 | | 43625663 | | human5SrRNA | |
| chr6 | 47074873 | | 47074911 | | human5SrRNA | |
| chr6 | 52797772 | | 52797812 | | human5SrRNA | |
| chr6 | 69693255 | | 69693298 | | human5SrRNA | |
| chr6 | 76942016 | | 76942054 | | human5SrRNA | |
| chr6 | 89446895 | | 89446935 | | human5SrRNA | |
| chr6 | 90961046 | | 90961087 | | human5SrRNA | |
| chr6 | 106897254 | | 106897338 | | human5SrRNA | |
| chr6 | 108573605 | | 108573692 | | human5SrRNA | |
| chr6 | 110074369 | | 110074458 | | human5SrRNA | |
| chr6 | 114541861 | | 114541949 | | human5SrRNA | |
| chr6 | 116499410 | | 116499496 | | human5SrRNA | |
| chr6 | 118349016 | | 118349055 | | human5SrRNA | |
| chr6 | 126301022 | | 126301080 | | human5SrRNA | |
| chr6 | 126386952 | | 126386993 | | human5SrRNA | |
| chr6 | 127639030 | | 127639118 | | human5SrRNA | |
| chr6 | 129814542 | | 129814634 | | human5SrRNA | |
| chr6 | 132072970 | | 132073008 | | human5SrRNA | |
| chr6 | 135596960 | | 135597001 | | human5SrRNA | |
| chr6 | 137627146 | | 137627187 | | human5SrRNA | |
| chr6 | 140243505 | | 140243544 | | human5SrRNA | |
| chr6 | 140479752 | | 140479831 | | human5SrRNA | |
| chr6 | 153056935 | | 153056972 | | human5SrRNA | |
| chr6 | 160102696 | | 160102740 | | human5SrRNA | |
| chr6 | 161207529 | | 161207613 | | human5SrRNA | |
| chr6 | 162473066 | | 162473108 | | human5SrRNA | |
| chr6 | 163906225 | | 163906265 | | human5SrRNA | |
| chr6 | 165823051 | | 165823140 | | human5SrRNA | |
| chr7 | 5170829 | | 5170870 | | human5SrRNA | |
| chr7 | 11994348 | | 11994389 | | human5SrRNA | |
| chr7 | 14615741 | | 14615782 | | human5SrRNA | |
| chr7 | 22338213 | | 22338302 | | human5SrRNA | |
| chr7 | 33217352 | | 33217443 | | human5SrRNA | |
| chr7 | 36450442 | | 36450483 | | human5SrRNA | |
| chr7 | 36633290 | | 36633375 | | human5SrRNA | |
| chr7 | 40301845 | | 40301891 | | human5SrRNA | |
| chr7 | 46120151 | | 46120190 | | human5SrRNA | |
| chr7 | 66015592 | | 66015681 | | human5SrRNA | |
| chr7 | 66681435 | | 66681479 | | human5SrRNA | |
| chr7 | 72062544 | | 72062588 | | human5SrRNA | |
| chr7 | 73901777 | | 73901870 | | human5SrRNA | |
| chr7 | 74011129 | | 74011218 | | human5SrRNA | |
| chr7 | 77339561 | | 77339600 | | human5SrRNA | |
| chr7 | 92043498 | | 92043539 | | human5SrRNA | |
| chr7 | 98863456 | | 98863497 | | human5SrRNA | |
| chr7 | 107927137 | | 107927177 | | human5SrRNA | |
| chr7 | 120723782 | | 120723871 | | human5SrRNA | |
| chr7 | 125923152 | | 125923193 | | human5SrRNA | |
| chr7 | 126159708 | | 126159749 | | human5SrRNA | |
| chr7 | 128074994 | | 128075034 | | human5SrRNA | |
| chr7 | 129396106 | | 129396193 | | human5SrRNA | |
| chr7 | 131924564 | | 131924605 | | human5SrRNA | |
| chr7 | 140070239 | | 140070327 | | human5SrRNA | |
| chr7 | 140486727 | | 140486777 | | human5SrRNA | |
| chr7 | 141107343 | | 141107384 | | human5SrRNA | |
| chr7 | 148481397 | | 148481485 | | human5SrRNA | |
| chr7 | 149811829 | | 149811867 | | human5SrRNA | |
| chr7 | 153683126 | | 153683164 | | human5SrRNA | |
| chr7 | 158026215 | | 158026258 | | human5SrRNA | |
| chr8 | 3558014 | | 3558093 | | human5SrRNA | |
| chr8 | 4038757 | | 4038797 | | human5SrRNA | |
| chr8 | 4969322 | | 4969359 | | human5SrRNA | |
| chr8 | 13393538 | | 13393617 | | human5SrRNA | |
| chr8 | 17605627 | | 17605717 | | human5SrRNA | |
| chr8 | 20147685 | | 20147774 | | human5SrRNA | |
| chr8 | 28387928 | | 28388016 | | human5SrRNA | |
| chr8 | 28906032 | | 28906120 | | human5SrRNA | |
| chr8 | 31455631 | | 31455720 | | human5SrRNA | |
| chr8 | 36621963 | | 36622036 | | human5SrRNA | |
| chr8 | 50080451 | | 50080498 | | human5SrRNA | |
| chr8 | 52458866 | | 52458953 | | human5SrRNA | |
| chr8 | 53003216 | | 53003257 | | human5SrRNA | |
| chr8 | 56657789 | | 56657876 | | human5SrRNA | |
| chr8 | 57201888 | | 57201928 | | human5SrRNA | |
| chr8 | 58039232 | | 58039318 | | human5SrRNA | |
| chr8 | 60368444 | | 60368533 | | human5SrRNA | |
| chr8 | 61156162 | | 61156203 | | human5SrRNA | |
| chr8 | 63051551 | | 63051602 | | human5SrRNA | |
| chr8 | 66510360 | | 66510401 | | human5SrRNA | |
| chr8 | 68011538 | | 68011627 | | human5SrRNA | |
| chr8 | 77819699 | | 77819752 | | human5SrRNA | |
| chr8 | 84673783 | | 84673824 | | human5SrRNA | |
| chr8 | 87128873 | | 87128918 | | human5SrRNA | |
| chr8 | 89408691 | | 89408776 | | human5SrRNA | |
| chr8 | 93179689 | | 93179778 | | human5SrRNA | |
| chr8 | 94453505 | | 94453594 | | human5SrRNA | |
| chr8 | 94709614 | | 94709688 | | human5SrRNA | |
| chr8 | 97857964 | | 97858005 | | human5SrRNA | |
| chr8 | 103533412 | | 103533462 | | human5SrRNA | |
| chr8 | 106206881 | | 106206921 | | human5SrRNA | |
| chr8 | 111990681 | | 111990720 | | human5SrRNA | |
| chr8 | 117034928 | | 117035015 | | human5SrRNA | |
| chr8 | 117444152 | | 117444193 | | human5SrRNA | |
| chr8 | 117952299 | | 117952336 | | human5SrRNA | |
| chr8 | 121386128 | | 121386169 | | human5SrRNA | |
| chr8 | 123958074 | | 123958115 | | human5SrRNA | |
| chr8 | 124232700 | | 124232750 | | human5SrRNA | |
| chr8 | 131391575 | | 131391624 | | human5SrRNA | |
| chr8 | 133766520 | | 133766561 | | human5SrRNA | |
| chr9 | 401794 | | 401835 | | human5SrRNA | |
| chr9 | 7071821 | | 7071862 | | human5SrRNA | |
| chr9 | 8740133 | | 8740176 | | human5SrRNA | |
| chr9 | 15220864 | | 15220962 | | human5SrRNA | |
| chr9 | 29042224 | | 29042265 | | human5SrRNA | |
| chr9 | 32293556 | | 32293636 | | human5SrRNA | |
| chr9 | 71835185 | | 71835226 | | human5SrRNA | |
| chr9 | 74914669 | | 74914749 | | human5SrRNA | |
| chr9 | 76045222 | | 76045262 | | human5SrRNA | |
| chr9 | 81503777 | | 81503826 | | human5SrRNA | |
| chr9 | 84500412 | | 84500490 | | human5SrRNA | |
| chr9 | 85698146 | | 85698184 | | human5SrRNA | |
| chr9 | 86618417 | | 86618455 | | human5SrRNA | |
| chr9 | 88082356 | | 88082405 | | human5SrRNA | |
| chr9 | 90606457 | | 90606497 | | human5SrRNA | |
| chr9 | 101908159 | | 101908247 | | human5SrRNA | |
| chr9 | 109963064 | | 109963142 | | human5SrRNA | |
| chr9 | 111398598 | | 111398678 | | human5SrRNA | |
| chr9 | 112778498 | | 112778539 | | human5SrRNA | |
| chr9 | 115014538 | | 115014627 | | human5SrRNA | |
| chr9 | 124252617 | | 124252658 | | human5SrRNA | |
| chr10 | 327976 | | 328065 | | human5SrRNA | |
| chr10 | 4176715 | | 4176756 | | human5SrRNA | |
| chr10 | 5942281 | | 5942322 | | human5SrRNA | |
| chr10 | 14705404 | | 14705493 | | human5SrRNA | |
| chr10 | 18907898 | | 18907948 | | human5SrRNA | |
| chr10 | 21454526 | | 21454564 | | human5SrRNA | |
| chr10 | 25178168 | | 25178208 | | human5SrRNA | |
| chr10 | 29013719 | | 29013763 | | human5SrRNA | |
| chr10 | 31828325 | | 31828373 | | human5SrRNA | |
| chr10 | 43736713 | | 43736744 | | human5SrRNA | |
| chr10 | 55654118 | | 55654162 | | human5SrRNA | |
| chr10 | 55934992 | | 55935033 | | human5SrRNA | |
| chr10 | 62277182 | | 62277222 | | human5SrRNA | |
| chr10 | 66572203 | | 66572244 | | human5SrRNA | |
| chr10 | 74695827 | | 74695865 | | human5SrRNA | |
| chr10 | 95062947 | | 95063031 | | human5SrRNA | |
| chr10 | 95270348 | | 95270437 | | human5SrRNA | |
| chr10 | 108729780 | | 108729868 | | human5SrRNA | |
| chr10 | 120050214 | | 120050254 | | human5SrRNA | |
| chr10 | 124717100 | | 124717141 | | human5SrRNA | |
| chr10 | 127311181 | | 127311235 | | human5SrRNA | |
| chr10 | 127834062 | | 127834151 | | human5SrRNA | |
| chr11 | 8739124 | | 8739166 | | human5SrRNA | |
| chr11 | 10240192 | | 10240231 | | human5SrRNA | |
| chr11 | 21318776 | | 21318815 | | human5SrRNA | |
| chr11 | 22709897 | | 22709986 | | human5SrRNA | |
| chr11 | 27543184 | | 27543272 | | human5SrRNA | |
| chr11 | 31621436 | | 31621485 | | human5SrRNA | |
| chr11 | 32185732 | | 32185772 | | human5SrRNA | |
| chr11 | 38785183 | | 38785223 | | human5SrRNA | |
| chr11 | 57217656 | | 57217745 | | human5SrRNA | |
| chr11 | 57587125 | | 57587168 | | human5SrRNA | |
| chr11 | 73909810 | | 73909899 | | human5SrRNA | |
| chr11 | 75645977 | | 75646064 | | human5SrRNA | |
| chr11 | 86478535 | | 86478575 | | human5SrRNA | |
| chr11 | 95257210 | | 95257298 | | human5SrRNA | |
| chr11 | 96207767 | | 96207855 | | human5SrRNA | |
| chr11 | 96565912 | | 96565952 | | human5SrRNA | |
| chr11 | 99219887 | | 99219928 | | human5SrRNA | |
| chr11 | 105160242 | | 105160283 | | human5SrRNA | |
| chr11 | 105265688 | | 105265729 | | human5SrRNA | |
| chr11 | 106201704 | | 106201745 | | human5SrRNA | |
| chr11 | 110022144 | | 110022185 | | human5SrRNA | |
| chr11 | 113297897 | | 113297939 | | human5SrRNA | |
| chr11 | 124506355 | | 124506444 | | human5SrRNA | |
| chr12 | 12490900 | | 12490940 | | human5SrRNA | |
| chr12 | 13199086 | | 13199175 | | human5SrRNA | |
| chr12 | 13440667 | | 13440713 | | human5SrRNA | |
| chr12 | 13593844 | | 13593935 | | human5SrRNA | |
| chr12 | 26526584 | | 26526666 | | human5SrRNA | |
| chr12 | 27960716 | | 27960754 | | human5SrRNA | |
| chr12 | 28658327 | | 28658416 | | human5SrRNA | |
| chr12 | 32664773 | | 32664822 | | human5SrRNA | |
| chr12 | 44082971 | | 44083025 | | human5SrRNA | |
| chr12 | 45417402 | | 45417451 | | human5SrRNA | |
| chr12 | 46634765 | | 46634806 | | human5SrRNA | |
| chr12 | 49071773 | | 49071835 | | human5SrRNA | |
| chr12 | 50637226 | | 50637275 | | human5SrRNA | |
| chr12 | 62787811 | | 62787851 | | human5SrRNA | |
| chr12 | 71936902 | | 71936943 | | human5SrRNA | |
| chr12 | 72228954 | | 72228995 | | human5SrRNA | |
| chr12 | 90120711 | | 90120799 | | human5SrRNA | |
| chr12 | 90682699 | | 90682740 | | human5SrRNA | |
| chr12 | 97834006 | | 97834053 | | human5SrRNA | |
| chr12 | 99027725 | | 99027766 | | human5SrRNA | |
| chr12 | 99477594 | | 99477682 | | human5SrRNA | |
| chr12 | 102173592 | | 102173682 | | human5SrRNA | |
| chr12 | 102230596 | | 102230686 | | human5SrRNA | |
| chr12 | 104519014 | | 104519103 | | human5SrRNA | |
| chr12 | 106126439 | | 106126480 | | human5SrRNA | |
| chr12 | 107880323 | | 107880412 | | human5SrRNA | |
| chr12 | 111777331 | | 111777415 | | human5SrRNA | |
| chr12 | 114642866 | | 114642907 | | human5SrRNA | |
| chr12 | 116968374 | | 116968416 | | human5SrRNA | |
| chr13 | 21920155 | | 21920196 | | human5SrRNA | |
| chr13 | 22041029 | | 22041069 | | human5SrRNA | |
| chr13 | 28109702 | | 28109743 | | human5SrRNA | |
| chr13 | 28718750 | | 28718788 | | human5SrRNA | |
| chr13 | 30773161 | | 30773199 | | human5SrRNA | |
| chr13 | 32374762 | | 32374799 | | human5SrRNA | |
| chr13 | 38429018 | | 38429107 | | human5SrRNA | |
| chr13 | 43409804 | | 43409845 | | human5SrRNA | |
| chr13 | 49339028 | | 49339116 | | human5SrRNA | |
| chr13 | 70598214 | | 70598262 | | human5SrRNA | |
| chr13 | 92957223 | | 92957262 | | human5SrRNA | |
| chr13 | 94677763 | | 94677852 | | human5SrRNA | |
| chr13 | 98015266 | | 98015355 | | human5SrRNA | |
| chr13 | 98360063 | | 98360104 | | human5SrRNA | |
| chr14 | 20883146 | | 20883235 | | human5SrRNA | |
| chr14 | 22069930 | | 22070018 | | human5SrRNA | |
| chr14 | 33550350 | | 33550392 | | human5SrRNA | |
| chr14 | 50019351 | | 50019437 | | human5SrRNA | |
| chr14 | 55483612 | | 55483653 | | human5SrRNA | |
| chr14 | 67726944 | | 67727031 | | human5SrRNA | |
| chr14 | 76070516 | | 76070605 | | human5SrRNA | |
| chr14 | 76529560 | | 76529601 | | human5SrRNA | |
| chr14 | 77870248 | | 77870336 | | human5SrRNA | |
| chr14 | 78644176 | | 78644264 | | human5SrRNA | |
| chr14 | 83523448 | | 83523486 | | human5SrRNA | |
| chr14 | 89988578 | | 89988621 | | human5SrRNA | |
| chr14 | 92270233 | | 92270316 | | human5SrRNA | |
| chr14 | 92630838 | | 92630879 | | human5SrRNA | |
| chr14 | 97294895 | | 97294936 | | human5SrRNA | |
| chr14 | 100048780 | | 100048821 | | human5SrRNA | |
| chr14 | 105590081 | | 105590122 | | human5SrRNA | |
| chr14 | 107092677 | | 107092766 | | human5SrRNA | |
| chr15 | 20466419 | | 20466459 | | human5SrRNA | |
| chr15 | 45007980 | | 45008022 | | human5SrRNA | |
| chr15 | 55578306 | | 55578394 | | human5SrRNA | |
| chr15 | 55655754 | | 55655843 | | human5SrRNA | |
| chr15 | 62977554 | | 62977594 | | human5SrRNA | |
| chr15 | 63720697 | | 63720735 | | human5SrRNA | |
| chr15 | 78691414 | | 78691455 | | human5SrRNA | |
| chr15 | 79789536 | | 79789574 | | human5SrRNA | |
| chr15 | 83259188 | | 83259229 | | human5SrRNA | |
| chr16 | 9693946 | | 9694041 | | human5SrRNA | |
| chr16 | 12007673 | | 12007711 | | human5SrRNA | |
| chr16 | 17258196 | | 17258245 | | human5SrRNA | |
| chr16 | 18103586 | | 18103627 | | human5SrRNA | |
| chr16 | 19074481 | | 19074520 | | human5SrRNA | |
| chr16 | 21049821 | | 21049862 | | human5SrRNA | |
| chr16 | 25489429 | | 25489463 | | human5SrRNA | |
| chr16 | 29327126 | | 29327170 | | human5SrRNA | |
| chr16 | 33392131 | | 33392171 | | human5SrRNA | |
| chr16 | 47484277 | | 47484372 | | human5SrRNA | |
| chr16 | 48934359 | | 48934400 | | human5SrRNA | |
| chr16 | 59765259 | | 59765347 | | human5SrRNA | |
| chr16 | 66335712 | | 66335801 | | human5SrRNA | |
| chr16 | 68776382 | | 68776476 | | human5SrRNA | |
| chr16 | 68971729 | | 68971767 | | human5SrRNA | |
| chr16 | 70829689 | | 70829727 | | human5SrRNA | |
| chr16 | 73737988 | | 73738029 | | human5SrRNA | |
| chr16 | 76778888 | | 76778923 | | human5SrRNA | |
| chr16 | 80388721 | | 80388762 | | human5SrRNA | |
| chr16 | 84073551 | | 84073641 | | human5SrRNA | |
| chr16 | 84296443 | | 84296534 | | human5SrRNA | |
| chr16 | 88055915 | | 88055959 | | human5SrRNA | |
| chr17 | 2092239 | | 2092280 | | human5SrRNA | |
| chr17 | 6504282 | | 6504371 | | human5SrRNA | |
| chr17 | 9845156 | | 9845197 | | human5SrRNA | |
| chr17 | 15685656 | | 15685729 | | human5SrRNA | |
| chr17 | 29606329 | | 29606370 | | human5SrRNA | |
| chr17 | 32447100 | | 32447187 | | human5SrRNA | |
| chr17 | 39874406 | | 39874495 | | human5SrRNA | |
| chr17 | 43404774 | | 43404863 | | human5SrRNA | |
| chr17 | 43635021 | | 43635062 | | human5SrRNA | |
| chr17 | 44036808 | | 44036849 | | human5SrRNA | |
| chr17 | 44582719 | | 44582760 | | human5SrRNA | |
| chr17 | 47693434 | | 47693475 | | human5SrRNA | |
| chr17 | 50979466 | | 50979505 | | human5SrRNA | |
| chr17 | 53056946 | | 53056987 | | human5SrRNA | |
| chr17 | 55369203 | | 55369244 | | human5SrRNA | |
| chr17 | 56804100 | | 56804142 | | human5SrRNA | |
| chr17 | 57007390 | | 57007431 | | human5SrRNA | |
| chr17 | 62900684 | | 62900725 | | human5SrRNA | |
| chr17 | 72413753 | | 72413843 | | human5SrRNA | |
| chr18 | 9923916 | | 9923999 | | human5SrRNA | |
| chr18 | 13407510 | | 13407554 | | human5SrRNA | |
| chr18 | 15053400 | | 15053436 | | human5SrRNA | |
| chr18 | 35243166 | | 35243207 | | human5SrRNA | |
| chr18 | 46259320 | | 46259362 | | human5SrRNA | |
| chr18 | 47472671 | | 47472758 | | human5SrRNA | |
| chr18 | 47845374 | | 47845466 | | human5SrRNA | |
| chr18 | 57379954 | | 57379994 | | human5SrRNA | |
| chr18 | 67339200 | | 67339241 | | human5SrRNA | |
| chr18 | 72162067 | | 72162109 | | human5SrRNA | |
| chr18 | 72245077 | | 72245117 | | human5SrRNA | |
| chr19 | 453136 | | 453224 | | human5SrRNA | |
| chr19 | 18082835 | | 18082883 | | human5SrRNA | |
| chr19 | 29492574 | | 29492663 | | human5SrRNA | |
| chr19 | 33501933 | | 33501973 | | human5SrRNA | |
| chr19 | 34507783 | | 34507824 | | human5SrRNA | |
| chr19 | 34628206 | | 34628247 | | human5SrRNA | |
| chr19 | 35143449 | | 35143490 | | human5SrRNA | |
| chr19 | 51654205 | | 51654248 | | human5SrRNA | |
| chr19 | 52571512 | | 52571601 | | human5SrRNA | |
| chr19 | 52621011 | | 52621090 | | human5SrRNA | |
| chr19 | 54128141 | | 54128184 | | human5SrRNA | |
| chr19 | 58378631 | | 58378720 | | human5SrRNA | |
| chr20 | 2392662 | | 2392699 | | human5SrRNA | |
| chr20 | 5079033 | | 5079121 | | human5SrRNA | |
| chr20 | 15933520 | | 15933569 | | human5SrRNA | |
| chr20 | 18414486 | | 18414573 | | human5SrRNA | |
| chr20 | 18696137 | | 18696179 | | human5SrRNA | |
| chr20 | 21465226 | | 21465316 | | human5SrRNA | |
| chr20 | 22758538 | | 22758627 | | human5SrRNA | |
| chr20 | 29944673 | | 29944761 | | human5SrRNA | |
| chr20 | 32430070 | | 32430117 | | human5SrRNA | |
| chr20 | 39482779 | | 39482869 | | human5SrRNA | |
| chr20 | 49421152 | | 49421193 | | human5SrRNA | |
| chr21 | 20660435 | | 20660472 | | human5SrRNA | |
| chr21 | 26574521 | | 26574610 | | human5SrRNA | |
| chr21 | 26931501 | | 26931542 | | human5SrRNA | |
| chr21 | 30580387 | | 30580428 | | human5SrRNA | |
| chr21 | 33856170 | | 33856215 | | human5SrRNA | |
| chr21 | 37012237 | | 37012277 | | human5SrRNA | |
| chr21 | 39194581 | | 39194623 | | human5SrRNA | |
| chr21 | 45309531 | | 45309572 | | human5SrRNA | |
| chr22 | 26111046 | | 26111136 | | human5SrRNA | |
| chr22 | 31701390 | | 31701479 | | human5SrRNA | |
| chr22 | 33816271 | | 33816312 | | human5SrRNA | |
| chr22 | 39644734 | | 39644831 | | human5SrRNA | |
| chr22 | 45573505 | | 45573544 | | human5SrRNA | |
| chrX | 10598087 | | 10598123 | | human5SrRNA | |
| chrX | 17648358 | | 17648399 | | human5SrRNA | |
| chrX | 17841806 | | 17841847 | | human5SrRNA | |
| chrX | 37912829 | | 37912870 | | human5SrRNA | |
| chrX | 41152051 | | 41152140 | | human5SrRNA | |
| chrX | 44564653 | | 44564703 | | human5SrRNA | |
| chrX | 48082887 | | 48082929 | | human5SrRNA | |
| chrX | 48094788 | | 48094887 | | human5SrRNA | |
| chrX | 50545676 | | 50545733 | | human5SrRNA | |
| chrX | 51186890 | | 51186928 | | human5SrRNA | |
| chrX | 53773055 | | 53773103 | | human5SrRNA | |
| chrX | 55288040 | | 55288080 | | human5SrRNA | |
| chrX | 57789061 | | 57789110 | | human5SrRNA | |
| chrX | 63360950 | | 63360991 | | human5SrRNA | |
| chrX | 70654815 | | 70654855 | | human5SrRNA | |
| chrX | 71223494 | | 71223531 | | human5SrRNA | |
| chrX | 73576007 | | 73576044 | | human5SrRNA | |
| chrX | 75875439 | | 75875528 | | human5SrRNA | |
| chrX | 77092697 | | 77092734 | | human5SrRNA | |
| chrX | 80649908 | | 80649949 | | human5SrRNA | |
| chrX | 101558219 | | 101558257 | | human5SrRNA | |
| chrX | 101638740 | | 101638778 | | human5SrRNA | |
| chrX | 103105752 | | 103105841 | | human5SrRNA | |
| chrX | 106962551 | | 106962592 | | human5SrRNA | |
| chrX | 122388042 | | 122388084 | | human5SrRNA | |
| chrX | 123197332 | | 123197374 | | human5SrRNA | |
| chrX | 128733732 | | 128733773 | | human5SrRNA | |
| chrX | 128896279 | | 128896320 | | human5SrRNA | |
| chrX | 131072895 | | 131072983 | | human5SrRNA | |
| chrX | 145509001 | | 145509090 | | human5SrRNA | |
| chrY | 14446444 | | 14446486 | | human5SrRNA | |
| **hY1** | | | | | | |
| chr1 | 240861620 | | 240861730 | | hY1 | |
| chr2 | 138472902 | | 138473006 | | hY1 | |
| chr6 | 144300375 | | 144300429 | | hY1 | |
| chr10 | 97090168 | | 97090237 | | hY1 | |
| chr16 | 75668800 | | 75668840 | | hY1 | |
| chr18 | 29843103 | | 29843199 | | hY1 | |
| chrX | 108987876 | | 108987978 | | hY1 | |
| **hY3** | | | | | | |
| chr6 | 70995247 | | 70995281 | | hY3 | |
| chr12 | 113563391 | | 113563439 | | hY3 | |
| chr13 | 43019756 | | 43019798 | | hY3 | |
| **7SK** | | | | | | |
| chr1 | 31316672 | | 31316909 | | humanRN7SK | |
| chr1 | 33802226 | | 33802465 | | humanRN7SK | |
| chr1 | 55842295 | | 55842524 | | humanRN7SK | |
| chr1 | 97161515 | | 97161732 | | humanRN7SK | |
| chr1 | 192844877 | | 192845113 | | humanRN7SK | |
| chr1 | 226633639 | | 226633872 | | humanRN7SK | |
| chr2 | 48444715 | | 48444953 | | humanRN7SK | |
| chr2 | 56178914 | | 56179149 | | humanRN7SK | |
| chr2 | 76672296 | | 76672536 | | humanRN7SK | |
| chr2 | 124626904 | | 124627147 | | humanRN7SK | |
| chr2 | 133281190 | | 133281431 | | humanRN7SK | |
| chr2 | 207086039 | | 207086276 | | humanRN7SK | |
| chr2 | 220455468 | | 220455706 | | humanRN7SK | |
| chr3 | 2130661 | | 2130895 | | humanRN7SK | |
| chr3 | 41808180 | | 41808411 | | humanRN7SK | |
| chr3 | 56106132 | | 56106369 | | humanRN7SK | |
| chr3 | 83737524 | | 83737760 | | humanRN7SK | |
| chr3 | 142392444 | | 142392684 | | humanRN7SK | |
| chr3 | 156735131 | | 156735378 | | humanRN7SK | |
| chr3 | 191810911 | | 191811149 | | humanRN7SK | |
| chr4 | 4922563 | | 4922791 | | humanRN7SK | |
| chr4 | 5460364 | | 5460608 | | humanRN7SK | |
| chr4 | 7113891 | | 7114126 | | humanRN7SK | |
| chr4 | 45997136 | | 45997375 | | humanRN7SK | |
| chr4 | 56406754 | | 56407002 | | humanRN7SK | |
| chr4 | 97269945 | | 97270188 | | humanRN7SK | |
| chr4 | 140606342 | | 140606581 | | humanRN7SK | |
| chr4 | 145956284 | | 145956526 | | humanRN7SK | |
| chr5 | 6848337 | | 6848575 | | humanRN7SK | |
| chr5 | 17345725 | | 17345964 | | humanRN7SK | |
| chr5 | 41175140 | | 41175376 | | humanRN7SK | |
| chr5 | 68266335 | | 68266577 | | humanRN7SK | |
| chr5 | 101638208 | | 101638448 | | humanRN7SK | |
| chr5 | 109035303 | | 109035535 | | humanRN7SK | |
| chr5 | 113589210 | | 113589422 | | humanRN7SK | |
| chr5 | 143914988 | | 143915233 | | humanRN7SK | |
| chr5 | 146092175 | | 146092414 | | humanRN7SK | |
| chr5 | 148495807 | | 148496042 | | humanRN7SK | |
| chr5 | 151083899 | | 151084139 | | humanRN7SK | |
| chr5 | 179272832 | | 179273068 | | humanRN7SK | |
| chr6 | 22085774 | | 22086020 | | humanRN7SK | |
| chr6 | 30832027 | | 30832270 | | humanRN7SK | |
| chr6 | 76364777 | | 76365015 | | humanRN7SK | |
| chr6 | 90710987 | | 90711225 | | humanRN7SK | |
| chr6 | 106352248 | | 106352495 | | humanRN7SK | |
| chr6 | 132141474 | | 132141712 | | humanRN7SK | |
| chr6 | 141807310 | | 141807549 | | humanRN7SK | |
| chr7 | 3038728 | | 3038973 | | humanRN7SK | |
| chr7 | 12916309 | | 12916507 | | humanRN7SK | |
| chr7 | 18847665 | | 18847900 | | humanRN7SK | |
| chr7 | 71150491 | | 71150732 | | humanRN7SK | |
| chr7 | 94430826 | | 94431070 | | humanRN7SK | |
| chr7 | 102497899 | | 102498132 | | humanRN7SK | |
| chr7 | 103124749 | | 103124985 | | humanRN7SK | |
| chr7 | 143899637 | | 143899681 | | humanRN7SK | |
| chr7 | 144045500 | | 144045544 | | humanRN7SK | |
| chr7 | 154973591 | | 154973826 | | humanRN7SK | |
| chr8 | 50242769 | | 50243008 | | humanRN7SK | |
| chr8 | 92607193 | | 92607430 | | humanRN7SK | |
| chr8 | 99933535 | | 99933776 | | humanRN7SK | |
| chr8 | 102150187 | | 102150420 | | humanRN7SK | |
| chr8 | 129232750 | | 129232996 | | humanRN7SK | |
| chr9 | 34049964 | | 34050212 | | humanRN7SK | |
| chr9 | 34270865 | | 34270920 | | humanRN7SK | |
| chr9 | 37661813 | | 37662046 | | humanRN7SK | |
| chr9 | 77414811 | | 77415057 | | humanRN7SK | |
| chr9 | 80780381 | | 80780612 | | humanRN7SK | |
| chr9 | 88873340 | | 88873561 | | humanRN7SK | |
| chr9 | 102046305 | | 102046541 | | humanRN7SK | |
| chr9 | 103343423 | | 103343661 | | humanRN7SK | |
| chr9 | 107859037 | | 107859266 | | humanRN7SK | |
| chr9 | 109442258 | | 109442501 | | humanRN7SK | |
| chr9 | 119959397 | | 119959638 | | humanRN7SK | |
| chr10 | 6191586 | | 6191832 | | humanRN7SK | |
| chr10 | 17524461 | | 17524708 | | humanRN7SK | |
| chr10 | 22222072 | | 22222310 | | humanRN7SK | |
| chr10 | 25682534 | | 25682771 | | humanRN7SK | |
| chr10 | 69917836 | | 69918075 | | humanRN7SK | |
| chr10 | 91923461 | | 91923717 | | humanRN7SK | |
| chr10 | 110700831 | | 110701068 | | humanRN7SK | |
| chr10 | 123170430 | | 123170665 | | humanRN7SK | |
| chr11 | 13374755 | | 13374997 | | humanRN7SK | |
| chr11 | 16863800 | | 16864037 | | humanRN7SK | |
| chr11 | 57219165 | | 57219400 | | humanRN7SK | |
| chr11 | 67129885 | | 67130127 | | humanRN7SK | |
| chr11 | 73543496 | | 73543735 | | humanRN7SK | |
| chr11 | 74396270 | | 74396508 | | humanRN7SK | |
| chr11 | 100710362 | | 100710594 | | humanRN7SK | |
| chr11 | 127277746 | | 127277976 | | humanRN7SK | |
| chr12 | 10236785 | | 10237021 | | humanRN7SK | |
| chr12 | 13836476 | | 13836714 | | humanRN7SK | |
| chr12 | 14706666 | | 14706904 | | humanRN7SK | |
| chr12 | 25932280 | | 25932503 | | humanRN7SK | |
| chr12 | 66686463 | | 66686705 | | humanRN7SK | |
| chr12 | 76709532 | | 76709759 | | humanRN7SK | |
| chr12 | 80737339 | | 80737571 | | humanRN7SK | |
| chr12 | 94402594 | | 94402823 | | humanRN7SK | |
| chr12 | 110100750 | | 110100988 | | humanRN7SK | |
| chr12 | 112704881 | | 112705119 | | humanRN7SK | |
| chr13 | 57459498 | | 57459734 | | humanRN7SK | |
| chr13 | 96493580 | | 96493819 | | humanRN7SK | |
| chr13 | 98856177 | | 98856446 | | humanRN7SK | |
| chr13 | 99857962 | | 99858203 | | humanRN7SK | |
| chr13 | 110567443 | | 110567680 | | humanRN7SK | |
| chr14 | 24261131 | | 24261338 | | humanRN7SK | |
| chr14 | 37063113 | | 37063352 | | humanRN7SK | |
| chr14 | 50535286 | | 50535530 | | humanRN7SK | |
| chr14 | 85655240 | | 85655474 | | humanRN7SK | |
| chr14 | 90178855 | | 90179094 | | humanRN7SK | |
| chr14 | 97104624 | | 97104851 | | humanRN7SK | |
| chr15 | 41137670 | | 41137740 | | humanRN7SK | |
| chr15 | 47012473 | | 47012709 | | humanRN7SK | |
| chr15 | 47564795 | | 47565026 | | humanRN7SK | |
| chr15 | 58996114 | | 58996353 | | humanRN7SK | |
| chr15 | 97385299 | | 97385539 | | humanRN7SK | |
| chr16 | 29742489 | | 29742725 | | humanRN7SK | |
| chr16 | 51705233 | | 51705474 | | humanRN7SK | |
| chr16 | 81995531 | | 81995772 | | humanRN7SK | |
| chr17 | 35038649 | | 35038890 | | humanRN7SK | |
| chr17 | 70660052 | | 70660291 | | humanRN7SK | |
| chr18 | 29298856 | | 29299097 | | humanRN7SK | |
| chr18 | 36596078 | | 36596308 | | humanRN7SK | |
| chr18 | 43569679 | | 43569911 | | humanRN7SK | |
| chr19 | 33353762 | | 33353998 | | humanRN7SK | |
| chr19 | 56248651 | | 56248892 | | humanRN7SK | |
| chr20 | 17556925 | | 17557154 | | humanRN7SK | |
| chr20 | 33969364 | | 33969610 | | humanRN7SK | |
| chr20 | 41691966 | | 41692201 | | humanRN7SK | |
| chr20 | 45487973 | | 45488211 | | humanRN7SK | |
| chr20 | 51714192 | | 51714436 | | humanRN7SK | |
| chr21 | 26473445 | | 26473683 | | humanRN7SK | |
| chr22 | 20455732 | | 20455969 | | humanRN7SK | |
| chr22 | 21743480 | | 21743717 | | humanRN7SK | |
| chr22 | 21899696 | | 21899933 | | humanRN7SK | |
| chr22 | 26360023 | | 26360239 | | humanRN7SK | |
| chr22 | 50918730 | | 50918940 | | humanRN7SK | |
| chrX | 13614355 | | 13614590 | | humanRN7SK | |
| chrX | 95665549 | | 95665781 | | humanRN7SK | |
| chrX | 144138628 | | 144138867 | | humanRN7SK | |
| chrY | 7192393 | | 7192636 | | humanRN7SK | |
| **U2 snRNA** | | | | | | |
| chr1 | 7856843 | | 7856877 | | humanRNU2 | |
| chr1 | 17617162 | | 17617205 | | humanRNU2 | |
| chr1 | 23831837 | | 23831872 | | humanRNU2 | |
| chr1 | 24000997 | | 24001031 | | humanRNU2 | |
| chr1 | 25487187 | | 25487225 | | humanRNU2 | |
| chr1 | 25542994 | | 25543032 | | humanRNU2 | |
| chr1 | 32402072 | | 32402104 | | humanRNU2 | |
| chr1 | 37983032 | | 37983069 | | humanRNU2 | |
| chr1 | 40995290 | | 40995322 | | humanRNU2 | |
| chr1 | 42640454 | | 42640488 | | humanRNU2 | |
| chr1 | 46753609 | | 46753647 | | humanRNU2 | |
| chr1 | 52078445 | | 52078482 | | humanRNU2 | |
| chr1 | 53391740 | | 53391775 | | humanRNU2 | |
| chr1 | 53541212 | | 53541246 | | humanRNU2 | |
| chr1 | 64982856 | | 64982892 | | humanRNU2 | |
| chr1 | 65050892 | | 65050928 | | humanRNU2 | |
| chr1 | 67442503 | | 67442536 | | humanRNU2 | |
| chr1 | 70227102 | | 70227137 | | humanRNU2 | |
| chr1 | 75917609 | | 75917642 | | humanRNU2 | |
| chr1 | 90656881 | | 90656916 | | humanRNU2 | |
| chr1 | 91878206 | | 91878259 | | humanRNU2 | |
| chr1 | 94279762 | | 94279796 | | humanRNU2 | |
| chr1 | 107771994 | | 107772035 | | humanRNU2 | |
| chr1 | 110856158 | | 110856192 | | humanRNU2 | |
| chr1 | 111672677 | | 111672711 | | humanRNU2 | |
| chr1 | 112102892 | | 112102921 | | humanRNU2 | |
| chr1 | 115201625 | | 115201659 | | humanRNU2 | |
| chr1 | 115671397 | | 115671431 | | humanRNU2 | |
| chr1 | 119860626 | | 119860663 | | humanRNU2 | |
| chr1 | 144944672 | | 144944728 | | humanRNU2 | |
| chr1 | 160082438 | | 160082480 | | humanRNU2 | |
| chr1 | 176213119 | | 176213165 | | humanRNU2 | |
| chr1 | 180535043 | | 180535077 | | humanRNU2 | |
| chr1 | 180765885 | | 180765919 | | humanRNU2 | |
| chr1 | 182916435 | | 182916471 | | humanRNU2 | |
| chr1 | 183429931 | | 183429970 | | humanRNU2 | |
| chr1 | 192840106 | | 192840148 | | humanRNU2 | |
| chr1 | 201450105 | | 201450137 | | humanRNU2 | |
| chr1 | 208847353 | | 208847387 | | humanRNU2 | |
| chr1 | 214115161 | | 214115218 | | humanRNU2 | |
| chr1 | 214427875 | | 214427913 | | humanRNU2 | |
| chr1 | 215783627 | | 215783661 | | humanRNU2 | |
| chr1 | 219776810 | | 219776852 | | humanRNU2 | |
| chr1 | 224606548 | | 224606579 | | humanRNU2 | |
| chr1 | 232970084 | | 232970119 | | humanRNU2 | |
| chr1 | 233025327 | | 233025359 | | humanRNU2 | |
| chr1 | 238443176 | | 238443211 | | humanRNU2 | |
| chr1 | 238662627 | | 238662661 | | humanRNU2 | |
| chr1 | 242543303 | | 242543345 | | humanRNU2 | |
| chr1 | 244770553 | | 244770588 | | humanRNU2 | |
| chr1 | 246061437 | | 246061474 | | humanRNU2 | |
| chr2 | 4249789 | | 4249823 | | humanRNU2 | |
| chr2 | 8932601 | | 8932646 | | humanRNU2 | |
| chr2 | 11701320 | | 11701376 | | humanRNU2 | |
| chr2 | 16700972 | | 16701007 | | humanRNU2 | |
| chr2 | 16858492 | | 16858526 | | humanRNU2 | |
| chr2 | 18353880 | | 18353912 | | humanRNU2 | |
| chr2 | 25991890 | | 25991945 | | humanRNU2 | |
| chr2 | 39073873 | | 39073908 | | humanRNU2 | |
| chr2 | 43003086 | | 43003120 | | humanRNU2 | |
| chr2 | 43796461 | | 43796496 | | humanRNU2 | |
| chr2 | 43991549 | | 43991581 | | humanRNU2 | |
| chr2 | 45635497 | | 45635531 | | humanRNU2 | |
| chr2 | 52758172 | | 52758213 | | humanRNU2 | |
| chr2 | 54053028 | | 54053062 | | humanRNU2 | |
| chr2 | 54979817 | | 54979852 | | humanRNU2 | |
| chr2 | 57517365 | | 57517405 | | humanRNU2 | |
| chr2 | 65684032 | | 65684070 | | humanRNU2 | |
| chr2 | 69047691 | | 69047723 | | humanRNU2 | |
| chr2 | 70151828 | | 70151862 | | humanRNU2 | |
| chr2 | 74171178 | | 74171212 | | humanRNU2 | |
| chr2 | 74801528 | | 74801563 | | humanRNU2 | |
| chr2 | 86379906 | | 86379944 | | humanRNU2 | |
| chr2 | 96746594 | | 96746640 | | humanRNU2 | |
| chr2 | 96960585 | | 96960623 | | humanRNU2 | |
| chr2 | 100929101 | | 100929136 | | humanRNU2 | |
| chr2 | 111976077 | | 111976111 | | humanRNU2 | |
| chr2 | 112791847 | | 112791882 | | humanRNU2 | |
| chr2 | 113268905 | | 113268942 | | humanRNU2 | |
| chr2 | 114212954 | | 114212988 | | humanRNU2 | |
| chr2 | 121608645 | | 121608679 | | humanRNU2 | |
| chr2 | 125728170 | | 125728202 | | humanRNU2 | |
| chr2 | 125935169 | | 125935207 | | humanRNU2 | |
| chr2 | 136334907 | | 136334945 | | humanRNU2 | |
| chr2 | 140863431 | | 140863464 | | humanRNU2 | |
| chr2 | 149903841 | | 149903883 | | humanRNU2 | |
| chr2 | 150745538 | | 150745570 | | humanRNU2 | |
| chr2 | 158443350 | | 158443384 | | humanRNU2 | |
| chr2 | 159783284 | | 159783317 | | humanRNU2 | |
| chr2 | 165525831 | | 165525865 | | humanRNU2 | |
| chr2 | 174197189 | | 174197221 | | humanRNU2 | |
| chr2 | 184554367 | | 184554401 | | humanRNU2 | |
| chr2 | 203820013 | | 203820044 | | humanRNU2 | |
| chr2 | 210787831 | | 210787865 | | humanRNU2 | |
| chr2 | 212574511 | | 212574543 | | humanRNU2 | |
| chr2 | 214443364 | | 214443398 | | humanRNU2 | |
| chr2 | 228158385 | | 228158421 | | humanRNU2 | |
| chr2 | 228976954 | | 228976988 | | humanRNU2 | |
| chr3 | 1794995 | | 1795030 | | humanRNU2 | |
| chr3 | 12108382 | | 12108421 | | humanRNU2 | |
| chr3 | 15046348 | | 15046378 | | humanRNU2 | |
| chr3 | 15350845 | | 15350877 | | humanRNU2 | |
| chr3 | 17697309 | | 17697343 | | humanRNU2 | |
| chr3 | 21625961 | | 21625995 | | humanRNU2 | |
| chr3 | 26131401 | | 26131437 | | humanRNU2 | |
| chr3 | 27334356 | | 27334390 | | humanRNU2 | |
| chr3 | 27884754 | | 27884790 | | humanRNU2 | |
| chr3 | 31118187 | | 31118242 | | humanRNU2 | |
| chr3 | 33071771 | | 33071811 | | humanRNU2 | |
| chr3 | 33814874 | | 33814910 | | humanRNU2 | |
| chr3 | 39434448 | | 39434482 | | humanRNU2 | |
| chr3 | 41297686 | | 41297724 | | humanRNU2 | |
| chr3 | 41619331 | | 41619365 | | humanRNU2 | |
| chr3 | 42370809 | | 42370844 | | humanRNU2 | |
| chr3 | 45338311 | | 45338353 | | humanRNU2 | |
| chr3 | 59968237 | | 59968277 | | humanRNU2 | |
| chr3 | 61244914 | | 61244952 | | humanRNU2 | |
| chr3 | 66062408 | | 66062446 | | humanRNU2 | |
| chr3 | 68907300 | | 68907336 | | humanRNU2 | |
| chr3 | 69679150 | | 69679182 | | humanRNU2 | |
| chr3 | 72848113 | | 72848163 | | humanRNU2 | |
| chr3 | 98354883 | | 98354915 | | humanRNU2 | |
| chr3 | 100772728 | | 100772762 | | humanRNU2 | |
| chr3 | 111372623 | | 111372657 | | humanRNU2 | |
| chr3 | 111558669 | | 111558704 | | humanRNU2 | |
| chr3 | 113888321 | | 113888355 | | humanRNU2 | |
| chr3 | 117356178 | | 117356213 | | humanRNU2 | |
| chr3 | 124309472 | | 124309506 | | humanRNU2 | |
| chr3 | 133522094 | | 133522129 | | humanRNU2 | |
| chr3 | 139876814 | | 139876847 | | humanRNU2 | |
| chr3 | 141747751 | | 141747784 | | humanRNU2 | |
| chr3 | 149121772 | | 149121827 | | humanRNU2 | |
| chr3 | 149959564 | | 149959620 | | humanRNU2 | |
| chr3 | 156885176 | | 156885206 | | humanRNU2 | |
| chr3 | 159586297 | | 159586331 | | humanRNU2 | |
| chr3 | 173598072 | | 173598107 | | humanRNU2 | |
| chr3 | 179049115 | | 179049149 | | humanRNU2 | |
| chr3 | 182494524 | | 182494559 | | humanRNU2 | |
| chr3 | 186512346 | | 186512386 | | humanRNU2 | |
| chr3 | 188386619 | | 188386664 | | humanRNU2 | |
| chr3 | 189129454 | | 189129487 | | humanRNU2 | |
| chr3 | 190477504 | | 190477552 | | humanRNU2 | |
| chr3 | 191873374 | | 191873408 | | humanRNU2 | |
| chr4 | 3180476 | | 3180511 | | humanRNU2 | |
| chr4 | 17560656 | | 17560691 | | humanRNU2 | |
| chr4 | 19382529 | | 19382564 | | humanRNU2 | |
| chr4 | 38310281 | | 38310318 | | humanRNU2 | |
| chr4 | 38330491 | | 38330521 | | humanRNU2 | |
| chr4 | 39967311 | | 39967346 | | humanRNU2 | |
| chr4 | 47310881 | | 47310937 | | humanRNU2 | |
| chr4 | 63240740 | | 63240774 | | humanRNU2 | |
| chr4 | 74315373 | | 74315408 | | humanRNU2 | |
| chr4 | 75541642 | | 75541676 | | humanRNU2 | |
| chr4 | 76750691 | | 76750742 | | humanRNU2 | |
| chr4 | 79629343 | | 79629377 | | humanRNU2 | |
| chr4 | 91956881 | | 91956915 | | humanRNU2 | |
| chr4 | 93257580 | | 93257615 | | humanRNU2 | |
| chr4 | 95939157 | | 95939187 | | humanRNU2 | |
| chr4 | 96171895 | | 96171929 | | humanRNU2 | |
| chr4 | 99276661 | | 99276696 | | humanRNU2 | |
| chr4 | 99866898 | | 99866934 | | humanRNU2 | |
| chr4 | 101236319 | | 101236361 | | humanRNU2 | |
| chr4 | 102995940 | | 102995978 | | humanRNU2 | |
| chr4 | 103308106 | | 103308138 | | humanRNU2 | |
| chr4 | 103967511 | | 103967566 | | humanRNU2 | |
| chr4 | 106722343 | | 106722374 | | humanRNU2 | |
| chr4 | 109218449 | | 109218481 | | humanRNU2 | |
| chr4 | 110389076 | | 110389109 | | humanRNU2 | |
| chr4 | 113910239 | | 113910274 | | humanRNU2 | |
| chr4 | 116557791 | | 116557825 | | humanRNU2 | |
| chr4 | 119725868 | | 119725898 | | humanRNU2 | |
| chr4 | 120615004 | | 120615042 | | humanRNU2 | |
| chr4 | 139501159 | | 139501189 | | humanRNU2 | |
| chr4 | 148376249 | | 148376288 | | humanRNU2 | |
| chr4 | 149731565 | | 149731600 | | humanRNU2 | |
| chr4 | 151220623 | | 151220665 | | humanRNU2 | |
| chr4 | 151393710 | | 151393745 | | humanRNU2 | |
| chr4 | 159936847 | | 159936881 | | humanRNU2 | |
| chr4 | 166214380 | | 166214461 | | humanRNU2 | |
| chr4 | 169066382 | | 169066416 | | humanRNU2 | |
| chr4 | 169248230 | | 169248262 | | humanRNU2 | |
| chr4 | 169303517 | | 169303553 | | humanRNU2 | |
| chr4 | 171003006 | | 171003040 | | humanRNU2 | |
| chr4 | 174875537 | | 174875571 | | humanRNU2 | |
| chr4 | 182835065 | | 182835098 | | humanRNU2 | |
| chr4 | 183469812 | | 183469870 | | humanRNU2 | |
| chr4 | 186963764 | | 186963798 | | humanRNU2 | |
| chr5 | 23243507 | | 23243542 | | humanRNU2 | |
| chr5 | 24255488 | | 24255521 | | humanRNU2 | |
| chr5 | 28689364 | | 28689397 | | humanRNU2 | |
| chr5 | 32033138 | | 32033175 | | humanRNU2 | |
| chr5 | 50293586 | | 50293621 | | humanRNU2 | |
| chr5 | 53110503 | | 53110537 | | humanRNU2 | |
| chr5 | 54539751 | | 54539781 | | humanRNU2 | |
| chr5 | 57738369 | | 57738403 | | humanRNU2 | |
| chr5 | 64885219 | | 64885287 | | humanRNU2 | |
| chr5 | 65925673 | | 65925708 | | humanRNU2 | |
| chr5 | 72345880 | | 72345914 | | humanRNU2 | |
| chr5 | 78667781 | | 78667813 | | humanRNU2 | |
| chr5 | 81124268 | | 81124301 | | humanRNU2 | |
| chr5 | 84746810 | | 84746843 | | humanRNU2 | |
| chr5 | 86502883 | | 86502927 | | humanRNU2 | |
| chr5 | 89761906 | | 89761940 | | humanRNU2 | |
| chr5 | 91188669 | | 91188702 | | humanRNU2 | |
| chr5 | 106542119 | | 106542153 | | humanRNU2 | |
| chr5 | 107175621 | | 107175655 | | humanRNU2 | |
| chr5 | 109166141 | | 109166176 | | humanRNU2 | |
| chr5 | 111058181 | | 111058217 | | humanRNU2 | |
| chr5 | 112267775 | | 112267815 | | humanRNU2 | |
| chr5 | 120755906 | | 120755940 | | humanRNU2 | |
| chr5 | 128770398 | | 128770432 | | humanRNU2 | |
| chr5 | 134189969 | | 134190000 | | humanRNU2 | |
| chr5 | 135989367 | | 135989401 | | humanRNU2 | |
| chr5 | 136574522 | | 136574561 | | humanRNU2 | |
| chr5 | 136668415 | | 136668454 | | humanRNU2 | |
| chr5 | 137402964 | | 137402999 | | humanRNU2 | |
| chr5 | 142029512 | | 142029548 | | humanRNU2 | |
| chr5 | 145813324 | | 145813353 | | humanRNU2 | |
| chr5 | 149359370 | | 149359401 | | humanRNU2 | |
| chr5 | 150661750 | | 150661782 | | humanRNU2 | |
| chr5 | 150817029 | | 150817066 | | humanRNU2 | |
| chr5 | 154857003 | | 154857038 | | humanRNU2 | |
| chr5 | 167893837 | | 167893870 | | humanRNU2 | |
| chr5 | 177562690 | | 177562727 | | humanRNU2 | |
| chr6 | 10673908 | | 10673945 | | humanRNU2 | |
| chr6 | 12737032 | | 12737067 | | humanRNU2 | |
| chr6 | 14092891 | | 14092924 | | humanRNU2 | |
| chr6 | 15005764 | | 15005798 | | humanRNU2 | |
| chr6 | 17501505 | | 17501540 | | humanRNU2 | |
| chr6 | 20484646 | | 20484680 | | humanRNU2 | |
| chr6 | 20622923 | | 20622957 | | humanRNU2 | |
| chr6 | 26065699 | | 26065736 | | humanRNU2 | |
| chr6 | 29422679 | | 29422713 | | humanRNU2 | |
| chr6 | 33439514 | | 33439548 | | humanRNU2 | |
| chr6 | 36461586 | | 36461644 | | humanRNU2 | |
| chr6 | 38592340 | | 38592377 | | humanRNU2 | |
| chr6 | 39049032 | | 39049067 | | humanRNU2 | |
| chr6 | 42725612 | | 42725642 | | humanRNU2 | |
| chr6 | 44461413 | | 44461445 | | humanRNU2 | |
| chr6 | 45117609 | | 45117641 | | humanRNU2 | |
| chr6 | 49922922 | | 49922958 | | humanRNU2 | |
| chr6 | 52598329 | | 52598364 | | humanRNU2 | |
| chr6 | 56508573 | | 56508606 | | humanRNU2 | |
| chr6 | 66020996 | | 66021038 | | humanRNU2 | |
| chr6 | 69494195 | | 69494228 | | humanRNU2 | |
| chr6 | 82660003 | | 82660038 | | humanRNU2 | |
| chr6 | 86523200 | | 86523253 | | humanRNU2 | |
| chr6 | 92907020 | | 92907053 | | humanRNU2 | |
| chr6 | 94041879 | | 94041916 | | humanRNU2 | |
| chr6 | 101959533 | | 101959565 | | humanRNU2 | |
| chr6 | 110068297 | | 110068332 | | humanRNU2 | |
| chr6 | 110953403 | | 110953437 | | humanRNU2 | |
| chr6 | 113750026 | | 113750061 | | humanRNU2 | |
| chr6 | 115653480 | | 115653514 | | humanRNU2 | |
| chr6 | 117033545 | | 117033586 | | humanRNU2 | |
| chr6 | 124444079 | | 124444116 | | humanRNU2 | |
| chr6 | 126241117 | | 126241152 | | humanRNU2 | |
| chr6 | 134976033 | | 134976066 | | humanRNU2 | |
| chr6 | 135598055 | | 135598092 | | humanRNU2 | |
| chr6 | 138473464 | | 138473499 | | humanRNU2 | |
| chr6 | 139784134 | | 139784164 | | humanRNU2 | |
| chr6 | 153590378 | | 153590419 | | humanRNU2 | |
| chr6 | 162104398 | | 162104433 | | humanRNU2 | |
| chr6 | 163879217 | | 163879256 | | humanRNU2 | |
| chr6 | 168820071 | | 168820104 | | humanRNU2 | |
| chr6 | 169456020 | | 169456055 | | humanRNU2 | |
| chr7 | 3769468 | | 3769502 | | humanRNU2 | |
| chr7 | 6247714 | | 6247749 | | humanRNU2 | |
| chr7 | 16508027 | | 16508065 | | humanRNU2 | |
| chr7 | 21580427 | | 21580458 | | humanRNU2 | |
| chr7 | 23629047 | | 23629080 | | humanRNU2 | |
| chr7 | 26208675 | | 26208713 | | humanRNU2 | |
| chr7 | 35816400 | | 35816433 | | humanRNU2 | |
| chr7 | 37072599 | | 37072634 | | humanRNU2 | |
| chr7 | 44335012 | | 44335044 | | humanRNU2 | |
| chr7 | 44406795 | | 44406829 | | humanRNU2 | |
| chr7 | 64325169 | | 64325201 | | humanRNU2 | |
| chr7 | 66641418 | | 66641454 | | humanRNU2 | |
| chr7 | 67567174 | | 67567208 | | humanRNU2 | |
| chr7 | 72100878 | | 72100919 | | humanRNU2 | |
| chr7 | 74044574 | | 74044608 | | humanRNU2 | |
| chr7 | 78218950 | | 78218985 | | humanRNU2 | |
| chr7 | 80787191 | | 80787226 | | humanRNU2 | |
| chr7 | 84721275 | | 84721315 | | humanRNU2 | |
| chr7 | 92630497 | | 92630532 | | humanRNU2 | |
| chr7 | 99470530 | | 99470562 | | humanRNU2 | |
| chr7 | 114354315 | | 114354350 | | humanRNU2 | |
| chr7 | 115293351 | | 115293389 | | humanRNU2 | |
| chr7 | 116543373 | | 116543409 | | humanRNU2 | |
| chr7 | 135043118 | | 135043153 | | humanRNU2 | |
| chr7 | 136973392 | | 136973427 | | humanRNU2 | |
| chr7 | 143613693 | | 143613725 | | humanRNU2 | |
| chr7 | 144356990 | | 144357025 | | humanRNU2 | |
| chr7 | 145556748 | | 145556783 | | humanRNU2 | |
| chr7 | 152627845 | | 152627879 | | humanRNU2 | |
| chr8 | 8480310 | | 8480342 | | humanRNU2 | |
| chr8 | 10962279 | | 10962312 | | humanRNU2 | |
| chr8 | 19323816 | | 19323853 | | humanRNU2 | |
| chr8 | 24171710 | | 24171744 | | humanRNU2 | |
| chr8 | 24541412 | | 24541446 | | humanRNU2 | |
| chr8 | 31024125 | | 31024159 | | humanRNU2 | |
| chr8 | 32873684 | | 32873719 | | humanRNU2 | |
| chr8 | 49098317 | | 49098349 | | humanRNU2 | |
| chr8 | 51646818 | | 51646860 | | humanRNU2 | |
| chr8 | 53656953 | | 53656986 | | humanRNU2 | |
| chr8 | 55698429 | | 55698462 | | humanRNU2 | |
| chr8 | 56993255 | | 56993295 | | humanRNU2 | |
| chr8 | 58212912 | | 58212943 | | humanRNU2 | |
| chr8 | 62730532 | | 62730567 | | humanRNU2 | |
| chr8 | 70795781 | | 70795816 | | humanRNU2 | |
| chr8 | 71492355 | | 71492390 | | humanRNU2 | |
| chr8 | 73744101 | | 73744139 | | humanRNU2 | |
| chr8 | 74415892 | | 74415926 | | humanRNU2 | |
| chr8 | 75836534 | | 75836574 | | humanRNU2 | |
| chr8 | 85422476 | | 85422510 | | humanRNU2 | |
| chr8 | 90593963 | | 90593996 | | humanRNU2 | |
| chr8 | 93105780 | | 93105814 | | humanRNU2 | |
| chr8 | 94812494 | | 94812537 | | humanRNU2 | |
| chr8 | 96856433 | | 96856467 | | humanRNU2 | |
| chr8 | 97701998 | | 97702033 | | humanRNU2 | |
| chr8 | 98958508 | | 98958555 | | humanRNU2 | |
| chr8 | 99217967 | | 99217999 | | humanRNU2 | |
| chr8 | 110886191 | | 110886247 | | humanRNU2 | |
| chr8 | 120327014 | | 120327048 | | humanRNU2 | |
| chr8 | 141692551 | | 141692586 | | humanRNU2 | |
| chr9 | 161799 | | 161833 | | humanRNU2 | |
| chr9 | 1036783 | | 1036817 | | humanRNU2 | |
| chr9 | 4400167 | | 4400202 | | humanRNU2 | |
| chr9 | 6876239 | | 6876273 | | humanRNU2 | |
| chr9 | 8553658 | | 8553707 | | humanRNU2 | |
| chr9 | 11366441 | | 11366485 | | humanRNU2 | |
| chr9 | 15260738 | | 15260776 | | humanRNU2 | |
| chr9 | 19013161 | | 19013195 | | humanRNU2 | |
| chr9 | 26182997 | | 26183031 | | humanRNU2 | |
| chr9 | 26676010 | | 26676043 | | humanRNU2 | |
| chr9 | 29922834 | | 29922865 | | humanRNU2 | |
| chr9 | 42570632 | | 42570669 | | humanRNU2 | |
| chr9 | 42674127 | | 42674161 | | humanRNU2 | |
| chr9 | 42931241 | | 42931278 | | humanRNU2 | |
| chr9 | 66266800 | | 66266837 | | humanRNU2 | |
| chr9 | 67104237 | | 67104274 | | humanRNU2 | |
| chr9 | 67216092 | | 67216129 | | humanRNU2 | |
| chr9 | 69245308 | | 69245342 | | humanRNU2 | |
| chr9 | 69578097 | | 69578134 | | humanRNU2 | |
| chr9 | 70472804 | | 70472838 | | humanRNU2 | |
| chr9 | 70874084 | | 70874118 | | humanRNU2 | |
| chr9 | 72470260 | | 72470294 | | humanRNU2 | |
| chr9 | 75254086 | | 75254120 | | humanRNU2 | |
| chr9 | 79994611 | | 79994644 | | humanRNU2 | |
| chr9 | 86162746 | | 86162780 | | humanRNU2 | |
| chr9 | 87080203 | | 87080238 | | humanRNU2 | |
| chr9 | 87226603 | | 87226632 | | humanRNU2 | |
| chr9 | 89315077 | | 89315114 | | humanRNU2 | |
| chr9 | 99491337 | | 99491372 | | humanRNU2 | |
| chr9 | 106694509 | | 106694548 | | humanRNU2 | |
| chr9 | 114130408 | | 114130442 | | humanRNU2 | |
| chr9 | 115884921 | | 115884965 | | humanRNU2 | |
| chr9 | 116571898 | | 116571930 | | humanRNU2 | |
| chr9 | 118496671 | | 118496727 | | humanRNU2 | |
| chr9 | 119856832 | | 119856867 | | humanRNU2 | |
| chr9 | 127867711 | | 127867770 | | humanRNU2 | |
| chr9 | 128420635 | | 128420672 | | humanRNU2 | |
| chr10 | 10521931 | | 10521966 | | humanRNU2 | |
| chr10 | 14947214 | | 14947253 | | humanRNU2 | |
| chr10 | 16518002 | | 16518060 | | humanRNU2 | |
| chr10 | 17535955 | | 17535989 | | humanRNU2 | |
| chr10 | 18888170 | | 18888200 | | humanRNU2 | |
| chr10 | 23011615 | | 23011654 | | humanRNU2 | |
| chr10 | 24061784 | | 24061821 | | humanRNU2 | |
| chr10 | 25546444 | | 25546479 | | humanRNU2 | |
| chr10 | 27521084 | | 27521118 | | humanRNU2 | |
| chr10 | 28961080 | | 28961109 | | humanRNU2 | |
| chr10 | 30847349 | | 30847383 | | humanRNU2 | |
| chr10 | 33092096 | | 33092128 | | humanRNU2 | |
| chr10 | 33763557 | | 33763597 | | humanRNU2 | |
| chr10 | 34394698 | | 34394733 | | humanRNU2 | |
| chr10 | 44430353 | | 44430388 | | humanRNU2 | |
| chr10 | 44915677 | | 44915711 | | humanRNU2 | |
| chr10 | 45197175 | | 45197216 | | humanRNU2 | |
| chr10 | 46612998 | | 46613032 | | humanRNU2 | |
| chr10 | 46862108 | | 46862142 | | humanRNU2 | |
| chr10 | 47342917 | | 47342951 | | humanRNU2 | |
| chr10 | 48799926 | | 48799960 | | humanRNU2 | |
| chr10 | 49051866 | | 49051900 | | humanRNU2 | |
| chr10 | 49320443 | | 49320477 | | humanRNU2 | |
| chr10 | 52345472 | | 52345509 | | humanRNU2 | |
| chr10 | 62672629 | | 62672685 | | humanRNU2 | |
| chr10 | 68732846 | | 68732878 | | humanRNU2 | |
| chr10 | 69980223 | | 69980276 | | humanRNU2 | |
| chr10 | 70649053 | | 70649087 | | humanRNU2 | |
| chr10 | 71323212 | | 71323247 | | humanRNU2 | |
| chr10 | 76352605 | | 76352639 | | humanRNU2 | |
| chr10 | 78616114 | | 78616149 | | humanRNU2 | |
| chr10 | 86633051 | | 86633083 | | humanRNU2 | |
| chr10 | 87334499 | | 87334546 | | humanRNU2 | |
| chr10 | 88874066 | | 88874101 | | humanRNU2 | |
| chr10 | 93697804 | | 93697839 | | humanRNU2 | |
| chr10 | 95181859 | | 95181894 | | humanRNU2 | |
| chr10 | 96334785 | | 96334819 | | humanRNU2 | |
| chr10 | 97670490 | | 97670524 | | humanRNU2 | |
| chr10 | 99497761 | | 99497796 | | humanRNU2 | |
| chr10 | 99627863 | | 99627898 | | humanRNU2 | |
| chr10 | 99798568 | | 99798602 | | humanRNU2 | |
| chr10 | 101730307 | | 101730345 | | humanRNU2 | |
| chr10 | 104744769 | | 104744804 | | humanRNU2 | |
| chr10 | 108767623 | | 108767666 | | humanRNU2 | |
| chr10 | 115119791 | | 115119825 | | humanRNU2 | |
| chr10 | 116826499 | | 116826531 | | humanRNU2 | |
| chr10 | 119963577 | | 119963612 | | humanRNU2 | |
| chr10 | 120263851 | | 120263883 | | humanRNU2 | |
| chr10 | 133158960 | | 133158991 | | humanRNU2 | |
| chr11 | 7500474 | | 7500508 | | humanRNU2 | |
| chr11 | 10403723 | | 10403756 | | humanRNU2 | |
| chr11 | 12348971 | | 12349005 | | humanRNU2 | |
| chr11 | 17006887 | | 17006921 | | humanRNU2 | |
| chr11 | 18074689 | | 18074723 | | humanRNU2 | |
| chr11 | 21175203 | | 21175236 | | humanRNU2 | |
| chr11 | 22415892 | | 22415924 | | humanRNU2 | |
| chr11 | 34621145 | | 34621180 | | humanRNU2 | |
| chr11 | 39275486 | | 39275521 | | humanRNU2 | |
| chr11 | 43890562 | | 43890599 | | humanRNU2 | |
| chr11 | 46235331 | | 46235372 | | humanRNU2 | |
| chr11 | 49139696 | | 49139729 | | humanRNU2 | |
| chr11 | 49244063 | | 49244119 | | humanRNU2 | |
| chr11 | 57410114 | | 57410144 | | humanRNU2 | |
| chr11 | 58143193 | | 58143228 | | humanRNU2 | |
| chr11 | 60361858 | | 60361900 | | humanRNU2 | |
| chr11 | 77694820 | | 77694855 | | humanRNU2 | |
| chr11 | 89357220 | | 89357276 | | humanRNU2 | |
| chr11 | 89449879 | | 89449912 | | humanRNU2 | |
| chr11 | 94729569 | | 94729612 | | humanRNU2 | |
| chr11 | 96757917 | | 96757952 | | humanRNU2 | |
| chr11 | 100483969 | | 100484004 | | humanRNU2 | |
| chr11 | 101928768 | | 101928804 | | humanRNU2 | |
| chr11 | 101965396 | | 101965433 | | humanRNU2 | |
| chr11 | 102718116 | | 102718151 | | humanRNU2 | |
| chr11 | 102902522 | | 102902555 | | humanRNU2 | |
| chr11 | 114582851 | | 114582887 | | humanRNU2 | |
| chr11 | 118677611 | | 118677649 | | humanRNU2 | |
| chr11 | 123293368 | | 123293403 | | humanRNU2 | |
| chr11 | 126054702 | | 126054736 | | humanRNU2 | |
| chr11 | 134482050 | | 134482094 | | humanRNU2 | |
| chr11 | 134644965 | | 134644998 | | humanRNU2 | |
| chr12 | 1456423 | | 1456455 | | humanRNU2 | |
| chr12 | 8006662 | | 8006694 | | humanRNU2 | |
| chr12 | 8111155 | | 8111188 | | humanRNU2 | |
| chr12 | 8138304 | | 8138336 | | humanRNU2 | |
| chr12 | 9308600 | | 9308635 | | humanRNU2 | |
| chr12 | 10969926 | | 10969960 | | humanRNU2 | |
| chr12 | 12756810 | | 12756843 | | humanRNU2 | |
| chr12 | 19032373 | | 19032426 | | humanRNU2 | |
| chr12 | 21763810 | | 21763845 | | humanRNU2 | |
| chr12 | 22149732 | | 22149763 | | humanRNU2 | |
| chr12 | 32861251 | | 32861286 | | humanRNU2 | |
| chr12 | 41947047 | | 41947086 | | humanRNU2 | |
| chr12 | 44279447 | | 44279481 | | humanRNU2 | |
| chr12 | 49641242 | | 49641276 | | humanRNU2 | |
| chr12 | 57815268 | | 57815305 | | humanRNU2 | |
| chr12 | 67817780 | | 67817823 | | humanRNU2 | |
| chr12 | 81777242 | | 81777276 | | humanRNU2 | |
| chr12 | 85287749 | | 85287805 | | humanRNU2 | |
| chr12 | 88789440 | | 88789491 | | humanRNU2 | |
| chr12 | 89690267 | | 89690301 | | humanRNU2 | |
| chr12 | 89698973 | | 89699007 | | humanRNU2 | |
| chr12 | 93152646 | | 93152677 | | humanRNU2 | |
| chr12 | 93919890 | | 93919921 | | humanRNU2 | |
| chr12 | 95519026 | | 95519063 | | humanRNU2 | |
| chr12 | 96236649 | | 96236683 | | humanRNU2 | |
| chr12 | 100988927 | | 100988961 | | humanRNU2 | |
| chr12 | 102539648 | | 102539682 | | humanRNU2 | |
| chr12 | 106591496 | | 106591533 | | humanRNU2 | |
| chr12 | 114075442 | | 114075477 | | humanRNU2 | |
| chr12 | 114631329 | | 114631364 | | humanRNU2 | |
| chr12 | 123906725 | | 123906761 | | humanRNU2 | |
| chr12 | 124773195 | | 124773231 | | humanRNU2 | |
| chr13 | 27248433 | | 27248467 | | humanRNU2 | |
| chr13 | 29681946 | | 29681993 | | humanRNU2 | |
| chr13 | 33278623 | | 33278657 | | humanRNU2 | |
| chr13 | 34412336 | | 34412369 | | humanRNU2 | |
| chr13 | 45882387 | | 45882421 | | humanRNU2 | |
| chr13 | 48060757 | | 48060789 | | humanRNU2 | |
| chr13 | 49916990 | | 49917022 | | humanRNU2 | |
| chr13 | 51396091 | | 51396124 | | humanRNU2 | |
| chr13 | 55156542 | | 55156584 | | humanRNU2 | |
| chr13 | 56995693 | | 56995724 | | humanRNU2 | |
| chr13 | 59391275 | | 59391309 | | humanRNU2 | |
| chr13 | 59909147 | | 59909179 | | humanRNU2 | |
| chr13 | 61103845 | | 61103879 | | humanRNU2 | |
| chr13 | 62231558 | | 62231598 | | humanRNU2 | |
| chr13 | 62341476 | | 62341511 | | humanRNU2 | |
| chr13 | 69247529 | | 69247562 | | humanRNU2 | |
| chr13 | 73583516 | | 73583551 | | humanRNU2 | |
| chr13 | 80476551 | | 80476588 | | humanRNU2 | |
| chr13 | 100995961 | | 100995993 | | humanRNU2 | |
| chr13 | 101845668 | | 101845700 | | humanRNU2 | |
| chr13 | 103810428 | | 103810458 | | humanRNU2 | |
| chr13 | 111136043 | | 111136078 | | humanRNU2 | |
| chr13 | 112246642 | | 112246681 | | humanRNU2 | |
| chr14 | 22541128 | | 22541162 | | humanRNU2 | |
| chr14 | 23094160 | | 23094194 | | humanRNU2 | |
| chr14 | 28279433 | | 28279470 | | humanRNU2 | |
| chr14 | 30505489 | | 30505524 | | humanRNU2 | |
| chr14 | 40991535 | | 40991569 | | humanRNU2 | |
| chr14 | 45876988 | | 45877027 | | humanRNU2 | |
| chr14 | 54592756 | | 54592793 | | humanRNU2 | |
| chr14 | 55252245 | | 55252280 | | humanRNU2 | |
| chr14 | 64157915 | | 64157949 | | humanRNU2 | |
| chr14 | 66126279 | | 66126313 | | humanRNU2 | |
| chr14 | 66369712 | | 66369746 | | humanRNU2 | |
| chr14 | 71691202 | | 71691238 | | humanRNU2 | |
| chr14 | 74122737 | | 74122773 | | humanRNU2 | |
| chr14 | 75188420 | | 75188454 | | humanRNU2 | |
| chr14 | 75737440 | | 75737483 | | humanRNU2 | |
| chr14 | 76255625 | | 76255659 | | humanRNU2 | |
| chr14 | 84519479 | | 84519513 | | humanRNU2 | |
| chr14 | 88795486 | | 88795519 | | humanRNU2 | |
| chr14 | 94670505 | | 94670538 | | humanRNU2 | |
| chr14 | 103754975 | | 103755014 | | humanRNU2 | |
| chr15 | 20264553 | | 20264593 | | humanRNU2 | |
| chr15 | 21271317 | | 21271357 | | humanRNU2 | |
| chr15 | 23918507 | | 23918542 | | humanRNU2 | |
| chr15 | 26387894 | | 26387928 | | humanRNU2 | |
| chr15 | 26785087 | | 26785118 | | humanRNU2 | |
| chr15 | 28122854 | | 28122888 | | humanRNU2 | |
| chr15 | 35119025 | | 35119059 | | humanRNU2 | |
| chr15 | 36538370 | | 36538405 | | humanRNU2 | |
| chr15 | 38178984 | | 38179018 | | humanRNU2 | |
| chr15 | 44937349 | | 44937384 | | humanRNU2 | |
| chr15 | 46544450 | | 46544487 | | humanRNU2 | |
| chr15 | 52976824 | | 52976857 | | humanRNU2 | |
| chr15 | 53944174 | | 53944233 | | humanRNU2 | |
| chr15 | 62488759 | | 62488798 | | humanRNU2 | |
| chr15 | 63506750 | | 63506782 | | humanRNU2 | |
| chr15 | 64749568 | | 64749603 | | humanRNU2 | |
| chr15 | 65058003 | | 65058037 | | humanRNU2 | |
| chr15 | 67554005 | | 67554052 | | humanRNU2 | |
| chr15 | 68559101 | | 68559136 | | humanRNU2 | |
| chr15 | 73643301 | | 73643337 | | humanRNU2 | |
| chr15 | 75240288 | | 75240322 | | humanRNU2 | |
| chr15 | 75791088 | | 75791122 | | humanRNU2 | |
| chr15 | 75828623 | | 75828654 | | humanRNU2 | |
| chr15 | 76965152 | | 76965187 | | humanRNU2 | |
| chr15 | 79858898 | | 79858949 | | humanRNU2 | |
| chr15 | 83763556 | | 83763602 | | humanRNU2 | |
| chr15 | 85238983 | | 85239016 | | humanRNU2 | |
| chr15 | 85545839 | | 85545874 | | humanRNU2 | |
| chr15 | 93024515 | | 93024548 | | humanRNU2 | |
| chr16 | 1729306 | | 1729341 | | humanRNU2 | |
| chr16 | 16218051 | | 16218082 | | humanRNU2 | |
| chr16 | 21074329 | | 21074369 | | humanRNU2 | |
| chr16 | 23236839 | | 23236873 | | humanRNU2 | |
| chr16 | 23666305 | | 23666340 | | humanRNU2 | |
| chr16 | 55061615 | | 55061656 | | humanRNU2 | |
| chr16 | 56031473 | | 56031505 | | humanRNU2 | |
| chr16 | 56230954 | | 56230986 | | humanRNU2 | |
| chr16 | 57201727 | | 57201766 | | humanRNU2 | |
| chr16 | 60677020 | | 60677053 | | humanRNU2 | |
| chr16 | 62361763 | | 62361795 | | humanRNU2 | |
| chr16 | 67528713 | | 67528748 | | humanRNU2 | |
| chr16 | 70378233 | | 70378268 | | humanRNU2 | |
| chr16 | 70533232 | | 70533269 | | humanRNU2 | |
| chr17 | 2467957 | | 2467993 | | humanRNU2 | |
| chr17 | 3228991 | | 3229025 | | humanRNU2 | |
| chr17 | 12114495 | | 12114531 | | humanRNU2 | |
| chr17 | 19591171 | | 19591207 | | humanRNU2 | |
| chr17 | 21686128 | | 21686164 | | humanRNU2 | |
| chr17 | 25911224 | | 25911259 | | humanRNU2 | |
| chr17 | 27870639 | | 27870674 | | humanRNU2 | |
| chr17 | 28614408 | | 28614441 | | humanRNU2 | |
| chr17 | 30852588 | | 30852625 | | humanRNU2 | |
| chr17 | 31159060 | | 31159094 | | humanRNU2 | |
| chr17 | 34926888 | | 34926929 | | humanRNU2 | |
| chr17 | 35254753 | | 35254785 | | humanRNU2 | |
| chr17 | 37011097 | | 37011135 | | humanRNU2 | |
| chr17 | 44029381 | | 44029415 | | humanRNU2 | |
| chr17 | 45913332 | | 45913364 | | humanRNU2 | |
| chr17 | 49172801 | | 49172832 | | humanRNU2 | |
| chr17 | 50099397 | | 50099426 | | humanRNU2 | |
| chr17 | 51686863 | | 51686896 | | humanRNU2 | |
| chr17 | 54323598 | | 54323637 | | humanRNU2 | |
| chr17 | 56702062 | | 56702094 | | humanRNU2 | |
| chr17 | 57469467 | | 57469502 | | humanRNU2 | |
| chr17 | 61441915 | | 61441949 | | humanRNU2 | |
| chr18 | 7326659 | | 7326693 | | humanRNU2 | |
| chr18 | 12577683 | | 12577717 | | humanRNU2 | |
| chr18 | 21464093 | | 21464127 | | humanRNU2 | |
| chr18 | 23657546 | | 23657580 | | humanRNU2 | |
| chr18 | 24278053 | | 24278088 | | humanRNU2 | |
| chr18 | 28322501 | | 28322541 | | humanRNU2 | |
| chr18 | 29100681 | | 29100718 | | humanRNU2 | |
| chr18 | 30058132 | | 30058164 | | humanRNU2 | |
| chr18 | 43814108 | | 43814142 | | humanRNU2 | |
| chr18 | 44731051 | | 44731086 | | humanRNU2 | |
| chr18 | 53083686 | | 53083718 | | humanRNU2 | |
| chr18 | 56593472 | | 56593528 | | humanRNU2 | |
| chr18 | 57739383 | | 57739417 | | humanRNU2 | |
| chr18 | 62364176 | | 62364208 | | humanRNU2 | |
| chr18 | 64446256 | | 64446290 | | humanRNU2 | |
| chr18 | 66189058 | | 66189090 | | humanRNU2 | |
| chr18 | 69084595 | | 69084629 | | humanRNU2 | |
| chr18 | 69560085 | | 69560119 | | humanRNU2 | |
| chr18 | 73404765 | | 73404801 | | humanRNU2 | |
| chr19 | 2886563 | | 2886597 | | humanRNU2 | |
| chr19 | 13454661 | | 13454694 | | humanRNU2 | |
| chr19 | 16956721 | | 16956764 | | humanRNU2 | |
| chr19 | 33581270 | | 33581304 | | humanRNU2 | |
| chr19 | 40302068 | | 40302101 | | humanRNU2 | |
| chr19 | 40922787 | | 40922825 | | humanRNU2 | |
| chr19 | 49137508 | | 49137543 | | humanRNU2 | |
| chr19 | 55804382 | | 55804416 | | humanRNU2 | |
| chr20 | 2620385 | | 2620417 | | humanRNU2 | |
| chr20 | 13685263 | | 13685298 | | humanRNU2 | |
| chr20 | 15874303 | | 15874337 | | humanRNU2 | |
| chr20 | 30348634 | | 30348668 | | humanRNU2 | |
| chr20 | 36090349 | | 36090384 | | humanRNU2 | |
| chr20 | 37572666 | | 37572700 | | humanRNU2 | |
| chr20 | 45490775 | | 45490810 | | humanRNU2 | |
| chr20 | 57639836 | | 57639869 | | humanRNU2 | |
| chr21 | 15419865 | | 15419906 | | humanRNU2 | |
| chr21 | 16508003 | | 16508039 | | humanRNU2 | |
| chr21 | 18946329 | | 18946362 | | humanRNU2 | |
| chr21 | 19139111 | | 19139158 | | humanRNU2 | |
| chr21 | 20195359 | | 20195394 | | humanRNU2 | |
| chr21 | 21425662 | | 21425694 | | humanRNU2 | |
| chr21 | 21908132 | | 21908164 | | humanRNU2 | |
| chr21 | 28284983 | | 28285023 | | humanRNU2 | |
| chr21 | 35106564 | | 35106595 | | humanRNU2 | |
| chr21 | 35310552 | | 35310585 | | humanRNU2 | |
| chr21 | 40625857 | | 40625892 | | humanRNU2 | |
| chr22 | 26069350 | | 26069383 | | humanRNU2 | |
| chr22 | 27424408 | | 27424441 | | humanRNU2 | |
| chr22 | 28496163 | | 28496200 | | humanRNU2 | |
| chr22 | 29328787 | | 29328822 | | humanRNU2 | |
| chr22 | 34726677 | | 34726711 | | humanRNU2 | |
| chr22 | 34979653 | | 34979688 | | humanRNU2 | |
| chr22 | 41138752 | | 41138794 | | humanRNU2 | |
| chr22 | 42281931 | | 42281966 | | humanRNU2 | |
| chr22 | 45667700 | | 45667732 | | humanRNU2 | |
| chr22 | 48431680 | | 48431715 | | humanRNU2 | |
| chrX | 21923062 | | 21923094 | | humanRNU2 | |
| chrX | 22800343 | | 22800378 | | humanRNU2 | |
| chrX | 22806494 | | 22806532 | | humanRNU2 | |
| chrX | 23460829 | | 23460864 | | humanRNU2 | |
| chrX | 23710434 | | 23710468 | | humanRNU2 | |
| chrX | 24431520 | | 24431554 | | humanRNU2 | |
| chrX | 24685237 | | 24685271 | | humanRNU2 | |
| chrX | 27484652 | | 27484686 | | humanRNU2 | |
| chrX | 29769567 | | 29769623 | | humanRNU2 | |
| chrX | 35737808 | | 35737843 | | humanRNU2 | |
| chrX | 38309128 | | 38309164 | | humanRNU2 | |
| chrX | 40881496 | | 40881530 | | humanRNU2 | |
| chrX | 43558176 | | 43558207 | | humanRNU2 | |
| chrX | 53780168 | | 53780199 | | humanRNU2 | |
| chrX | 54053348 | | 54053383 | | humanRNU2 | |
| chrX | 54915954 | | 54915993 | | humanRNU2 | |
| chrX | 56650340 | | 56650373 | | humanRNU2 | |
| chrX | 56720979 | | 56721013 | | humanRNU2 | |
| chrX | 71507419 | | 71507453 | | humanRNU2 | |
| chrX | 93270552 | | 93270587 | | humanRNU2 | |
| chrX | 94558379 | | 94558413 | | humanRNU2 | |
| chrX | 94689373 | | 94689407 | | humanRNU2 | |
| chrX | 95685574 | | 95685609 | | humanRNU2 | |
| chrX | 99179757 | | 99179790 | | humanRNU2 | |
| chrX | 99220427 | | 99220464 | | humanRNU2 | |
| chrX | 100323911 | | 100323963 | | humanRNU2 | |
| chrX | 104536986 | | 104537019 | | humanRNU2 | |
| chrX | 104764860 | | 104764894 | | humanRNU2 | |
| chrX | 109492157 | | 109492192 | | humanRNU2 | |
| chrX | 125135000 | | 125135033 | | humanRNU2 | |
| chrX | 137739864 | | 137739899 | | humanRNU2 | |
| chrX | 152229917 | | 152229962 | | humanRNU2 | |
| chrX | 152327380 | | 152327425 | | humanRNU2 | |
| chrX | 153788817 | | 153788850 | | humanRNU2 | |
| chrX | 153872077 | | 153872110 | | humanRNU2 | |
| chrX | 155115087 | | 155115130 | | humanRNU2 | |
| chrY | 59218093 | | 59218136 | | humanRNU2 | |
| **U11** | | | | | | |
| chr11 | 75612026 | | 75612097 | | RNU11human | |
| **U1** | | | | | | |
| chr2 | 185290437 | | 185290472 | | U1 | |
| chr3 | 48521763 | | 48521802 | | U1 | |
| chr3 | 69415612 | | 69415650 | | U1 | |
| chr7 | 155416809 | | 155416844 | | U1 | |
| chr7 | 87245910 | | 87245944 | | U1 | |
| chr10 | 93766184 | | 93766223 | | U1 | |
| chr16 | 65818009 | | 65818044 | | U1 | |
| chr16 | 81294749 | | 81294784 | | U1 | |
| chr17 | 70222806 | | 70222846 | | U1 | |
| chr18 | 40975575 | | 40975610 | | U1 | |
| chrX | 7345128 | | 7345163 | | U1 | |
| chrX | 41580091 | | 41580126 | | U1 | |
| chrX | 127256763 | | 127256798 | | U1 | |
| **U3** | | | | | | |
| chr1 | 116892438 | | 116892474 | | U3 | |
| chr2 | 11609242 | | 11609274 | | U3 | |
| chr2 | 68570557 | | 68570598 | | U3 | |
| chr3 | 6150001 | | 6150038 | | U3 | |
| chr3 | 90475385 | | 90475432 | | U3 | |
| chr3 | 178961837 | | 178961871 | | U3 | |
| chr4 | 39962786 | | 39962825 | | U3 | |
| chr4 | 74424951 | | 74424991 | | U3 | |
| chr5 | 22944286 | | 22944317 | | U3 | |
| chr5 | 122941976 | | 122942008 | | U3 | |
| chr5 | 142355138 | | 142355175 | | U3 | |
| chr6 | 53012640 | | 53012727 | | U3 | |
| chr6 | 69375061 | | 69375137 | | U3 | |
| chr6 | 121764763 | | 121764805 | | U3 | |
| chr6 | 133157175 | | 133157216 | | U3 | |
| chr8 | 15400410 | | 15400447 | | U3 | |
| chr8 | 135983025 | | 135983078 | | U3 | |
| chr9 | 125707174 | | 125707210 | | U3 | |
| chr10 | 36707368 | | 36707409 | | U3 | |
| chr10 | 37893299 | | 37893340 | | U3 | |
| chr10 | 88810003 | | 88810075 | | U3 | |
| chr12 | 30052836 | | 30052873 | | U3 | |
| chr12 | 93585059 | | 93585103 | | U3 | |
| chr12 | 124128752 | | 124128825 | | U3 | |
| chr14 | 31775726 | | 31775799 | | U3 | |
| chr14 | 35388550 | | 35388588 | | U3 | |
| chr14 | 85738276 | | 85738349 | | U3 | |
| chr14 | 106803757 | | 106803802 | | U3 | |
| chr14 | 107279296 | | 107279341 | | U3 | |
| chr17 | 66254229 | | 66254267 | | U3 | |
| chr18 | 74792959 | | 74793008 | | U3 | |
| chr20 | 15029241 | | 15029305 | | U3 | |
| chr20 | 39998290 | | 39998354 | | U3 | |
| chr20 | 55182734 | | 55182802 | | U3 | |
| chr22 | 17062944 | | 17062981 | | U3 | |
| chr22 | 28189339 | | 28189382 | | U3 | |
| chrX | 155191073 | | 155191110 | | U3 | |
| chrY | 59294079 | | 59294116 | | U3 | |
| **U4** | | | | | | |
| chr1 | 48913608 | | 48913678 | | U4 | |
| chr1 | 66560158 | | 66560229 | | U4 | |
| chr1 | 93166425 | | 93166491 | | U4 | |
| chr1 | 223547164 | | 223547229 | | U4 | |
| chr1 | 233584463 | | 233584527 | | U4 | |
| chr2 | 128229810 | | 128229877 | | U4 | |
| chr2 | 239711520 | | 239711600 | | U4 | |
| chr3 | 20038295 | | 20038414 | | U4 | |
| chr3 | 172514281 | | 172514348 | | U4 | |
| chr3 | 175327267 | | 175327336 | | U4 | |
| chr4 | 120288717 | | 120288788 | | U4 | |
| chr4 | 138414394 | | 138414467 | | U4 | |
| chr4 | 166173732 | | 166173803 | | U4 | |
| chr4 | 186947673 | | 186947740 | | U4 | |
| chr5 | 115395701 | | 115395739 | | U4 | |
| chr5 | 178919088 | | 178919158 | | U4 | |
| chr6 | 148207845 | | 148207911 | | U4 | |
| chr7 | 140752049 | | 140752115 | | U4 | |
| chr8 | 53355255 | | 53355327 | | U4 | |
| chr8 | 82414536 | | 82414602 | | U4 | |
| chr8 | 113160989 | | 113161054 | | U4 | |
| chr8 | 124467280 | | 124467347 | | U4 | |
| chr8 | 129022789 | | 129022857 | | U4 | |
| chr9 | 20418319 | | 20418385 | | U4 | |
| chr9 | 36267780 | | 36267846 | | U4 | |
| chr9 | 85890856 | | 85890922 | | U4 | |
| chr9 | 94093606 | | 94093670 | | U4 | |
| chr10 | 17944817 | | 17944882 | | U4 | |
| chr10 | 18191731 | | 18191796 | | U4 | |
| chr10 | 23510509 | | 23510574 | | U4 | |
| chr10 | 111629616 | | 111629682 | | U4 | |
| chr11 | 66382493 | | 66382561 | | U4 | |
| chr11 | 101399066 | | 101399157 | | U4 | |
| chr11 | 105695360 | | 105695428 | | U4 | |
| chr11 | 129984157 | | 129984230 | | U4 | |
| chr12 | 28472331 | | 28472396 | | U4 | |
| chr14 | 62489499 | | 62489555 | | U4 | |
| chr16 | 68363366 | | 68363432 | | U4 | |
| chr17 | 2186792 | | 2186850 | | U4 | |
| chr18 | 33844752 | | 33844830 | | U4 | |
| chr22 | 38275746 | | 38275810 | | U4 | |
| chrX | 16893323 | | 16893390 | | U4 | |
| **U5** | | | | | | |
| chr1 | 35409146 | | 35409194 | | U5 | |
| chr1 | 66216584 | | 66216636 | | U5 | |
| chr1 | 86375303 | | 86375346 | | U5 | |
| chr1 | 99495633 | | 99495678 | | U5 | |
| chr1 | 161745921 | | 161745969 | | U5 | |
| chr1 | 165097497 | | 165097543 | | U5 | |
| chr1 | 175512956 | | 175513004 | | U5 | |
| chr1 | 211366115 | | 211366158 | | U5 | |
| chr1 | 221704683 | | 221704722 | | U5 | |
| chr1 | 225866682 | | 225866722 | | U5 | |
| chr1 | 236026688 | | 236026750 | | U5 | |
| chr1 | 237150755 | | 237150791 | | U5 | |
| chr2 | 11834850 | | 11834903 | | U5 | |
| chr2 | 50291780 | | 50291838 | | U5 | |
| chr2 | 84883882 | | 84883927 | | U5 | |
| chr2 | 183275905 | | 183275951 | | U5 | |
| chr2 | 222234346 | | 222234390 | | U5 | |
| chr2 | 228096966 | | 228097014 | | U5 | |
| chr2 | 228525098 | | 228525141 | | U5 | |
| chr3 | 45076010 | | 45076081 | | U5 | |
| chr3 | 81320943 | | 81320990 | | U5 | |
| chr3 | 94257307 | | 94257351 | | U5 | |
| chr3 | 101886173 | | 101886227 | | U5 | |
| chr3 | 122624897 | | 122624945 | | U5 | |
| chr3 | 152072670 | | 152072718 | | U5 | |
| chr3 | 176601683 | | 176601734 | | U5 | |
| chr3 | 188187711 | | 188187759 | | U5 | |
| chr4 | 55895029 | | 55895081 | | U5 | |
| chr4 | 65244514 | | 65244557 | | U5 | |
| chr4 | 69155712 | | 69155762 | | U5 | |
| chr4 | 92519397 | | 92519438 | | U5 | |
| chr4 | 107452372 | | 107452418 | | U5 | |
| chr4 | 113161176 | | 113161219 | | U5 | |
| chr4 | 128776380 | | 128776428 | | U5 | |
| chr4 | 137693432 | | 137693470 | | U5 | |
| chr4 | 186001048 | | 186001090 | | U5 | |
| chr5 | 77501127 | | 77501174 | | U5 | |
| chr5 | 80501461 | | 80501508 | | U5 | |
| chr5 | 113572216 | | 113572265 | | U5 | |
| chr5 | 115187933 | | 115187981 | | U5 | |
| chr5 | 116404602 | | 116404648 | | U5 | |
| chr5 | 117323607 | | 117323650 | | U5 | |
| chr6 | 5923135 | | 5923173 | | U5 | |
| chr6 | 29070525 | | 29070568 | | U5 | |
| chr6 | 29143215 | | 29143255 | | U5 | |
| chr6 | 49305012 | | 49305061 | | U5 | |
| chr6 | 86610644 | | 86610698 | | U5 | |
| chr6 | 131178059 | | 131178106 | | U5 | |
| chr7 | 16095131 | | 16095174 | | U5 | |
| chr7 | 68579496 | | 68579542 | | U5 | |
| chr7 | 69617924 | | 69617971 | | U5 | |
| chr7 | 87797933 | | 87797964 | | U5 | |
| chr7 | 89854164 | | 89854205 | | U5 | |
| chr7 | 96952238 | | 96952281 | | U5 | |
| chr8 | 13720746 | | 13720790 | | U5 | |
| chr8 | 81658496 | | 81658548 | | U5 | |
| chr8 | 87875615 | | 87875665 | | U5 | |
| chr8 | 91462471 | | 91462522 | | U5 | |
| chr8 | 109309333 | | 109309365 | | U5 | |
| chr8 | 110937758 | | 110937800 | | U5 | |
| chr8 | 125881225 | | 125881276 | | U5 | |
| chr8 | 131597090 | | 131597140 | | U5 | |
| chr8 | 144539510 | | 144539558 | | U5 | |
| chr9 | 1906157 | | 1906195 | | U5 | |
| chr9 | 12843242 | | 12843282 | | U5 | |
| chr9 | 17108133 | | 17108177 | | U5 | |
| chr9 | 22302995 | | 22303052 | | U5 | |
| chr9 | 79384093 | | 79384136 | | U5 | |
| chr9 | 92899910 | | 92899960 | | U5 | |
| chr9 | 112735171 | | 112735218 | | U5 | |
| chr9 | 121902991 | | 121903034 | | U5 | |
| chr9 | 135249262 | | 135249305 | | U5 | |
| chr9 | 135297015 | | 135297077 | | U5 | |
| chr10 | 72130847 | | 72130897 | | U5 | |
| chr11 | 11963529 | | 11963570 | | U5 | |
| chr11 | 78237441 | | 78237489 | | U5 | |
| chr11 | 86890580 | | 86890627 | | U5 | |
| chr12 | 22700489 | | 22700538 | | U5 | |
| chr12 | 52117886 | | 52117930 | | U5 | |
| chr12 | 78172582 | | 78172629 | | U5 | |
| chr12 | 101000777 | | 101000820 | | U5 | |
| chr12 | 106728926 | | 106728977 | | U5 | |
| chr12 | 128308765 | | 128308798 | | U5 | |
| chr12 | 131499436 | | 131499486 | | U5 | |
| chr13 | 55465414 | | 55465462 | | U5 | |
| chr14 | 20803144 | | 20803190 | | U5 | |
| chr14 | 50763480 | | 50763527 | | U5 | |
| chr14 | 68884790 | | 68884833 | | U5 | |
| chr14 | 87789724 | | 87789766 | | U5 | |
| chr15 | 35284046 | | 35284094 | | U5 | |
| chr15 | 93741152 | | 93741191 | | U5 | |
| chr16 | 29175225 | | 29175273 | | U5 | |
| chr16 | 48094702 | | 48094745 | | U5 | |
| chr16 | 69555874 | | 69555918 | | U5 | |
| chr16 | 74803428 | | 74803475 | | U5 | |
| chr16 | 88159257 | | 88159305 | | U5 | |
| chr17 | 35630900 | | 35630941 | | U5 | |
| chr17 | 42612547 | | 42612596 | | U5 | |
| chr17 | 46850742 | | 46850789 | | U5 | |
| chr17 | 64078176 | | 64078224 | | U5 | |
| chr18 | 29761199 | | 29761242 | | U5 | |
| chr18 | 31350363 | | 31350407 | | U5 | |
| chr18 | 33576390 | | 33576438 | | U5 | |
| chr19 | 15037943 | | 15037993 | | U5 | |
| chr20 | 1430625 | | 1430673 | | U5 | |
| chr20 | 38691522 | | 38691568 | | U5 | |
| chr20 | 48016413 | | 48016464 | | U5 | |
| chr22 | 40739119 | | 40739168 | | U5 | |
| chrX | 5232256 | | 5232299 | | U5 | |
| chrX | 26320527 | | 26320578 | | U5 | |
| chrX | 63168484 | | 63168528 | | U5 | |
| chrX | 88281804 | | 88281848 | | U5 | |
| chrX | 105949939 | | 105949982 | | U5 | |
| chrX | 106247101 | | 106247152 | | U5 | |
| chrX | 115487797 | | 115487843 | | U5 | |
| **U6** | | | | | | |
| chr1 | 18109965 | | 18109997 | | U6 | |
| chr1 | 26757814 | | 26757844 | | U6 | |
| chr1 | 42521374 | | 42521417 | | U6 | |
| chr1 | 54993081 | | 54993123 | | U6 | |
| chr1 | 84713262 | | 84713297 | | U6 | |
| chr1 | 102325407 | | 102325479 | | U6 | |
| chr1 | 164949299 | | 164949374 | | U6 | |
| chr1 | 205707710 | | 205707745 | | U6 | |
| chr1 | 247770426 | | 247770501 | | U6 | |
| chr2 | 42021662 | | 42021705 | | U6 | |
| chr2 | 43635446 | | 43635516 | | U6 | |
| chr2 | 73525812 | | 73525874 | | U6 | |
| chr2 | 74893434 | | 74893469 | | U6 | |
| chr2 | 148634991 | | 148635083 | | U6 | |
| chr2 | 230802521 | | 230802598 | | U6 | |
| chr2 | 232376601 | | 232376635 | | U6 | |
| chr2 | 233647375 | | 233647444 | | U6 | |
| chr2 | 238471808 | | 238471875 | | U6 | |
| chr3 | 17438373 | | 17438412 | | U6 | |
| chr3 | 32953297 | | 32953332 | | U6 | |
| chr3 | 108572964 | | 108572993 | | U6 | |
| chr3 | 121306397 | | 121306443 | | U6 | |
| chr3 | 122261631 | | 122261671 | | U6 | |
| chr3 | 130851776 | | 130851812 | | U6 | |
| chr3 | 136440268 | | 136440337 | | U6 | |
| chr3 | 160814714 | | 160814745 | | U6 | |
| chr3 | 169430765 | | 169430809 | | U6 | |
| chr3 | 170512921 | | 170512960 | | U6 | |
| chr3 | 192734057 | | 192734087 | | U6 | |
| chr4 | 46116236 | | 46116267 | | U6 | |
| chr4 | 53582262 | | 53582304 | | U6 | |
| chr4 | 74010744 | | 74010775 | | U6 | |
| chr4 | 106404301 | | 106404339 | | U6 | |
| chr4 | 109455763 | | 109455792 | | U6 | |
| chr4 | 178593579 | | 178593613 | | U6 | |
| chr5 | 3729751 | | 3729786 | | U6 | |
| chr5 | 31597613 | | 31597642 | | U6 | |
| chr5 | 40662262 | | 40662299 | | U6 | |
| chr5 | 83492701 | | 83492773 | | U6 | |
| chr5 | 89417789 | | 89417828 | | U6 | |
| chr5 | 92863994 | | 92864038 | | U6 | |
| chr5 | 116397603 | | 116397632 | | U6 | |
| chr5 | 126555979 | | 126556009 | | U6 | |
| chr5 | 126964407 | | 126964436 | | U6 | |
| chr5 | 139364060 | | 139364102 | | U6 | |
| chr5 | 148562204 | | 148562235 | | U6 | |
| chr5 | 172789148 | | 172789243 | | U6 | |
| chr5 | 178164173 | | 178164202 | | U6 | |
| chr6 | 12392559 | | 12392589 | | U6 | |
| chr6 | 20993739 | | 20993799 | | U6 | |
| chr6 | 56615104 | | 56615150 | | U6 | |
| chr6 | 82641155 | | 82641190 | | U6 | |
| chr6 | 105214697 | | 105214766 | | U6 | |
| chr6 | 115188071 | | 115188140 | | U6 | |
| chr7 | 25880601 | | 25880630 | | U6 | |
| chr7 | 28984101 | | 28984154 | | U6 | |
| chr7 | 44639918 | | 44639962 | | U6 | |
| chr7 | 68235078 | | 68235108 | | U6 | |
| chr7 | 73672883 | | 73672946 | | U6 | |
| chr7 | 83733502 | | 83733531 | | U6 | |
| chr8 | 24923103 | | 24923132 | | U6 | |
| chr8 | 37526416 | | 37526503 | | U6 | |
| chr8 | 67767749 | | 67767779 | | U6 | |
| chr8 | 67860783 | | 67860819 | | U6 | |
| chr8 | 68137423 | | 68137454 | | U6 | |
| chr8 | 88476535 | | 88476571 | | U6 | |
| chr8 | 124451377 | | 124451408 | | U6 | |
| chr8 | 126460137 | | 126460166 | | U6 | |
| chr9 | 2812485 | | 2812516 | | U6 | |
| chr9 | 3592332 | | 3592361 | | U6 | |
| chr9 | 81161397 | | 81161435 | | U6 | |
| chr9 | 88001542 | | 88001571 | | U6 | |
| chr9 | 88590243 | | 88590278 | | U6 | |
| chr9 | 96137838 | | 96137913 | | U6 | |
| chr9 | 98329059 | | 98329088 | | U6 | |
| chr9 | 127452494 | | 127452524 | | U6 | |
| chr10 | 744625 | | 744661 | | U6 | |
| chr10 | 13574400 | | 13574438 | | U6 | |
| chr10 | 24932456 | | 24932491 | | U6 | |
| chr10 | 32683509 | | 32683593 | | U6 | |
| chr10 | 42964889 | | 42964922 | | U6 | |
| chr10 | 56212207 | | 56212272 | | U6 | |
| chr10 | 104908408 | | 104908437 | | U6 | |
| chr10 | 118297695 | | 118297769 | | U6 | |
| chr11 | 72688642 | | 72688673 | | U6 | |
| chr11 | 86110033 | | 86110080 | | U6 | |
| chr11 | 104012071 | | 104012117 | | U6 | |
| chr11 | 109760260 | | 109760298 | | U6 | |
| chr11 | 111780687 | | 111780718 | | U6 | |
| chr12 | 17130123 | | 17130155 | | U6 | |
| chr12 | 45948373 | | 45948442 | | U6 | |
| chr12 | 53095849 | | 53095885 | | U6 | |
| chr12 | 62727895 | | 62727978 | | U6 | |
| chr12 | 105455907 | | 105455936 | | U6 | |
| chr12 | 116661808 | | 116661845 | | U6 | |
| chr12 | 119767633 | | 119767668 | | U6 | |
| chr13 | 44303990 | | 44304036 | | U6 | |
| chr13 | 71033598 | | 71033677 | | U6 | |
| chr13 | 88457611 | | 88457640 | | U6 | |
| chr14 | 29451541 | | 29451616 | | U6 | |
| chr14 | 31122528 | | 31122557 | | U6 | |
| chr14 | 32131268 | | 32131349 | | U6 | |
| chr14 | 64225419 | | 64225454 | | U6 | |
| chr14 | 77854611 | | 77854640 | | U6 | |
| chr15 | 31513177 | | 31513208 | | U6 | |
| chr15 | 44524506 | | 44524544 | | U6 | |
| chr15 | 51817567 | | 51817612 | | U6 | |
| chr15 | 68910970 | | 68910999 | | U6 | |
| chr15 | 76129026 | | 76129056 | | U6 | |
| chr16 | 3070070 | | 3070107 | | U6 | |
| chr16 | 15370919 | | 15370954 | | U6 | |
| chr16 | 16572827 | | 16572862 | | U6 | |
| chr16 | 18326847 | | 18326882 | | U6 | |
| chr16 | 22154920 | | 22154949 | | U6 | |
| chr16 | 69312854 | | 69312883 | | U6 | |
| chr16 | 75377852 | | 75377887 | | U6 | |
| chr16 | 79287400 | | 79287432 | | U6 | |
| chr17 | 14518080 | | 14518109 | | U6 | |
| chr17 | 26794683 | | 26794733 | | U6 | |
| chr17 | 29427486 | | 29427526 | | U6 | |
| chr18 | 48523983 | | 48524014 | | U6 | |
| chr19 | 33223683 | | 33223713 | | U6 | |
| chr19 | 33580432 | | 33580462 | | U6 | |
| chr19 | 58709795 | | 58709849 | | U6 | |
| chr20 | 14937750 | | 14937779 | | U6 | |
| chr22 | 22055723 | | 22055752 | | U6 | |
| chr22 | 39139874 | | 39139912 | | U6 | |
| chrX | 1948797 | | 1948836 | | U6 | |
| chrX | 34145377 | | 34145407 | | U6 | |
| chrX | 34477606 | | 34477644 | | U6 | |
| chrX | 34965161 | | 34965199 | | U6 | |
| chrX | 36837676 | | 36837710 | | U6 | |
| chrX | 37031876 | | 37031912 | | U6 | |
| chrX | 37397958 | | 37397996 | | U6 | |
| chrX | 48013423 | | 48013502 | | U6 | |
| chrX | 51613813 | | 51613905 | | U6 | |
| chrX | 77683199 | | 77683234 | | U6 | |
| chrX | 93258832 | | 93258921 | | U6 | |
| chrX | 97169502 | | 97169531 | | U6 | |
| chrX | 110664764 | | 110664835 | | U6 | |
| chrX | 128956657 | | 128956686 | | U6 | |
| chrX | 131714156 | | 131714251 | | U6 | |
| chrX | 154774668 | | 154774708 | | U6 | |
| chrY | 1898797 | | 1898836 | | U6 | |
| **U6atac** | | | | | | |
| chr3 | 52594587 | | 52594666 | | U6atac | |
| chr3 | 110270718 | | 110270812 | | U6atac | |
| chr7 | 144148774 | | 144148862 | | U6atac | |
| chr16 | 70886601 | | 70886681 | | U6atac | |
| **U13** | | | | | | |
| chr6 | 163647941 | | 163648034 | | U13 | |
| chr9 | 117327559 | | 117327633 | | U13 | |
| **further snoRNAs** | | | | | | |
| chr1 | 49210088 | | 49210143 | | SNORD121B | |
| chr4 | 151594488 | | 151594521 | | HBII-99B | |
| chr18 | 64153998 | | 64154034 | | ACA3-2 | |
| **tRNAs** | | | | | | |
| chr7 | 4678816 | | 4678853 | | chr6.trna166-AlaAGC | |
| chr9 | 112409526 | | 112409560 | | chr6.trna113-AlaTGC | |
| chr11 | 73169387 | | 73169426 | | chr6.trna70-AlaCGC | |
| chr16 | 8705746 | | 8705802 | | chr11.trna6-AlaTGC | |
| chr1 | 65818079 | | 65818141 | | chr6.trna52-ArgTCT | |
| chr1 | 73232482 | | 73232533 | | chr3.trna11-ArgACG | |
| chr1 | 175396176 | | 175396226 | | chr3.trna11-ArgACG | |
| chr1 | 241774077 | | 241774114 | | chr9.trna2-ArgTCG | |
| chr2 | 149511839 | | 149511884 | | chr14.trna7-ArgACG | |
| chr2 | 155256108 | | 155256159 | | chr14.trna7-ArgACG | |
| chr2 | 177166752 | | 177166788 | | chr11.trna3-ArgTCT | |
| chr2 | 198099548 | | 198099585 | | chr17.trna21-ArgCCT | |
| chr2 | 201528145 | | 201528182 | | chr11.trna3-ArgTCT | |
| chr3 | 87752527 | | 87752562 | | chr7.trna3-ArgCCT | |
| chr3 | 123701893 | | 123701938 | | chr14.trna7-ArgACG | |
| chr3 | 168433790 | | 168433828 | | chr1.trna9-ArgTCT | |
| chr4 | 107282924 | | 107282959 | | chr7.trna3-ArgCCT | |
| chr4 | 140079043 | | 140079096 | | chr3.trna11-ArgACG | |
| chr5 | 15346895 | | 15346946 | | chr3.trna11-ArgACG | |
| chr5 | 25503091 | | 25503142 | | chr1.trna9-ArgTCT | |
| chr5 | 65732333 | | 65732370 | | chr16.trna2-ArgCCT | |
| chr5 | 88914789 | | 88914826 | | chr17.trna18-ArgCCT | |
| chr5 | 103012706 | | 103012763 | | chr9.trna4-ArgTCT | |
| chr5 | 104705836 | | 104705881 | | chr3.trna11-ArgACG | |
| chr6 | 18053617 | | 18053668 | | chr3.trna11-ArgACG | |
| chr6 | 116785249 | | 116785314 | | chr9.trna4-ArgTCT | |
| chr6 | 131954665 | | 131954715 | | chr6.trna52-ArgTCT | |
| chr7 | 10254375 | | 10254419 | | chr14.trna7-ArgACG | |
| chr7 | 22325729 | | 22325770 | | chr3.trna11-ArgACG | |
| chr8 | 19191734 | | 19191777 | | chr1.trna9-ArgTCT | |
| chr8 | 43105131 | | 43105182 | | chr14.trna7-ArgACG | |
| chr8 | 74689218 | | 74689262 | | chr6.trna4-ArgTCG | |
| chr8 | 117578225 | | 117578270 | | chr14.trna7-ArgACG | |
| chr8 | 122827872 | | 122827911 | | chr11.trna3-ArgTCT | |
| chr8 | 126631420 | | 126631457 | | chr7.trna3-ArgCCT | |
| chr9 | 82127479 | | 82127526 | | chr11.trna3-ArgTCT | |
| chr9 | 85300019 | | 85300070 | | chr3.trna11-ArgACG | |
| chr9 | 109457645 | | 109457688 | | chr17.trna4-ArgTCT | |
| chr10 | 54760400 | | 54760436 | | chr11.trna3-ArgTCT | |
| chr10 | 73707823 | | 73707874 | | chr3.trna11-ArgACG | |
| chr10 | 98319084 | | 98319132 | | chr17.trna4-ArgTCT | |
| chr11 | 11956031 | | 11956080 | | chr3.trna11-ArgACG | |
| chr11 | 35413477 | | 35413540 | | chr6.trna52-ArgTCT | |
| chr11 | 102339078 | | 102339139 | | chr6.trna52-ArgTCT | |
| chr11 | 104112789 | | 104112850 | | chr9.trna4-ArgTCT | |
| chr11 | 104224718 | | 104224753 | | chr17.trna4-ArgTCT | |
| chr11 | 113539318 | | 113539362 | | chr17.trna4-ArgTCT | |
| chr12 | 23894174 | | 23894214 | | chr17.trna4-ArgTCT | |
| chr12 | 75641984 | | 75642030 | | chr6.trna4-ArgTCG | |
| chr13 | 21063263 | | 21063312 | | chr7.trna3-ArgCCT | |
| chr13 | 23567921 | | 23567959 | | chr11.trna3-ArgTCT | |
| chr14 | 59270781 | | 59270826 | | chr1.trna9-ArgTCT | |
| chr14 | 103197446 | | 103197490 | | chr6.trna52-ArgTCT | |
| chr15 | 26657555 | | 26657599 | | chr3.trna11-ArgACG | |
| chr16 | 9911077 | | 9911114 | | chr11.trna3-ArgTCT | |
| chr16 | 11080324 | | 11080363 | | chr1.trna9-ArgTCT | |
| chr16 | 75438755 | | 75438793 | | chr1.trna9-ArgTCT | |
| chr17 | 11796270 | | 11796322 | | chr14.trna7-ArgACG | |
| chr19 | 57126981 | | 57127030 | | chr11.trna3-ArgTCT | |
| chr20 | 3064520 | | 3064574 | | chr9.trna4-ArgTCT | |
| chr21 | 15527457 | | 15527495 | | chr1.trna9-ArgTCT | |
| chr21 | 18120409 | | 18120449 | | chr1.trna9-ArgTCT | |
| chrX | 15727101 | | 15727139 | | chr11.trna3-ArgTCT | |
| chrX | 95514403 | | 95514453 | | chr17.trna18-ArgCCT | |
| chrX | 139658558 | | 139658614 | | chr6.trna52-ArgTCT | |
| chr3 | 53058205 | | 53058266 | | chr1.trna125-AsnGTT | |
| chr10 | 119631836 | | 119631884 | | chr1.trna7-AsnGTT | |
| chr19 | 58146494 | | 58146556 | | chr1.trna104-AsnGTT | |
| chr21 | 30342574 | | 30342612 | | chr1.trna11-AsnGTT | |
| chr2 | 81968070 | | 81968105 | | chr1.trna69-AspGTC | |
| chr2 | 189331566 | | 189331602 | | chr1.trna69-AspGTC | |
| chr2 | 197743183 | | 197743219 | | chr1.trna69-AspGTC | |
| chr2 | 214600666 | | 214600698 | | chr9.trna6-AspGTC | |
| chr2 | 217063110 | | 217063147 | | chr1.trna69-AspGTC | |
| chr6 | 138909975 | | 138910009 | | chr12.trna5-AspGTC | |
| chr10 | 476761 | | 476798 | | chr1.trna69-AspGTC | |
| chr15 | 77126863 | | 77126899 | | chr9.trna6-AspGTC | |
| chr20 | 12549517 | | 12549564 | | chr5.trna22-AspGTC | |
| chr20 | 30463691 | | 30463727 | | chr1.trna69-AspGTC | |
| chr21 | 34667996 | | 34668030 | | chr1.trna69-AspGTC | |
| chrY | 25342900 | | 25342934 | | chr5.trna22-AspGTC | |
| chrY | 25367901 | | 25367935 | | chr1.trna69-AspGTC | |
| chrY | 26957285 | | 26957319 | | chr1.trna69-AspGTC | |
| chrY | 26982287 | | 26982321 | | chr5.trna22-AspGTC | |
| chr1 | 91201618 | | 91201651 | | chr3.trna7-CysGCA | |
| chr1 | 198856993 | | 198857035 | | chr7.trna11-CysGCA | |
| chr1 | 248571376 | | 248571412 | | chr7.trna17-CysGCA | |
| chr2 | 10026425 | | 10026466 | | chr7.trna20-CysGCA | |
| chr2 | 45740194 | | 45740230 | | chr7.trna16-CysGCA | |
| chr2 | 60886994 | | 60887037 | | chr3.trna6-CysGCA | |
| chr2 | 166505020 | | 166505063 | | chr17.trna29-CysGCA | |
| chr2 | 180728986 | | 180729020 | | chr15.trna3-CysGCA | |
| chr2 | 202852569 | | 202852608 | | chr7.trna21-CysGCA | |
| chr3 | 41076147 | | 41076184 | | chr14.trna8-CysGCA | |
| chr3 | 98055197 | | 98055230 | | chr7.trna18-CysGCA | |
| chr3 | 98802349 | | 98802385 | | chr17.trna15-CysGCA | |
| chr3 | 111513866 | | 111513902 | | chr17.trna15-CysGCA | |
| chr3 | 127767619 | | 127767655 | | chr7.trna16-CysGCA | |
| chr4 | 95144295 | | 95144338 | | chr7.trna7-CysGCA | |
| chr4 | 103865125 | | 103865168 | | chr17.trna29-CysGCA | |
| chr4 | 116882297 | | 116882340 | | chr17.trna29-CysGCA | |
| chr4 | 139749102 | | 139749142 | | chr7.trna5-CysGCA | |
| chr4 | 141198021 | | 141198069 | | chr17.trna29-CysGCA | |
| chr5 | 13997596 | | 13997631 | | chr14.trna8-CysGCA | |
| chr5 | 41120021 | | 41120055 | | chr14.trna8-CysGCA | |
| chr5 | 96875031 | | 96875064 | | chr17.trna15-CysGCA | |
| chr5 | 113453522 | | 113453567 | | chr7.trna20-CysGCA | |
| chr5 | 131516473 | | 131516514 | | chr17.trna15-CysGCA | |
| chr5 | 171920589 | | 171920625 | | chr14.trna8-CysGCA | |
| chr6 | 36204501 | | 36204535 | | chr17.trna15-CysGCA | |
| chr6 | 57019473 | | 57019509 | | chr17.trna15-CysGCA | |
| chr6 | 93492803 | | 93492839 | | chr5.trna1-CysACA | |
| chr6 | 143491546 | | 143491590 | | chr7.trna7-CysGCA | |
| chr6 | 156768505 | | 156768545 | | chr17.trna15-CysGCA | |
| chr6 | 164509826 | | 164509858 | | chr14.trna8-CysGCA | |
| chr7 | 2579822 | | 2579857 | | chr7.trna18-CysGCA | |
| chr7 | 69433530 | | 69433575 | | chr17.trna29-CysGCA | |
| chr7 | 129933555 | | 129933611 | | chr7.trna25-CysGCA | |
| chr8 | 49205754 | | 49205797 | | chr7.trna20-CysGCA | |
| chr8 | 88608166 | | 88608201 | | chr17.trna15-CysGCA | |
| chr9 | 541440 | | 541476 | | chr7.trna18-CysGCA | |
| chr9 | 78115511 | | 78115548 | | chr17.trna15-CysGCA | |
| chr9 | 96324149 | | 96324186 | | chr7.trna16-CysGCA | |
| chr9 | 98070002 | | 98070035 | | chr17.trna15-CysGCA | |
| chr9 | 109548243 | | 109548279 | | chr17.trna15-CysGCA | |
| chr9 | 123851330 | | 123851366 | | chr17.trna15-CysGCA | |
| chr10 | 6252978 | | 6253021 | | chr7.trna16-CysGCA | |
| chr10 | 14981699 | | 14981740 | | chr7.trna18-CysGCA | |
| chr10 | 15063693 | | 15063734 | | chr7.trna18-CysGCA | |
| chr10 | 86820426 | | 86820462 | | chr17.trna28-CysGCA | |
| chr10 | 97252272 | | 97252311 | | chr14.trna8-CysGCA | |
| chr10 | 98452480 | | 98452515 | | chr17.trna15-CysGCA | |
| chr10 | 112778896 | | 112778929 | | chr14.trna8-CysGCA | |
| chr10 | 125664631 | | 125664667 | | chr17.trna15-CysGCA | |
| chr10 | 129309528 | | 129309564 | | chr14.trna8-CysGCA | |
| chr11 | 15266857 | | 15266900 | | chr17.trna29-CysGCA | |
| chr11 | 46793218 | | 46793252 | | chr14.trna8-CysGCA | |
| chr11 | 64854323 | | 64854364 | | chr7.trna8-CysGCA | |
| chr11 | 99325279 | | 99325323 | | chr14.trna8-CysGCA | |
| chr12 | 413668 | | 413704 | | chr17.trna15-CysGCA | |
| chr12 | 54020736 | | 54020776 | | chr14.trna8-CysGCA | |
| chr13 | 74715232 | | 74715277 | | chr17.trna29-CysGCA | |
| chr14 | 55883663 | | 55883706 | | chr17.trna29-CysGCA | |
| chr15 | 39163363 | | 39163406 | | chr14.trna8-CysGCA | |
| chr15 | 80269654 | | 80269691 | | chr3.trna12-CysGCA | |
| chr16 | 75019810 | | 75019852 | | chr7.trna14-CysGCA | |
| chr17 | 16891426 | | 16891460 | | chr14.trna8-CysGCA | |
| chr17 | 64441227 | | 64441269 | | chr17.trna29-CysGCA | |
| chr19 | 36538000 | | 36538044 | | chr17.trna15-CysGCA | |
| chr19 | 57904021 | | 57904056 | | chr17.trna15-CysGCA | |
| chr20 | 8593436 | | 8593480 | | chr3.trna6-CysGCA | |
| chr20 | 34076120 | | 34076154 | | chr17.trna15-CysGCA | |
| chr21 | 21108437 | | 21108472 | | chr14.trna8-CysGCA | |
| chrX | 14727843 | | 14727883 | | chr14.trna8-CysGCA | |
| chrX | 24619979 | | 24620020 | | chr17.trna29-CysGCA | |
| chrY | 14834535 | | 14834577 | | chr7.trna16-CysGCA | |
| chr1 | 9862225 | | 9862259 | | chr16.trna21-GlnCTG | |
| chr1 | 18920246 | | 18920281 | | chr15.trna7-GlnCTG | |
| chr1 | 21876327 | | 21876369 | | chr15.trna7-GlnCTG | |
| chr1 | 85000256 | | 85000295 | | chr16.trna21-GlnCTG | |
| chr1 | 94606201 | | 94606243 | | chr6.trna79-GlnTTG | |
| chr1 | 108432811 | | 108432843 | | chr15.trna7-GlnCTG | |
| chr1 | 111532017 | | 111532053 | | chr1.trna15-GlnCTG | |
| chr2 | 30787359 | | 30787391 | | chr1.trna15-GlnCTG | |
| chr2 | 33795828 | | 33795870 | | chr9.trna5-GlnCTG | |
| chr2 | 39047517 | | 39047559 | | chr1.trna15-GlnCTG | |
| chr2 | 131586208 | | 131586247 | | chr6.trna64-GlnTTG | |
| chr2 | 141685670 | | 141685706 | | chr1.trna15-GlnCTG | |
| chr2 | 151302759 | | 151302794 | | chr1.trna15-GlnCTG | |
| chr2 | 158741349 | | 158741396 | | chr6.trna42-GlnCTG | |
| chr3 | 110359089 | | 110359131 | | chr1.trna15-GlnCTG | |
| chr3 | 128844980 | | 128845017 | | chr1.trna15-GlnCTG | |
| chr3 | 154247090 | | 154247132 | | chr1.trna15-GlnCTG | |
| chr4 | 42675707 | | 42675738 | | chr12.trna15-GlnTTG | |
| chr4 | 57971857 | | 57971900 | | chr16.trna21-GlnCTG | |
| chr4 | 111336352 | | 111336397 | | chr1.trna15-GlnCTG | |
| chr4 | 133585386 | | 133585427 | | chr1.trna120-GlnCTG | |
| chr4 | 167533146 | | 167533178 | | chr6.trna131-GlnCTG | |
| chr4 | 186117074 | | 186117107 | | chr1.trna15-GlnCTG | |
| chr5 | 83927443 | | 83927497 | | chr1.trna23-GlnCTG | |
| chr5 | 118650348 | | 118650393 | | chr16.trna21-GlnCTG | |
| chr6 | 10508828 | | 10508865 | | chr15.trna7-GlnCTG | |
| chr6 | 142361996 | | 142362055 | | chr2.trna28-GlnTTG | |
| chr7 | 33624115 | | 33624151 | | chr1.trna15-GlnCTG | |
| chr7 | 34625221 | | 34625278 | | chr16.trna21-GlnCTG | |
| chr7 | 53863656 | | 53863693 | | chr6.trna131-GlnCTG | |
| chr7 | 81030381 | | 81030442 | | chr20.trna1-GlnCTG | |
| chr7 | 89903945 | | 89903981 | | chr1.trna15-GlnCTG | |
| chr8 | 24809496 | | 24809533 | | chr15.trna7-GlnCTG | |
| chr8 | 85880335 | | 85880379 | | chr6.trna42-GlnCTG | |
| chr8 | 102797261 | | 102797314 | | chr9.trna5-GlnCTG | |
| chr8 | 114225198 | | 114225240 | | chr1.trna15-GlnCTG | |
| chr8 | 121563534 | | 121563576 | | chr1.trna15-GlnCTG | |
| chr8 | 125728021 | | 125728058 | | chr16.trna21-GlnCTG | |
| chr9 | 86861415 | | 86861457 | | chr1.trna15-GlnCTG | |
| chr11 | 7721505 | | 7721550 | | chr1.trna15-GlnCTG | |
| chr11 | 35001077 | | 35001114 | | chr15.trna7-GlnCTG | |
| chr11 | 40722222 | | 40722264 | | chr1.trna15-GlnCTG | |
| chr11 | 62126498 | | 62126535 | | chr15.trna7-GlnCTG | |
| chr11 | 62388461 | | 62388502 | | chr15.trna7-GlnCTG | |
| chr11 | 80916998 | | 80917037 | | chr12.trna15-GlnTTG | |
| chr11 | 123835841 | | 123835886 | | chr1.trna15-GlnCTG | |
| chr12 | 1250604 | | 1250649 | | chr1.trna15-GlnCTG | |
| chr12 | 41387699 | | 41387739 | | chr1.trna120-GlnCTG | |
| chr12 | 64771670 | | 64771712 | | chr9.trna5-GlnCTG | |
| chr12 | 84601691 | | 84601732 | | chr1.trna15-GlnCTG | |
| chr12 | 96011924 | | 96011958 | | chr15.trna7-GlnCTG | |
| chr13 | 26867159 | | 26867201 | | chr1.trna112-GlnCTG | |
| chr13 | 54250580 | | 54250627 | | chr1.trna15-GlnCTG | |
| chr13 | 109615389 | | 109615447 | | chr9.trna5-GlnCTG | |
| chr14 | 42207886 | | 42207933 | | chr1.trna15-GlnCTG | |
| chr14 | 76869616 | | 76869672 | | chr20.trna1-GlnCTG | |
| chr15 | 36252806 | | 36252849 | | chr16.trna21-GlnCTG | |
| chr15 | 72394875 | | 72394917 | | chr1.trna15-GlnCTG | |
| chr18 | 4070380 | | 4070420 | | chr1.trna15-GlnCTG | |
| chr18 | 32061880 | | 32061922 | | chr1.trna112-GlnCTG | |
| chr18 | 45816176 | | 45816213 | | chr15.trna7-GlnCTG | |
| chr19 | 10963143 | | 10963180 | | chr6.trna131-GlnCTG | |
| chr19 | 32486717 | | 32486757 | | chr1.trna15-GlnCTG | |
| chrX | 363163 | | 363212 | | chr1.trna22-GlnCTG | |
| chrX | 25289840 | | 25289894 | | chr9.trna5-GlnCTG | |
| chrX | 27302349 | | 27302385 | | chr1.trna15-GlnCTG | |
| chrX | 85256924 | | 85256960 | | chr6.trna131-GlnCTG | |
| chrX | 106573845 | | 106573887 | | chr16.trna21-GlnCTG | |
| chrX | 112256145 | | 112256182 | | chr1.trna15-GlnCTG | |
| chrX | 134118666 | | 134118708 | | chr16.trna21-GlnCTG | |
| chrY | 313163 | | 313212 | | chr1.trna22-GlnCTG | |
| chr1 | 36264895 | | 36264932 | | chr1.trna116-GluCTC | |
| chr1 | 39579940 | | 39579976 | | chr1.trna5-GluTTC | |
| chr1 | 198075139 | | 198075175 | | chr1.trna116-GluCTC | |
| chr2 | 140030405 | | 140030440 | | chr1.trna116-GluCTC | |
| chr2 | 205969571 | | 205969607 | | chr1.trna5-GluTTC | |
| chr3 | 21591504 | | 21591540 | | chr1.trna59-GluCTC | |
| chr3 | 72783915 | | 72783951 | | chr1.trna59-GluCTC | |
| chr3 | 155378883 | | 155378924 | | chr2.trna25-GluCTC | |
| chr4 | 86243639 | | 86243674 | | chr1.trna116-GluCTC | |
| chr5 | 96908084 | | 96908120 | | chr1.trna5-GluTTC | |
| chr5 | 118824428 | | 118824463 | | chr1.trna116-GluCTC | |
| chr8 | 125785385 | | 125785421 | | chr1.trna116-GluCTC | |
| chr9 | 115192403 | | 115192439 | | chr13.trna2-GluCTC | |
| chr10 | 30618657 | | 30618693 | | chr1.trna105-GluTTC | |
| chr11 | 91818550 | | 91818588 | | chr1.trna116-GluCTC | |
| chr15 | 73212640 | | 73212676 | | chr1.trna116-GluCTC | |
| chr17 | 40409950 | | 40409986 | | chr20.trna7-GluCTC | |
| chr18 | 19393377 | | 19393417 | | chr2.trna18-GluCTC | |
| chr18 | 31480739 | | 31480776 | | chr14.trna14-GluTTC | |
| chr21 | 37725742 | | 37725777 | | chr1.trna5-GluTTC | |
| chrX | 20630297 | | 20630333 | | chr1.trna116-GluCTC | |
| chrX | 50374801 | | 50374837 | | chr1.trna5-GluTTC | |
| chrX | 83508946 | | 83508979 | | chr1.trna116-GluCTC | |
| chr1 | 3041068 | | 3041105 | | chr17.trna10-GlyTCC | |
| chr1 | 86865158 | | 86865195 | | chr1.trna117-GlyTCC | |
| chr1 | 212748435 | | 212748471 | | chr16.trna34-GlyCCC | |
| chr1 | 229673604 | | 229673639 | | chr1.trna68-GlyGCC | |
| chr1 | 230556934 | | 230556970 | | chr19.trna2-GlyTCC | |
| chr2 | 37424520 | | 37424555 | | chr1.trna117-GlyTCC | |
| chr2 | 40742213 | | 40742248 | | chr19.trna2-GlyTCC | |
| chr2 | 103340479 | | 103340512 | | chr1.trna117-GlyTCC | |
| chr2 | 122716394 | | 122716431 | | chr1.trna117-GlyTCC | |
| chr2 | 127599409 | | 127599442 | | chr1.trna117-GlyTCC | |
| chr2 | 133718522 | | 133718559 | | chr1.trna117-GlyTCC | |
| chr2 | 197053936 | | 197053974 | | chr1.trna91-GlyCCC | |
| chr2 | 199695024 | | 199695061 | | chr17.trna10-GlyTCC | |
| chr2 | 211796824 | | 211796861 | | chr19.trna2-GlyTCC | |
| chr3 | 15549499 | | 15549535 | | chr1.trna133-GlyCCC | |
| chr3 | 15847980 | | 15848018 | | chr1.trna117-GlyTCC | |
| chr3 | 22547882 | | 22547916 | | chr16.trna34-GlyCCC | |
| chr3 | 34742646 | | 34742685 | | chr1.trna117-GlyTCC | |
| chr3 | 133162574 | | 133162603 | | chr16.trna34-GlyCCC | |
| chr3 | 135929722 | | 135929755 | | chr1.trna117-GlyTCC | |
| chr3 | 148394630 | | 148394667 | | chr1.trna117-GlyTCC | |
| chr3 | 155163377 | | 155163411 | | chr16.trna34-GlyCCC | |
| chr4 | 41184469 | | 41184506 | | chr1.trna117-GlyTCC | |
| chr4 | 64509411 | | 64509446 | | chr1.trna91-GlyCCC | |
| chr4 | 88527422 | | 88527457 | | chr1.trna133-GlyCCC | |
| chr4 | 91798914 | | 91798949 | | chr16.trna34-GlyCCC | |
| chr4 | 121332541 | | 121332575 | | chr18.trna2-GlyTCC | |
| chr4 | 122880464 | | 122880498 | | chr16.trna34-GlyCCC | |
| chr4 | 190512250 | | 190512287 | | chr1.trna117-GlyTCC | |
| chr5 | 2455462 | | 2455498 | | chr1.trna117-GlyTCC | |
| chr5 | 16166039 | | 16166074 | | chr1.trna91-GlyCCC | |
| chr5 | 54080799 | | 54080835 | | chr17.trna10-GlyTCC | |
| chr5 | 155564766 | | 155564812 | | chr16.trna18-GlyGCC | |
| chr5 | 167988020 | | 167988057 | | chr1.trna117-GlyTCC | |
| chr6 | 2999554 | | 2999588 | | chr16.trna34-GlyCCC | |
| chr6 | 42529263 | | 42529307 | | chr1.trna82-GlyTCC | |
| chr6 | 49644855 | | 49644890 | | chr16.trna34-GlyCCC | |
| chr6 | 73549633 | | 73549668 | | chr1.trna35-GlyGCC | |
| chr6 | 119031819 | | 119031855 | | chr17.trna10-GlyTCC | |
| chr6 | 122964534 | | 122964570 | | chr1.trna117-GlyTCC | |
| chr6 | 134617610 | | 134617647 | | chr1.trna117-GlyTCC | |
| chr6 | 138724246 | | 138724283 | | chr1.trna117-GlyTCC | |
| chr6 | 155086342 | | 155086380 | | chr1.trna117-GlyTCC | |
| chr6 | 167297415 | | 167297452 | | chr1.trna117-GlyTCC | |
| chr7 | 35060765 | | 35060802 | | chr1.trna117-GlyTCC | |
| chr7 | 44444746 | | 44444783 | | chr1.trna117-GlyTCC | |
| chr7 | 44730048 | | 44730079 | | chr17.trna10-GlyTCC | |
| chr7 | 68601780 | | 68601817 | | chr19.trna2-GlyTCC | |
| chr7 | 71595436 | | 71595471 | | chr1.trna117-GlyTCC | |
| chr7 | 97113984 | | 97114018 | | chr16.trna34-GlyCCC | |
| chr7 | 105394938 | | 105394974 | | chr1.trna117-GlyTCC | |
| chr7 | 124967603 | | 124967638 | | chr1.trna35-GlyGCC | |
| chr7 | 130596195 | | 130596231 | | chr1.trna82-GlyTCC | |
| chr9 | 5194372 | | 5194409 | | chr1.trna117-GlyTCC | |
| chr9 | 31569235 | | 31569272 | | chr1.trna117-GlyTCC | |
| chr9 | 135806884 | | 135806921 | | chr1.trna117-GlyTCC | |
| chr10 | 965268 | | 965313 | | chr1.trna35-GlyGCC | |
| chr10 | 17350048 | | 17350084 | | chr19.trna2-GlyTCC | |
| chr10 | 22778379 | | 22778416 | | chr1.trna117-GlyTCC | |
| chr10 | 68915503 | | 68915540 | | chr19.trna2-GlyTCC | |
| chr10 | 70318709 | | 70318743 | | chr19.trna2-GlyTCC | |
| chr10 | 71982317 | | 71982352 | | chr1.trna68-GlyGCC | |
| chr10 | 111737698 | | 111737731 | | chr1.trna117-GlyTCC | |
| chr11 | 11532111 | | 11532147 | | chr1.trna117-GlyTCC | |
| chr11 | 13910277 | | 13910316 | | chr1.trna117-GlyTCC | |
| chr11 | 31858423 | | 31858458 | | chr1.trna35-GlyGCC | |
| chr11 | 113856086 | | 113856121 | | chr1.trna117-GlyTCC | |
| chr12 | 5006176 | | 5006210 | | chr1.trna35-GlyGCC | |
| chr12 | 58889118 | | 58889157 | | chr19.trna2-GlyTCC | |
| chr12 | 70941146 | | 70941183 | | chr19.trna2-GlyTCC | |
| chr12 | 121111555 | | 121111589 | | chr1.trna91-GlyCCC | |
| chr12 | 124599675 | | 124599711 | | chr1.trna117-GlyTCC | |
| chr13 | 64794644 | | 64794675 | | chr1.trna82-GlyTCC | |
| chr13 | 89494694 | | 89494729 | | chr1.trna68-GlyGCC | |
| chr13 | 95203007 | | 95203044 | | chr19.trna2-GlyTCC | |
| chr14 | 32373682 | | 32373715 | | chr17.trna10-GlyTCC | |
| chr14 | 66708591 | | 66708628 | | chr1.trna117-GlyTCC | |
| chr14 | 73786038 | | 73786075 | | chr1.trna117-GlyTCC | |
| chr14 | 77048572 | | 77048609 | | chr1.trna117-GlyTCC | |
| chr14 | 82126929 | | 82126962 | | chr16.trna18-GlyGCC | |
| chr14 | 102246129 | | 102246168 | | chr1.trna117-GlyTCC | |
| chr15 | 74962719 | | 74962756 | | chr1.trna117-GlyTCC | |
| chr15 | 78338628 | | 78338662 | | chr16.trna25-GlyGCC | |
| chr16 | 67659510 | | 67659547 | | chr17.trna10-GlyTCC | |
| chr17 | 74884893 | | 74884929 | | chr1.trna117-GlyTCC | |
| chr18 | 22687555 | | 22687591 | | chr17.trna10-GlyTCC | |
| chr18 | 41720884 | | 41720921 | | chr17.trna10-GlyTCC | |
| chr18 | 55998346 | | 55998383 | | chr1.trna117-GlyTCC | |
| chr19 | 8793667 | | 8793703 | | chr16.trna25-GlyGCC | |
| chr19 | 43177835 | | 43177872 | | chr1.trna117-GlyTCC | |
| chr19 | 53615664 | | 53615711 | | chr1.trna117-GlyTCC | |
| chr19 | 58472364 | | 58472402 | | chr1.trna117-GlyTCC | |
| chr20 | 24382438 | | 24382475 | | chr1.trna117-GlyTCC | |
| chr21 | 23099384 | | 23099415 | | chr1.trna68-GlyGCC | |
| chr22 | 27314981 | | 27315016 | | chr17.trna13-GlyCCC | |
| chrX | 88080386 | | 88080423 | | chr1.trna117-GlyTCC | |
| chr2 | 225798823 | | 225798879 | | chr1.trna111-HisGTG | |
| chr7 | 82564998 | | 82565033 | | chr1.trna111-HisGTG | |
| chr10 | 21826175 | | 21826210 | | chr1.trna111-HisGTG | |
| chr11 | 17272944 | | 17272979 | | chr1.trna111-HisGTG | |
| chr11 | 109473924 | | 109473959 | | chr1.trna111-HisGTG | |
| chr19 | 1638691 | | 1638726 | | chr1.trna111-HisGTG | |
| chr19 | 47114779 | | 47114814 | | chr1.trna111-HisGTG | |
| chr20 | 9984920 | | 9984955 | | chr1.trna111-HisGTG | |
| chr22 | 24214712 | | 24214749 | | chr1.trna106-HisGTG | |
| chr1 | 38744787 | | 38744835 | | chr2.trna5-IleTAT | |
| chr1 | 69416979 | | 69417018 | | chr6.trna63-IleTAT | |
| chr1 | 160326727 | | 160326763 | | chr6.trna80-IleAAT | |
| chr1 | 176276294 | | 176276330 | | chr6.trna153-IleAAT | |
| chr1 | 209750061 | | 209750104 | | chr2.trna5-IleTAT | |
| chr1 | 241105566 | | 241105608 | | chr6.trna63-IleTAT | |
| chr2 | 51163329 | | 51163367 | | chr6.trna163-IleAAT | |
| chr2 | 227307897 | | 227307936 | | chr6.trna153-IleAAT | |
| chr2 | 230103189 | | 230103229 | | chr6.trna63-IleTAT | |
| chr3 | 55801231 | | 55801271 | | chr6.trna63-IleTAT | |
| chr3 | 68319302 | | 68319347 | | chr19.trna10-IleTAT | |
| chr3 | 68357660 | | 68357700 | | chr6.trna63-IleTAT | |
| chr3 | 151657141 | | 151657181 | | chr19.trna10-IleTAT | |
| chr3 | 180213941 | | 180213979 | | chr6.trna63-IleTAT | |
| chr4 | 55649695 | | 55649735 | | chr2.trna5-IleTAT | |
| chr4 | 156522102 | | 156522142 | | chr19.trna10-IleTAT | |
| chr5 | 82105677 | | 82105717 | | chr19.trna10-IleTAT | |
| chr5 | 110356659 | | 110356696 | | chr19.trna10-IleTAT | |
| chr5 | 118246881 | | 118246921 | | chr19.trna10-IleTAT | |
| chr5 | 161840577 | | 161840612 | | chr6.trna59-IleAAT | |
| chr6 | 480546 | | 480584 | | chr19.trna10-IleTAT | |
| chr6 | 42513330 | | 42513395 | | chr2.trna5-IleTAT | |
| chr6 | 99074658 | | 99074698 | | chr6.trna63-IleTAT | |
| chr6 | 120823007 | | 120823045 | | chr6.trna63-IleTAT | |
| chr6 | 165899892 | | 165899934 | | chr19.trna10-IleTAT | |
| chr7 | 78873388 | | 78873425 | | chr6.trna55-IleTAT | |
| chr7 | 95095378 | | 95095415 | | chr6.trna153-IleAAT | |
| chr7 | 122175990 | | 122176025 | | chr17.trna34-IleAAT | |
| chr7 | 148274634 | | 148274672 | | chr6.trna153-IleAAT | |
| chr7 | 155723810 | | 155723850 | | chr2.trna5-IleTAT | |
| chr9 | 19685038 | | 19685078 | | chr2.trna5-IleTAT | |
| chr9 | 94550154 | | 94550192 | | chr6.trna153-IleAAT | |
| chr9 | 110200660 | | 110200698 | | chr2.trna5-IleTAT | |
| chr10 | 9863687 | | 9863738 | | chr6.trna63-IleTAT | |
| chr10 | 30009335 | | 30009375 | | chr6.trna63-IleTAT | |
| chr10 | 58801967 | | 58802007 | | chr2.trna5-IleTAT | |
| chr10 | 64085552 | | 64085594 | | chr19.trna10-IleTAT | |
| chr10 | 74732137 | | 74732178 | | chr6.trna59-IleAAT | |
| chr10 | 75281056 | | 75281113 | | chr19.trna10-IleTAT | |
| chr10 | 77154266 | | 77154309 | | chr2.trna5-IleTAT | |
| chr10 | 114588051 | | 114588091 | | chr6.trna63-IleTAT | |
| chr11 | 16590981 | | 16591036 | | chr6.trna29-IleTAT | |
| chr11 | 19163539 | | 19163577 | | chr6.trna59-IleAAT | |
| chr11 | 73217390 | | 73217430 | | chr19.trna10-IleTAT | |
| chr11 | 90124272 | | 90124327 | | chr6.trna29-IleTAT | |
| chr11 | 94803733 | | 94803771 | | chr6.trna59-IleAAT | |
| chr11 | 105866798 | | 105866843 | | chr6.trna153-IleAAT | |
| chr12 | 18298064 | | 18298102 | | chr6.trna38-IleAAT | |
| chr12 | 23308352 | | 23308391 | | chr6.trna63-IleTAT | |
| chr12 | 105658491 | | 105658531 | | chr6.trna63-IleTAT | |
| chr12 | 120045142 | | 120045184 | | chr6.trna55-IleTAT | |
| chr12 | 126068966 | | 126069003 | | chr6.trna59-IleAAT | |
| chr13 | 50445088 | | 50445126 | | chrX.trna5-IleGAT | |
| chr13 | 54830869 | | 54830909 | | chr6.trna63-IleTAT | |
| chr14 | 21828873 | | 21828910 | | chr6.trna59-IleAAT | |
| chr14 | 65336094 | | 65336132 | | chr6.trna80-IleAAT | |
| chr14 | 66624582 | | 66624622 | | chr2.trna5-IleTAT | |
| chr16 | 10531190 | | 10531224 | | chr6.trna153-IleAAT | |
| chr16 | 26269506 | | 26269551 | | chr2.trna5-IleTAT | |
| chr16 | 48903014 | | 48903054 | | chr19.trna10-IleTAT | |
| chr16 | 66159299 | | 66159335 | | chr6.trna59-IleAAT | |
| chr17 | 1448903 | | 1448947 | | chr2.trna5-IleTAT | |
| chr17 | 38738606 | | 38738647 | | chr6.trna63-IleTAT | |
| chr17 | 73264416 | | 73264456 | | chr6.trna63-IleTAT | |
| chr18 | 23498947 | | 23498984 | | chr6.trna59-IleAAT | |
| chr18 | 69955498 | | 69955533 | | chr6.trna59-IleAAT | |
| chr20 | 9365376 | | 9365415 | | chr6.trna59-IleAAT | |
| chr20 | 15017736 | | 15017785 | | chr2.trna5-IleTAT | |
| chr21 | 16449381 | | 16449424 | | chr6.trna29-IleTAT | |
| chr22 | 44327171 | | 44327217 | | chr2.trna5-IleTAT | |
| chrX | 25083300 | | 25083344 | | chr2.trna5-IleTAT | |
| chrX | 42105286 | | 42105327 | | chr6.trna63-IleTAT | |
| chrX | 44945041 | | 44945081 | | chr2.trna5-IleTAT | |
| chrX | 105906395 | | 105906432 | | chr6.trna165-IleAAT | |
| chrX | 115426973 | | 115427025 | | chr19.trna10-IleTAT | |
| chr1 | 242247809 | | 242247851 | | chr1.trna58-LeuCAA | |
| chr1 | 242466324 | | 242466365 | | chr16.trna17-LeuCAG | |
| chr3 | 130577648 | | 130577685 | | chr11.trna4-LeuTAA | |
| chr3 | 178811691 | | 178811778 | | chr6.trna140-LeuCAA | |
| chr5 | 10231231 | | 10231281 | | chr6.trna141-LeuCAA | |
| chr5 | 52158127 | | 52158173 | | chr16.trna17-LeuCAG | |
| chr5 | 58322328 | | 58322368 | | chr6.trna140-LeuCAA | |
| chr7 | 33374299 | | 33374333 | | chr1.trna60-LeuTAA | |
| chr8 | 40197163 | | 40197204 | | chr6.trna140-LeuCAA | |
| chr9 | 31042969 | | 31043007 | | chr1.trna58-LeuCAA | |
| chr10 | 24253790 | | 24253832 | | chr14.trna1-LeuAAG | |
| chr10 | 38584628 | | 38584669 | | chr1.trna34-LeuCAG | |
| chr10 | 95707851 | | 95707887 | | chr6.trna78-LeuAAG | |
| chr14 | 28507636 | | 28507672 | | chr1.trna34-LeuCAG | |
| chrX | 45385270 | | 45385308 | | chr1.trna58-LeuCAA | |
| chrX | 108240760 | | 108240791 | | chr14.trna2-LeuTAG | |
| chr1 | 163051440 | | 163051477 | | chr1.trna128-LysCTT | |
| chr1 | 189525951 | | 189525986 | | chr16.trna30-LysCTT | |
| chr2 | 44613322 | | 44613351 | | chr1.trna128-LysCTT | |
| chr2 | 72215338 | | 72215380 | | chr1.trna128-LysCTT | |
| chr2 | 108090709 | | 108090748 | | chr19.trna7-LysTTT | |
| chr2 | 113538733 | | 113538773 | | chr1.trna128-LysCTT | |
| chr3 | 149458711 | | 149458746 | | chr1.trna119-LysCTT | |
| chr3 | 189376628 | | 189376663 | | chr1.trna128-LysCTT | |
| chr4 | 52899312 | | 52899349 | | chr1.trna128-LysCTT | |
| chr4 | 70990364 | | 70990405 | | chr1.trna119-LysCTT | |
| chr5 | 127497639 | | 127497675 | | chr16.trna5-LysCTT | |
| chr6 | 24110377 | | 24110412 | | chr1.trna119-LysCTT | |
| chr7 | 32321279 | | 32321314 | | chr1.trna128-LysCTT | |
| chr7 | 43590959 | | 43591015 | | chr1.trna128-LysCTT | |
| chr8 | 17737817 | | 17737853 | | chr1.trna119-LysCTT | |
| chr8 | 66387833 | | 66387870 | | chr12.trna1-LysTTT | |
| chr9 | 5934740 | | 5934777 | | chr16.trna32-LysCTT | |
| chr9 | 100414945 | | 100414983 | | chr19.trna5-LysCTT | |
| chr11 | 109709716 | | 109709753 | | chr1.trna119-LysCTT | |
| chr12 | 15871590 | | 15871628 | | chr1.trna128-LysCTT | |
| chr12 | 56761200 | | 56761235 | | chr1.trna119-LysCTT | |
| chr12 | 98546511 | | 98546548 | | chr1.trna119-LysCTT | |
| chr13 | 29940639 | | 29940670 | | chr1.trna119-LysCTT | |
| chr13 | 42805952 | | 42805988 | | chr1.trna119-LysCTT | |
| chr15 | 81734431 | | 81734468 | | chr1.trna119-LysCTT | |
| chr15 | 90327894 | | 90327923 | | chr16.trna30-LysCTT | |
| chr16 | 53675472 | | 53675508 | | chr1.trna119-LysCTT | |
| chr18 | 43123360 | | 43123395 | | chr1.trna128-LysCTT | |
| chrX | 47349470 | | 47349506 | | chr1.trna128-LysCTT | |
| chrX | 128354061 | | 128354110 | | chr16.trna23-LysTTT | |
| chrX | 154038624 | | 154038660 | | chr19.trna6-LysCTT | |
| chr1 | 17509435 | | 17509472 | | chr6.trna75-MetCAT | |
| chr1 | 111475574 | | 111475609 | | chr6.trna21-MetCAT | |
| chr1 | 146953349 | | 146953382 | | chr16.trna20-MetCAT | |
| chr1 | 190612339 | | 190612383 | | chr8.trna10-MetCAT | |
| chr2 | 119262686 | | 119262726 | | chr8.trna10-MetCAT | |
| chr2 | 135574486 | | 135574523 | | chr6.trna75-MetCAT | |
| chr2 | 176772560 | | 176772597 | | chr1.trna32-MetCAT | |
| chr2 | 177125140 | | 177125176 | | chr1.trna32-MetCAT | |
| chr2 | 181875141 | | 181875185 | | chr6.trna21-MetCAT | |
| chr2 | 187972243 | | 187972279 | | chr8.trna10-MetCAT | |
| chr2 | 201911899 | | 201911943 | | chr6.trna21-MetCAT | |
| chr2 | 216912663 | | 216912700 | | chr6.trna75-MetCAT | |
| chr3 | 110807438 | | 110807473 | | chr1.trna32-MetCAT | |
| chr3 | 196979728 | | 196979765 | | chr16.trna20-MetCAT | |
| chr4 | 31924298 | | 31924334 | | chr6.trna75-MetCAT | |
| chr4 | 37709360 | | 37709397 | | chr16.trna22-MetCAT | |
| chr4 | 53274842 | | 53274876 | | chr1.trna32-MetCAT | |
| chr4 | 146483384 | | 146483420 | | chr1.trna32-MetCAT | |
| chr5 | 12314420 | | 12314463 | | chr6.trna21-MetCAT | |
| chr5 | 14693593 | | 14693652 | | chr16.trna20-MetCAT | |
| chr5 | 93841585 | | 93841639 | | chr6.trna21-MetCAT | |
| chr5 | 126562592 | | 126562629 | | chr8.trna10-MetCAT | |
| chr5 | 171814000 | | 171814049 | | chr6.trna75-MetCAT | |
| chr5 | 177711803 | | 177711846 | | chr16.trna20-MetCAT | |
| chr6 | 25418874 | | 25418911 | | chr6.trna162-MetCAT | |
| chr6 | 84348286 | | 84348323 | | chr6.trna75-MetCAT | |
| chr6 | 90030115 | | 90030150 | | chr6.trna75-MetCAT | |
| chr6 | 125701631 | | 125701673 | | chr6.trna75-MetCAT | |
| chr6 | 151823465 | | 151823501 | | chr16.trna22-MetCAT | |
| chr6 | 158034698 | | 158034741 | | chr16.trna22-MetCAT | |
| chr7 | 76118768 | | 76118800 | | chr1.trna32-MetCAT | |
| chr7 | 76616871 | | 76616903 | | chr1.trna32-MetCAT | |
| chr7 | 89935576 | | 89935612 | | chr6.trna61-MetCAT | |
| chr7 | 130576074 | | 130576121 | | chr6.trna61-MetCAT | |
| chr8 | 3505904 | | 3505941 | | chr16.trna22-MetCAT | |
| chr8 | 125629034 | | 125629080 | | chr6.trna75-MetCAT | |
| chr8 | 135989178 | | 135989214 | | chr6.trna61-MetCAT | |
| chr9 | 82320576 | | 82320618 | | chr16.trna20-MetCAT | |
| chr10 | 35141146 | | 35141175 | | chr16.trna22-MetCAT | |
| chr11 | 60984592 | | 60984623 | | chr1.trna32-MetCAT | |
| chr11 | 61003421 | | 61003452 | | chr1.trna32-MetCAT | |
| chr11 | 72159451 | | 72159488 | | chr16.trna22-MetCAT | |
| chr11 | 106285321 | | 106285369 | | chr16.trna20-MetCAT | |
| chr12 | 17094599 | | 17094642 | | chr6.trna21-MetCAT | |
| chr12 | 33572511 | | 33572566 | | chr6.trna75-MetCAT | |
| chr13 | 81433700 | | 81433738 | | chr6.trna75-MetCAT | |
| chr14 | 76973439 | | 76973483 | | chr16.trna20-MetCAT | |
| chr16 | 34361226 | | 34361263 | | chr6.trna75-MetCAT | |
| chr16 | 56746291 | | 56746331 | | chr6.trna21-MetCAT | |
| chr16 | 58414791 | | 58414824 | | chr6.trna75-MetCAT | |
| chr18 | 38772394 | | 38772432 | | chr6.trna75-MetCAT | |
| chr19 | 23766251 | | 23766289 | | chr6.trna75-MetCAT | |
| chr19 | 44526364 | | 44526399 | | chr6.trna75-MetCAT | |
| chr19 | 44835203 | | 44835247 | | chr6.trna21-MetCAT | |
| chr20 | 21418400 | | 21418435 | | chr6.trna21-MetCAT | |
| chr21 | 37273509 | | 37273552 | | chr6.trna21-MetCAT | |
| chrX | 12235532 | | 12235584 | | chr16.trna20-MetCAT | |
| chrX | 33367771 | | 33367807 | | chr6.trna75-MetCAT | |
| chrX | 66004486 | | 66004520 | | chr6.trna21-MetCAT | |
| chrX | 76851713 | | 76851758 | | chr6.trna75-MetCAT | |
| chr1 | 35998350 | | 35998389 | | chr6.trna112-PheGAA | |
| chr1 | 79161173 | | 79161210 | | chr6.trna106-PheGAA | |
| chr1 | 157035131 | | 157035176 | | chr6.trna56-PheGAA | |
| chr1 | 213020170 | | 213020206 | | chr6.trna88-PheGAA | |
| chr1 | 233943582 | | 233943619 | | chr11.trna15-PheGAA | |
| chr2 | 73246472 | | 73246506 | | chr11.trna13-PheGAA | |
| chr4 | 78877797 | | 78877831 | | chr6.trna56-PheGAA | |
| chr4 | 173887183 | | 173887225 | | chr11.trna15-PheGAA | |
| chr4 | 174532535 | | 174532581 | | chr6.trna56-PheGAA | |
| chr4 | 183408720 | | 183408763 | | chr11.trna15-PheGAA | |
| chr5 | 110115129 | | 110115174 | | chr6.trna56-PheGAA | |
| chr5 | 131487771 | | 131487816 | | chr6.trna56-PheGAA | |
| chr5 | 179574898 | | 179574935 | | chr6.trna112-PheGAA | |
| chr6 | 118120826 | | 118120871 | | chr6.trna56-PheGAA | |
| chr8 | 72697783 | | 72697828 | | chr6.trna56-PheGAA | |
| chr9 | 112366249 | | 112366286 | | chr6.trna112-PheGAA | |
| chr10 | 89658551 | | 89658596 | | chr6.trna56-PheGAA | |
| chr11 | 6989002 | | 6989048 | | chr6.trna56-PheGAA | |
| chr12 | 19766759 | | 19766796 | | chr11.trna15-PheGAA | |
| chr13 | 64791523 | | 64791560 | | chr6.trna112-PheGAA | |
| chr14 | 87921874 | | 87921909 | | chr6.trna112-PheGAA | |
| chr15 | 20688080 | | 20688117 | | chr6.trna112-PheGAA | |
| chr15 | 23355530 | | 23355567 | | chr6.trna112-PheGAA | |
| chr15 | 28543972 | | 28544009 | | chr6.trna112-PheGAA | |
| chr15 | 28857858 | | 28857895 | | chr6.trna112-PheGAA | |
| chr15 | 77613982 | | 77614027 | | chr6.trna56-PheGAA | |
| chr15 | 84549838 | | 84549883 | | chr11.trna13-PheGAA | |
| chr18 | 30829215 | | 30829260 | | chr6.trna56-PheGAA | |
| chrY | 16826642 | | 16826687 | | chr6.trna56-PheGAA | |
| chr2 | 80656558 | | 80656596 | | chr10.trna1-ProGGG | |
| chr9 | 117105213 | | 117105274 | | chr1.trna61-ProTGG | |
| chr14 | 75358453 | | 75358488 | | chr16.trna11-ProAGG | |
| chr1 | 45932805 | | 45932843 | | chr6.trna31-SerGCT | |
| chr2 | 136691031 | | 136691068 | | chr6.trna31-SerGCT | |
| chr2 | 228625137 | | 228625174 | | chr15.trna10-SerGCT | |
| chr3 | 104974905 | | 104974943 | | chr6.trna137-SerCGA | |
| chr6 | 130293594 | | 130293628 | | chr6.trna31-SerGCT | |
| chr9 | 2130352 | | 2130390 | | chr6.trna145-SerAGA | |
| chr12 | 13088545 | | 13088578 | | chr6.trna41-SerACT | |
| chrX | 12060106 | | 12060140 | | chr7.trna12-SerAGA | |
| chrX | 15651440 | | 15651477 | | chr6.trna148-SerTGA | |
| chr1 | 25809189 | | 25809239 | | chr4.trna5-SupTTA | |
| chr2 | 39648406 | | 39648463 | | chr14.trna11-SupCTA | |
| chr2 | 107708792 | | 107708848 | | chr14.trna11-SupCTA | |
| chr6 | 146916502 | | 146916554 | | chr14.trna11-SupCTA | |
| chr12 | 103581162 | | 103581221 | | chr4.trna5-SupTTA | |
| chr17 | 28611096 | | 28611156 | | chr4.trna5-SupTTA | |
| chr1 | 14938794 | | 14938828 | | chr14.trna21-ThrTGT | |
| chr1 | 36325797 | | 36325836 | | chr6.trna125-ThrCGT | |
| chr1 | 44099855 | | 44099892 | | chr16.trna15-ThrCGT | |
| chr1 | 238349762 | | 238349802 | | chr17.trna36-ThrAGT | |
| chr3 | 89957336 | | 89957372 | | chr14.trna20-ThrTGT | |
| chr3 | 111840748 | | 111840787 | | chr6.trna34-ThrAGT | |
| chr5 | 90168332 | | 90168370 | | chr14.trna20-ThrTGT | |
| chr6 | 91653907 | | 91653948 | | chr1.trna56-ThrTGT | |
| chr6 | 137414466 | | 137414505 | | chr6.trna60-ThrAGT | |
| chr6 | 159889221 | | 159889257 | | chr14.trna20-ThrTGT | |
| chr7 | 89872115 | | 89872161 | | chr6.trna151-ThrCGT | |
| chr7 | 98522041 | | 98522079 | | chr17.trna36-ThrAGT | |
| chr7 | 100136277 | | 100136317 | | chr14.trna20-ThrTGT | |
| chr7 | 130446281 | | 130446325 | | chr17.trna36-ThrAGT | |
| chr8 | 74711616 | | 74711654 | | chr5.trna13-ThrTGT | |
| chr9 | 36284314 | | 36284352 | | chr14.trna20-ThrTGT | |
| chr9 | 106179837 | | 106179876 | | chr17.trna40-ThrAGT | |
| chr12 | 7912130 | | 7912169 | | chr14.trna20-ThrTGT | |
| chr12 | 41492775 | | 41492814 | | chr6.trna125-ThrCGT | |
| chr13 | 58448937 | | 58448975 | | chr6.trna60-ThrAGT | |
| chr15 | 49132848 | | 49132895 | | chr6.trna60-ThrAGT | |
| chr15 | 49161962 | | 49162001 | | chr17.trna36-ThrAGT | |
| chr17 | 8506310 | | 8506354 | | chr6.trna34-ThrAGT | |
| chr18 | 54065255 | | 54065293 | | chr14.trna20-ThrTGT | |
| chr18 | 73829910 | | 73829947 | | chr14.trna20-ThrTGT | |
| chrX | 50810321 | | 50810356 | | chr16.trna15-ThrCGT | |
| chrX | 96138970 | | 96139008 | | chr6.trna34-ThrAGT | |
| chrX | 99282426 | | 99282464 | | chr14.trna20-ThrTGT | |
| chr2 | 195521985 | | 195522018 | | chr17.trna12-TrpCCA | |
| chr3 | 112146841 | | 112146879 | | chr17.trna12-TrpCCA | |
| chr5 | 85130607 | | 85130643 | | chr17.trna12-TrpCCA | |
| chr6 | 46400552 | | 46400588 | | chr17.trna12-TrpCCA | |
| chr7 | 72229708 | | 72229741 | | chr17.trna6-TrpCCA | |
| chr10 | 71592249 | | 71592286 | | chr17.trna12-TrpCCA | |
| chr13 | 86475864 | | 86475900 | | chr17.trna39-TrpCCA | |
| chr1 | 180020386 | | 180020429 | | chr14.trna17-TyrGTA | |
| chr2 | 114988355 | | 114988393 | | chr8.trna12-TyrGTA | |
| chr2 | 162597790 | | 162597834 | | chr2.trna2-TyrGTA | |
| chr6 | 101965266 | | 101965322 | | chr14.trna19-TyrGTA | |
| chr6 | 152826802 | | 152826850 | | chr6.trna17-TyrGTA | |
| chr9 | 93909945 | | 93909992 | | chr8.trna4-TyrGTA | |
| chr9 | 125785214 | | 125785253 | | chr14.trna16-TyrGTA | |
| chr11 | 85436578 | | 85436630 | | chr2.trna14-TyrATA | |
| chr11 | 124570147 | | 124570193 | | chr14.trna18-TyrGTA | |
| chr12 | 53546503 | | 53546539 | | chr2.trna14-TyrATA | |
| chr17 | 43693736 | | 43693783 | | chr14.trna18-TyrGTA | |
| chr17 | 44306668 | | 44306715 | | chr14.trna18-TyrGTA | |
| chr18 | 47913650 | | 47913691 | | chr14.trna5-TyrGTA | |
| chrX | 44467490 | | 44467530 | | chr6.trna17-TyrGTA | |
| chrX | 63756584 | | 63756628 | | chr14.trna16-TyrGTA | |
| chrX | 75663764 | | 75663807 | | chr6.trna17-TyrGTA | |
| chrX | 80420322 | | 80420363 | | chr6.trna17-TyrGTA | |
| chrX | 116634116 | | 116634157 | | chr2.trna2-TyrGTA | |
| chr1 | 22012181 | | 22012217 | | chr6.trna37-ValAAC | |
| chr1 | 97018452 | | 97018485 | | chr6.trna37-ValAAC | |
| chr1 | 100339368 | | 100339403 | | chr1.trna85-ValCAC | |
| chr2 | 47507895 | | 47507932 | | chr3.trna2-ValAAC | |
| chr4 | 174883118 | | 174883153 | | chr3.trna2-ValAAC | |
| chr5 | 54871979 | | 54872016 | | chr6.trna157-ValCAC | |
| chr5 | 97065638 | | 97065672 | | chr19.trna13-ValCAC | |
| chr5 | 126787041 | | 126787076 | | chr1.trna90-ValCAC | |
| chr7 | 2624552 | | 2624588 | | chr1.trna85-ValCAC | |
| chr7 | 31010477 | | 31010524 | | chr6.trna115-ValAAC | |
| chr9 | 5759496 | | 5759535 | | chr20.trna3-ValCAC | |
| chr9 | 8214622 | | 8214653 | | chr6.trna152-ValCAC | |
| chr9 | 104328711 | | 104328743 | | chr6.trna37-ValAAC | |
| chr10 | 35841383 | | 35841422 | | chr1.trna63-ValAAC | |
| chr10 | 73798540 | | 73798576 | | chr6.trna136-ValAAC | |
| chr11 | 44784467 | | 44784503 | | chr1.trna85-ValCAC | |
| chr11 | 100765357 | | 100765395 | | chr11.trna17-ValTAC | |
| chr11 | 109283608 | | 109283642 | | chr6.trna32-ValCAC | |
| chr12 | 71892272 | | 71892312 | | chr11.trna17-ValTAC | |
| chr12 | 84683560 | | 84683596 | | chr19.trna13-ValCAC | |
| chr12 | 88403859 | | 88403895 | | chr3.trna2-ValAAC | |
| chr13 | 47817005 | | 47817041 | | chr5.trna15-ValAAC | |
| chr14 | 33032015 | | 33032046 | | chr6.trna152-ValCAC | |
| chr14 | 94883064 | | 94883100 | | chr3.trna2-ValAAC | |
| chr15 | 44218573 | | 44218605 | | chr6.trna152-ValCAC | |
| chr15 | 72419089 | | 72419125 | | chr1.trna1-ValCAC | |
| chr18 | 57641449 | | 57641486 | | chr1.trna85-ValCAC | |
| chrX | 10253784 | | 10253820 | | chr3.trna2-ValAAC | |
| chrX | 64749286 | | 64749322 | | chr1.trna1-ValCAC | |

**Table S3. 5’ truncated tailless retropseudogenes in human**

Listed are the coordinates of all human 5’ truncated and 3’ tailless retropseudogenes derived from 18S and 28S rRNAs as well as histones retrieved under relaxed conditions of the “tailless” Python-script.

| **18S rRNA** | | |
| --- | --- | --- |
| **Chr** | **Start** | **End** |
| chr5 | 10376781 | 10376831 |
| chr15 | 72588710 | 72588765 |
| **28S rRNA** | | |
| **Chr** | **Start** | **End** |
| chr1 | 1815108 | 1815204 |
| chr2 | 67566599 | 67566641 |
| chr2 | 203210989 | 203211097 |
| chr3 | 119248243 | 119248323 |
| chr3 | 134680233 | 134680280 |
| chr3 | 141772470 | 141772534 |
| chr11 | 46642426 | 46642457 |
| chr11 | 87439415 | 87439469 |
| chr17 | 38383642 | 38383708 |
| **histones** | | |
| **Chr** | **Start** | **End** |
| chr1 | 26770506 | 26770554 |
| chr1 | 52929667 | 52929720 |
| chr1 | 85379768 | 85379841 |
| chr1 | 156752510 | 156752550 |
| chr2 | 24993063 | 24993118 |
| chr2 | 30337586 | 30337868 |
| chr2 | 177893738 | 177893782 |
| chr2 | 180543848 | 180543896 |
| chr2 | 191598987 | 191599062 |
| chr2 | 235687594 | 235687630 |
| chr3 | 45050542 | 45050588 |
| chr3 | 103081314 | 103081357 |
| chr4 | 3811741 | 3811794 |
| chr4 | 121732772 | 121732888 |
| chr4 | 131503267 | 131503457 |
| chr4 | 153781420 | 153781475 |
| chr5 | 40113205 | 40113235 |
| chr5 | 111859222 | 111859267 |
| chr5 | 116097705 | 116097736 |
| chr5 | 154437921 | 154437966 |
| chr6 | 39347756 | 39347796 |
| chr6 | 52237771 | 52237819 |
| chr6 | 53565416 | 53565462 |
| chr6 | 62245177 | 62245212 |
| chr7 | 4496251 | 4496290 |
| chr7 | 115691650 | 115691716 |
| chr7 | 134447174 | 134447211 |
| chr8 | 32566046 | 32566098 |
| chr9 | 22938600 | 22938673 |
| chr9 | 122937868 | 122937900 |
| chr10 | 7453099 | 7453131 |
| chr10 | 73335079 | 73335118 |
| chr10 | 111955846 | 111955893 |
| chr10 | 119439359 | 119439438 |
| chr11 | 125648043 | 125648114 |
| chr11 | 127374254 | 127374284 |
| chr11 | 128782785 | 128782829 |
| chr11 | 130532022 | 130532052 |
| chr13 | 31226204 | 31226280 |
| chr13 | 89810610 | 89810660 |
| chr14 | 66296543 | 66296709 |
| chr15 | 46500762 | 46500795 |
| chr15 | 86091432 | 86091467 |
| chr16 | 65863384 | 65863414 |
| chr17 | 54571364 | 54571399 |
| chr18 | 35865664 | 35865700 |
| chr19 | 34185195 | 34185244 |
| chr20 | 15260415 | 15260453 |
| chr21 | 34804453 | 34804509 |
| chrX | 55075883 | 55075919 |
| chrX | 65976602 | 65976643 |
| chrX | 112219112 | 112219152 |
| chrX | 119413494 | 119413551 |

**Table S4. Summary of the GPAC pattern of tailless retropseudogenes**

All coordinates of tailless retropseudogenes (Table S3) were used in a GPAC run. The results were screened for clear presence/absence patterns (clear insertion boundaries of the element marked as +/- in the GPAC output). The amount of clear cases was summarized for each analyzed species.

p/a = presence/absence pattern; OWM = Old World monkey; NWM = New World monkey

| Species | | | Clear p/a cases |  |
| --- | --- | --- | --- | --- |
| Human | Great Apes | Anthropoidea | 19 | Hominini |
| Chimp |  |  | 0 | human+chimp |
| Gorilla |  |  | 26 | Homininae |
| Orangutan |  |  | 36 | Hominidae |
| Rhesus | OWM |  | 38 | Catarrhini |
| Marmoset | NWM |  | **258** | **Anthropoidea** |
| Tarsius |  |  | 15 | Haplorrhini |
| Bushbaby |  | Strepsirrhini | 14 | Primates |

**Table S5. Summary of the GPAC pattern of solitary ERV-LTRs**

All coordinates of full-length solitary ERV-LTR-elements were used for a GPAC run. The result was screened for clear presence/absence patterns (clear insertion boundaries of the element marked as +/- in the GPAC output). The amount of clear cases was summarized for each analyzed species.

p/a = presence/absence pattern; OWM = Old World monkey; NWM = New World monkey

| Species | | | Clear p/a cases |  |
| --- | --- | --- | --- | --- |
| Human | Great Apes | Anthropoidea | 73 | human |
| Chimp |  |  | 10 | human+chimp |
| Gorilla |  |  | 220 | Homininae |
| Orangutan |  |  | 1,362 | Hominidae |
| Rhesus | OWM |  | 443 | Catarrhini |
| Marmoset | NWM |  | **8,888** | **Anthropoidea** |
| Tarsius |  |  | 227 | Haplorrhini |
| Bushbaby |  | Strepsirrhini | 432 | Primates |
