# Supplementary material for: Ancient Traces of Tailless Retropseudogenes in Therian Genomes
Source: Genome Biol Evol. 2015 Feb 26;7(3):889–900. doi: 10.1093/gbe/evv040 (PMC5322556; doi:10.1093/gbe/evv040)
Supplement: Supplementary Data [file supp_evv040_file_S2.docx]

**Accession numbers of all histone mRNAs used for histone-derived tailless retropseudogene screens.**

NM_001002916, NM_001017990, NM_001017991, NM_001040158, NM_001164416, NM_002105, NM_002106, NM_003493, NM_003495, NM_003509, NM_003510, NM_003511, NM_003512, NM_003513, NM_003514, NM_003516, NM_003517, NM_003518, NM_003519, NM_003520, NM_003521, NM_003522, NM_003523, NM_003524, NM_003525, NM_003526, NM_003527, NM_003528, NM_003529, NM_003530, NM_003531, NM_003532, NM_003533, NM_003534, NM_003535, NM_003536, NM_003537, NM_003538, NM_003539, NM_003540, NM_003541, NM_003542, NM_003543, NM_003544, NM_003545, NM_003546, NM_003547, NM_005318, NM_005319, NM_005320, NM_005321, NM_005322, NM_005323, NM_005325, NM_006026, NM_012412, NM_018649, NM_021018, NM_021052, NM_021058, NM_021059, NM_021062, NM_021063, NM_021064, NM_021065, NM_021066, NM_021968, NM_080593, NM_080720, NM_153833, NM_170610, NM_170745, NM_175054, NM_181788

**Accession numbers of all housekeeping mRNAs used for housekeeping gene-derived tailless retropseudogene screens.**

NM_000020, NM_000034, NM_000100, NM_000175, NM_000182, NM_000183, NM_000199, NM_000214, NM_000239, NM_000269, NM_000291, NM_000356, NM_000367, NM_000398, NM_000405, NM_000431, NM_000454, NM_000516, NM_000529, NM_000754, NM_000757, NM_000801, NM_000802, NM_000841, NM_000858, NM_000884, NM_000918, NM_000937, NM_000967, NM_000969, NM_000973, NM_000975, NM_000977, NM_000979, NM_000981, NM_000985, NM_000988, NM_000992, NM_000994, NM_000995, NM_000997, NM_000999, NM_001001, NM_001003, NM_001004, NM_001009, NM_001013, NM_001014, NM_001015, NM_001016, NM_001017, NM_001018, NM_001020, NM_001022, NM_001026, NM_001028, NM_001053, NM_001064, NM_001069, NM_001101, NM_001111, NM_001119, NM_001120, NM_001127, NM_001130, NM_001155, NM_001183, NM_001207, NM_001247, NM_001250, NM_001273, NM_001281, NM_001296, NM_001320, NM_001328, NM_001344, NM_001350, NM_001355, NM_001360, NM_001402, NM_001418, NM_001420, NM_001428, NM_001436, NM_001440, NM_001469, NM_001487, NM_001493, NM_001494, NM_001502, NM_001536, NM_001537, NM_001614, NM_001619, NM_001623, NM_001642, NM_001654, NM_001658, NM_001659, NM_001660, NM_001662, NM_001664, NM_001667, NM_001675, NM_001687, NM_001689, NM_001694, NM_001696, NM_001697, NM_001728, NM_001743, NM_001746, NM_001749, NM_001760, NM_001785, NM_001788, NM_001810, NM_001823, NM_001833, NM_001834, NM_001843, NM_001848, NM_001861, NM_001862, NM_001863, NM_001865, NM_001867, NM_001894, NM_001904, NM_001909, NM_001914, NM_001916, NM_001950, NM_001958, NM_001961, NM_001967, NM_001997, NM_002002, NM_002032, NM_002046, NM_002048, NM_002070, NM_002080, NM_002085, NM_002088, NM_002107, NM_002117, NM_002127, NM_002128, NM_002138, NM_002140, NM_002149, NM_002167, NM_002227, NM_002300, NM_002308, NM_002315, NM_002355, NM_002375, NM_002383, NM_002406, NM_002415, NM_002419, NM_002434, NM_002444, NM_002455, NM_002467, NM_002473, NM_002488, NM_002494, NM_002512, NM_002539, NM_002568, NM_002574, NM_002622, NM_002624, NM_002631, NM_002635, NM_002636, NM_002648, NM_002654, NM_002686, NM_002714, NM_002733, NM_002743, NM_002792, NM_002793, NM_002794, NM_002796, NM_002799, NM_002808, NM_002809, NM_002812, NM_002815, NM_002818, NM_002819, NM_002823, NM_002931, NM_002939, NM_002946, NM_002948, NM_002950, NM_002952, NM_002954, NM_002967, NM_003016, NM_003021, NM_003089, NM_003091, NM_003096, NM_003131, NM_003132, NM_003134, NM_003145, NM_003186, NM_003190, NM_003217, NM_003313, NM_003314, NM_003321, NM_003329, NM_003334, NM_003339, NM_003345, NM_003365, NM_003377, NM_003379, NM_003404, NM_003405, NM_003406, NM_003430, NM_003465, NM_003466, NM_003475, NM_003526, NM_003562, NM_003573, NM_003576, NM_003641, NM_003680, NM_003746, NM_003752, NM_003753, NM_003754, NM_003755, NM_003757, NM_003766, NM_003769, NM_003779, NM_003795, NM_003801, NM_003815, NM_003860, NM_003899, NM_003906, NM_003910, NM_003915, NM_003952, NM_003969, NM_003973, NM_004039, NM_004046, NM_004047, NM_004048, NM_004068, NM_004069, NM_004074, NM_004121, NM_004146, NM_004161, NM_004168, NM_004176, NM_004197, NM_004231, NM_004255, NM_004285, NM_004308, NM_004309, NM_004339, NM_004356, NM_004373, NM_004383, NM_004394, NM_004404, NM_004436, NM_004450, NM_004494, NM_004499, NM_004515, NM_004517, NM_004541, NM_004552, NM_004584, NM_004587, NM_004596, NM_004597, NM_004640, NM_004643, NM_004649, NM_004651, NM_004689, NM_004710, NM_004712, NM_004718, NM_004729, NM_004759, NM_004781, NM_004785, NM_004888, NM_004889, NM_004890, NM_004893, NM_004894, NM_004898, NM_004907, NM_004910, NM_004924, NM_004926, NM_004930, NM_004952, NM_004960, NM_005001, NM_005022, NM_005053, NM_005080, NM_005088, NM_005103, NM_005105, NM_005112, NM_005157, NM_005165, NM_005175, NM_005180, NM_005216, NM_005219, NM_005273, NM_005340, NM_005347, NM_005354, NM_005370, NM_005381, NM_005418, NM_005439, NM_005456, NM_005494, NM_005507, NM_005520, NM_005545, NM_005548, NM_005550, NM_005561, NM_005566, NM_005617, NM_005629, NM_005682, NM_005698, NM_005718, NM_005719, NM_005726, NM_005731, NM_005745, NM_005762, NM_005786, NM_005787, NM_005801, NM_005839, NM_005870, NM_005884, NM_005891, NM_005917, NM_005954, NM_005984, NM_005997, NM_005998, NM_006003, NM_006004, NM_006010, NM_006013, NM_006032, NM_006039, NM_006066, NM_006082, NM_006086, NM_006098, NM_006118, NM_006145, NM_006148, NM_006156, NM_006221, NM_006262, NM_006268, NM_006283, NM_006289, NM_006295, NM_006325, NM_006333, NM_006349, NM_006351, NM_006354, NM_006356, NM_006362, NM_006368, NM_006372, NM_006389, NM_006401, NM_006411, NM_006423, NM_006429, NM_006442, NM_006445, NM_006460, NM_006510, NM_006513, NM_006570, NM_006595, NM_006597, NM_006612, NM_006623, NM_006659, NM_006666, NM_006688, NM_006694, NM_006703, NM_006711, NM_006743, NM_006755, NM_006782, NM_006796, NM_006801, NM_006808, NM_006815, NM_006817, NM_006826, NM_006830, NM_006833, NM_006867, NM_006888, NM_006899, NM_006908, NM_006936, NM_006937, NM_007008, NM_007067, NM_007100, NM_007103, NM_007104, NM_007108, NM_007144, NM_007182, NM_007209, NM_007245, NM_007260, NM_007262, NM_007263, NM_007278, NM_007285, NM_007286, NM_007355, NM_007359, NM_007363, NM_012099, NM_012100, NM_012102, NM_012106, NM_012111, NM_012127, NM_012138, NM_012179, NM_012227, NM_012401, NM_012407, NM_012412, NM_012423, NM_013232, NM_013234, NM_013310, NM_013318, NM_014225, NM_014228, NM_014231, NM_014255, NM_014281, NM_014302, NM_014390, NM_014402, NM_014420, NM_014453, NM_014508, NM_014604, NM_014623, NM_014630, NM_014654, NM_014694, NM_014696, NM_014730, NM_014754, NM_014761, NM_014764, NM_014774, NM_014874, NM_014891, NM_014901, NM_014916, NM_014944, NM_014972, NM_015024, NM_015292, NM_015318, NM_015343, NM_015399, NM_015456, NM_015646, NM_015680, NM_016237, NM_016292, NM_016457, NM_016532, NM_017432, NM_017510, NM_017670, NM_017797, NM_017828, NM_018955, NM_018975, NM_019059, NM_019884, NM_020150, NM_020151, NM_020195, NM_020360, NM_020529, NM_021009, NM_021019, NM_021074, NM_021103, NM_021107, NM_021128, NM_021134, NM_021642, NM_021953, NM_021959, NM_021960, NM_021974, NM_021975, NM_021983, NM_022551, NM_022830, NM_023009, NM_024011, NM_024069, NM_024092, NM_024112, NM_024798, NM_030662, NM_030796, NM_031420, NM_032348, NM_032378, NM_032635, NM_032801, NM_033142, NM_033546, NM_133476, NM_144565, NM_144582

**Accession numbers of all mono-exonic mRNAs used mono-exonic gene-derived tailless retropseudogene screens.**

NM_001001656, NM_001001657, NM_001001658, NM_001001659, NM_001001667, NM_001001674, NM_001001786, NM_001001802, NM_001001821, NM_001001824, NM_001001827, NM_001001912, NM_001001913, NM_001001914, NM_001001915, NM_001001916, NM_001001918, NM_001001919, NM_001001921, NM_001001923, NM_001001952, NM_001001953, NM_001001954, NM_001001956, NM_001001957, NM_001001958, NM_001001959, NM_001001960, NM_001001963, NM_001001964, NM_001001965, NM_001001966, NM_001001967, NM_001001968, NM_001002255, NM_001002907, NM_001002917, NM_001002918, NM_001002925, NM_001003443, NM_001003745, NM_001003750, NM_001004052, NM_001004058, NM_001004059, NM_001004064, NM_001004124, NM_001004134, NM_001004135, NM_001004136, NM_001004137, NM_001004195, NM_001004450, NM_001004451, NM_001004452, NM_001004453, NM_001004454, NM_001004456, NM_001004457, NM_001004458, NM_001004459, NM_001004460, NM_001004461, NM_001004462, NM_001004463, NM_001004464, NM_001004465, NM_001004466, NM_001004467, NM_001004469, NM_001004473, NM_001004474, NM_001004475, NM_001004476, NM_001004477, NM_001004478, NM_001004479, NM_001004480, NM_001004481, NM_001004482, NM_001004483, NM_001004485, NM_001004486, NM_001004487, NM_001004488, NM_001004489, NM_001004490, NM_001004492, NM_001004684, NM_001004685, NM_001004687, NM_001004689, NM_001004690, NM_001004691, NM_001004692, NM_001004693, NM_001004694, NM_001004695, NM_001004697, NM_001004699, NM_001004701, NM_001004702, NM_001004703, NM_001004704, NM_001004705, NM_001004706, NM_001004707, NM_001004708, NM_001004711, NM_001004712, NM_001004714, NM_001004717, NM_001004723, NM_001004724, NM_001004725, NM_001004726, NM_001004727, NM_001004728, NM_001004729, NM_001004730, NM_001004734, NM_001004735, NM_001004736, NM_001004737, NM_001004738, NM_001004739, NM_001004740, NM_001004741, NM_001004743, NM_001004744, NM_001004745, NM_001004748, NM_001004749, NM_001004750, NM_001004752, NM_001004755, NM_001004758, NM_001004760, NM_001005160, NM_001005164, NM_001005165, NM_001005167, NM_001005168, NM_001005169, NM_001005174, NM_001005177, NM_001005178, NM_001005181, NM_001005182, NM_001005183, NM_001005185, NM_001005186, NM_001005187, NM_001005188, NM_001005189, NM_001005190, NM_001005192, NM_001005194, NM_001005195, NM_001005196, NM_001005197, NM_001005199, NM_001005200, NM_001005201, NM_001005202, NM_001005204, NM_001005211, NM_001005213, NM_001005216, NM_001005218, NM_001005221, NM_001005224, NM_001005234, NM_001005235, NM_001005236, NM_001005237, NM_001005238, NM_001005239, NM_001005240, NM_001005245, NM_001005274, NM_001005276, NM_001005277, NM_001005278, NM_001005279, NM_001005280, NM_001005281, NM_001005282, NM_001005284, NM_001005285, NM_001005286, NM_001005287, NM_001005289, NM_001005323, NM_001005324, NM_001005325, NM_001005326, NM_001005327, NM_001005328, NM_001005329, NM_001005338, NM_001005464, NM_001005465, NM_001005466, NM_001005467, NM_001005468, NM_001005469, NM_001005470, NM_001005471, NM_001005479, NM_001005480, NM_001005482, NM_001005483, NM_001005484, NM_001005487, NM_001005489, NM_001005490, NM_001005491, NM_001005492, NM_001005493, NM_001005494, NM_001005495, NM_001005496, NM_001005497, NM_001005499, NM_001005500, NM_001005501, NM_001005504, NM_001005512, NM_001005514, NM_001005515, NM_001005516, NM_001005517, NM_001005518, NM_001005519, NM_001005522, NM_001005853, NM_001005922, NM_001007249, NM_001007532, NM_001008739, NM_001012416, NM_001012503, NM_001012708, NM_001012975, NM_001013354, NM_001013355, NM_001013358, NM_001017990, NM_001017991, NM_001024599, NM_001024679, NM_001024822, NM_001029886, NM_001034077, NM_001039792, NM_001040071, NM_001040874, NM_001077711, NM_001083308, NM_001089591, NM_001097643, NM_001099219, NM_001101389, NM_001105569, NM_001105581, NM_001123068, NM_001123375, NM_001123387, NM_001128077, NM_001128598, NM_001135789, NM_001143883, NM_001144032, NM_001146033, NM_001160325, NM_001164261, NM_001164262, NM_001164377, NM_001164405, NM_001164434, NM_001164435, NM_001165252, NM_001165877, NM_001185149, NM_001190460, NM_001195081, NM_001195135, NM_001195522, NM_001214, NM_001257305, NM_001258283, NM_001258284, NM_001258285, NM_001271682, NM_002169, NM_002171, NM_002172, NM_002173, NM_002176, NM_002548, NM_002550, NM_003140, NM_003493, NM_003495, NM_003509, NM_003510, NM_003511, NM_003512, NM_003513, NM_003514, NM_003516, NM_003517, NM_003518, NM_003519, NM_003520, NM_003521, NM_003522, NM_003523, NM_003524, NM_003525, NM_003526, NM_003527, NM_003529, NM_003531, NM_003532, NM_003533, NM_003534, NM_003535, NM_003536, NM_003537, NM_003538, NM_003539, NM_003540, NM_003541, NM_003542, NM_003543, NM_003544, NM_003545, NM_003546, NM_003547, NM_003548, NM_003553, NM_003554, NM_003555, NM_003697, NM_003700, NM_004122, NM_005285, NM_005299, NM_005303, NM_005319, NM_005320, NM_005321, NM_005322, NM_005323, NM_005325, NM_005421, NM_006189, NM_006308, NM_006607, NM_006637, NM_006900, NM_012131, NM_012274, NM_012352, NM_012353, NM_012360, NM_012363, NM_012364, NM_012365, NM_012367, NM_012373, NM_012374, NM_012375, NM_012377, NM_012378, NM_012403, NM_012404, NM_013938, NM_014380, NM_014565, NM_014566, NM_014626, NM_016347, NM_016944, NM_016945, NM_017422, NM_017504, NM_018111, NM_018973, NM_021002, NM_021018, NM_021052, NM_021057, NM_021058, NM_021059, NM_021062, NM_021063, NM_021064, NM_021065, NM_021066, NM_021068, NM_021247, NM_021268, NM_021968, NM_022076, NM_022375, NM_023918, NM_023921, NM_023922, NM_024013, NM_030901, NM_030903, NM_030908, NM_030946, NM_030966, NM_030967, NM_030975, NM_031287, NM_031894, NM_031958, NM_031959, NM_031963, NM_031964, NM_033032, NM_033061, NM_033062, NM_033179, NM_033184, NM_033185, NM_033187, NM_033188, NM_033191, NM_033194, NM_033445, NM_054104, NM_054105, NM_054106, NM_054107, NM_080596, NM_080720, NM_080746, NM_080859, NM_130844, NM_133498, NM_138331, NM_145208, NM_152898, NM_153444, NM_153445, NM_170610, NM_170745, NM_172194, NM_173351, NM_173857, NM_175054, NM_175055, NM_175065, NM_175857, NM_175858, NM_176791, NM_176882, NM_176883, NM_176887, NM_176888, NM_176890, NM_177437, NM_177455, NM_177478, NM_178168, NM_178230, NM_178348, NM_178354, NM_178356, NM_178431, NM_178433, NM_178434, NM_178483, NM_178511, NM_181429, NM_181599, NM_181600, NM_181602, NM_181604, NM_181605, NM_181607, NM_181608, NM_181609, NM_181610, NM_181611, NM_181612, NM_181614, NM_181615, NM_181616, NM_181617, NM_181619, NM_181620, NM_181621, NM_181622, NM_181623, NM_181624, NM_181684, NM_181686, NM_181791, NM_198180, NM_198694, NM_198695, NM_198696, NM_198697, NM_198698, NM_198699, NM_198923, NM_198944, NM_199290, NM_203303, NM_205859, NM_206880, NM_206899, NM_207186
