# Supplementary material for: Ancient Traces of Tailless Retropseudogenes in Therian Genomes
Source: Genome Biol Evol. 2015 Feb 26;7(3):889–900. doi: 10.1093/gbe/evv040 (PMC5322556; doi:10.1093/gbe/evv040)
Supplement: Supplementary Data [file supp_evv040_Table_S6.docx]

**Table S6. Tailless retropseudogenes in non-human mammals**

Listed are the coordinates of 5S rRNA- and U2-derived tailless retropseudogenes in all non-human mammalian genomes.

| **5S rRNA** | | | |
| --- | --- | --- | --- |
| **Species** | **Chr** | **Start** | **End** |
| ***Bos taurus*** | chr01 | 10181339 | 10181379 |
|  | chr01 | 17827715 | 17827803 |
|  | chr01 | 18424225 | 18424259 |
|  | chr01 | 36019797 | 36019847 |
|  | chr01 | 42994212 | 42994259 |
|  | chr01 | 53941769 | 53941857 |
|  | chr01 | 65954263 | 65954304 |
|  | chr01 | 68426382 | 68426472 |
|  | chr01 | 71150777 | 71150872 |
|  | chr01 | 73498580 | 73498612 |
|  | chr01 | 88544194 | 88544235 |
|  | chr01 | 90393049 | 90393138 |
|  | chr01 | 93356880 | 93356970 |
|  | chr01 | 93663021 | 93663060 |
|  | chr01 | 124790931 | 124791010 |
|  | chr01 | 138063521 | 138063560 |
|  | chr01 | 146301561 | 146301600 |
|  | chr02 | 8880321 | 8880362 |
|  | chr02 | 15040044 | 15040085 |
|  | chr02 | 17970417 | 17970454 |
|  | chr02 | 27437849 | 27437934 |
|  | chr02 | 34923410 | 34923497 |
|  | chr02 | 35278739 | 35278827 |
|  | chr02 | 36337760 | 36337801 |
|  | chr02 | 70436501 | 70436551 |
|  | chr02 | 76957809 | 76957849 |
|  | chr02 | 81143111 | 81143152 |
|  | chr02 | 85261787 | 85261828 |
|  | chr02 | 85814101 | 85814142 |
|  | chr02 | 87546891 | 87546967 |
|  | chr02 | 102191332 | 102191368 |
|  | chr02 | 107097397 | 107097439 |
|  | chr02 | 118975657 | 118975746 |
|  | chr02 | 128694420 | 128694461 |
|  | chr02 | 130421231 | 130421272 |
|  | chr02 | 136045213 | 136045267 |
|  | chr03 | 12215617 | 12215658 |
|  | chr03 | 12367500 | 12367541 |
|  | chr03 | 15224299 | 15224339 |
|  | chr03 | 40919599 | 40919651 |
|  | chr03 | 43515581 | 43515670 |
|  | chr03 | 51571678 | 51571765 |
|  | chr03 | 63084089 | 63084130 |
|  | chr03 | 80769394 | 80769481 |
|  | chr03 | 83838485 | 83838576 |
|  | chr03 | 99385488 | 99385586 |
|  | chr03 | 99742715 | 99742803 |
|  | chr03 | 100692392 | 100692481 |
|  | chr03 | 106742430 | 106742479 |
|  | chr04 | 19249432 | 19249473 |
|  | chr04 | 19829293 | 19829339 |
|  | chr04 | 27893004 | 27893054 |
|  | chr04 | 29265114 | 29265203 |
|  | chr04 | 40887346 | 40887433 |
|  | chr04 | 43204088 | 43204135 |
|  | chr04 | 51630477 | 51630565 |
|  | chr04 | 65990254 | 65990292 |
|  | chr04 | 71909360 | 71909401 |
|  | chr04 | 77094904 | 77094945 |
|  | chr04 | 86517566 | 86517606 |
|  | chr04 | 87159817 | 87159859 |
|  | chr04 | 89006581 | 89006669 |
|  | chr04 | 89159312 | 89159400 |
|  | chr04 | 92574689 | 92574730 |
|  | chr04 | 113107110 | 113107200 |
|  | chr04 | 113362110 | 113362155 |
|  | chr04 | 113544682 | 113544727 |
|  | chr05 | 1419350 | 1419392 |
|  | chr05 | 15624631 | 15624675 |
|  | chr05 | 15694046 | 15694135 |
|  | chr05 | 29170300 | 29170388 |
|  | chr05 | 31298319 | 31298407 |
|  | chr05 | 32418969 | 32419010 |
|  | chr05 | 44255549 | 44255634 |
|  | chr05 | 49450309 | 49450397 |
|  | chr05 | 49746226 | 49746266 |
|  | chr05 | 65529890 | 65529978 |
|  | chr05 | 77966473 | 77966559 |
|  | chr05 | 83571785 | 83571864 |
|  | chr05 | 85914728 | 85914817 |
|  | chr05 | 102480670 | 102480707 |
|  | chr05 | 102623986 | 102624023 |
|  | chr05 | 107676275 | 107676316 |
|  | chr05 | 108007792 | 108007881 |
|  | chr05 | 111866543 | 111866634 |
|  | chr06 | 4903634 | 4903723 |
|  | chr06 | 15754978 | 15755019 |
|  | chr06 | 19374836 | 19374876 |
|  | chr06 | 24463189 | 24463244 |
|  | chr06 | 32350000 | 32350041 |
|  | chr06 | 32584143 | 32584185 |
|  | chr06 | 32774248 | 32774288 |
|  | chr06 | 36201477 | 36201518 |
|  | chr06 | 45845714 | 45845802 |
|  | chr06 | 46501972 | 46502061 |
|  | chr06 | 57102227 | 57102276 |
|  | chr06 | 62337201 | 62337242 |
|  | chr06 | 62581505 | 62581594 |
|  | chr06 | 68966744 | 68966834 |
|  | chr06 | 72020146 | 72020235 |
|  | chr06 | 99860099 | 99860140 |
|  | chr06 | 103155346 | 103155385 |
|  | chr06 | 106113779 | 106113867 |
|  | chr06 | 110967669 | 110967718 |
|  | chr06 | 111006497 | 111006540 |
|  | chr06 | 114414466 | 114414555 |
|  | chr07 | 5401386 | 5401428 |
|  | chr07 | 16373063 | 16373113 |
|  | chr07 | 21019702 | 21019742 |
|  | chr07 | 23852870 | 23852967 |
|  | chr07 | 27257925 | 27258023 |
|  | chr07 | 33278651 | 33278740 |
|  | chr07 | 38692505 | 38692594 |
|  | chr07 | 40166158 | 40166247 |
|  | chr07 | 46231367 | 46231408 |
|  | chr07 | 53091004 | 53091042 |
|  | chr07 | 63703333 | 63703421 |
|  | chr07 | 67309965 | 67310000 |
|  | chr07 | 68693467 | 68693555 |
|  | chr07 | 83353879 | 83353915 |
|  | chr07 | 103066824 | 103066866 |
|  | chr08 | 18910428 | 18910517 |
|  | chr08 | 21197122 | 21197212 |
|  | chr08 | 24128841 | 24128930 |
|  | chr08 | 29713721 | 29713810 |
|  | chr08 | 40407316 | 40407357 |
|  | chr08 | 45491853 | 45491908 |
|  | chr08 | 51610515 | 51610555 |
|  | chr08 | 54628104 | 54628144 |
|  | chr08 | 64096233 | 64096322 |
|  | chr08 | 73225642 | 73225731 |
|  | chr08 | 93576897 | 93576941 |
|  | chr08 | 107024980 | 107025034 |
|  | chr09 | 68677461 | 68677549 |
|  | chr09 | 70106221 | 70106300 |
|  | chr09 | 73759428 | 73759469 |
|  | chr09 | 74586391 | 74586481 |
|  | chr09 | 82721474 | 82721515 |
|  | chr09 | 86836822 | 86836911 |
|  | chr09 | 89045272 | 89045312 |
|  | chr09 | 92428962 | 92429050 |
|  | chr09 | 94976441 | 94976530 |
|  | chr09 | 97377300 | 97377341 |
|  | chr09 | 100448476 | 100448565 |
|  | chr10 | 606007 | 606061 |
|  | chr10 | 9218628 | 9218717 |
|  | chr10 | 15115568 | 15115656 |
|  | chr10 | 15324932 | 15324982 |
|  | chr10 | 18501851 | 18501895 |
|  | chr10 | 34366011 | 34366050 |
|  | chr10 | 51885669 | 51885757 |
|  | chr10 | 52766699 | 52766739 |
|  | chr10 | 55273776 | 55273864 |
|  | chr10 | 64188247 | 64188336 |
|  | chr10 | 65798496 | 65798585 |
|  | chr10 | 68185346 | 68185436 |
|  | chr10 | 77300497 | 77300586 |
|  | chr11 | 5938055 | 5938144 |
|  | chr11 | 18182965 | 18183006 |
|  | chr11 | 20879550 | 20879588 |
|  | chr11 | 23132719 | 23132810 |
|  | chr11 | 38589452 | 38589493 |
|  | chr11 | 52875146 | 52875186 |
|  | chr11 | 55568729 | 55568770 |
|  | chr11 | 56977692 | 56977781 |
|  | chr11 | 72950195 | 72950236 |
|  | chr11 | 84969484 | 84969573 |
|  | chr11 | 86381231 | 86381320 |
|  | chr12 | 33142212 | 33142262 |
|  | chr12 | 40812746 | 40812787 |
|  | chr12 | 67660415 | 67660506 |
|  | chr12 | 69593009 | 69593095 |
|  | chr12 | 78528089 | 78528133 |
|  | chr13 | 47573474 | 47573565 |
|  | chr13 | 48316814 | 48316901 |
|  | chr13 | 51542324 | 51542366 |
|  | chr13 | 52270797 | 52270836 |
|  | chr13 | 52992748 | 52992793 |
|  | chr13 | 55255233 | 55255268 |
|  | chr13 | 61279934 | 61279974 |
|  | chr13 | 63144344 | 63144386 |
|  | chr13 | 65689874 | 65689961 |
|  | chr13 | 76349412 | 76349453 |
|  | chr14 | 8284523 | 8284611 |
|  | chr14 | 11759889 | 11759979 |
|  | chr14 | 11802958 | 11802999 |
|  | chr14 | 15494259 | 15494309 |
|  | chr14 | 16832034 | 16832122 |
|  | chr14 | 52780650 | 52780730 |
|  | chr14 | 54224435 | 54224475 |
|  | chr14 | 56700315 | 56700356 |
|  | chr14 | 56717006 | 56717094 |
|  | chr14 | 65342378 | 65342417 |
|  | chr14 | 76808570 | 76808614 |
|  | chr15 | 17113799 | 17113840 |
|  | chr15 | 22839655 | 22839696 |
|  | chr15 | 25187744 | 25187784 |
|  | chr15 | 38707975 | 38708016 |
|  | chr15 | 41449187 | 41449237 |
|  | chr15 | 57287214 | 57287302 |
|  | chr16 | 16739109 | 16739198 |
|  | chr16 | 19094224 | 19094311 |
|  | chr16 | 31142384 | 31142474 |
|  | chr16 | 40692503 | 40692591 |
|  | chr16 | 53755266 | 53755355 |
|  | chr16 | 62222527 | 62222618 |
|  | chr16 | 65966789 | 65966879 |
|  | chr16 | 66902011 | 66902096 |
|  | chr16 | 69084293 | 69084331 |
|  | chr16 | 76163658 | 76163705 |
|  | chr16 | 81445702 | 81445743 |
|  | chr17 | 1257134 | 1257213 |
|  | chr17 | 10760804 | 10760845 |
|  | chr17 | 14714208 | 14714258 |
|  | chr17 | 55250876 | 55250949 |
|  | chr17 | 59299488 | 59299577 |
|  | chr17 | 60155819 | 60155860 |
|  | chr17 | 62089472 | 62089522 |
|  | chr17 | 64583147 | 64583184 |
|  | chr18 | 8443138 | 8443227 |
|  | chr18 | 8986694 | 8986735 |
|  | chr18 | 20707597 | 20707685 |
|  | chr18 | 21207311 | 21207360 |
|  | chr18 | 34487100 | 34487141 |
|  | chr18 | 48185675 | 48185764 |
|  | chr18 | 49919677 | 49919767 |
|  | chr18 | 58210520 | 58210607 |
|  | chr18 | 58436585 | 58436673 |
|  | chr18 | 60275895 | 60275936 |
|  | chr19 | 4149693 | 4149734 |
|  | chr19 | 6705142 | 6705184 |
|  | chr19 | 23780436 | 23780477 |
|  | chr19 | 24116424 | 24116465 |
|  | chr19 | 36129943 | 36129976 |
|  | chr19 | 48703871 | 48703955 |
|  | chr19 | 52872966 | 52873010 |
|  | chr19 | 56136254 | 56136295 |
|  | chr20 | 19841014 | 19841103 |
|  | chr20 | 20653650 | 20653736 |
|  | chr20 | 23346730 | 23346770 |
|  | chr20 | 56882722 | 56882771 |
|  | chr20 | 70834692 | 70834779 |
|  | chr21 | 40604219 | 40604308 |
|  | chr21 | 42557966 | 42558055 |
|  | chr21 | 46911091 | 46911135 |
|  | chr21 | 59166092 | 59166165 |
|  | chr21 | 59766775 | 59766811 |
|  | chr21 | 63370491 | 63370528 |
|  | chr21 | 69505319 | 69505361 |
|  | chr22 | 11262135 | 11262176 |
|  | chr22 | 20841427 | 20841516 |
|  | chr22 | 29829019 | 29829108 |
|  | chr22 | 43238072 | 43238117 |
|  | chr22 | 49324441 | 49324484 |
|  | chr22 | 52249303 | 52249391 |
|  | chr22 | 53210285 | 53210326 |
|  | chr22 | 57185927 | 57185963 |
|  | chr22 | 57293533 | 57293574 |
|  | chr23 | 7979444 | 7979493 |
|  | chr23 | 9296903 | 9296944 |
|  | chr23 | 30280440 | 30280530 |
|  | chr23 | 38778770 | 38778858 |
|  | chr23 | 43286677 | 43286731 |
|  | chr23 | 44035253 | 44035349 |
|  | chr23 | 47337650 | 47337739 |
|  | chr23 | 51686170 | 51686257 |
|  | chr24 | 11009207 | 11009296 |
|  | chr24 | 11711409 | 11711499 |
|  | chr24 | 12444656 | 12444695 |
|  | chr24 | 28204747 | 28204788 |
|  | chr24 | 43002879 | 43002968 |
|  | chr24 | 45367631 | 45367674 |
|  | chr24 | 46051052 | 46051141 |
|  | chr24 | 46069853 | 46069895 |
|  | chr24 | 47594460 | 47594502 |
|  | chr24 | 62597866 | 62597946 |
|  | chr25 | 7564683 | 7564770 |
|  | chr25 | 9797431 | 9797463 |
|  | chr25 | 11592300 | 11592341 |
|  | chr25 | 17495484 | 17495525 |
|  | chr25 | 18971364 | 18971405 |
|  | chr25 | 29661792 | 29661833 |
|  | chr25 | 31466294 | 31466383 |
|  | chr25 | 37539908 | 37539958 |
|  | chr26 | 834242 | 834280 |
|  | chr26 | 20966142 | 20966181 |
|  | chr26 | 22308107 | 22308146 |
|  | chr26 | 25366088 | 25366177 |
|  | chr26 | 33068745 | 33068794 |
|  | chr26 | 35343645 | 35343693 |
|  | chr27 | 40026276 | 40026379 |
|  | chr28 | 8421609 | 8421650 |
|  | chr28 | 9665773 | 9665869 |
|  | chr28 | 10826202 | 10826301 |
|  | chr28 | 23217770 | 23217807 |
|  | chr28 | 29491229 | 29491305 |
|  | chr29 | 17828478 | 17828567 |
|  | chr29 | 29690424 | 29690512 |
|  | chr29 | 31477067 | 31477107 |
|  | chr29 | 32487691 | 32487735 |
|  | chr29 | 43344974 | 43345061 |
|  | chr29 | 47818362 | 47818413 |
|  | chr29 | 50787228 | 50787314 |
|  | chrX | 17086896 | 17086934 |
|  | chrX | 22758273 | 22758310 |
|  | chrX | 24639693 | 24639733 |
|  | chrX | 25131324 | 25131375 |
|  | chrX | 81099030 | 81099118 |
|  | chrX | 91141062 | 91141141 |
|  | chrX | 93144875 | 93144964 |
|  | chrX | 93426859 | 93426900 |
|  | chrX | 103516537 | 103516586 |
|  | chrX | 126788000 | 126788050 |
|  | chrX | 140555643 | 140555682 |
|  | chrX | 142878363 | 142878450 |
|  | chrX | 148538948 | 148539037 |
| ***Canis lupus familiaris*** | chr01 | 7120004 | 7120043 |
|  | chr01 | 9186728 | 9186769 |
|  | chr01 | 12568024 | 12568116 |
|  | chr01 | 12905418 | 12905456 |
|  | chr01 | 24255784 | 24255873 |
|  | chr01 | 24887660 | 24887697 |
|  | chr01 | 25398470 | 25398511 |
|  | chr01 | 32507007 | 32507048 |
|  | chr01 | 33512126 | 33512167 |
|  | chr01 | 57178598 | 57178638 |
|  | chr01 | 58048101 | 58048142 |
|  | chr01 | 59277253 | 59277295 |
|  | chr01 | 73484050 | 73484091 |
|  | chr01 | 75858764 | 75858853 |
|  | chr01 | 81902867 | 81902908 |
|  | chr01 | 89344692 | 89344733 |
|  | chr01 | 93165666 | 93165759 |
|  | chr01 | 94942337 | 94942427 |
|  | chr01 | 112922026 | 112922114 |
|  | chr01 | 114453292 | 114453382 |
|  | chr01 | 119546234 | 119546325 |
|  | chr01 | 122537854 | 122537895 |
|  | chr02 | 10036439 | 10036530 |
|  | chr02 | 12959077 | 12959169 |
|  | chr02 | 14320737 | 14320826 |
|  | chr02 | 22456796 | 22456837 |
|  | chr02 | 25529796 | 25529837 |
|  | chr02 | 32885709 | 32885750 |
|  | chr02 | 34683140 | 34683184 |
|  | chr02 | 36144572 | 36144612 |
|  | chr02 | 41883794 | 41883835 |
|  | chr02 | 51376042 | 51376083 |
|  | chr02 | 54282666 | 54282707 |
|  | chr02 | 58192490 | 58192536 |
|  | chr02 | 67817745 | 67817832 |
|  | chr02 | 69157423 | 69157464 |
|  | chr02 | 70600792 | 70600833 |
|  | chr03 | 12168954 | 12168995 |
|  | chr03 | 17295293 | 17295382 |
|  | chr03 | 19333164 | 19333205 |
|  | chr03 | 22987423 | 22987459 |
|  | chr03 | 31032756 | 31032797 |
|  | chr03 | 32014391 | 32014431 |
|  | chr03 | 42123795 | 42123831 |
|  | chr03 | 42317205 | 42317295 |
|  | chr03 | 46315231 | 46315270 |
|  | chr03 | 49010416 | 49010458 |
|  | chr03 | 53166158 | 53166204 |
|  | chr03 | 59282218 | 59282258 |
|  | chr03 | 61958679 | 61958767 |
|  | chr03 | 82191883 | 82191924 |
|  | chr03 | 82904032 | 82904121 |
|  | chr03 | 87245335 | 87245385 |
|  | chr04 | 6802367 | 6802454 |
|  | chr04 | 12814821 | 12814910 |
|  | chr04 | 13229572 | 13229612 |
|  | chr04 | 13578983 | 13579071 |
|  | chr04 | 19908772 | 19908861 |
|  | chr04 | 20883525 | 20883605 |
|  | chr05 | 824997 | 825044 |
|  | chr06 | 9541276 | 9541316 |
|  | chr06 | 10458341 | 10458390 |
|  | chr06 | 15378964 | 15379001 |
|  | chr06 | 17675821 | 17675911 |
|  | chr07 | 3806197 | 3806238 |
|  | chr08 | 3539644 | 3539685 |
|  | chr08 | 10485021 | 10485061 |
|  | chr08 | 11585770 | 11585805 |
|  | chr08 | 13900262 | 13900312 |
|  | chr08 | 16599275 | 16599316 |
|  | chr08 | 18369987 | 18370036 |
|  | chr08 | 41218351 | 41218392 |
|  | chr08 | 46241266 | 46241359 |
|  | chr09 | 10287374 | 10287463 |
|  | chr09 | 18000356 | 18000445 |
|  | chr09 | 18023869 | 18023958 |
|  | chr09 | 18854179 | 18854220 |
|  | chr09 | 22365481 | 22365519 |
|  | chr10 | 4283935 | 4283975 |
|  | chr10 | 9144977 | 9145018 |
|  | chr10 | 16084971 | 16085012 |
|  | chr10 | 29948274 | 29948319 |
|  | chr10 | 33286345 | 33286398 |
|  | chr10 | 60253714 | 60253751 |
|  | chr10 | 67602912 | 67602957 |
|  | chr11 | 1684926 | 1685015 |
|  | chr11 | 16949883 | 16949929 |
|  | chr11 | 31262816 | 31262856 |
|  | chr11 | 42531893 | 42531983 |
|  | chr11 | 50929817 | 50929904 |
|  | chr11 | 54508104 | 54508194 |
|  | chr11 | 55189493 | 55189533 |
|  | chr11 | 57946104 | 57946193 |
|  | chr11 | 66673495 | 66673536 |
|  | chr11 | 69034296 | 69034337 |
|  | chr12 | 1713539 | 1713628 |
|  | chr12 | 21845643 | 21845684 |
|  | chr12 | 25578010 | 25578063 |
|  | chr12 | 28645568 | 28645608 |
|  | chr12 | 31603332 | 31603374 |
|  | chr12 | 35466883 | 35466926 |
|  | chr12 | 36390772 | 36390861 |
|  | chr12 | 39333424 | 39333464 |
|  | chr12 | 43857136 | 43857225 |
|  | chr12 | 51526992 | 51527033 |
|  | chr12 | 53338252 | 53338341 |
|  | chr12 | 55108493 | 55108582 |
|  | chr12 | 57897646 | 57897735 |
|  | chr12 | 61225386 | 61225432 |
|  | chr12 | 61522523 | 61522564 |
|  | chr12 | 63734921 | 63734962 |
|  | chr13 | 19892634 | 19892722 |
|  | chr13 | 26498826 | 26498913 |
|  | chr13 | 32660930 | 32661020 |
|  | chr13 | 33432920 | 33432958 |
|  | chr13 | 45836878 | 45836962 |
|  | chr13 | 48492550 | 48492639 |
|  | chr13 | 56119854 | 56119895 |
|  | chr13 | 62381729 | 62381770 |
|  | chr13 | 62716031 | 62716073 |
|  | chr14 | 3833886 | 3833924 |
|  | chr14 | 3992969 | 3993064 |
|  | chr14 | 19245725 | 19245766 |
|  | chr14 | 25087994 | 25088086 |
|  | chr14 | 28560689 | 28560730 |
|  | chr14 | 31217387 | 31217478 |
|  | chr14 | 44885104 | 44885145 |
|  | chr14 | 53013031 | 53013116 |
|  | chr14 | 59695559 | 59695598 |
|  | chr15 | 13531498 | 13531537 |
|  | chr15 | 14706527 | 14706568 |
|  | chr15 | 14967446 | 14967486 |
|  | chr15 | 18395491 | 18395531 |
|  | chr15 | 41611526 | 41611567 |
|  | chr15 | 51850864 | 51850905 |
|  | chr15 | 54953232 | 54953273 |
|  | chr15 | 56005117 | 56005166 |
|  | chr16 | 13495617 | 13495661 |
|  | chr16 | 15535449 | 15535538 |
|  | chr16 | 26447658 | 26447698 |
|  | chr16 | 26936051 | 26936089 |
|  | chr16 | 38933844 | 38933933 |
|  | chr16 | 51128387 | 51128425 |
|  | chr16 | 51861662 | 51861706 |
|  | chr17 | 4733131 | 4733176 |
|  | chr17 | 12950135 | 12950177 |
|  | chr17 | 45513709 | 45513747 |
|  | chr17 | 46193494 | 46193541 |
|  | chr17 | 62649673 | 62649711 |
|  | chr18 | 1505013 | 1505055 |
|  | chr18 | 1772605 | 1772694 |
|  | chr18 | 9834534 | 9834623 |
|  | chr18 | 25051121 | 25051163 |
|  | chr18 | 29692589 | 29692630 |
|  | chr18 | 29941641 | 29941730 |
|  | chr18 | 39629563 | 39629599 |
|  | chr18 | 50172217 | 50172258 |
|  | chr19 | 8796077 | 8796119 |
|  | chr19 | 14586796 | 14586836 |
|  | chr19 | 34740243 | 34740335 |
|  | chr19 | 37141665 | 37141703 |
|  | chr19 | 38201024 | 38201065 |
|  | chr19 | 39359750 | 39359788 |
|  | chr19 | 41779761 | 41779800 |
|  | chr19 | 43448589 | 43448631 |
|  | chr19 | 44204233 | 44204273 |
|  | chr19 | 47782033 | 47782083 |
|  | chr20 | 11923327 | 11923369 |
|  | chr20 | 40342361 | 40342402 |
|  | chr20 | 44340208 | 44340295 |
|  | chr20 | 53488569 | 53488610 |
|  | chr21 | 1610276 | 1610317 |
|  | chr21 | 2723815 | 2723864 |
|  | chr22 | 1466948 | 1467008 |
|  | chr22 | 10866813 | 10866901 |
|  | chr22 | 13992915 | 13992956 |
|  | chr22 | 16590633 | 16590722 |
|  | chr22 | 17628670 | 17628709 |
|  | chr22 | 18551429 | 18551470 |
|  | chr22 | 27576569 | 27576608 |
|  | chr22 | 28454413 | 28454453 |
|  | chr22 | 29355325 | 29355419 |
|  | chr22 | 29685307 | 29685357 |
|  | chr22 | 30636209 | 30636288 |
|  | chr22 | 34275201 | 34275240 |
|  | chr22 | 35150495 | 35150542 |
|  | chr22 | 37422940 | 37423028 |
|  | chr22 | 39950936 | 39950974 |
|  | chr22 | 40720576 | 40720665 |
|  | chr22 | 46684140 | 46684226 |
|  | chr22 | 54524791 | 54524870 |
|  | chr23 | 14096500 | 14096589 |
|  | chr23 | 16217529 | 16217570 |
|  | chr23 | 22165421 | 22165510 |
|  | chr23 | 23377140 | 23377228 |
|  | chr23 | 26445880 | 26445920 |
|  | chr23 | 36896168 | 36896257 |
|  | chr23 | 42758355 | 42758408 |
|  | chr23 | 50242529 | 50242576 |
|  | chr24 | 17836810 | 17836859 |
|  | chr24 | 21129404 | 21129451 |
|  | chr24 | 24729976 | 24730065 |
|  | chr25 | 20906864 | 20906952 |
|  | chr25 | 21080329 | 21080367 |
|  | chr25 | 23298647 | 23298688 |
|  | chr25 | 24775484 | 24775520 |
|  | chr25 | 32920132 | 32920177 |
|  | chr25 | 35292269 | 35292358 |
|  | chr25 | 36408064 | 36408105 |
|  | chr26 | 2780346 | 2780443 |
|  | chr26 | 6673174 | 6673264 |
|  | chr26 | 10242760 | 10242848 |
|  | chr26 | 14124513 | 14124602 |
|  | chr26 | 22832427 | 22832468 |
|  | chr26 | 33334044 | 33334085 |
|  | chr27 | 12333999 | 12334088 |
|  | chr27 | 12877618 | 12877662 |
|  | chr27 | 19665920 | 19666009 |
|  | chr27 | 20294722 | 20294811 |
|  | chr27 | 21200224 | 21200265 |
|  | chr28 | 3545203 | 3545244 |
|  | chr28 | 14486699 | 14486788 |
|  | chr28 | 15222346 | 15222385 |
|  | chr28 | 16700349 | 16700438 |
|  | chr30 | 5456594 | 5456635 |
|  | chr30 | 7185645 | 7185685 |
|  | chr32 | 471019 | 471108 |
|  | chr32 | 4831929 | 4831967 |
|  | chr32 | 14575179 | 14575220 |
|  | chr32 | 14974849 | 14974889 |
|  | chr32 | 18702552 | 18702590 |
|  | chr32 | 20930108 | 20930149 |
|  | chr32 | 25434268 | 25434308 |
|  | chr32 | 33637975 | 33638016 |
|  | chr32 | 34603397 | 34603486 |
|  | chr32 | 36753463 | 36753504 |
|  | chr32 | 37631506 | 37631547 |
|  | chr33 | 309300 | 309341 |
|  | chr33 | 1984270 | 1984368 |
|  | chr33 | 3093787 | 3093828 |
|  | chr33 | 4380214 | 4380254 |
|  | chr33 | 13291401 | 13291442 |
|  | chr33 | 19532215 | 19532257 |
|  | chr33 | 23955099 | 23955184 |
|  | chr33 | 24145753 | 24145790 |
|  | chr33 | 26976735 | 26976776 |
|  | chr33 | 29352589 | 29352629 |
|  | chr34 | 727551 | 727639 |
|  | chr34 | 27374053 | 27374095 |
|  | chr34 | 27705332 | 27705419 |
|  | chr34 | 37233485 | 37233528 |
|  | chr35 | 6451923 | 6452013 |
|  | chr36 | 4339890 | 4339980 |
|  | chr36 | 11380262 | 11380303 |
|  | chr36 | 14112213 | 14112249 |
|  | chr36 | 14834420 | 14834462 |
|  | chr36 | 29049180 | 29049221 |
|  | chr37 | 2617459 | 2617498 |
|  | chr37 | 2700570 | 2700615 |
|  | chr37 | 4283591 | 4283679 |
|  | chr38 | 2499134 | 2499173 |
|  | chr38 | 3971936 | 3971977 |
| ***Mus musculus*** | chr1 | 78278017 | 78278061 |
|  | chr1 | 138878287 | 138878377 |
|  | chr1 | 194733572 | 194733613 |
|  | chr2 | 12262261 | 12262302 |
|  | chr2 | 25856592 | 25856671 |
|  | chr2 | 27130891 | 27130931 |
|  | chr2 | 29459194 | 29459280 |
|  | chr2 | 34454813 | 34454853 |
|  | chr2 | 56410184 | 56410226 |
|  | chr2 | 77455468 | 77455509 |
|  | chr2 | 109323482 | 109323560 |
|  | chr2 | 112469277 | 112469364 |
|  | chr2 | 123318130 | 123318171 |
|  | chr2 | 141632483 | 141632570 |
|  | chr2 | 166655052 | 166655141 |
|  | chr3 | 22566407 | 22566457 |
|  | chr3 | 27389499 | 27389587 |
|  | chr3 | 69685468 | 69685545 |
|  | chr3 | 96672239 | 96672318 |
|  | chr3 | 127486347 | 127486389 |
|  | chr3 | 127581165 | 127581209 |
|  | chr3 | 131002567 | 131002656 |
|  | chr4 | 33180489 | 33180529 |
|  | chr4 | 49204213 | 49204256 |
|  | chr4 | 57704698 | 57704737 |
|  | chr4 | 118490477 | 118490513 |
|  | chr4 | 139405806 | 139405843 |
|  | chr4 | 149817732 | 149817821 |
|  | chr5 | 37092282 | 37092360 |
|  | chr5 | 43420993 | 43421027 |
|  | chr5 | 119297245 | 119297295 |
|  | chr6 | 6997219 | 6997309 |
|  | chr6 | 23519746 | 23519795 |
|  | chr6 | 54284441 | 54284493 |
|  | chr7 | 6512235 | 6512323 |
|  | chr7 | 26197553 | 26197594 |
|  | chr7 | 68040281 | 68040373 |
|  | chr7 | 90293734 | 90293826 |
|  | chr8 | 35957680 | 35957730 |
|  | chr8 | 47719247 | 47719336 |
|  | chr8 | 65174377 | 65174414 |
|  | chr9 | 47510880 | 47510970 |
|  | chr9 | 122041638 | 122041677 |
|  | chr10 | 19795907 | 19795948 |
|  | chr10 | 27952376 | 27952460 |
|  | chr10 | 40551249 | 40551297 |
|  | chr10 | 75379678 | 75379719 |
|  | chr10 | 118827962 | 118828050 |
|  | chr11 | 68053335 | 68053391 |
|  | chr11 | 86408826 | 86408924 |
|  | chr11 | 89217130 | 89217171 |
|  | chr11 | 104930974 | 104931066 |
|  | chr12 | 12106737 | 12106826 |
|  | chr12 | 68600970 | 68601059 |
|  | chr12 | 105645843 | 105645934 |
|  | chr13 | 81827779 | 81827820 |
|  | chr13 | 103396202 | 103396291 |
|  | chr13 | 116675881 | 116675957 |
|  | chr14 | 20081958 | 20082016 |
|  | chr14 | 30818952 | 30819034 |
|  | chr14 | 35800654 | 35800741 |
|  | chr14 | 36855955 | 36856044 |
|  | chr14 | 76269094 | 76269137 |
|  | chr14 | 110161835 | 110161924 |
|  | chr15 | 6947995 | 6948036 |
|  | chr15 | 30590290 | 30590375 |
|  | chr15 | 57433196 | 57433246 |
|  | chr15 | 79692501 | 79692590 |
|  | chr15 | 87169113 | 87169192 |
|  | chr16 | 37854781 | 37854867 |
|  | chr16 | 41217745 | 41217835 |
|  | chr16 | 89932072 | 89932155 |
|  | chr17 | 83947588 | 83947678 |
|  | chr17 | 89495296 | 89495330 |
|  | chr17 | 94764826 | 94764910 |
|  | chr18 | 6085371 | 6085460 |
|  | chr18 | 39570832 | 39570914 |
|  | chr18 | 48256800 | 48256840 |
|  | chr18 | 60101220 | 60101263 |
|  | chr19 | 16088000 | 16088041 |
|  | chr19 | 22625492 | 22625571 |
|  | chr19 | 28082574 | 28082616 |
|  | chr19 | 36299815 | 36299853 |
|  | chrX | 13215719 | 13215808 |
|  | chrX | 36427327 | 36427413 |
|  | chrX | 136281270 | 136281308 |
|  | chrX | 151985311 | 151985401 |
|  | chrX | 157638207 | 157638295 |
|  | chrX | 159449277 | 159449366 |
|  | chrX | 163192432 | 163192516 |
| ***Loxodonta africana*** | contig_650 | 41171 | 41212 |
|  | contig_1476 | 14619 | 14664 |
|  | contig_2465 | 107854 | 107891 |
|  | contig_11252 | 105435 | 105476 |
|  | contig_22048 | 23307 | 23347 |
|  | contig_22671 | 59583 | 59623 |
|  | contig_25548 | 12045 | 12086 |
|  | contig_26678 | 88902 | 88943 |
|  | contig_27409 | 109894 | 109943 |
|  | contig_32508 | 61508 | 61549 |
|  | contig_34381 | 127724 | 127775 |
|  | contig_35175 | 43158 | 43202 |
|  | contig_40266 | 15555 | 15596 |
|  | contig_42255 | 2852 | 2889 |
|  | contig_51899 | 14513 | 14554 |
|  | contig_53729 | 956 | 1043 |
|  | contig_73625 | 97582 | 97630 |
| ***Choloepus hoffmanni*** | GeneScaffold_2586 | 69586 | 69629 |
|  | GeneScaffold_4085 | 111966 | 112006 |
|  | GeneScaffold_6915 | 59369 | 59457 |
|  | scaffold_62397 | 7093 | 7180 |
|  | scaffold_132582 | 4703 | 4741 |
| ***Monodelphis domestica*** | chr1.265000001-270000000 | 3372792 | 3372881 |
|  | chr2.410000001-415000000 | 652659 | 652755 |
|  | chr3.120000001-125000000 | 4787120 | 4787159 |
|  | chr3.195000001-200000000 | 1465656 | 1465745 |
|  | chr3.450000001-455000000 | 1273700 | 1273739 |
|  | chr4.130000001-135000000 | 1899226 | 1899267 |
|  | chr7.55000001-60000000 | 4338512 | 4338553 |
|  | chr8.220000001-225000000 | 1394956 | 1394997 |
| ***Sarcophilus harrisii*** | GL834469.1 | 1711089 | 1711178 |
|  | GL834617.1 | 1301647 | 1301737 |
|  | GL834708.1 | 275420 | 275455 |
|  | GL840619.1 | 1541 | 1585 |
|  | GL841189.1 | 872657 | 872746 |
|  | GL841348.1 | 318078 | 318118 |
|  | GL849592.1 | 2818739 | 2818816 |
|  | GL849617.1 | 1021263 | 1021349 |
|  | GL856785.1 | 1636587 | 1636675 |
|  | GL856907.1 | 1472078 | 1472167 |
|  | GL856967.1 | 2063311 | 2063359 |
|  | GL861700.1 | 781002 | 781088 |
|  | GL864757.1 | 1277314 | 1277390 |
| ***Macropus eugenii*** | chr03 | 338775182 | 338775273 |
| ***Anolis carolinensis*** | chr01 | 27076844 | 27076934 |
|  | chr02 | 65361011 | 65361063 |
|  | chr02 | 153706359 | 153706399 |
|  | chr03 | 19134816 | 19134864 |
|  | chr03 | 201261258 | 201261300 |
|  | chr04 | 58504295 | 58504331 |
|  | chr05 | 100027713 | 100027750 |
|  | chr06 | 9308653 | 9308734 |
| **U2 snRNA** | | | |
| **Species** | **Chr** | **Start** | **End** |
| ***Bos taurus*** | chr01 | 33454113 | 33454146 |
|  | chr01 | 37269152 | 37269184 |
|  | chr01 | 50894840 | 50894875 |
|  | chr01 | 69620068 | 69620103 |
|  | chr01 | 74433470 | 74433504 |
|  | chr01 | 91337313 | 91337356 |
|  | chr01 | 97728834 | 97728871 |
|  | chr01 | 108419459 | 108419493 |
|  | chr01 | 115164980 | 115165014 |
|  | chr01 | 122615298 | 122615375 |
|  | chr01 | 124202562 | 124202596 |
|  | chr01 | 136551302 | 136551337 |
|  | chr01 | 151078514 | 151078549 |
|  | chr02 | 33537976 | 33538015 |
|  | chr02 | 34598188 | 34598220 |
|  | chr02 | 59596356 | 59596396 |
|  | chr02 | 74430781 | 74430812 |
|  | chr02 | 90405550 | 90405582 |
|  | chr02 | 94548635 | 94548674 |
|  | chr02 | 100016373 | 100016410 |
|  | chr02 | 102101693 | 102101723 |
|  | chr02 | 103917386 | 103917420 |
|  | chr02 | 109943595 | 109943630 |
|  | chr02 | 116140038 | 116140072 |
|  | chr02 | 116804552 | 116804586 |
|  | chr02 | 131330192 | 131330226 |
|  | chr03 | 23624309 | 23624345 |
|  | chr03 | 35801683 | 35801715 |
|  | chr03 | 48044281 | 48044315 |
|  | chr03 | 76847392 | 76847431 |
|  | chr03 | 78422434 | 78422466 |
|  | chr03 | 85692123 | 85692156 |
|  | chr03 | 110389208 | 110389250 |
|  | chr03 | 111019559 | 111019616 |
|  | chr03 | 112764126 | 112764157 |
|  | chr04 | 868973 | 869014 |
|  | chr04 | 25837325 | 25837373 |
|  | chr04 | 34940438 | 34940473 |
|  | chr04 | 37908414 | 37908452 |
|  | chr04 | 65556219 | 65556253 |
|  | chr04 | 66978920 | 66978955 |
|  | chr04 | 69726677 | 69726711 |
|  | chr04 | 79455644 | 79455678 |
|  | chr04 | 83088887 | 83088918 |
|  | chr04 | 84383364 | 84383398 |
|  | chr04 | 102372833 | 102372871 |
|  | chr04 | 114532519 | 114532551 |
|  | chr05 | 18380686 | 18380718 |
|  | chr05 | 37029120 | 37029155 |
|  | chr05 | 43032007 | 43032042 |
|  | chr05 | 59350950 | 59350984 |
|  | chr05 | 85921200 | 85921235 |
|  | chr05 | 90332290 | 90332324 |
|  | chr05 | 109514794 | 109514828 |
|  | chr06 | 6899700 | 6899749 |
|  | chr06 | 23387975 | 23388010 |
|  | chr06 | 23474558 | 23474593 |
|  | chr06 | 23819638 | 23819676 |
|  | chr06 | 27452596 | 27452635 |
|  | chr06 | 37809866 | 37809902 |
|  | chr06 | 41457136 | 41457170 |
|  | chr06 | 46337479 | 46337509 |
|  | chr06 | 63724286 | 63724324 |
|  | chr06 | 87231405 | 87231438 |
|  | chr06 | 99254262 | 99254291 |
|  | chr06 | 115176684 | 115176714 |
|  | chr06 | 116797431 | 116797469 |
|  | chr06 | 116804924 | 116804962 |
|  | chr06 | 117523470 | 117523503 |
|  | chr07 | 12579373 | 12579413 |
|  | chr07 | 16662009 | 16662043 |
|  | chr07 | 18924098 | 18924132 |
|  | chr07 | 22767655 | 22767685 |
|  | chr07 | 33221738 | 33221773 |
|  | chr07 | 38634907 | 38634940 |
|  | chr07 | 38967405 | 38967440 |
|  | chr07 | 68059185 | 68059218 |
|  | chr07 | 74190892 | 74190951 |
|  | chr07 | 77233923 | 77233959 |
|  | chr07 | 87179044 | 87179077 |
|  | chr07 | 111336755 | 111336789 |
|  | chr08 | 2233446 | 2233484 |
|  | chr08 | 20647417 | 20647451 |
|  | chr08 | 24222435 | 24222469 |
|  | chr08 | 43121115 | 43121154 |
|  | chr08 | 44773415 | 44773449 |
|  | chr08 | 48020173 | 48020207 |
|  | chr08 | 51356499 | 51356536 |
|  | chr08 | 52373677 | 52373730 |
|  | chr08 | 59981793 | 59981830 |
|  | chr08 | 77069405 | 77069440 |
|  | chr08 | 77397751 | 77397786 |
|  | chr08 | 91862359 | 91862418 |
|  | chr09 | 7102965 | 7103000 |
|  | chr09 | 15119495 | 15119534 |
|  | chr09 | 39824752 | 39824788 |
|  | chr09 | 48161415 | 48161450 |
|  | chr09 | 52956308 | 52956343 |
|  | chr09 | 66341650 | 66341685 |
|  | chr09 | 84671158 | 84671192 |
|  | chr09 | 89639484 | 89639519 |
|  | chr10 | 5579862 | 5579901 |
|  | chr10 | 25668846 | 25668879 |
|  | chr10 | 37218840 | 37218869 |
|  | chr10 | 45772661 | 45772695 |
|  | chr10 | 55480778 | 55480810 |
|  | chr10 | 67607610 | 67607642 |
|  | chr10 | 71750139 | 71750173 |
|  | chr10 | 73201031 | 73201071 |
|  | chr10 | 81529023 | 81529108 |
|  | chr10 | 84985242 | 84985274 |
|  | chr10 | 92726655 | 92726689 |
|  | chr10 | 103933668 | 103933703 |
|  | chr11 | 11080809 | 11080844 |
|  | chr11 | 21641987 | 21642024 |
|  | chr11 | 21670492 | 21670526 |
|  | chr11 | 24992530 | 24992565 |
|  | chr11 | 25390552 | 25390589 |
|  | chr11 | 36997320 | 36997353 |
|  | chr11 | 38654902 | 38654959 |
|  | chr11 | 46606238 | 46606268 |
|  | chr11 | 47034105 | 47034139 |
|  | chr11 | 52751689 | 52751720 |
|  | chr11 | 55634039 | 55634074 |
|  | chr12 | 21933168 | 21933200 |
|  | chr12 | 41448905 | 41448948 |
|  | chr12 | 51289880 | 51289926 |
|  | chr12 | 57950775 | 57950809 |
|  | chr12 | 80367427 | 80367460 |
|  | chr13 | 2461253 | 2461288 |
|  | chr13 | 7423418 | 7423453 |
|  | chr13 | 53911021 | 53911060 |
|  | chr13 | 58701858 | 58701890 |
|  | chr13 | 61057491 | 61057526 |
|  | chr13 | 73795176 | 73795235 |
|  | chr13 | 79785481 | 79785512 |
|  | chr14 | 12095530 | 12095562 |
|  | chr14 | 39216250 | 39216282 |
|  | chr14 | 43723560 | 43723600 |
|  | chr14 | 45903022 | 45903054 |
|  | chr14 | 67505437 | 67505475 |
|  | chr15 | 20750450 | 20750484 |
|  | chr15 | 21845559 | 21845612 |
|  | chr15 | 36532549 | 36532583 |
|  | chr15 | 64742750 | 64742784 |
|  | chr15 | 67875090 | 67875123 |
|  | chr15 | 75145729 | 75145762 |
|  | chr16 | 8850073 | 8850113 |
|  | chr16 | 24513463 | 24513498 |
|  | chr16 | 27944199 | 27944236 |
|  | chr16 | 39129308 | 39129341 |
|  | chr16 | 47681688 | 47681739 |
|  | chr16 | 56090216 | 56090250 |
|  | chr16 | 62484006 | 62484050 |
|  | chr17 | 12709857 | 12709890 |
|  | chr17 | 27524285 | 27524320 |
|  | chr17 | 57063315 | 57063349 |
|  | chr17 | 63696399 | 63696434 |
|  | chr17 | 66368667 | 66368709 |
|  | chr17 | 68738354 | 68738418 |
|  | chr18 | 1072160 | 1072198 |
|  | chr18 | 15943260 | 15943294 |
|  | chr18 | 30137297 | 30137331 |
|  | chr18 | 35712900 | 35712942 |
|  | chr18 | 43327414 | 43327456 |
|  | chr18 | 58707341 | 58707373 |
|  | chr18 | 64702161 | 64702199 |
|  | chr19 | 10156898 | 10156934 |
|  | chr19 | 13975435 | 13975469 |
|  | chr19 | 15987349 | 15987393 |
|  | chr19 | 16436983 | 16437017 |
|  | chr19 | 25422584 | 25422614 |
|  | chr19 | 31382875 | 31382920 |
|  | chr19 | 32279259 | 32279293 |
|  | chr19 | 40357790 | 40357826 |
|  | chr19 | 55437142 | 55437176 |
|  | chr19 | 57018059 | 57018093 |
|  | chr20 | 5150941 | 5150975 |
|  | chr20 | 14136619 | 14136656 |
|  | chr20 | 19217551 | 19217585 |
|  | chr20 | 35144039 | 35144073 |
|  | chr20 | 37738505 | 37738540 |
|  | chr20 | 42050038 | 42050072 |
|  | chr20 | 42184615 | 42184649 |
|  | chr20 | 64958149 | 64958183 |
|  | chr21 | 39721105 | 39721136 |
|  | chr21 | 42131068 | 42131102 |
|  | chr21 | 42700241 | 42700280 |
|  | chr21 | 49539533 | 49539568 |
|  | chr21 | 53375655 | 53375689 |
|  | chr22 | 14761537 | 14761574 |
|  | chr22 | 30497364 | 30497398 |
|  | chr22 | 35315712 | 35315752 |
|  | chr22 | 46782640 | 46782690 |
|  | chr22 | 55540297 | 55540346 |
|  | chr23 | 7397881 | 7397916 |
|  | chr23 | 20117102 | 20117153 |
|  | chr23 | 37828985 | 37829019 |
|  | chr23 | 41419069 | 41419103 |
|  | chr23 | 49608536 | 49608571 |
|  | chr24 | 8583480 | 8583513 |
|  | chr24 | 16661142 | 16661181 |
|  | chr24 | 34564248 | 34564282 |
|  | chr24 | 35132242 | 35132300 |
|  | chr24 | 36835002 | 36835036 |
|  | chr24 | 46044001 | 46044036 |
|  | chr24 | 47727457 | 47727490 |
|  | chr24 | 50114189 | 50114223 |
|  | chr24 | 60031088 | 60031124 |
|  | chr25 | 15229471 | 15229507 |
|  | chr25 | 22067667 | 22067702 |
|  | chr25 | 40715416 | 40715450 |
|  | chr26 | 2650063 | 2650097 |
|  | chr26 | 18743044 | 18743084 |
|  | chr26 | 36849225 | 36849259 |
|  | chr27 | 1992656 | 1992687 |
|  | chr28 | 1029930 | 1029964 |
|  | chr28 | 13362637 | 13362671 |
|  | chr28 | 14177217 | 14177251 |
|  | chr28 | 27855879 | 27855940 |
|  | chr28 | 34682585 | 34682615 |
|  | chr28 | 35961477 | 35961511 |
|  | chr28 | 36650009 | 36650038 |
|  | chr29 | 9441691 | 9441726 |
|  | chr29 | 14723544 | 14723578 |
|  | chr29 | 25742021 | 25742056 |
|  | chr29 | 31303832 | 31303867 |
|  | chr29 | 32728718 | 32728751 |
|  | chr29 | 38145405 | 38145437 |
|  | chr29 | 46651820 | 46651855 |
|  | chrX | 648432 | 648469 |
|  | chrX | 17121270 | 17121303 |
|  | chrX | 23259351 | 23259386 |
|  | chrX | 25835676 | 25835710 |
|  | chrX | 32097067 | 32097114 |
|  | chrX | 46146947 | 46146994 |
|  | chrX | 83019504 | 83019540 |
|  | chrX | 85364143 | 85364181 |
|  | chrX | 86948359 | 86948394 |
|  | chrX | 90367425 | 90367459 |
|  | chrX | 103701898 | 103701938 |
|  | chrX | 110941393 | 110941432 |
|  | chrX | 121432062 | 121432106 |
|  | chrX | 125084770 | 125084802 |
|  | chrX | 128501642 | 128501681 |
|  | chrX | 129245045 | 129245085 |
|  | chrX | 132297306 | 132297340 |
|  | chrX | 142035395 | 142035429 |
| ***Canis lupus familiaris*** | chr01 | 5475880 | 5475913 |
|  | chr01 | 8236489 | 8236523 |
|  | chr01 | 18262540 | 18262574 |
|  | chr01 | 19362014 | 19362043 |
|  | chr01 | 25631927 | 25631961 |
|  | chr01 | 25719834 | 25719868 |
|  | chr01 | 30193125 | 30193156 |
|  | chr01 | 38729043 | 38729075 |
|  | chr01 | 41885937 | 41885974 |
|  | chr01 | 43291485 | 43291519 |
|  | chr01 | 49643827 | 49643882 |
|  | chr01 | 56051728 | 56051758 |
|  | chr01 | 60409049 | 60409078 |
|  | chr01 | 60852752 | 60852786 |
|  | chr01 | 61294630 | 61294670 |
|  | chr01 | 63097577 | 63097613 |
|  | chr01 | 67294379 | 67294413 |
|  | chr01 | 67501291 | 67501324 |
|  | chr01 | 75309867 | 75309902 |
|  | chr01 | 80087170 | 80087204 |
|  | chr01 | 81332396 | 81332429 |
|  | chr01 | 84605104 | 84605142 |
|  | chr01 | 85548008 | 85548043 |
|  | chr01 | 86450630 | 86450664 |
|  | chr01 | 89032367 | 89032400 |
|  | chr01 | 89747494 | 89747529 |
|  | chr01 | 91726117 | 91726149 |
|  | chr01 | 93774747 | 93774793 |
|  | chr01 | 94175293 | 94175323 |
|  | chr01 | 95441218 | 95441250 |
|  | chr01 | 95824518 | 95824556 |
|  | chr01 | 99519181 | 99519216 |
|  | chr01 | 103022323 | 103022357 |
|  | chr01 | 103137791 | 103137828 |
|  | chr01 | 104115072 | 104115105 |
|  | chr01 | 104168035 | 104168068 |
|  | chr01 | 104251346 | 104251379 |
|  | chr01 | 104403233 | 104403266 |
|  | chr01 | 104479825 | 104479858 |
|  | chr01 | 104625737 | 104625772 |
|  | chr01 | 108969853 | 108969888 |
|  | chr01 | 117054809 | 117054841 |
|  | chr01 | 118075920 | 118075960 |
|  | chr02 | 21526134 | 21526169 |
|  | chr02 | 35948460 | 35948502 |
|  | chr02 | 46952340 | 46952374 |
|  | chr02 | 47593458 | 47593493 |
|  | chr02 | 51098820 | 51098854 |
|  | chr02 | 56496056 | 56496094 |
|  | chr02 | 58487345 | 58487377 |
|  | chr02 | 65517264 | 65517296 |
|  | chr02 | 72248995 | 72249027 |
|  | chr02 | 77175228 | 77175261 |
|  | chr02 | 77510875 | 77510911 |
|  | chr03 | 721085 | 721120 |
|  | chr03 | 9464359 | 9464394 |
|  | chr03 | 12189739 | 12189774 |
|  | chr03 | 19527094 | 19527128 |
|  | chr03 | 24437377 | 24437412 |
|  | chr03 | 25088130 | 25088174 |
|  | chr03 | 27469781 | 27469821 |
|  | chr03 | 27905137 | 27905170 |
|  | chr03 | 30220927 | 30220965 |
|  | chr03 | 32856351 | 32856386 |
|  | chr03 | 37264776 | 37264822 |
|  | chr03 | 37937991 | 37938027 |
|  | chr03 | 38110223 | 38110254 |
|  | chr03 | 38855424 | 38855457 |
|  | chr03 | 54450557 | 54450593 |
|  | chr03 | 62988303 | 62988337 |
|  | chr03 | 64312254 | 64312287 |
|  | chr03 | 69537913 | 69537951 |
|  | chr03 | 73608287 | 73608317 |
|  | chr03 | 75031263 | 75031305 |
|  | chr03 | 79080468 | 79080503 |
|  | chr03 | 81273523 | 81273557 |
|  | chr03 | 81993545 | 81993584 |
|  | chr03 | 83734772 | 83734806 |
|  | chr03 | 84209943 | 84209976 |
|  | chr03 | 86671878 | 86671915 |
|  | chr04 | 9693213 | 9693250 |
|  | chr04 | 9731541 | 9731583 |
|  | chr04 | 14206830 | 14206864 |
|  | chr04 | 19116938 | 19116979 |
|  | chr04 | 19449871 | 19449901 |
|  | chr04 | 19547384 | 19547426 |
|  | chr04 | 19982409 | 19982450 |
|  | chr04 | 21328091 | 21328129 |
|  | chr05 | 4214998 | 4215035 |
|  | chr05 | 4915491 | 4915528 |
|  | chr05 | 10323657 | 10323693 |
|  | chr05 | 10444562 | 10444596 |
|  | chr06 | 808359 | 808391 |
|  | chr06 | 10034251 | 10034284 |
|  | chr06 | 12079455 | 12079502 |
|  | chr06 | 13831751 | 13831786 |
|  | chr07 | 6752252 | 6752292 |
|  | chr07 | 8144142 | 8144186 |
|  | chr07 | 8903123 | 8903155 |
|  | chr07 | 9778005 | 9778039 |
|  | chr07 | 10300142 | 10300183 |
|  | chr07 | 12530669 | 12530701 |
|  | chr07 | 12843507 | 12843539 |
|  | chr07 | 13886235 | 13886268 |
|  | chr07 | 14362861 | 14362893 |
|  | chr08 | 1302018 | 1302050 |
|  | chr08 | 2540585 | 2540619 |
|  | chr08 | 10932943 | 10932977 |
|  | chr08 | 11492811 | 11492847 |
|  | chr08 | 13420766 | 13420801 |
|  | chr08 | 13455935 | 13455970 |
|  | chr08 | 16369706 | 16369745 |
|  | chr08 | 23248542 | 23248580 |
|  | chr08 | 29305112 | 29305147 |
|  | chr08 | 31301948 | 31301983 |
|  | chr08 | 32114695 | 32114727 |
|  | chr08 | 35775165 | 35775210 |
|  | chr08 | 38020532 | 38020566 |
|  | chr08 | 39884019 | 39884053 |
|  | chr09 | 1461947 | 1461979 |
|  | chr09 | 9094310 | 9094344 |
|  | chr09 | 9127334 | 9127367 |
|  | chr09 | 9455826 | 9455858 |
|  | chr09 | 11307628 | 11307657 |
|  | chr09 | 13317032 | 13317066 |
|  | chr09 | 14037976 | 14038010 |
|  | chr09 | 15054576 | 15054608 |
|  | chr09 | 21980746 | 21980780 |
|  | chr10 | 15067055 | 15067093 |
|  | chr10 | 23673451 | 23673483 |
|  | chr10 | 30933168 | 30933203 |
|  | chr10 | 31736467 | 31736502 |
|  | chr10 | 42937840 | 42937872 |
|  | chr10 | 44754652 | 44754687 |
|  | chr10 | 50232810 | 50232844 |
|  | chr10 | 50778114 | 50778146 |
|  | chr10 | 55081946 | 55081977 |
|  | chr10 | 57868456 | 57868487 |
|  | chr10 | 64322785 | 64322821 |
|  | chr11 | 1448659 | 1448692 |
|  | chr11 | 5116101 | 5116136 |
|  | chr11 | 12514398 | 12514430 |
|  | chr11 | 14201150 | 14201185 |
|  | chr11 | 22611951 | 22611991 |
|  | chr11 | 26035684 | 26035715 |
|  | chr11 | 30205938 | 30205972 |
|  | chr11 | 31333836 | 31333871 |
|  | chr11 | 35849492 | 35849531 |
|  | chr11 | 36623160 | 36623195 |
|  | chr11 | 37931963 | 37932001 |
|  | chr11 | 38805368 | 38805402 |
|  | chr11 | 46473355 | 46473389 |
|  | chr11 | 47379945 | 47379980 |
|  | chr11 | 47937517 | 47937549 |
|  | chr11 | 53403232 | 53403267 |
|  | chr11 | 53515673 | 53515707 |
|  | chr11 | 55756323 | 55756358 |
|  | chr11 | 55945524 | 55945558 |
|  | chr11 | 58947728 | 58947784 |
|  | chr11 | 61668513 | 61668553 |
|  | chr11 | 62506836 | 62506871 |
|  | chr11 | 65161635 | 65161666 |
|  | chr11 | 66180307 | 66180341 |
|  | chr11 | 68124184 | 68124216 |
|  | chr11 | 73651825 | 73651860 |
|  | chr11 | 74309740 | 74309780 |
|  | chr12 | 1618954 | 1618989 |
|  | chr12 | 4825976 | 4826010 |
|  | chr12 | 5645641 | 5645675 |
|  | chr12 | 12049518 | 12049552 |
|  | chr12 | 14124890 | 14124925 |
|  | chr12 | 14165786 | 14165837 |
|  | chr12 | 18107567 | 18107602 |
|  | chr12 | 20586166 | 20586220 |
|  | chr12 | 20642475 | 20642508 |
|  | chr12 | 22392641 | 22392677 |
|  | chr12 | 22486909 | 22486943 |
|  | chr12 | 28392851 | 28392908 |
|  | chr12 | 28939240 | 28939275 |
|  | chr12 | 30114397 | 30114436 |
|  | chr12 | 31565520 | 31565552 |
|  | chr12 | 34082905 | 34082937 |
|  | chr12 | 34126983 | 34127015 |
|  | chr12 | 43275848 | 43275885 |
|  | chr12 | 52457756 | 52457788 |
|  | chr12 | 53460279 | 53460312 |
|  | chr12 | 58821086 | 58821120 |
|  | chr12 | 61207733 | 61207767 |
|  | chr12 | 64765149 | 64765183 |
|  | chr12 | 69941496 | 69941534 |
|  | chr12 | 69997656 | 69997693 |
|  | chr12 | 70553812 | 70553846 |
|  | chr12 | 71600652 | 71600682 |
|  | chr13 | 1181570 | 1181604 |
|  | chr13 | 1827026 | 1827060 |
|  | chr13 | 4504045 | 4504080 |
|  | chr13 | 8410887 | 8410945 |
|  | chr13 | 14888195 | 14888230 |
|  | chr13 | 21640513 | 21640547 |
|  | chr13 | 23485341 | 23485375 |
|  | chr13 | 24303728 | 24303761 |
|  | chr13 | 26500154 | 26500192 |
|  | chr13 | 38686876 | 38686911 |
|  | chr13 | 41860799 | 41860855 |
|  | chr13 | 41978857 | 41978889 |
|  | chr13 | 42251169 | 42251202 |
|  | chr13 | 43963062 | 43963103 |
|  | chr13 | 45734143 | 45734177 |
|  | chr13 | 45893954 | 45893986 |
|  | chr13 | 46283296 | 46283333 |
|  | chr13 | 48203867 | 48203910 |
|  | chr13 | 48512408 | 48512464 |
|  | chr13 | 54669279 | 54669310 |
|  | chr13 | 55563615 | 55563649 |
|  | chr13 | 58313044 | 58313076 |
|  | chr13 | 61350975 | 61351031 |
|  | chr14 | 2613380 | 2613414 |
|  | chr14 | 17697369 | 17697401 |
|  | chr14 | 17852371 | 17852404 |
|  | chr14 | 20397575 | 20397608 |
|  | chr14 | 23085110 | 23085149 |
|  | chr14 | 26011841 | 26011876 |
|  | chr14 | 29059122 | 29059156 |
|  | chr14 | 33363213 | 33363247 |
|  | chr14 | 45237292 | 45237329 |
|  | chr14 | 49505555 | 49505590 |
|  | chr14 | 57175128 | 57175169 |
|  | chr15 | 1757049 | 1757083 |
|  | chr15 | 1757317 | 1757351 |
|  | chr15 | 1873875 | 1873919 |
|  | chr15 | 4656921 | 4656956 |
|  | chr15 | 4703065 | 4703100 |
|  | chr15 | 9626925 | 9626959 |
|  | chr15 | 14434798 | 14434843 |
|  | chr15 | 14561390 | 14561428 |
|  | chr15 | 15407983 | 15408016 |
|  | chr15 | 15863597 | 15863629 |
|  | chr15 | 16705143 | 16705175 |
|  | chr15 | 18284575 | 18284609 |
|  | chr15 | 30426905 | 30426938 |
|  | chr15 | 30702499 | 30702533 |
|  | chr15 | 33583112 | 33583144 |
|  | chr15 | 33597160 | 33597195 |
|  | chr15 | 35443887 | 35443924 |
|  | chr15 | 53411612 | 53411642 |
|  | chr15 | 55542766 | 55542798 |
|  | chr15 | 61014585 | 61014619 |
|  | chr15 | 63203553 | 63203586 |
|  | chr16 | 3061807 | 3061840 |
|  | chr16 | 8172667 | 8172725 |
|  | chr16 | 10015356 | 10015390 |
|  | chr16 | 14416433 | 14416471 |
|  | chr16 | 16510527 | 16510559 |
|  | chr16 | 23235555 | 23235590 |
|  | chr16 | 25821068 | 25821105 |
|  | chr16 | 28693732 | 28693765 |
|  | chr16 | 30314271 | 30314305 |
|  | chr16 | 34181900 | 34181935 |
|  | chr16 | 48144572 | 48144606 |
|  | chr16 | 53002092 | 53002124 |
|  | chr16 | 53156976 | 53157010 |
|  | chr16 | 53441723 | 53441762 |
|  | chr16 | 56247406 | 56247439 |
|  | chr17 | 567141 | 567175 |
|  | chr17 | 6577947 | 6577981 |
|  | chr17 | 12986099 | 12986133 |
|  | chr17 | 14253510 | 14253542 |
|  | chr17 | 14626573 | 14626613 |
|  | chr17 | 21717126 | 21717160 |
|  | chr17 | 24231742 | 24231776 |
|  | chr17 | 37375725 | 37375759 |
|  | chr17 | 37698097 | 37698131 |
|  | chr17 | 48133457 | 48133516 |
|  | chr17 | 48391793 | 48391872 |
|  | chr17 | 48425765 | 48425812 |
|  | chr17 | 48525531 | 48525565 |
|  | chr17 | 49347807 | 49347845 |
|  | chr17 | 54067566 | 54067600 |
|  | chr17 | 58589922 | 58589957 |
|  | chr17 | 60037936 | 60037976 |
|  | chr17 | 60614966 | 60614998 |
|  | chr17 | 60658676 | 60658710 |
|  | chr17 | 62135897 | 62135929 |
|  | chr17 | 62957228 | 62957262 |
|  | chr17 | 62976422 | 62976454 |
|  | chr18 | 2157343 | 2157377 |
|  | chr18 | 8154130 | 8154165 |
|  | chr18 | 10163084 | 10163118 |
|  | chr18 | 20099793 | 20099827 |
|  | chr18 | 21432055 | 21432100 |
|  | chr18 | 37182288 | 37182321 |
|  | chr18 | 38640134 | 38640168 |
|  | chr18 | 39993652 | 39993684 |
|  | chr18 | 42690315 | 42690350 |
|  | chr18 | 44801591 | 44801626 |
|  | chr19 | 1998244 | 1998278 |
|  | chr19 | 6589704 | 6589738 |
|  | chr19 | 9086944 | 9086984 |
|  | chr19 | 13968330 | 13968365 |
|  | chr19 | 17899190 | 17899224 |
|  | chr19 | 20630164 | 20630196 |
|  | chr19 | 29437622 | 29437655 |
|  | chr19 | 30429217 | 30429251 |
|  | chr19 | 35322701 | 35322733 |
|  | chr19 | 40778492 | 40778531 |
|  | chr19 | 43344907 | 43344941 |
|  | chr19 | 44010834 | 44010868 |
|  | chr19 | 46118396 | 46118431 |
|  | chr19 | 51844115 | 51844147 |
|  | chr20 | 5451615 | 5451651 |
|  | chr20 | 6429629 | 6429661 |
|  | chr20 | 8400138 | 8400173 |
|  | chr20 | 10165407 | 10165446 |
|  | chr20 | 18098778 | 18098814 |
|  | chr20 | 23632567 | 23632598 |
|  | chr20 | 25382593 | 25382628 |
|  | chr20 | 29002466 | 29002501 |
|  | chr20 | 29303272 | 29303307 |
|  | chr20 | 38007437 | 38007472 |
|  | chr20 | 42171659 | 42171693 |
|  | chr20 | 46419525 | 46419562 |
|  | chr20 | 52903913 | 52903949 |
|  | chr21 | 2448968 | 2449002 |
|  | chr21 | 3091824 | 3091856 |
|  | chr21 | 10336079 | 10336138 |
|  | chr21 | 12463881 | 12463916 |
|  | chr22 | 5774385 | 5774417 |
|  | chr22 | 9660062 | 9660104 |
|  | chr22 | 17087955 | 17087988 |
|  | chr22 | 18668897 | 18668934 |
|  | chr22 | 18813524 | 18813558 |
|  | chr22 | 20629087 | 20629126 |
|  | chr22 | 20784853 | 20784882 |
|  | chr22 | 23403585 | 23403619 |
|  | chr22 | 27412534 | 27412565 |
|  | chr22 | 29480846 | 29480880 |
|  | chr22 | 34206454 | 34206489 |
|  | chr22 | 46317286 | 46317321 |
|  | chr22 | 46516619 | 46516659 |
|  | chr22 | 48031524 | 48031556 |
|  | chr22 | 50614629 | 50614664 |
|  | chr22 | 53468342 | 53468377 |
|  | chr22 | 57136931 | 57136971 |
|  | chr22 | 59680475 | 59680509 |
|  | chr23 | 7087551 | 7087585 |
|  | chr23 | 9466151 | 9466190 |
|  | chr23 | 9492111 | 9492144 |
|  | chr23 | 15095232 | 15095277 |
|  | chr23 | 19935166 | 19935201 |
|  | chr23 | 24071274 | 24071310 |
|  | chr23 | 37112043 | 37112077 |
|  | chr23 | 37598843 | 37598877 |
|  | chr23 | 37856400 | 37856433 |
|  | chr23 | 40147550 | 40147626 |
|  | chr23 | 42010675 | 42010709 |
|  | chr23 | 43957939 | 43957980 |
|  | chr23 | 45288009 | 45288050 |
|  | chr23 | 50329993 | 50330028 |
|  | chr23 | 51349011 | 51349043 |
|  | chr23 | 51838897 | 51838938 |
|  | chr23 | 51862046 | 51862083 |
|  | chr24 | 1572885 | 1572919 |
|  | chr24 | 3265844 | 3265879 |
|  | chr24 | 8310087 | 8310121 |
|  | chr24 | 10622185 | 10622217 |
|  | chr24 | 15014419 | 15014453 |
|  | chr24 | 21360546 | 21360579 |
|  | chr24 | 23880163 | 23880199 |
|  | chr24 | 25929194 | 25929226 |
|  | chr24 | 29462871 | 29462906 |
|  | chr24 | 36606982 | 36607016 |
|  | chr24 | 39926932 | 39926970 |
|  | chr25 | 2104443 | 2104475 |
|  | chr25 | 4522748 | 4522780 |
|  | chr25 | 9311626 | 9311676 |
|  | chr25 | 11446044 | 11446085 |
|  | chr25 | 14723154 | 14723190 |
|  | chr25 | 18558389 | 18558424 |
|  | chr25 | 19002068 | 19002107 |
|  | chr25 | 33360596 | 33360630 |
|  | chr25 | 34535844 | 34535880 |
|  | chr25 | 43601338 | 43601371 |
|  | chr25 | 44002258 | 44002289 |
|  | chr26 | 4726510 | 4726544 |
|  | chr26 | 14558571 | 14558606 |
|  | chr26 | 22719912 | 22719954 |
|  | chr26 | 23300786 | 23300827 |
|  | chr26 | 31200371 | 31200407 |
|  | chr26 | 34500492 | 34500525 |
|  | chr27 | 4300984 | 4301016 |
|  | chr27 | 6242162 | 6242212 |
|  | chr27 | 6765693 | 6765728 |
|  | chr27 | 7072070 | 7072107 |
|  | chr27 | 7214433 | 7214467 |
|  | chr27 | 7227193 | 7227228 |
|  | chr27 | 8251156 | 8251189 |
|  | chr27 | 9597072 | 9597108 |
|  | chr27 | 12728291 | 12728323 |
|  | chr27 | 19483222 | 19483258 |
|  | chr27 | 23082052 | 23082089 |
|  | chr27 | 23607481 | 23607516 |
|  | chr27 | 23652602 | 23652682 |
|  | chr27 | 24899616 | 24899651 |
|  | chr27 | 30157793 | 30157832 |
|  | chr27 | 30731171 | 30731204 |
|  | chr27 | 33016134 | 33016175 |
|  | chr27 | 34458561 | 34458596 |
|  | chr27 | 34473149 | 34473183 |
|  | chr28 | 4912056 | 4912090 |
|  | chr28 | 7408802 | 7408836 |
|  | chr28 | 8201422 | 8201456 |
|  | chr28 | 10147574 | 10147615 |
|  | chr28 | 14509849 | 14509882 |
|  | chr28 | 16806927 | 16806961 |
|  | chr30 | 2048379 | 2048412 |
|  | chr30 | 7309196 | 7309228 |
|  | chr30 | 7883800 | 7883835 |
|  | chr30 | 10197701 | 10197740 |
|  | chr32 | 139363 | 139398 |
|  | chr32 | 1295861 | 1295904 |
|  | chr32 | 2118862 | 2118896 |
|  | chr32 | 9047989 | 9048023 |
|  | chr32 | 11798811 | 11798853 |
|  | chr32 | 11883526 | 11883559 |
|  | chr32 | 15276450 | 15276484 |
|  | chr32 | 15349870 | 15349905 |
|  | chr32 | 19733235 | 19733268 |
|  | chr32 | 23695483 | 23695517 |
|  | chr32 | 25901536 | 25901568 |
|  | chr32 | 26160402 | 26160438 |
|  | chr32 | 30063059 | 30063103 |
|  | chr32 | 32652336 | 32652370 |
|  | chr32 | 37353699 | 37353734 |
|  | chr32 | 37616843 | 37616882 |
|  | chr33 | 1218740 | 1218772 |
|  | chr33 | 7865863 | 7865897 |
|  | chr33 | 8088180 | 8088215 |
|  | chr33 | 9600157 | 9600192 |
|  | chr33 | 17025516 | 17025550 |
|  | chr33 | 18323991 | 18324037 |
|  | chr33 | 19085104 | 19085139 |
|  | chr33 | 24091581 | 24091615 |
|  | chr33 | 28093479 | 28093519 |
|  | chr33 | 29274293 | 29274326 |
|  | chr33 | 29895327 | 29895361 |
|  | chr34 | 2120403 | 2120448 |
|  | chr34 | 6616758 | 6616789 |
|  | chr34 | 9624332 | 9624366 |
|  | chr34 | 12255770 | 12255804 |
|  | chr34 | 12458419 | 12458453 |
|  | chr34 | 12561049 | 12561083 |
|  | chr34 | 12798700 | 12798740 |
|  | chr34 | 15849285 | 15849320 |
|  | chr34 | 17866160 | 17866195 |
|  | chr34 | 18747180 | 18747213 |
|  | chr34 | 18963090 | 18963124 |
|  | chr34 | 23893466 | 23893502 |
|  | chr34 | 26530494 | 26530525 |
|  | chr34 | 27782223 | 27782257 |
|  | chr34 | 31956986 | 31957065 |
|  | chr34 | 32053345 | 32053380 |
|  | chr34 | 32308340 | 32308396 |
|  | chr36 | 3595830 | 3595865 |
|  | chr36 | 6333666 | 6333716 |
|  | chr36 | 12524065 | 12524097 |
|  | chr36 | 16593573 | 16593603 |
|  | chr36 | 18566654 | 18566688 |
|  | chr36 | 20589794 | 20589828 |
|  | chr36 | 21727047 | 21727081 |
|  | chr36 | 26132100 | 26132131 |
|  | chr36 | 27135737 | 27135768 |
|  | chr36 | 28704497 | 28704529 |
|  | chr37 | 5969418 | 5969450 |
|  | chr38 | 52570 | 52605 |
|  | chr38 | 237592 | 237632 |
|  | chr38 | 341455 | 341496 |
|  | chr38 | 6008521 | 6008555 |
|  | chr38 | 8545809 | 8545844 |
|  | chr38 | 11028373 | 11028408 |
|  | chr38 | 11915429 | 11915462 |
|  | chr38 | 13309818 | 13309852 |
|  | chr38 | 14252549 | 14252588 |
| ***Mus musculus*** | chr1 | 14911897 | 14911932 |
|  | chr1 | 16067759 | 16067794 |
|  | chr1 | 28382837 | 28382872 |
|  | chr1 | 29742975 | 29743009 |
|  | chr1 | 31717111 | 31717145 |
|  | chr1 | 37847247 | 37847282 |
|  | chr1 | 40521891 | 40521926 |
|  | chr1 | 42998838 | 42998872 |
|  | chr1 | 43034666 | 43034703 |
|  | chr1 | 45970327 | 45970361 |
|  | chr1 | 45982359 | 45982394 |
|  | chr1 | 63025755 | 63025790 |
|  | chr1 | 66658003 | 66658038 |
|  | chr1 | 68365479 | 68365514 |
|  | chr1 | 74365797 | 74365834 |
|  | chr1 | 78184770 | 78184805 |
|  | chr1 | 78667958 | 78667992 |
|  | chr1 | 81970690 | 81970725 |
|  | chr1 | 86583973 | 86584011 |
|  | chr1 | 86707339 | 86707373 |
|  | chr1 | 87421149 | 87421183 |
|  | chr1 | 91437939 | 91437974 |
|  | chr1 | 92892786 | 92892821 |
|  | chr1 | 97011635 | 97011669 |
|  | chr1 | 98363071 | 98363105 |
|  | chr1 | 108268620 | 108268654 |
|  | chr1 | 108853959 | 108853994 |
|  | chr1 | 111426069 | 111426104 |
|  | chr1 | 111850653 | 111850687 |
|  | chr1 | 111941012 | 111941047 |
|  | chr1 | 118539408 | 118539443 |
|  | chr1 | 118963318 | 118963353 |
|  | chr1 | 128370533 | 128370567 |
|  | chr1 | 129362482 | 129362517 |
|  | chr1 | 136905785 | 136905826 |
|  | chr1 | 147835836 | 147835872 |
|  | chr1 | 151260874 | 151260914 |
|  | chr1 | 151658363 | 151658398 |
|  | chr1 | 154313162 | 154313208 |
|  | chr1 | 161367342 | 161367376 |
|  | chr1 | 162288354 | 162288391 |
|  | chr1 | 164949929 | 164949959 |
|  | chr1 | 167657298 | 167657333 |
|  | chr1 | 169718405 | 169718440 |
|  | chr1 | 170093839 | 170093875 |
|  | chr1 | 174868234 | 174868272 |
|  | chr1 | 175447353 | 175447385 |
|  | chr1 | 182355959 | 182355993 |
|  | chr1 | 186810912 | 186810947 |
|  | chr1 | 190029799 | 190029833 |
|  | chr1 | 193025734 | 193025764 |
|  | chr2 | 4925751 | 4925785 |
|  | chr2 | 6166946 | 6166981 |
|  | chr2 | 8824774 | 8824808 |
|  | chr2 | 9752964 | 9752999 |
|  | chr2 | 19888665 | 19888700 |
|  | chr2 | 19945855 | 19945888 |
|  | chr2 | 20804277 | 20804312 |
|  | chr2 | 21142940 | 21142971 |
|  | chr2 | 27389118 | 27389152 |
|  | chr2 | 40095212 | 40095249 |
|  | chr2 | 40923699 | 40923735 |
|  | chr2 | 46412648 | 46412683 |
|  | chr2 | 58472561 | 58472595 |
|  | chr2 | 58788551 | 58788580 |
|  | chr2 | 68064710 | 68064755 |
|  | chr2 | 68843106 | 68843141 |
|  | chr2 | 69219299 | 69219334 |
|  | chr2 | 69656797 | 69656832 |
|  | chr2 | 87832147 | 87832179 |
|  | chr2 | 87845343 | 87845375 |
|  | chr2 | 88089932 | 88089962 |
|  | chr2 | 91180429 | 91180462 |
|  | chr2 | 100283441 | 100283476 |
|  | chr2 | 101818690 | 101818727 |
|  | chr2 | 102743564 | 102743604 |
|  | chr2 | 106455459 | 106455493 |
|  | chr2 | 107859855 | 107859893 |
|  | chr2 | 109697848 | 109697882 |
|  | chr2 | 112657874 | 112657907 |
|  | chr2 | 113476743 | 113476785 |
|  | chr2 | 115622990 | 115623025 |
|  | chr2 | 119061954 | 119061985 |
|  | chr2 | 120783600 | 120783634 |
|  | chr2 | 130897486 | 130897520 |
|  | chr2 | 137485476 | 137485510 |
|  | chr2 | 140592340 | 140592373 |
|  | chr2 | 141556217 | 141556263 |
|  | chr2 | 146306647 | 146306685 |
|  | chr2 | 146364013 | 146364048 |
|  | chr2 | 147649196 | 147649231 |
|  | chr2 | 149915166 | 149915201 |
|  | chr2 | 150180503 | 150180538 |
|  | chr2 | 150256266 | 150256301 |
|  | chr2 | 163158155 | 163158190 |
|  | chr2 | 172949738 | 172949774 |
|  | chr3 | 4757803 | 4757838 |
|  | chr3 | 6207327 | 6207361 |
|  | chr3 | 6355025 | 6355060 |
|  | chr3 | 10521800 | 10521835 |
|  | chr3 | 12016673 | 12016708 |
|  | chr3 | 25604077 | 25604109 |
|  | chr3 | 31146347 | 31146389 |
|  | chr3 | 37365454 | 37365492 |
|  | chr3 | 42454707 | 42454741 |
|  | chr3 | 42692695 | 42692730 |
|  | chr3 | 43271277 | 43271311 |
|  | chr3 | 45036019 | 45036054 |
|  | chr3 | 54486921 | 54486954 |
|  | chr3 | 57415775 | 57415809 |
|  | chr3 | 60104344 | 60104377 |
|  | chr3 | 61111255 | 61111290 |
|  | chr3 | 61359475 | 61359510 |
|  | chr3 | 63516814 | 63516848 |
|  | chr3 | 65919027 | 65919065 |
|  | chr3 | 66952282 | 66952317 |
|  | chr3 | 67559107 | 67559141 |
|  | chr3 | 69731847 | 69731882 |
|  | chr3 | 71651123 | 71651157 |
|  | chr3 | 75749999 | 75750033 |
|  | chr3 | 81121903 | 81121939 |
|  | chr3 | 82228370 | 82228421 |
|  | chr3 | 88592014 | 88592052 |
|  | chr3 | 94313037 | 94313075 |
|  | chr3 | 96620929 | 96620962 |
|  | chr3 | 99623200 | 99623240 |
|  | chr3 | 104284308 | 104284341 |
|  | chr3 | 110841033 | 110841068 |
|  | chr3 | 113621693 | 113621739 |
|  | chr3 | 114826378 | 114826409 |
|  | chr3 | 114837142 | 114837176 |
|  | chr3 | 116582072 | 116582105 |
|  | chr3 | 120501490 | 120501526 |
|  | chr3 | 121208993 | 121209028 |
|  | chr3 | 133027712 | 133027746 |
|  | chr3 | 141166050 | 141166085 |
|  | chr3 | 145977753 | 145977788 |
|  | chr4 | 5544883 | 5544918 |
|  | chr4 | 10226916 | 10226951 |
|  | chr4 | 12589035 | 12589074 |
|  | chr4 | 16935936 | 16935969 |
|  | chr4 | 16936840 | 16936873 |
|  | chr4 | 19700859 | 19700894 |
|  | chr4 | 20228805 | 20228851 |
|  | chr4 | 22612945 | 22612979 |
|  | chr4 | 35967074 | 35967113 |
|  | chr4 | 39865687 | 39865732 |
|  | chr4 | 40860299 | 40860336 |
|  | chr4 | 48762520 | 48762557 |
|  | chr4 | 53661577 | 53661612 |
|  | chr4 | 59170378 | 59170414 |
|  | chr4 | 62798910 | 62798948 |
|  | chr4 | 65683158 | 65683196 |
|  | chr4 | 68552938 | 68552972 |
|  | chr4 | 69251596 | 69251630 |
|  | chr4 | 73020983 | 73021015 |
|  | chr4 | 78263704 | 78263739 |
|  | chr4 | 94226042 | 94226077 |
|  | chr4 | 96699351 | 96699386 |
|  | chr4 | 102128049 | 102128081 |
|  | chr4 | 108094408 | 108094443 |
|  | chr4 | 108501068 | 108501103 |
|  | chr4 | 108866092 | 108866127 |
|  | chr4 | 112129512 | 112129546 |
|  | chr4 | 115811454 | 115811492 |
|  | chr4 | 116415851 | 116415897 |
|  | chr4 | 124341902 | 124341937 |
|  | chr4 | 127072034 | 127072068 |
|  | chr4 | 129119520 | 129119555 |
|  | chr4 | 132906764 | 132906817 |
|  | chr4 | 141844854 | 141844891 |
|  | chr4 | 142895679 | 142895713 |
|  | chr4 | 144611249 | 144611287 |
|  | chr4 | 150153017 | 150153056 |
|  | chr4 | 152067341 | 152067373 |
|  | chr5 | 7648932 | 7648964 |
|  | chr5 | 9065149 | 9065184 |
|  | chr5 | 22181577 | 22181612 |
|  | chr5 | 24137792 | 24137827 |
|  | chr5 | 25424796 | 25424830 |
|  | chr5 | 31224831 | 31224865 |
|  | chr5 | 32788031 | 32788066 |
|  | chr5 | 49132019 | 49132053 |
|  | chr5 | 49224373 | 49224406 |
|  | chr5 | 63016847 | 63016881 |
|  | chr5 | 63166302 | 63166336 |
|  | chr5 | 69055266 | 69055310 |
|  | chr5 | 73702702 | 73702736 |
|  | chr5 | 74819926 | 74819961 |
|  | chr5 | 79314259 | 79314293 |
|  | chr5 | 82247127 | 82247161 |
|  | chr5 | 83238358 | 83238401 |
|  | chr5 | 83602521 | 83602556 |
|  | chr5 | 89452755 | 89452798 |
|  | chr5 | 90847983 | 90848024 |
|  | chr5 | 97469255 | 97469289 |
|  | chr5 | 103721413 | 103721451 |
|  | chr5 | 104094495 | 104094530 |
|  | chr5 | 104862114 | 104862149 |
|  | chr5 | 111542734 | 111542769 |
|  | chr5 | 114355946 | 114355979 |
|  | chr5 | 115130114 | 115130148 |
|  | chr5 | 122226244 | 122226278 |
|  | chr5 | 122827049 | 122827083 |
|  | chr5 | 123628201 | 123628257 |
|  | chr5 | 125643596 | 125643631 |
|  | chr5 | 134999974 | 135000017 |
|  | chr5 | 143249363 | 143249407 |
|  | chr5 | 147398094 | 147398126 |
|  | chr5 | 149102379 | 149102417 |
|  | chr6 | 4467849 | 4467884 |
|  | chr6 | 8036546 | 8036581 |
|  | chr6 | 8550336 | 8550382 |
|  | chr6 | 26438671 | 26438714 |
|  | chr6 | 27197171 | 27197206 |
|  | chr6 | 29707750 | 29707783 |
|  | chr6 | 30102860 | 30102894 |
|  | chr6 | 35118299 | 35118333 |
|  | chr6 | 35510392 | 35510430 |
|  | chr6 | 43751365 | 43751400 |
|  | chr6 | 45010429 | 45010464 |
|  | chr6 | 45570938 | 45570967 |
|  | chr6 | 54620687 | 54620733 |
|  | chr6 | 55533525 | 55533560 |
|  | chr6 | 55817141 | 55817176 |
|  | chr6 | 56937031 | 56937071 |
|  | chr6 | 59611178 | 59611212 |
|  | chr6 | 60815265 | 60815298 |
|  | chr6 | 65127989 | 65128024 |
|  | chr6 | 72392336 | 72392370 |
|  | chr6 | 73908558 | 73908591 |
|  | chr6 | 76606412 | 76606445 |
|  | chr6 | 80185467 | 80185499 |
|  | chr6 | 93344650 | 93344684 |
|  | chr6 | 93872756 | 93872798 |
|  | chr6 | 101610790 | 101610828 |
|  | chr6 | 102697076 | 102697111 |
|  | chr6 | 103120336 | 103120373 |
|  | chr6 | 104910198 | 104910232 |
|  | chr6 | 106087802 | 106087837 |
|  | chr6 | 107872074 | 107872114 |
|  | chr6 | 123195674 | 123195713 |
|  | chr6 | 124402071 | 124402109 |
|  | chr6 | 126825660 | 126825694 |
|  | chr6 | 127525100 | 127525138 |
|  | chr6 | 128931545 | 128931578 |
|  | chr6 | 128960310 | 128960348 |
|  | chr6 | 128974595 | 128974628 |
|  | chr6 | 133977567 | 133977606 |
|  | chr6 | 137872856 | 137872890 |
|  | chr6 | 141970908 | 141970942 |
|  | chr7 | 10197149 | 10197183 |
|  | chr7 | 11688834 | 11688869 |
|  | chr7 | 26915635 | 26915671 |
|  | chr7 | 27050795 | 27050831 |
|  | chr7 | 36464457 | 36464491 |
|  | chr7 | 38852401 | 38852435 |
|  | chr7 | 42647613 | 42647647 |
|  | chr7 | 43109074 | 43109108 |
|  | chr7 | 44923508 | 44923542 |
|  | chr7 | 47069409 | 47069443 |
|  | chr7 | 52874936 | 52874970 |
|  | chr7 | 53758908 | 53758943 |
|  | chr7 | 56064419 | 56064460 |
|  | chr7 | 56751518 | 56751550 |
|  | chr7 | 66898807 | 66898843 |
|  | chr7 | 69116560 | 69116595 |
|  | chr7 | 75560347 | 75560383 |
|  | chr7 | 77549595 | 77549630 |
|  | chr7 | 88561061 | 88561095 |
|  | chr7 | 93319660 | 93319695 |
|  | chr7 | 101857791 | 101857826 |
|  | chr7 | 107698887 | 107698921 |
|  | chr7 | 108258838 | 108258872 |
|  | chr7 | 108764036 | 108764070 |
|  | chr7 | 109645183 | 109645218 |
|  | chr7 | 111939358 | 111939392 |
|  | chr7 | 115369956 | 115369991 |
|  | chr7 | 118285269 | 118285303 |
|  | chr7 | 123273482 | 123273516 |
|  | chr7 | 127047183 | 127047218 |
|  | chr7 | 129094448 | 129094483 |
|  | chr7 | 131458075 | 131458107 |
|  | chr7 | 133852170 | 133852204 |
|  | chr7 | 144750050 | 144750085 |
|  | chr8 | 7398830 | 7398864 |
|  | chr8 | 14223649 | 14223680 |
|  | chr8 | 14350199 | 14350232 |
|  | chr8 | 18500724 | 18500758 |
|  | chr8 | 23896635 | 23896673 |
|  | chr8 | 33540156 | 33540190 |
|  | chr8 | 34630673 | 34630708 |
|  | chr8 | 44697893 | 44697926 |
|  | chr8 | 46434620 | 46434654 |
|  | chr8 | 49781049 | 49781088 |
|  | chr8 | 50589388 | 50589423 |
|  | chr8 | 50660510 | 50660544 |
|  | chr8 | 60025280 | 60025313 |
|  | chr8 | 65498891 | 65498925 |
|  | chr8 | 66423844 | 66423877 |
|  | chr8 | 67629050 | 67629088 |
|  | chr8 | 67946662 | 67946698 |
|  | chr8 | 68402662 | 68402696 |
|  | chr8 | 72037642 | 72037699 |
|  | chr8 | 72080088 | 72080145 |
|  | chr8 | 78327497 | 78327535 |
|  | chr8 | 80721088 | 80721122 |
|  | chr8 | 82118863 | 82118895 |
|  | chr8 | 87940332 | 87940367 |
|  | chr8 | 93943726 | 93943760 |
|  | chr8 | 94323818 | 94323850 |
|  | chr8 | 96232956 | 96232991 |
|  | chr8 | 97126548 | 97126581 |
|  | chr8 | 102684558 | 102684591 |
|  | chr8 | 123372290 | 123372321 |
|  | chr9 | 23915334 | 23915367 |
|  | chr9 | 29487957 | 29487989 |
|  | chr9 | 31773310 | 31773348 |
|  | chr9 | 34576991 | 34577026 |
|  | chr9 | 48915394 | 48915425 |
|  | chr9 | 55021298 | 55021331 |
|  | chr9 | 57767412 | 57767447 |
|  | chr9 | 66616163 | 66616230 |
|  | chr9 | 73753683 | 73753718 |
|  | chr9 | 81357742 | 81357779 |
|  | chr9 | 88282660 | 88282695 |
|  | chr9 | 88701898 | 88701933 |
|  | chr9 | 88966723 | 88966758 |
|  | chr9 | 89228372 | 89228405 |
|  | chr9 | 90065811 | 90065846 |
|  | chr9 | 91831216 | 91831248 |
|  | chr9 | 95873409 | 95873444 |
|  | chr9 | 97254139 | 97254174 |
|  | chr9 | 100842805 | 100842836 |
|  | chr9 | 107125905 | 107125937 |
|  | chr9 | 107246168 | 107246203 |
|  | chr9 | 109544734 | 109544777 |
|  | chr9 | 110115774 | 110115809 |
|  | chr9 | 110138736 | 110138770 |
|  | chr9 | 110210086 | 110210118 |
|  | chr9 | 111184759 | 111184794 |
|  | chr10 | 3939353 | 3939389 |
|  | chr10 | 7854843 | 7854878 |
|  | chr10 | 12762041 | 12762079 |
|  | chr10 | 21461018 | 21461053 |
|  | chr10 | 22498476 | 22498511 |
|  | chr10 | 22562963 | 22562998 |
|  | chr10 | 25454969 | 25455004 |
|  | chr10 | 26073107 | 26073145 |
|  | chr10 | 28389557 | 28389592 |
|  | chr10 | 58991660 | 58991697 |
|  | chr10 | 61811070 | 61811105 |
|  | chr10 | 63226988 | 63227028 |
|  | chr10 | 65052766 | 65052812 |
|  | chr10 | 68022264 | 68022298 |
|  | chr10 | 71238849 | 71238893 |
|  | chr10 | 82164875 | 82164910 |
|  | chr10 | 85241045 | 85241077 |
|  | chr10 | 102360440 | 102360481 |
|  | chr10 | 112661949 | 112661984 |
|  | chr10 | 114995285 | 114995338 |
|  | chr10 | 117647039 | 117647074 |
|  | chr10 | 123432566 | 123432618 |
|  | chr10 | 128181959 | 128181996 |
|  | chr11 | 4291520 | 4291555 |
|  | chr11 | 4358815 | 4358850 |
|  | chr11 | 9796933 | 9796967 |
|  | chr11 | 12300499 | 12300538 |
|  | chr11 | 14106272 | 14106307 |
|  | chr11 | 24584979 | 24585013 |
|  | chr11 | 25196378 | 25196413 |
|  | chr11 | 32430128 | 32430163 |
|  | chr11 | 33250098 | 33250135 |
|  | chr11 | 43114456 | 43114489 |
|  | chr11 | 43975184 | 43975215 |
|  | chr11 | 59563320 | 59563353 |
|  | chr11 | 60084848 | 60084883 |
|  | chr11 | 64867214 | 64867248 |
|  | chr11 | 66057793 | 66057828 |
|  | chr11 | 66835547 | 66835579 |
|  | chr11 | 74649000 | 74649035 |
|  | chr11 | 77672549 | 77672583 |
|  | chr11 | 79915319 | 79915360 |
|  | chr11 | 85196011 | 85196049 |
|  | chr11 | 85575781 | 85575816 |
|  | chr11 | 89395891 | 89395932 |
|  | chr11 | 101078077 | 101078111 |
|  | chr11 | 105171106 | 105171141 |
|  | chr11 | 108621952 | 108621986 |
|  | chr11 | 110783661 | 110783696 |
|  | chr11 | 110951877 | 110951913 |
|  | chr11 | 112192522 | 112192556 |
|  | chr11 | 116041584 | 116041621 |
|  | chr11 | 121346587 | 121346622 |
|  | chr12 | 10490157 | 10490195 |
|  | chr12 | 13108467 | 13108503 |
|  | chr12 | 16947945 | 16947979 |
|  | chr12 | 29011601 | 29011637 |
|  | chr12 | 32617660 | 32617695 |
|  | chr12 | 33370125 | 33370164 |
|  | chr12 | 35409107 | 35409142 |
|  | chr12 | 35717888 | 35717923 |
|  | chr12 | 37001887 | 37001921 |
|  | chr12 | 37956939 | 37956974 |
|  | chr12 | 49985955 | 49985990 |
|  | chr12 | 54221766 | 54221799 |
|  | chr12 | 59992644 | 59992682 |
|  | chr12 | 60926765 | 60926799 |
|  | chr12 | 65498209 | 65498240 |
|  | chr12 | 66422339 | 66422383 |
|  | chr12 | 71006729 | 71006768 |
|  | chr12 | 71302143 | 71302178 |
|  | chr12 | 72082353 | 72082387 |
|  | chr12 | 85905133 | 85905167 |
|  | chr12 | 92923160 | 92923201 |
|  | chr12 | 101649371 | 101649411 |
|  | chr12 | 110997772 | 110997807 |
|  | chr12 | 119204454 | 119204497 |
|  | chr13 | 4865166 | 4865198 |
|  | chr13 | 6976900 | 6976939 |
|  | chr13 | 19881295 | 19881329 |
|  | chr13 | 20586907 | 20586942 |
|  | chr13 | 20660547 | 20660581 |
|  | chr13 | 23139367 | 23139399 |
|  | chr13 | 30466463 | 30466500 |
|  | chr13 | 33498735 | 33498770 |
|  | chr13 | 34402089 | 34402127 |
|  | chr13 | 49123838 | 49123872 |
|  | chr13 | 58137666 | 58137708 |
|  | chr13 | 61326718 | 61326752 |
|  | chr13 | 66730170 | 66730209 |
|  | chr13 | 80849098 | 80849141 |
|  | chr13 | 82675970 | 82676004 |
|  | chr13 | 82998183 | 82998220 |
|  | chr13 | 83034504 | 83034541 |
|  | chr13 | 91066969 | 91067004 |
|  | chr13 | 92811803 | 92811847 |
|  | chr13 | 99481314 | 99481349 |
|  | chr13 | 100538219 | 100538256 |
|  | chr13 | 103610228 | 103610262 |
|  | chr13 | 106260623 | 106260656 |
|  | chr13 | 109001738 | 109001774 |
|  | chr13 | 109307037 | 109307073 |
|  | chr13 | 113516885 | 113516923 |
|  | chr13 | 119437561 | 119437595 |
|  | chr13 | 119817299 | 119817338 |
|  | chr14 | 18200713 | 18200750 |
|  | chr14 | 20980681 | 20980718 |
|  | chr14 | 21249289 | 21249323 |
|  | chr14 | 24412368 | 24412402 |
|  | chr14 | 35394389 | 35394422 |
|  | chr14 | 45214033 | 45214070 |
|  | chr14 | 47134981 | 47135019 |
|  | chr14 | 47372520 | 47372554 |
|  | chr14 | 48712516 | 48712551 |
|  | chr14 | 52383251 | 52383285 |
|  | chr14 | 53863274 | 53863314 |
|  | chr14 | 55683870 | 55683917 |
|  | chr14 | 60572792 | 60572826 |
|  | chr14 | 64886453 | 64886488 |
|  | chr14 | 67522409 | 67522443 |
|  | chr14 | 67875810 | 67875845 |
|  | chr14 | 70299949 | 70299991 |
|  | chr14 | 73165263 | 73165298 |
|  | chr14 | 75663383 | 75663417 |
|  | chr14 | 77597332 | 77597367 |
|  | chr14 | 80852050 | 80852084 |
|  | chr14 | 87989482 | 87989517 |
|  | chr14 | 88758223 | 88758258 |
|  | chr14 | 96129788 | 96129823 |
|  | chr14 | 96949236 | 96949271 |
|  | chr14 | 99352056 | 99352169 |
|  | chr14 | 101623804 | 101623848 |
|  | chr14 | 105510965 | 105510999 |
|  | chr14 | 110579717 | 110579752 |
|  | chr14 | 112919070 | 112919105 |
|  | chr14 | 113079296 | 113079329 |
|  | chr14 | 116346114 | 116346149 |
|  | chr14 | 116805584 | 116805628 |
|  | chr14 | 117086021 | 117086062 |
|  | chr14 | 121397253 | 121397287 |
|  | chr14 | 121988366 | 121988401 |
|  | chr15 | 3417268 | 3417312 |
|  | chr15 | 5056292 | 5056328 |
|  | chr15 | 5088929 | 5088969 |
|  | chr15 | 7216337 | 7216379 |
|  | chr15 | 17001067 | 17001103 |
|  | chr15 | 18698298 | 18698333 |
|  | chr15 | 19677641 | 19677678 |
|  | chr15 | 24391040 | 24391077 |
|  | chr15 | 26892051 | 26892084 |
|  | chr15 | 28913719 | 28913754 |
|  | chr15 | 34411598 | 34411632 |
|  | chr15 | 40415257 | 40415294 |
|  | chr15 | 42337456 | 42337490 |
|  | chr15 | 45158234 | 45158270 |
|  | chr15 | 48578825 | 48578857 |
|  | chr15 | 49981421 | 49981454 |
|  | chr15 | 60949311 | 60949345 |
|  | chr15 | 68656756 | 68656796 |
|  | chr15 | 81135131 | 81135160 |
|  | chr15 | 83212811 | 83212846 |
|  | chr15 | 85946127 | 85946161 |
|  | chr15 | 93433364 | 93433397 |
|  | chr16 | 18360773 | 18360807 |
|  | chr16 | 18708044 | 18708080 |
|  | chr16 | 19533287 | 19533323 |
|  | chr16 | 21407519 | 21407554 |
|  | chr16 | 26480594 | 26480628 |
|  | chr16 | 26709772 | 26709806 |
|  | chr16 | 26831590 | 26831624 |
|  | chr16 | 32042439 | 32042472 |
|  | chr16 | 32606814 | 32606847 |
|  | chr16 | 33691899 | 33691945 |
|  | chr16 | 36409958 | 36409993 |
|  | chr16 | 37047803 | 37047833 |
|  | chr16 | 38152967 | 38153002 |
|  | chr16 | 38402014 | 38402051 |
|  | chr16 | 40696257 | 40696292 |
|  | chr16 | 41742652 | 41742684 |
|  | chr16 | 43357962 | 43357993 |
|  | chr16 | 43609044 | 43609077 |
|  | chr16 | 49378250 | 49378281 |
|  | chr16 | 50082625 | 50082660 |
|  | chr16 | 50324701 | 50324738 |
|  | chr16 | 51371388 | 51371422 |
|  | chr16 | 52407822 | 52407861 |
|  | chr16 | 52688204 | 52688246 |
|  | chr16 | 57704753 | 57704784 |
|  | chr16 | 60088517 | 60088551 |
|  | chr16 | 70313073 | 70313110 |
|  | chr16 | 74870694 | 74870740 |
|  | chr16 | 77927650 | 77927683 |
|  | chr16 | 89159070 | 89159105 |
|  | chr16 | 89770790 | 89770827 |
|  | chr16 | 92890095 | 92890130 |
|  | chr16 | 93011477 | 93011510 |
|  | chr17 | 6929319 | 6929353 |
|  | chr17 | 7959909 | 7959943 |
|  | chr17 | 12647520 | 12647554 |
|  | chr17 | 24726512 | 24726547 |
|  | chr17 | 32707475 | 32707512 |
|  | chr17 | 36897036 | 36897069 |
|  | chr17 | 42121610 | 42121640 |
|  | chr17 | 47704433 | 47704468 |
|  | chr17 | 47811887 | 47811921 |
|  | chr17 | 51443435 | 51443480 |
|  | chr17 | 55228082 | 55228116 |
|  | chr17 | 57268536 | 57268577 |
|  | chr17 | 64984022 | 64984055 |
|  | chr17 | 72678161 | 72678202 |
|  | chr17 | 75828209 | 75828243 |
|  | chr17 | 76918023 | 76918055 |
|  | chr17 | 83807845 | 83807880 |
|  | chr17 | 87106681 | 87106716 |
|  | chr17 | 92361297 | 92361331 |
|  | chr17 | 93722940 | 93722975 |
|  | chr17 | 93830231 | 93830272 |
|  | chr18 | 7191417 | 7191450 |
|  | chr18 | 7345979 | 7346014 |
|  | chr18 | 10162600 | 10162632 |
|  | chr18 | 10956233 | 10956268 |
|  | chr18 | 14593807 | 14593841 |
|  | chr18 | 15175413 | 15175457 |
|  | chr18 | 16503406 | 16503439 |
|  | chr18 | 16535201 | 16535236 |
|  | chr18 | 18911382 | 18911413 |
|  | chr18 | 20803148 | 20803183 |
|  | chr18 | 25250240 | 25250276 |
|  | chr18 | 30049830 | 30049865 |
|  | chr18 | 31921262 | 31921297 |
|  | chr18 | 32915486 | 32915526 |
|  | chr18 | 34703821 | 34703861 |
|  | chr18 | 43186578 | 43186613 |
|  | chr18 | 49766558 | 49766593 |
|  | chr18 | 51597451 | 51597490 |
|  | chr18 | 54812778 | 54812813 |
|  | chr18 | 57681826 | 57681861 |
|  | chr18 | 62800174 | 62800208 |
|  | chr18 | 63569742 | 63569779 |
|  | chr18 | 69368038 | 69368072 |
|  | chr18 | 72340162 | 72340199 |
|  | chr18 | 73382834 | 73382869 |
|  | chr18 | 76833244 | 76833283 |
|  | chr18 | 85559153 | 85559187 |
|  | chr19 | 3527524 | 3527559 |
|  | chr19 | 3744612 | 3744646 |
|  | chr19 | 8671340 | 8671381 |
|  | chr19 | 16787556 | 16787591 |
|  | chr19 | 18086149 | 18086184 |
|  | chr19 | 19451550 | 19451586 |
|  | chr19 | 23056333 | 23056365 |
|  | chr19 | 24300673 | 24300704 |
|  | chr19 | 29004571 | 29004606 |
|  | chr19 | 30345796 | 30345831 |
|  | chr19 | 32047593 | 32047628 |
|  | chr19 | 34063419 | 34063454 |
|  | chr19 | 36282652 | 36282687 |
|  | chr19 | 39054132 | 39054167 |
|  | chr19 | 40673284 | 40673319 |
|  | chr19 | 42521173 | 42521207 |
|  | chr19 | 49085163 | 49085198 |
|  | chr19 | 53715031 | 53715065 |
|  | chr19 | 53950011 | 53950046 |
|  | chr19 | 56180888 | 56180923 |
|  | chrX | 9336618 | 9336650 |
|  | chrX | 9516267 | 9516299 |
|  | chrX | 10616351 | 10616386 |
|  | chrX | 19910958 | 19910993 |
|  | chrX | 21353583 | 21353617 |
|  | chrX | 23718737 | 23718771 |
|  | chrX | 36612481 | 36612516 |
|  | chrX | 36741250 | 36741284 |
|  | chrX | 40229854 | 40229890 |
|  | chrX | 40769054 | 40769090 |
|  | chrX | 42356636 | 42356676 |
|  | chrX | 42474771 | 42474806 |
|  | chrX | 42666870 | 42666905 |
|  | chrX | 43240207 | 43240241 |
|  | chrX | 46156080 | 46156114 |
|  | chrX | 52815276 | 52815329 |
|  | chrX | 59854680 | 59854714 |
|  | chrX | 64797082 | 64797113 |
|  | chrX | 67190349 | 67190384 |
|  | chrX | 67571698 | 67571735 |
|  | chrX | 69910689 | 69910722 |
|  | chrX | 73404022 | 73404057 |
|  | chrX | 77297267 | 77297302 |
|  | chrX | 77880310 | 77880353 |
|  | chrX | 81772950 | 81772985 |
|  | chrX | 84938787 | 84938816 |
|  | chrX | 89002024 | 89002059 |
|  | chrX | 94660519 | 94660554 |
|  | chrX | 98972459 | 98972496 |
|  | chrX | 100251734 | 100251768 |
|  | chrX | 101386233 | 101386268 |
|  | chrX | 104831716 | 104831751 |
|  | chrX | 106573954 | 106573988 |
|  | chrX | 108749643 | 108749687 |
|  | chrX | 114825085 | 114825124 |
|  | chrX | 115545449 | 115545483 |
|  | chrX | 116871820 | 116871855 |
|  | chrX | 119298776 | 119298810 |
|  | chrX | 121602408 | 121602442 |
|  | chrX | 134544974 | 134545009 |
|  | chrX | 144770616 | 144770651 |
|  | chrX | 146867817 | 146867852 |
|  | chrX | 151982235 | 151982270 |
|  | chrX | 152188843 | 152188887 |
|  | chrX | 164961055 | 164961095 |
|  | chrX | 167137479 | 167137513 |
| ***Loxodonta africana*** | contig_180 | 72502 | 72537 |
|  | contig_2836 | 13003 | 13037 |
|  | contig_3700 | 81530 | 81563 |
|  | contig_5082 | 29490 | 29522 |
|  | contig_8198 | 31651 | 31686 |
|  | contig_8406 | 12168 | 12202 |
|  | contig_10728 | 46015 | 46046 |
|  | contig_12877 | 281856 | 281890 |
|  | contig_15514 | 3218 | 3250 |
|  | contig_17349 | 41784 | 41819 |
|  | contig_17962 | 4562 | 4597 |
|  | contig_22773 | 22918 | 22952 |
|  | contig_24816 | 219156 | 219191 |
|  | contig_27075 | 3607 | 3640 |
|  | contig_28570 | 29385 | 29419 |
|  | contig_30986 | 27177 | 27211 |
|  | contig_31756 | 199277 | 199307 |
|  | contig_31831 | 81277 | 81312 |
|  | contig_32124 | 32718 | 32753 |
|  | contig_36520 | 46384 | 46418 |
|  | contig_39800 | 17378 | 17415 |
|  | contig_41137 | 90224 | 90259 |
|  | contig_42742 | 1189 | 1223 |
|  | contig_43399 | 72888 | 72923 |
|  | contig_52456 | 26180 | 26215 |
|  | contig_54492 | 2925 | 2965 |
|  | contig_56474 | 1777 | 1832 |
|  | contig_58575 | 37164 | 37197 |
|  | contig_60109 | 5406 | 5440 |
|  | contig_61755 | 26513 | 26592 |
|  | contig_62055 | 47875 | 47907 |
|  | contig_62608 | 25138 | 25173 |
|  | contig_64296 | 27113 | 27152 |
|  | contig_67357 | 23713 | 23746 |
|  | contig_67842 | 79934 | 79968 |
|  | contig_71090 | 576 | 610 |
|  | contig_72116 | 191774 | 191809 |
|  | contig_74048 | 60552 | 60587 |
|  | contig_75999 | 34315 | 34351 |
|  | contig_78635 | 62680 | 62717 |
|  | contig_81600 | 33475 | 33509 |
|  | contig_84627 | 12357 | 12394 |
| ***Choloepus hoffmanni*** | GeneScaffold_413 | 208483 | 208517 |
|  | GeneScaffold_811 | 51249 | 51279 |
|  | GeneScaffold_2156 | 54617 | 54651 |
|  | GeneScaffold_2255 | 5339 | 5375 |
|  | GeneScaffold_2438 | 26510 | 26543 |
|  | GeneScaffold_2791 | 47681 | 47714 |
|  | GeneScaffold_3033 | 176219 | 176253 |
|  | GeneScaffold_3415 | 17228 | 17261 |
|  | GeneScaffold_3469 | 28812 | 28844 |
|  | GeneScaffold_4324 | 8535 | 8571 |
|  | GeneScaffold_4407 | 26313 | 26347 |
|  | GeneScaffold_4535 | 12881 | 12917 |
|  | GeneScaffold_5552 | 31492 | 31525 |
|  | GeneScaffold_5573 | 29203 | 29241 |
|  | GeneScaffold_6340 | 197421 | 197456 |
|  | GeneScaffold_6581 | 8747 | 8784 |
|  | GeneScaffold_6976 | 21390 | 21424 |
|  | GeneScaffold_7084 | 69415 | 69448 |
|  | GeneScaffold_7844 | 35274 | 35308 |
|  | scaffold_401 | 39138 | 39173 |
|  | scaffold_1335 | 18677 | 18711 |
|  | scaffold_2609 | 18862 | 18892 |
|  | scaffold_2811 | 7005 | 7040 |
|  | scaffold_3452 | 34723 | 34758 |
|  | scaffold_4836 | 15188 | 15223 |
|  | scaffold_5008 | 17754 | 17788 |
|  | scaffold_6778 | 13031 | 13068 |
|  | scaffold_7336 | 10306 | 10359 |
|  | scaffold_7652 | 25375 | 25409 |
|  | scaffold_7976 | 6562 | 6595 |
|  | scaffold_10532 | 11351 | 11385 |
|  | scaffold_12992 | 19232 | 19265 |
|  | scaffold_13653 | 15639 | 15674 |
|  | scaffold_13793 | 11299 | 11338 |
|  | scaffold_14113 | 9830 | 9864 |
|  | scaffold_14377 | 6464 | 6498 |
|  | scaffold_15392 | 18096 | 18135 |
|  | scaffold_15605 | 22386 | 22421 |
|  | scaffold_18831 | 108 | 143 |
|  | scaffold_29044 | 2512 | 2547 |
|  | scaffold_34973 | 7109 | 7149 |
|  | scaffold_47405 | 10330 | 10365 |
|  | scaffold_47521 | 9871 | 9901 |
|  | scaffold_48237 | 4116 | 4150 |
|  | scaffold_49821 | 10541 | 10576 |
|  | scaffold_50943 | 1527 | 1561 |
|  | scaffold_52211 | 1182 | 1221 |
|  | scaffold_56292 | 3805 | 3840 |
|  | scaffold_56411 | 12004 | 12045 |
|  | scaffold_57598 | 7755 | 7794 |
|  | scaffold_58054 | 829 | 863 |
|  | scaffold_60511 | 9435 | 9469 |
|  | scaffold_68090 | 1275 | 1311 |
|  | scaffold_73697 | 900 | 940 |
|  | scaffold_78334 | 2877 | 2912 |
|  | scaffold_85855 | 2681 | 2718 |
|  | scaffold_94426 | 1939 | 1969 |
|  | scaffold_106171 | 3607 | 3639 |
|  | scaffold_116510 | 2210 | 2244 |
|  | scaffold_117178 | 110 | 144 |
|  | scaffold_121292 | 2364 | 2401 |
|  | scaffold_124296 | 3564 | 3605 |
|  | scaffold_130179 | 6500 | 6533 |
|  | scaffold_149221 | 711 | 744 |
|  | scaffold_160047 | 3684 | 3714 |
|  | scaffold_160905 | 2752 | 2786 |
|  | scaffold_164830 | 389 | 426 |
|  | scaffold_164975 | 4756 | 4790 |
|  | scaffold_166585 | 3998 | 4032 |
|  | scaffold_178340 | 183 | 218 |
|  | scaffold_187576 | 2642 | 2679 |
|  | scaffold_188756 | 3710 | 3743 |
|  | scaffold_224305 | 481 | 515 |
|  | scaffold_227952 | 896 | 926 |
|  | scaffold_245828 | 315 | 353 |
|  | scaffold_254534 | 1122 | 1156 |
|  | scaffold_267101 | 1332 | 1369 |
|  | scaffold_310128 | 1102 | 1134 |
|  | scaffold_312873 | 1275 | 1309 |
|  | scaffold_323380 | 688 | 731 |
|  | scaffold_377546 | 265 | 298 |
|  | scaffold_438278 | 687 | 727 |
| ***Monodelphis domestica*** | chr1.1-5000000 | 4246582 | 4246620 |
|  | chr1.10000001-15000000 | 315851 | 315897 |
|  | chr1.110000001-115000000 | 3657235 | 3657274 |
|  | chr1.115000001-120000000 | 4028585 | 4028617 |
|  | chr1.135000001-140000000 | 1932389 | 1932428 |
|  | chr1.135000001-140000000 | 2754119 | 2754159 |
|  | chr1.15000001-20000000 | 664086 | 664120 |
|  | chr1.160000001-165000000 | 4658347 | 4658386 |
|  | chr1.165000001-170000000 | 10314 | 10347 |
|  | chr1.180000001-185000000 | 2084447 | 2084493 |
|  | chr1.180000001-185000000 | 3623368 | 3623402 |
|  | chr1.185000001-190000000 | 3242274 | 3242307 |
|  | chr1.190000001-195000000 | 380006 | 380043 |
|  | chr1.200000001-205000000 | 4837161 | 4837201 |
|  | chr1.220000001-225000000 | 98030 | 98064 |
|  | chr1.220000001-225000000 | 541205 | 541247 |
|  | chr1.230000001-235000000 | 1180871 | 1180903 |
|  | chr1.240000001-245000000 | 923933 | 923968 |
|  | chr1.240000001-245000000 | 4423319 | 4423354 |
|  | chr1.245000001-250000000 | 3056957 | 3056991 |
|  | chr1.25000001-30000000 | 3312063 | 3312093 |
|  | chr1.265000001-270000000 | 2027025 | 2027059 |
|  | chr1.270000001-275000000 | 212826 | 212871 |
|  | chr1.270000001-275000000 | 2149211 | 2149251 |
|  | chr1.270000001-275000000 | 2224092 | 2224126 |
|  | chr1.270000001-275000000 | 3466813 | 3466855 |
|  | chr1.275000001-280000000 | 3164976 | 3165010 |
|  | chr1.275000001-280000000 | 3352321 | 3352362 |
|  | chr1.275000001-280000000 | 4004214 | 4004247 |
|  | chr1.300000001-305000000 | 2677077 | 2677112 |
|  | chr1.30000001-35000000 | 3868369 | 3868403 |
|  | chr1.315000001-320000000 | 4510805 | 4510844 |
|  | chr1.325000001-330000000 | 4065202 | 4065234 |
|  | chr1.350000001-355000000 | 3796278 | 3796313 |
|  | chr1.35000001-40000000 | 173105 | 173149 |
|  | chr1.35000001-40000000 | 174380 | 174424 |
|  | chr1.375000001-380000000 | 274682 | 274717 |
|  | chr1.385000001-390000000 | 2015239 | 2015277 |
|  | chr1.395000001-400000000 | 1472415 | 1472450 |
|  | chr1.395000001-400000000 | 4058255 | 4058290 |
|  | chr1.40000001-45000000 | 2307818 | 2307853 |
|  | chr1.415000001-420000000 | 960852 | 960887 |
|  | chr1.425000001-430000000 | 1188606 | 1188641 |
|  | chr1.455000001-460000000 | 1072023 | 1072057 |
|  | chr1.455000001-460000000 | 1554753 | 1554786 |
|  | chr1.515000001-520000000 | 3955160 | 3955194 |
|  | chr1.540000001-545000000 | 2416472 | 2416506 |
|  | chr1.540000001-545000000 | 4632531 | 4632563 |
|  | chr1.550000001-555000000 | 2742145 | 2742179 |
|  | chr1.55000001-60000000 | 3154425 | 3154460 |
|  | chr1.560000001-565000000 | 2327298 | 2327333 |
|  | chr1.570000001-575000000 | 2872349 | 2872384 |
|  | chr1.590000001-595000000 | 3457120 | 3457154 |
|  | chr1.600000001-605000000 | 4216624 | 4216662 |
|  | chr1.615000001-620000000 | 4989544 | 4989578 |
|  | chr1.620000001-625000000 | 2662789 | 2662824 |
|  | chr1.645000001-650000000 | 1222341 | 1222378 |
|  | chr1.655000001-660000000 | 90291 | 90326 |
|  | chr1.665000001-670000000 | 2198158 | 2198188 |
|  | chr1.665000001-670000000 | 3021637 | 3021672 |
|  | chr1.675000001-680000000 | 4973592 | 4973622 |
|  | chr1.685000001-690000000 | 501140 | 501181 |
|  | chr1.70000001-75000000 | 3019910 | 3019950 |
|  | chr1.720000001-725000000 | 167937 | 167975 |
|  | chr1.720000001-725000000 | 398153 | 398187 |
|  | chr1.730000001-735000000 | 614254 | 614294 |
|  | chr1.80000001-85000000 | 1637341 | 1637378 |
|  | chr1.80000001-85000000 | 3046198 | 3046237 |
|  | chr1.80000001-85000000 | 4723958 | 4724001 |
|  | chr2.10000001-15000000 | 2540252 | 2540285 |
|  | chr2.110000001-115000000 | 1683808 | 1683845 |
|  | chr2.110000001-115000000 | 1686121 | 1686158 |
|  | chr2.110000001-115000000 | 2972892 | 2972927 |
|  | chr2.115000001-120000000 | 4561927 | 4561961 |
|  | chr2.120000001-125000000 | 849264 | 849297 |
|  | chr2.120000001-125000000 | 2864996 | 2865029 |
|  | chr2.120000001-125000000 | 4275414 | 4275454 |
|  | chr2.135000001-140000000 | 2065493 | 2065528 |
|  | chr2.135000001-140000000 | 3727062 | 3727095 |
|  | chr2.135000001-140000000 | 4301156 | 4301194 |
|  | chr2.140000001-145000000 | 4653421 | 4653455 |
|  | chr2.150000001-155000000 | 1227445 | 1227483 |
|  | chr2.150000001-155000000 | 4956378 | 4956410 |
|  | chr2.155000001-160000000 | 3752194 | 3752229 |
|  | chr2.160000001-165000000 | 259563 | 259600 |
|  | chr2.165000001-170000000 | 1029330 | 1029368 |
|  | chr2.195000001-200000000 | 2130592 | 2130635 |
|  | chr2.195000001-200000000 | 2791538 | 2791572 |
|  | chr2.235000001-240000000 | 193492 | 193527 |
|  | chr2.245000001-250000000 | 603931 | 603965 |
|  | chr2.265000001-270000000 | 702349 | 702386 |
|  | chr2.295000001-300000000 | 1627048 | 1627088 |
|  | chr2.300000001-305000000 | 2214329 | 2214363 |
|  | chr2.300000001-305000000 | 4928264 | 4928294 |
|  | chr2.30000001-35000000 | 2941698 | 2941738 |
|  | chr2.305000001-310000000 | 203597 | 203636 |
|  | chr2.315000001-320000000 | 865578 | 865613 |
|  | chr2.340000001-345000000 | 3865962 | 3865995 |
|  | chr2.345000001-350000000 | 2881406 | 2881445 |
|  | chr2.35000001-40000000 | 2620557 | 2620596 |
|  | chr2.355000001-360000000 | 1559856 | 1559899 |
|  | chr2.365000001-370000000 | 2324983 | 2325023 |
|  | chr2.370000001-375000000 | 553588 | 553622 |
|  | chr2.375000001-380000000 | 1499213 | 1499245 |
|  | chr2.380000001-385000000 | 1442480 | 1442517 |
|  | chr2.385000001-390000000 | 1832994 | 1833032 |
|  | chr2.420000001-425000000 | 4592446 | 4592483 |
|  | chr2.425000001-430000000 | 2936539 | 2936574 |
|  | chr2.440000001-445000000 | 1757675 | 1757710 |
|  | chr2.440000001-445000000 | 2372551 | 2372585 |
|  | chr2.460000001-465000000 | 504875 | 504912 |
|  | chr2.475000001-480000000 | 3050901 | 3050935 |
|  | chr2.485000001-490000000 | 1841590 | 1841624 |
|  | chr2.485000001-490000000 | 4389516 | 4389556 |
|  | chr2.490000001-495000000 | 3839184 | 3839219 |
|  | chr2.5000001-10000000 | 784649 | 784682 |
|  | chr2.520000001-525000000 | 3416292 | 3416340 |
|  | chr2.70000001-75000000 | 3669013 | 3669047 |
|  | chr2.85000001-90000000 | 4728761 | 4728797 |
|  | chr2.90000001-95000000 | 2748492 | 2748526 |
|  | chr2.90000001-95000000 | 3369510 | 3369541 |
|  | chr3.100000001-105000000 | 38627 | 38667 |
|  | chr3.10000001-15000000 | 3775617 | 3775656 |
|  | chr3.105000001-110000000 | 243793 | 243828 |
|  | chr3.105000001-110000000 | 2304736 | 2304780 |
|  | chr3.115000001-120000000 | 786272 | 786306 |
|  | chr3.135000001-140000000 | 1297172 | 1297206 |
|  | chr3.140000001-145000000 | 3474469 | 3474511 |
|  | chr3.145000001-150000000 | 1418379 | 1418412 |
|  | chr3.145000001-150000000 | 3394214 | 3394244 |
|  | chr3.160000001-165000000 | 1078293 | 1078334 |
|  | chr3.160000001-165000000 | 3866123 | 3866165 |
|  | chr3.165000001-170000000 | 627730 | 627767 |
|  | chr3.210000001-215000000 | 1045670 | 1045705 |
|  | chr3.230000001-235000000 | 3935651 | 3935690 |
|  | chr3.240000001-245000000 | 3242365 | 3242399 |
|  | chr3.245000001-250000000 | 2717275 | 2717314 |
|  | chr3.275000001-280000000 | 1330957 | 1330995 |
|  | chr3.285000001-290000000 | 4310082 | 4310121 |
|  | chr3.290000001-295000000 | 4426657 | 4426694 |
|  | chr3.295000001-300000000 | 4075225 | 4075259 |
|  | chr3.30000001-35000000 | 867054 | 867090 |
|  | chr3.320000001-325000000 | 2720528 | 2720567 |
|  | chr3.325000001-330000000 | 3280307 | 3280344 |
|  | chr3.325000001-330000000 | 4338227 | 4338261 |
|  | chr3.335000001-340000000 | 5603 | 5639 |
|  | chr3.335000001-340000000 | 4088045 | 4088083 |
|  | chr3.350000001-355000000 | 4787505 | 4787539 |
|  | chr3.370000001-375000000 | 3266455 | 3266488 |
|  | chr3.380000001-385000000 | 2594610 | 2594645 |
|  | chr3.390000001-395000000 | 94942 | 94975 |
|  | chr3.40000001-45000000 | 4373797 | 4373837 |
|  | chr3.415000001-420000000 | 3470836 | 3470875 |
|  | chr3.420000001-425000000 | 3294598 | 3294630 |
|  | chr3.430000001-435000000 | 4363025 | 4363057 |
|  | chr3.45000001-50000000 | 3492043 | 3492077 |
|  | chr3.460000001-465000000 | 661868 | 661904 |
|  | chr3.485000001-490000000 | 2743885 | 2743934 |
|  | chr3.485000001-490000000 | 4968105 | 4968145 |
|  | chr3.490000001-495000000 | 2529812 | 2529842 |
|  | chr3.495000001-500000000 | 1761015 | 1761049 |
|  | chr3.50000001-55000000 | 677359 | 677392 |
|  | chr3.505000001-510000000 | 1236098 | 1236143 |
|  | chr3.515000001-520000000 | 1242673 | 1242707 |
|  | chr3.65000001-70000000 | 259554 | 259589 |
|  | chr3.70000001-75000000 | 4459482 | 4459521 |
|  | chr3.90000001-95000000 | 4987544 | 4987584 |
|  | chr3.95000001-100000000 | 3110386 | 3110421 |
|  | chr4.1-5000000 | 491703 | 491738 |
|  | chr4.145000001-150000000 | 227982 | 228013 |
|  | chr4.155000001-160000000 | 1562978 | 1563013 |
|  | chr4.180000001-185000000 | 2754085 | 2754122 |
|  | chr4.185000001-190000000 | 1233437 | 1233475 |
|  | chr4.185000001-190000000 | 4106291 | 4106328 |
|  | chr4.190000001-195000000 | 472873 | 472914 |
|  | chr4.190000001-195000000 | 1203832 | 1203865 |
|  | chr4.190000001-195000000 | 4473955 | 4473991 |
|  | chr4.200000001-205000000 | 2829770 | 2829802 |
|  | chr4.215000001-220000000 | 1247562 | 1247601 |
|  | chr4.240000001-245000000 | 2109197 | 2109232 |
|  | chr4.245000001-250000000 | 3821631 | 3821666 |
|  | chr4.250000001-255000000 | 304130 | 304167 |
|  | chr4.250000001-255000000 | 3324717 | 3324758 |
|  | chr4.25000001-30000000 | 2446548 | 2446600 |
|  | chr4.255000001-260000000 | 2915688 | 2915720 |
|  | chr4.30000001-35000000 | 1256093 | 1256130 |
|  | chr4.315000001-320000000 | 3544942 | 3544974 |
|  | chr4.320000001-325000000 | 1835872 | 1835902 |
|  | chr4.320000001-325000000 | 3449986 | 3450023 |
|  | chr4.320000001-325000000 | 3937172 | 3937209 |
|  | chr4.325000001-330000000 | 617920 | 617958 |
|  | chr4.335000001-340000000 | 4551870 | 4551909 |
|  | chr4.370000001-375000000 | 1526867 | 1526911 |
|  | chr4.395000001-400000000 | 1805658 | 1805709 |
|  | chr4.400000001-405000000 | 2077692 | 2077730 |
|  | chr4.405000001-410000000 | 4469146 | 4469187 |
|  | chr4.410000001-415000000 | 1297569 | 1297604 |
|  | chr4.425000001-430000000 | 4337662 | 4337697 |
|  | chr4.5000001-10000000 | 4804470 | 4804503 |
|  | chr4.55000001-60000000 | 2460156 | 2460202 |
|  | chr4.65000001-70000000 | 2386047 | 2386082 |
|  | chr4.65000001-70000000 | 3563459 | 3563494 |
|  | chr4.70000001-75000000 | 2801432 | 2801466 |
|  | chr4.90000001-95000000 | 150362 | 150411 |
|  | chr5.1-5000000 | 2653973 | 2654006 |
|  | chr5.100000001-105000000 | 4867136 | 4867175 |
|  | chr5.150000001-155000000 | 4750383 | 4750423 |
|  | chr5.150000001-155000000 | 4844485 | 4844518 |
|  | chr5.155000001-160000000 | 120721 | 120756 |
|  | chr5.155000001-160000000 | 2134093 | 2134130 |
|  | chr5.160000001-165000000 | 4449311 | 4449345 |
|  | chr5.170000001-175000000 | 3186440 | 3186474 |
|  | chr5.180000001-185000000 | 3789042 | 3789079 |
|  | chr5.200000001-205000000 | 2246379 | 2246413 |
|  | chr5.200000001-205000000 | 4892389 | 4892425 |
|  | chr5.210000001-215000000 | 124780 | 124823 |
|  | chr5.260000001-265000000 | 1107278 | 1107308 |
|  | chr5.285000001-290000000 | 513137 | 513172 |
|  | chr5.285000001-290000000 | 676779 | 676814 |
|  | chr5.290000001-295000000 | 4183409 | 4183448 |
|  | chr5.300000001-304825324 | 893201 | 893238 |
|  | chr5.300000001-304825324 | 2161845 | 2161889 |
|  | chr5.45000001-50000000 | 609663 | 609702 |
|  | chr5.50000001-55000000 | 2222250 | 2222290 |
|  | chr6.10000001-15000000 | 3503160 | 3503195 |
|  | chr6.120000001-125000000 | 3611099 | 3611136 |
|  | chr6.125000001-130000000 | 1952561 | 1952594 |
|  | chr6.130000001-135000000 | 330074 | 330105 |
|  | chr6.135000001-140000000 | 2925881 | 2925916 |
|  | chr6.140000001-145000000 | 2255373 | 2255418 |
|  | chr6.145000001-150000000 | 1806824 | 1806854 |
|  | chr6.150000001-155000000 | 755508 | 755543 |
|  | chr6.15000001-20000000 | 1327970 | 1328005 |
|  | chr6.165000001-170000000 | 4241113 | 4241148 |
|  | chr6.180000001-185000000 | 645722 | 645763 |
|  | chr6.185000001-190000000 | 1627675 | 1627714 |
|  | chr6.190000001-195000000 | 4812967 | 4813001 |
|  | chr6.195000001-200000000 | 243546 | 243578 |
|  | chr6.195000001-200000000 | 1131174 | 1131206 |
|  | chr6.195000001-200000000 | 1946309 | 1946342 |
|  | chr6.225000001-230000000 | 3754952 | 3754984 |
|  | chr6.235000001-240000000 | 736090 | 736122 |
|  | chr6.240000001-245000000 | 2418686 | 2418720 |
|  | chr6.25000001-30000000 | 2064942 | 2064979 |
|  | chr6.25000001-30000000 | 4252905 | 4252953 |
|  | chr6.260000001-265000000 | 2848680 | 2848715 |
|  | chr6.260000001-265000000 | 4472838 | 4472877 |
|  | chr6.30000001-35000000 | 166499 | 166541 |
|  | chr6.35000001-40000000 | 689057 | 689090 |
|  | chr6.45000001-50000000 | 3979926 | 3979965 |
|  | chr6.5000001-10000000 | 1589692 | 1589726 |
|  | chr6.5000001-10000000 | 1850655 | 1850689 |
|  | chr6.60000001-65000000 | 1565855 | 1565890 |
|  | chr7.135000001-140000000 | 1993785 | 1993819 |
|  | chr7.150000001-155000000 | 1022650 | 1022685 |
|  | chr7.155000001-160000000 | 1125481 | 1125520 |
|  | chr7.175000001-180000000 | 4890091 | 4890126 |
|  | chr7.180000001-185000000 | 434367 | 434406 |
|  | chr7.180000001-185000000 | 452121 | 452155 |
|  | chr7.185000001-190000000 | 2477678 | 2477713 |
|  | chr7.205000001-210000000 | 1391351 | 1391384 |
|  | chr7.205000001-210000000 | 2954140 | 2954179 |
|  | chr7.210000001-215000000 | 2195807 | 2195836 |
|  | chr7.210000001-215000000 | 2461456 | 2461499 |
|  | chr7.220000001-225000000 | 2484138 | 2484177 |
|  | chr7.250000001-255000000 | 906099 | 906147 |
|  | chr7.35000001-40000000 | 3567309 | 3567343 |
|  | chr7.50000001-55000000 | 691880 | 691914 |
|  | chr7.95000001-100000000 | 181084 | 181118 |
|  | chr8.10000001-15000000 | 2224902 | 2224937 |
|  | chr8.120000001-125000000 | 676578 | 676616 |
|  | chr8.140000001-145000000 | 897321 | 897377 |
|  | chr8.150000001-155000000 | 3132571 | 3132611 |
|  | chr8.160000001-165000000 | 2283612 | 2283652 |
|  | chr8.170000001-175000000 | 1002396 | 1002431 |
|  | chr8.170000001-175000000 | 3553817 | 3553852 |
|  | chr8.180000001-185000000 | 3986449 | 3986489 |
|  | chr8.190000001-195000000 | 1361958 | 1361997 |
|  | chr8.200000001-205000000 | 3008753 | 3008788 |
|  | chr8.220000001-225000000 | 1297350 | 1297388 |
|  | chr8.225000001-230000000 | 3218780 | 3218812 |
|  | chr8.265000001-270000000 | 878113 | 878143 |
|  | chr8.265000001-270000000 | 2217294 | 2217329 |
|  | chr8.290000001-295000000 | 861448 | 861487 |
|  | chr8.290000001-295000000 | 1190524 | 1190564 |
|  | chr8.30000001-35000000 | 4363983 | 4364024 |
|  | chr8.35000001-40000000 | 3652534 | 3652569 |
|  | chr8.45000001-50000000 | 4025069 | 4025102 |
|  | chr8.85000001-90000000 | 1380358 | 1380397 |
|  | chr8.90000001-95000000 | 2531615 | 2531653 |
|  | chrX.10000001-15000000 | 4153197 | 4153242 |
|  | chrX.20000001-25000000 | 3116306 | 3116341 |
|  | chrX.30000001-35000000 | 3939359 | 3939407 |
|  | chrX.40000001-45000000 | 1017156 | 1017190 |
|  | chrX.40000001-45000000 | 2377428 | 2377462 |
|  | chrX.45000001-50000000 | 4406679 | 4406709 |
|  | chrX.50000001-55000000 | 3667176 | 3667213 |
|  | chrX.55000001-60000000 | 1464369 | 1464404 |
|  | chrX.55000001-60000000 | 1652828 | 1652864 |
|  | chrX.65000001-70000000 | 4761503 | 4761551 |
| ***Sarcophilus harrisii*** | GL834476.1 | 50987 | 51024 |
|  | GL834643.1 | 1354812 | 1354847 |
|  | GL834659.1 | 815135 | 815175 |
|  | GL834769.1 | 232285 | 232314 |
|  | GL834769.1 | 1268136 | 1268170 |
|  | GL835760.1 | 90782 | 90820 |
|  | GL841155.1 | 868282 | 868312 |
|  | GL841240.1 | 694764 | 694796 |
|  | GL841404.1 | 1340913 | 1340950 |
|  | GL841614.1 | 103848 | 103878 |
|  | GL841630.1 | 1232998 | 1233034 |
|  | GL842284.1 | 64460 | 64494 |
|  | GL849725.1 | 83307 | 83337 |
|  | GL849813.1 | 574816 | 574854 |
|  | GL849815.1 | 1180512 | 1180549 |
|  | GL849904.1 | 1726941 | 1726976 |
|  | GL849905.1 | 1548511 | 1548550 |
|  | GL849910.1 | 767869 | 767904 |
|  | GL849910.1 | 978386 | 978416 |
|  | GL856754.1 | 1251743 | 1251779 |
|  | GL856816.1 | 1523036 | 1523096 |
|  | GL856817.1 | 45614 | 45656 |
|  | GL856820.1 | 824309 | 824346 |
|  | GL856958.1 | 745372 | 745439 |
|  | GL856961.1 | 1817023 | 1817060 |
|  | GL856965.1 | 179748 | 179782 |
|  | GL861628.1 | 2443669 | 2443704 |
|  | GL864753.1 | 128352 | 128393 |
|  | GL864774.1 | 1043649 | 1043686 |
|  | GL864833.1 | 797882 | 797919 |
|  | GL864835.1 | 3317779 | 3317818 |
|  | GL864880.1 | 438759 | 438794 |
|  | GL864882.1 | 1345186 | 1345223 |
|  | GL867569.1 | 176976 | 177011 |
| ***Macropus eugenii*** | chr01 | 299272041 | 299272081 |
| ***Anolis carolinensis*** | chr1 | 24974212 | 24974246 |
|  | chr1 | 25038773 | 25038807 |
|  | chr1 | 27704085 | 27704119 |
|  | chr1 | 63574065 | 63574103 |
|  | chr1 | 66016856 | 66016896 |
|  | chr1 | 88066509 | 88066556 |
|  | chr1 | 96733416 | 96733453 |
|  | chr1 | 149495486 | 149495526 |
|  | chr1 | 153640222 | 153640277 |
|  | chr1 | 154886925 | 154886960 |
|  | chr1 | 195255585 | 195255620 |
|  | chr1 | 217331079 | 217331113 |
|  | chr1 | 229916944 | 229916979 |
|  | chr1 | 249627761 | 249627793 |
|  | chr2 | 3320143 | 3320198 |
|  | chr2 | 6348847 | 6348954 |
|  | chr2 | 11862257 | 11862294 |
|  | chr2 | 18518316 | 18518348 |
|  | chr2 | 28540018 | 28540052 |
|  | chr2 | 45507543 | 45507576 |
|  | chr2 | 49743051 | 49743094 |
|  | chr2 | 58690496 | 58690533 |
|  | chr2 | 60733702 | 60733765 |
|  | chr2 | 71376340 | 71376372 |
|  | chr2 | 81092873 | 81092905 |
|  | chr2 | 98638519 | 98638562 |
|  | chr2 | 142858929 | 142858979 |
|  | chr2 | 158231185 | 158231220 |
|  | chr2 | 182307196 | 182307230 |
|  | chr2 | 184914053 | 184914086 |
|  | chr2 | 197093582 | 197093617 |
|  | chr3 | 8592256 | 8592293 |
|  | chr3 | 18557780 | 18557817 |
|  | chr3 | 41352061 | 41352098 |
|  | chr3 | 52262283 | 52262318 |
|  | chr3 | 52937748 | 52937801 |
|  | chr3 | 53462594 | 53462630 |
|  | chr3 | 69591064 | 69591154 |
|  | chr3 | 90822287 | 90822319 |
|  | chr3 | 128633821 | 128633866 |
|  | chr3 | 130543150 | 130543186 |
|  | chr3 | 130741669 | 130741704 |
|  | chr3 | 161147465 | 161147520 |
|  | chr4 | 16371425 | 16371460 |
|  | chr4 | 20336346 | 20336381 |
|  | chr4 | 71785036 | 71785079 |
|  | chr4 | 78526346 | 78526381 |
|  | chr4 | 82775223 | 82775260 |
|  | chr4 | 83545704 | 83545760 |
|  | chr4 | 112276940 | 112276970 |
|  | chr4 | 122386671 | 122386703 |
|  | chr4 | 122575416 | 122575449 |
|  | chr4 | 155735328 | 155735372 |
|  | chr5 | 7871991 | 7872024 |
|  | chr5 | 59254593 | 59254627 |
|  | chr5 | 60335013 | 60335068 |
|  | chr5 | 76233023 | 76233076 |
|  | chr5 | 132648786 | 132648821 |
|  | chr5 | 133129403 | 133129438 |
|  | chr6 | 27881914 | 27881948 |
|  | chr6 | 50774973 | 50775006 |
|  | chr6 | 51732397 | 51732432 |
|  | chr6 | 56324285 | 56324323 |
|  | chr6 | 79385809 | 79385847 |
