# Supplementary material for: Ancient Traces of Tailless Retropseudogenes in Therian Genomes
Source: Genome Biol Evol. 2015 Feb 26;7(3):889–900. doi: 10.1093/gbe/evv040 (PMC5322556; doi:10.1093/gbe/evv040)
Supplement: Supplementary Data [file supp_evv040_Table_S7-S12.docx]

**Table S7. Tailless retropseudogenes in plants**

Coordinates of U2-derived tailless retropseudogenes in plants (*Zea mays*).

| Chr | Start | End |
| --- | --- | --- |
| chr01 | 11916335 | 11916440 |
| chr01 | 89503678 | 89503786 |
| chr01 | 264808454 | 264808569 |
| chr01 | 268528097 | 268528164 |
| chr03 | 223672290 | 223672394 |
| chr05 | 138070741 | 138070812 |
| chr06 | 2059315 | 2059377 |
| chr08 | 109250846 | 109250952 |

**Table S8. Used genomes**

Versions and lengths of genomes used for tailless retropseudogene screening.

| **Species** | | **Version** | **Length** | **Length without N/X** |
| --- | --- | --- | --- | --- |
| Human | *Homo sapiens* | hg19 | 3,095,712,089 | 2,861,361,912 |
| Cattle | *Bos taurus* | 3.1 | 2,660,906,405 | 2,640,174,894 |
| Dog | *Canis lupus familiaris* | 3.1 | 1,398,303,923 | 1,392,182,617 |
| Mouse | *Mus musculus* | 10 | 2,725,521,370 | 2,647,521,528 |
| African savanna elephant | *Loxodonta africana* | 3.0 | 3,118,548,474 | 3,118,548,474 |
| Hoffmann’s two-fingered sloth | *Choloepus hoffmanni* | 1.0 | 2,467,493,193 | 2,060,419,685 |
| Gray short-tailed opossum | *Monodelphis domestica* | 5.0 | 3,502,373,038 | 3,412,622,278 |
| Tasmanian devil | *Sarcophilus harrisii* | 7.0 | 3,174,693,010 | 2,932,081,930 |
| Tammar wallaby | *Macropus eugenii* | 1.1 | 2,255,262,693 | 163,916,531 |
| Platypus | *Ornithorhynchus anatinus* | 5.0.1 | 417,111,767 | 390,788,187 |
| Carolina anole | *Anolis carolinensis* | 2.0 | 1,081,644,591 | 1,026,047,551 |
| Chicken | *Gallus gallus* | 4.0 | 991,081,418 | 948,004,525 |
| Arabidopsis | *Arabidopsis thaliana* | 10 | 119,146,348 | 118,960,744 |
| Rice | *Oryza sativa* | 8.0 | 374,471,240 | 374,306,765 |
| Maize | *Zea mays* | 1 | 1,996,997,808 | 1,983,832,998 |

**Table S9. Table 5S rRNAs**

The table contains information about the 5S rRNA target sequence used for each analyzed species. The accession number, the Consensus60 sequence or a reference to the RepeatMasker Library of GIRI is given.

| Species | source |
| --- | --- |
| *Homo sapiens* | X51545 |
| *Bos taurus* | Vertebrate 5S rRNA sequence of Giri |
| *Canis lupus familiaris* | Consensus60  GTCTACGGCCATACCACCCTGAACGCNCCCGATCTCGTCTGATCTCGGAAGCTAAGCAGGGTCGGGCCTGGTTAGTACTTGGATGGGAGACCGCCTGGGAATACCGGGTGCTGTAGGCTTT |
| *Mus musculus* | Consensus60 of NR_046119-NR_046120, NR_046122-NR_046126, NR_046128-NR_046120-NR_046130, NR_046132-NR_046134, NR_046141, NR_046143-NR_046146, NR_046148-NR_046151, NR_046153, NR_046156 |
| *Loxodonta africana* | Consensus60  GTCTACGGCCATACCACCCTGAACGMGCCCGATCTCRTCTGATCTCGGAAGCTAAGCAGGGTMGGGCCTGGTTAGTACTTGGATGGGAGACCGCCTGGGAATACCRGGTGCTGTAGGCTTT |
| *Choloepus hoffmanni* | Consensus60  GTCTACGGCCATACCACCCTGAACGCGCCCGATCTCGTCTGATCTCGGAAGCTAAGCAGGGTCGGGCCTGGTTAGTACTTGGATGGGAGACCGCCTGGGAATACCGGGTGCTGTAGGCTTT |
| *Monodelphis domestica* | Vertebrate 5S rRNA sequence of Giri |
| *Sarcophilus harrisii* | Consensus60  GTCTACGGCCATACCACCCTGAATGCACCCGATCTCMTCTGATCTCGGAAGCTAAGCAGGGTCGGGCCGTGGTTAGTACTTGGATGGGAGACCGCCTGGGAATACCGGGTGCTGTAGGCTTT |
| *Macropus eugenii* | Consensus60  GTCTACAGCCATACCACCCTGAACGCGCCTGATCTCGTCTGATCTCGGAAGCTAAGCAGGGTCGGGCCTGGTTAGTACTTGGATGGGAGACTWCCTGGGAATACCAGGTGCTGTAAGCTTT |
| *Ornithorhynchus anatinus* | Vertebrate 5S rRNA sequence of Giri |
| *Anolis carolinensis* | Consensus60  GTCTACGGCCATACCACCCTGAACACGCCCGATCTCGTCTGATCTCGGAAGCTAAGCAGGGTCGGGCCTGGTTAGTACTTGGATGGGAGACCGCCTGGGAATACCGGGTGCTGTAGGCTTT |
| *Gallus gallus* | Consensus60  GCCTACGGCCATCCCACCCTGGTAACGCCCGATCTCGTCTGATCTCGGAAGCTAAGCAGGGTCGGGCCTGGTTAGTACTTGGATGGGAGACCTCCTGGGAATACCGGGTGCTGTAGGCTTT |
| *Arabidopsis thaliana* | AF330992 |
| *Oryza sativa* | Consensus60 with blast of *A. thaliana* 5S sequence  GGATGCGATCATACCAGCACTAAAGCACCGGATCCCATCAGAACTCCGAAGTTAAGCGTGCTTGGGCGAGAGTAGTACTAGGATGGGTGACCTCCTGGGAAGTCCTCGTGTTGCATCCCTC |
| *Zea mays* | Consensus60 with blast of *A. thaliana* 5S sequence  CGGATGCGATCATACCAGCACTAAAGCACCGGATCCCATCAGAACTCCGAAGTTAAGCGTGCTTGGGCGAGAGTAGTACTAGGATGGGTGACCTCCTGGGAAGTCCTCGTGTTGCATTCCT |

**Table S10. U2 snRNA**

The table contains information about the U2 snRNA target sequence used for each analyzed species. The accession number, the Consensus60 sequence or a reference to the RepeatMasker Library of GIRI is given.

| Species | source |
| --- | --- |
| *Homo sapiens* | K03022 |
| *Bos taurus* | Consensus60  ATCGCTTCTCGGCCTTTTGGCTAAGATCAAGTGTAGTATCTGTTCTTATCAGTTTAATATCTGATACGTCCTCTATCCGAGGACAATATATTAAATGGATTTTTGGAGCAGGGAGTTGGAATAGGAGCTTGCTCCGTCCACTCCACGCATCGACCTGGTATTGCAGTACTTCCAGGAACGGTGCACC |
| *Canis lupus familiaris* | Consensus60  ATCGCTTCTCGGCCTTTTGGCTAAGATCAAGTGTAGTATCTGTTCTTATCAGTTTAATATCTGATACGTCCTCTATCCGAGGACAATATATTAAATGGATTTTTGGAGCAGGGAGATGGAATAGGAGCTTGCTCCGTCCACTCCGCGCATCGACCTGGTATTGCAGTACCTCCAGGAACGGTGCACC |
| *Mus musculus* | Consensus60  ATCGCTTCTCGGCCTTTTGGCTAAGATCAAGTGTAGTATCTGTTCTTATCAGTTTAATATCTGATACGTCCTCTATCCGAGGACAATATATTAAATGGATTTTTGGAAGTAGGAGTTGGAATAGGAGCTTGCTCCGTCCACTCCACGCATCGACCTGGTATTGCAGTACCTCCAGGAACGGTGCACC |
| *Loxodonta africana* | Consensus60  ATCGCTTCTCGGCCTTTTGGCTAAGATCAAGTGTAGTATCTGTTCTTATCAGTTTAATATCTGATACGTCCTCTATCCGAGGACAATATATTAAATGGATTTTTGGAGCTGGGAGWTGGAATAGGAGCTTGCTCCGTCCACTCCACGCATCGACCTGGTATTGCAGTACYTCCAGGAACGGTGCACC |
| *Choloepus hoffmanni* | Consensus60  ATCGCTTCTCGGCCTTTTGGCTAAGATCAAGTGTAGTATCTGTTCTTATCAGTTTAATATCTGATACGTCCTCTATCCGAGGACAACATATTAAACGGATTTTTGGAGCTGGGAGTCGGAATAGGAGCTTGCTCCGTCCACTCCACGCATCGACCTGGTATTGCAGTGCTTCCGGGAACGGTGCACC |
| *Monodelphis domestica* | Consensus60  ATCGCTTCTCGGCCTTTTGGCTAAGATCAAGTGTAGTATCTGTTCTTATCAGTTTAATATCTGATACGTCCTCTATCCGAGGACAATATATTAAATGGATTTTTGAAACAGGGAGTCGGAATAGGAGCTTGCTCCGTCCACTCCACGCATCGACCTGGTATTGCAGTACTTCCAGGAACGGTGCAC |
| *Sarcophilus harrisii* | Consensus60  ATCGCTTCTCGGCCTTTTGGCTAAGATCAAGTGTAGTATCTGTTCTTATCAGTTTAATATCTGATACGTCCTCTATCCGAGGACAATATATTAAATGGATTTTTGGAMCTGGGAGATGGAATAGGAGCTTGCTCCGTCCACTCCGCGCATCGACCCGGTATTGCAGTACTTCCGGGAACGGTGCACC |
| *Macropus eugenii* | Consensus60  ATCGCTTCTCGGCCTTTTGGCTAAGATCAAGTGTAGTATCTGTTCTTATCAGTTTAATATCTGATACGTCCTCTATCCGAGGACAATATATTAAATGGATTTTTGGAGCAGGGAGTCGGAATAGGAGCTTGCTCCGTCCACTCCACGCATCGACCCGGTATTGCAGTACTTCCGGGAACGGTGCACC |
| *Ornithorhynchus anatinus* | Consensus60  ATCGCTTCTCGGCCTTTTGGCTAAGATCAAGTGTAGTATCTGTTCTTATCAGTTTAATATCTGATACGTCCTCTATCCGAGGACAATATATTAAATGGATTTTTGGAGCCGGGAGATGGAATAGGGGCTTGCTCCGTCCACTCCACGCATCGACCTGGTATTGCAGTACTTCCAGGAACGGTGCACC |
| *Anolis carolinensis* | Consensus60  ATCGCTTCTCGGCCTTTTGGCTAAGATCAAGTGTAGTATCTGTTCTTATCAGTTTAATATCTGATACGTCCTCTATTTGAGGACTATATATTAAATGGATTTTTGGGCCTGGGAGATGGAATAGGGGCTTGCTCCATCCACTCCACGCATCGACCTGGTATTGCAGTGCCTCCAGGAACGGTGCACC |
| *Gallus gallus* | Vertebrate U2 snRNA sequence of Giri |
| *Arabidopsis thaliana* | X06474 |
| *Oryza sativa* | Consensus60 with blast of *A. thaliana* U2 sequence  ATACCTTTCTCGGCCTTTTGGCTAAGATCAAGTGTAGTATCTGTTCTTATCAGTTTAATATCTGATATGTGGGCCATGTGYCYACTTTGATATTAAATTTATTTTTYGTGGGGGAGRGTCCACCATAGTGGCTTGCCACTAGGGCCCTCRTGTGTCGCCTAGGCGTTGCACTACAGCCTTGGCTGGYGCACCCC |
| *Zea mays* | X16459 |

**Table S11. Smith Waterman substitution matrix**

Empirical substitution matrix used for the Smith Waterman algorithm.

|  | **A** | **C** | **G** | **T** | **N** | **R** | **Y** | **S** | **W** | **K** | **M** | **D** | **H** | **B** | **V** |
| --- | --- | --- | --- | --- | --- | --- | --- | --- | --- | --- | --- | --- | --- | --- | --- |
| **A** | 8 | -17 | -17 | -17 | 0 | 4 | -17 | -17 | 4 | -17 | 4 | 2 | 2 | -17 | 2 |
| **C** | -17 | 8 | -17 | -17 | 0 | -17 | 4 | 4 | -17 | -17 | 4 | -17 | 2 | 2 | 2 |
| **G** | -17 | -17 | 8 | -17 | 0 | 4 | -17 | 4 | -17 | 4 | -17 | 2 | -17 | 2 | 2 |
| **T** | -17 | -17 | -17 | 8 | 0 | -17 | 4 | -17 | 4 | 4 | -17 | 2 | 2 | 2 | -17 |
| **N** | 0 | 0 | 0 | 0 | 0 | 0 | 0 | 0 | 0 | 0 | 0 | 0 | 0 | 0 | 0 |
| **R** | 4 | -17 | 4 | -17 | 0 | 4 | -17 | 2 | 2 | 2 | 2 | 3 | 1 | 1 | 3 |
| **Y** | -17 | 4 | -17 | 4 | 0 | -17 | 4 | 2 | 4 | 4 | 4 | 1 | 3 | 3 | 1 |
| **S** | -17 | 4 | 4 | -17 | 0 | 2 | 2 | 3 | -17 | 2 | 2 | 1 | 1 | 3 | 3 |
| **W** | 4 | -17 | -17 | 4 | 0 | 3 | 3 | -17 | 4 | 2 | 2 | 3 | 3 | 1 | 1 |
| **K** | -17 | -17 | 4 | 4 | 0 | 2 | 2 | 2 | 2 | 4 | -17 | 3 | 1 | 3 | 1 |
| **M** | 4 | 4 | -17 | -17 | 0 | 2 | 2 | 2 | 2 | -17 | 4 | 1 | 3 | 1 | 3 |
| **D** | 2 | -17 | 2 | 2 | 0 | 2 | 1 | 1 | 2 | 2 | 1 | 3 | 2 | 2 | 2 |
| **H** | 2 | 2 | -17 | 2 | 0 | 1 | 2 | 1 | 1 | 2 | 2 | 2 | 3 | 2 | 2 |
| **B** | -17 | 2 | 2 | 2 | 0 | 1 | 2 | 2 | 1 | 2 | 1 | 2 | 2 | 3 | 2 |
| **V** | 2 | 2 | -17 | 2 | 0 | 2 | 1 | 2 | 1 | 1 | 2 | 2 | 2 | 2 | 3 |

**Table S12. Needleman Wunsch substitution matrix**

Empirical substitution matrix used for the Needleman Wunsch algorithm.

|  | **A** | **C** | **G** | **T** | **N** | **R** | **Y** | **S** | **W** | **K** | **M** | **D** | **H** | **B** | **V** |
| --- | --- | --- | --- | --- | --- | --- | --- | --- | --- | --- | --- | --- | --- | --- | --- |
| **A** | 7 | -5 | -5 | -5 | 0 | 4 | -7 | -7 | 4 | -7 | 4 | 2 | 2 | -7 | 2 |
| **C** | -5 | 7 | -5 | -5 | 0 | -7 | 4 | 4 | -7 | -7 | 4 | -7 | 2 | 2 | 2 |
| **G** | -5 | -5 | 7 | -5 | 0 | 4 | -7 | 4 | -7 | 4 | -7 | 2 | -7 | 2 | 2 |
| **T** | -5 | -5 | -5 | 7 | 0 | -7 | 4 | -7 | 4 | 4 | -7 | 2 | 2 | 2 | -7 |
| **N** | 0 | 0 | 0 | 0 | 0 | 0 | 0 | 0 | 0 | 0 | 0 | 0 | 0 | 0 | 0 |
| **R** | 4 | -7 | 4 | -7 | 0 | 4 | -7 | -7 | -7 | -7 | -7 | -7 | -7 | -7 | -7 |
| **Y** | -7 | 4 | -7 | 4 | 0 | -7 | 4 | -7 | -7 | -7 | -7 | -7 | -7 | -7 | -7 |
| **S** | -7 | 4 | 4 | -7 | 0 | -7 | -7 | 4 | -7 | -7 | -7 | -7 | -7 | -7 | -7 |
| **W** | 4 | -7 | -7 | 4 | 0 | -7 | -7 | -7 | 4 | -7 | -7 | -7 | -7 | -7 | -7 |
| **K** | -7 | -7 | 4 | 4 | 0 | -7 | -7 | -7 | -7 | 4 | -7 | -7 | -7 | -7 | -7 |
| **M** | 4 | 4 | -7 | -7 | 0 | -7 | -7 | -7 | -7 | -7 | 4 | -7 | -7 | -7 | -7 |
| **D** | 2 | -7 | 2 | 2 | 0 | -7 | -7 | -7 | -7 | -7 | -7 | 2 | -7 | -7 | -7 |
| **H** | 2 | 2 | -7 | 2 | 0 | -7 | -7 | -7 | -7 | -7 | -7 | -7 | 2 | -7 | -7 |
| **B** | -7 | 2 | 2 | 2 | 0 | -7 | -7 | -7 | -7 | -7 | -7 | -7 | -7 | 2 | -7 |
| **V** | 2 | 2 | 2 | -7 | 0 | -7 | -7 | -7 | -7 | -7 | -7 | -7 | -7 | -7 | 2 |
